# Characterization of Epstein-Barr Virus miRNAome in Nasopharyngeal Carcinoma by Deep Sequencing

**DOI:** 10.1371/journal.pone.0012745

**Published:** 2010-09-20

**Authors:** Shu-Jen Chen, Gian-Hung Chen, Yi-Hsuan Chen, Cheng-Yuan Liu, Kai-Ping Chang, Yu-Sun Chang, Hua-Chien Chen

**Affiliations:** 1 Department of Life Science, Chang Gung University, Taoyuan, Taiwan, Republic of China; 2 Molecular Medicine Research Center, Chang Gung University, Taoyuan, Taiwan, Republic of China; 3 Department of Otolaryngology, Chang Gung Memorial Hospital at Lin-Kou, Taoyuan, Taiwan, Republic of China; University of Hong Kong, Hong Kong

## Abstract

Virus-encoded microRNAs (miRNAs) have been shown to regulate a variety of biological processes involved in viral infection and viral-associated pathogenesis. Epstein-Barr virus (EBV) is a herpesvirus implicated in nasopharyngeal carcinoma (NPC) and other human malignancies. EBV-encoded miRNAs were among the first group of viral miRNAs identified. To understand the roles of EBV miRNAs in the pathogenesis of NPC, we utilized deep sequencing technology to characterize the EBV miRNA transcriptome in clinical NPC tissues. We obtained more than 110,000 sequence reads in NPC samples and identified 44 EBV BART miRNAs, including four new mature miRNAs derived from previously identified BART miRNA precursor hairpins. Further analysis revealed extensive sequence variations (isomiRs) of EBV miRNAs, including terminal isomiRs at both the 5′ and 3′ ends and nucleotide variants. Analysis of EBV genomic sequences indicated that the majority of EBV miRNA nucleotide variants resulted from post-transcriptional modifications. Read counts of individual EBV miRNA in NPC tissue spanned from a few reads to approximately 18,000 reads, confirming the wide expression range of EBV miRNAs. Several EBV miRNAs were expressed at levels similar to highly abundant human miRNAs. Sequence analysis revealed that most of the highly abundant EBV miRNAs share their seed sequences (nucleotides 2–7) with human miRNAs, suggesting that seed sequence content may be an important factor underlying the differential accumulation of BART miRNAs. Interestingly, many of these human miRNAs have been found to be dysregulated in human malignancies, including NPC. These observations not only provide a potential linkage between EBV miRNAs and human malignancy but also suggest a highly coordinated mechanism through which EBV miRNAs may mimic or compete with human miRNAs to affect cellular functions.

## Introduction

A unique feature of nasopharyngeal carcinoma (NPC) is its strong association with Epstein-Barr Virus (EBV) [1]. EBV genome can be detected in all cases of NPC, suggesting that products of EBV genome are involved in the pathogenesis of this malignancy. In addition to EBV-encoded protein-coding genes such as EBNA1 and LMP1, latently infected NPC cells and tissues also express high levels of non-coding EBV RNAs, including EBER1, EBER2 and multiple microRNAs (miRNAs) [2], [3], [4], [5]. EBV miRNAs were first identified by Pfeffer et al. via cloning of small RNAs from a Burkitt's lymphoma cell line latently infected with EBV [6]. The authors identified six mature miRNAs derived from five miRNA precursors in the EBV genome. Grundhoff et al. subsequently identified 18 new precursors that produce 22 mature miRNAs in EBV-infected Jijoye and BJAB cells by combining computational and microarray approach [7]. Cai et al. also identified additional EBV miRNAs from EBV-positive BC-1 cells and demonstrated that the EBV miRNAs were conserved during lymphocryptovirus evolution [8]. Recently, Zhu et al. used the Roche 454 system to sequence ∼2,000 reads of EBV miRNAs in NPC tissues and discovered five novel mature EBV miRNAs [9]. To date, a total of 25 EBV miRNA precursors with 44 mature miRNAs have been reported [10].

These EBV miRNA precursors are clustered in two regions of the EBV genome. Three precursors (miR-BHRF1-1 to miR-BHRF1-3) with four mature miRNAs are located within the mRNA of the BHRF1 (Bam HI fragment H rightward open reading frame 1) gene. An additional twenty-two precursors (miR-BART1 to miR-BART22) with forty mature miRNAs are located in intronic regions of the BART (Bam HI-A region rightward transcript) transcript [6], [7], [8], [9]. Studies using Northern blotting and cloning methods have clearly documented two distinct expression patterns of EBV miRNAs in EBV-infected B lymphoma cells and EBV-positive NPC cell lines [6]. The expression of BHRF1 miRNAs can be detected only in lytically infected cells, whereas expression of BART miRNAs can be detected in all forms of latency. Recent studies by Cosmopoulos et al. using real-time PCR technology confirmed the expression patterns observed in cell lines and provided further insights into the wide expression range of BART miRNAs in NPC clinical samples [11].

Recent studies imply that EBV miRNAs may be linked to the pathogenesis of NPC. Functional studies revealed that these viral miRNAs regulate EBV latent infection and modulate host immune responses by targeting a variety of viral and cellular genes. For example, BART2-5p miRNA has been shown to inhibit EBV lytic replication by targeting the viral DNA polymerase BALF5 [12], while BART1-5p, BART16-5p and BART17-5p have been found to suppress the expression of the viral oncoprotein LMP1 [13]. On the other hand, BHRF1-3 has been shown to modulate host immune responses by targeting the interferon-induced chemokine CXCL11 [14], while BART2-5p targets the host stress-induced Natural Killer cell ligand, MICB, to escape recognition and consequent elimination by NK cells [15]. Finally, BART5-5p has been shown to promote host cell survival by targeting a pro-apoptotic molecule, p53 up-regulated modulator of apoptosis (PUMA) [16]. Together, these observations indicate that EBV virus actively utilizes its miRNAs to flexibly manipulate various viral and cellular functions.

To better understand the EBV miRNA transcriptome in NPC, we took advantage of the massive parallel sequencing capability of the SOLiD system and sequenced more than 110,000 reads of EBV miRNAs in one pair of a clinical NPC tumor and its adjacent normal samples. The abundance of sequence data allowed us to thoroughly and confidently characterize these EBV miRNAs, to determine their sequence and to assess their expression levels *in vivo*. We (1) identified four new mature miRNAs derived from known BART precursor hairpins; (2) observed extensive sequence variations [17], including length heterogeneity and nucleotide variations of EBV miRNAs and the wide expression range; some of these variants are expressed at substantial levels and generate additional seed sequences for EBV miRNAs; (3) revealed that most of the highly abundant EBV miRNAs share their seed sequences with human miRNA, suggesting a connection between seed sequence content and the differential accumulation of these miRNAs. More interestingly, several human miRNAs sharing seed sequences with EBV miRNAs were found to be dysregulated in NPC tissues, providing a potential linkage between EBV miRNAs and human malignancy. Collectively, these data present a comprehensive profiling of EBV miRNAs in a disease context and implicate their pathological significance.

## Materials and Methods

### Clinical samples

Biopsy samples of NPC and adjacent normal nasopharynx tissue were obtained from patients undergoing surgery. Tissues were frozen immediately after surgical resection. Tissue specimens were collected in accordance with the Institutional Regulation Board of Chang Gung Memorial Hospital, Taiwan.

### Cell lines

Two EBV-positive NPC cell lines, c666-1 and HK1-EBV, and an EBV-negative NPC cell line, HK1, were kindly provided by Dr. George Tsao. B95.8, Daudi and Namalwa are EBV-positive Burkitt's lymphoma cell lines. All cell lines were maintained in RPMI-1640 medium supplemented with 10% fetal calf serum, 2 mM sodium pyruvate and 2 mM L-glutamate.

### Preparation of total RNA

Total RNA from tissues and cultured cells was prepared using TRIzol reagent (Invitrogen, Carlsbad, CA, USA) according to the manufacturer's protocol. The concentration of purified RNA was determined using a NanoDrop Spectrophotometer. The integrity of the RNA was evaluated using an Agilent 2100 BioAnalyzer (Agilent Technologies, Palo Alto, CA, USA). Aliquots of total RNA were used directly for miRNA quantification using stem-loop RT-PCR or subjected to small RNA library construction.

### Small RNA library preparation

A small RNA library was prepared using the SOLiD small RNA expression kit according to the manufacturer's protocol. In brief, small RNA was purified by polyacrylamide gel electrophoresis to enrich for molecules in the range of 18–40 nt. One hundred nanograms of small RNA was ligated to adaptors to the 5′ and 3′ termini of the RNA. Ligated RNA was converted to cDNA, then digested with RNAse H followed by PCR amplification. Amplified cDNA libraries were purified on a 6% polyacrylamide gel and quantified. The cDNA template (0.1 µg/uL) was subjected to emulsion PCR. Positive beads were enriched and deposited onto slides. The cDNAs from these libraries were sequenced using standard conditions for the SOLiD system by Mission Biotech (Taipei, Taiwan).

### Processing and analysis of deep sequencing data

Sequencing results were aligned to the reference human genome (NCBI Build 36) and EBV genome (NC_007605) using standard SOLiD mapping pipeline, allowing up to six mismatches out of 34 bp. All identical sequences were compiled to generate a data set of the unique sequences with associated read counts. Only unique sequences with read count greater than 2 were included for subsequent annotation and analysis. Annotation of human and EBV miRNA was based on the sequences registered in miRBase (version 13.0, http://www.mirbase.org/). Uniquely aligned sequences with corresponding read counts were imported into Excel to analyze terminal heterogeneity and nucleotide variation. Evolutionary conservation of miRNA was analyzed using the online miRviewer service (http://people.csail.mit.edu/akiezun/miRviewer/). Sequence logos were generated using the online WebLogo sequence logo generator (http://weblogo.berkeley.edu/logo.cgi).

### Stem-loop real-time PCR

The design of the stem-loop RT primer was adopted from the TaqMan miRNA assay described by Chen et al. [18]. For miRNA quantification, each RT primer contains a stem-loop sequence to enhance the binding specificity for mature miRNA and an 8-nt overhang at the 3′ end that complements the 3′ end of individual miRNA to prime the RT reaction. In some assays, 6-nt overhang RT primers were used to analyze 3′ terminal variants. EBV BART miRNAs were reverse transcribed using a pulsed reverse transcription reaction as described previously [18]. For each assay, 1 µg of total RNA was converted into cDNA in a 10-µl reaction mixture containing miRNA-specific stem-loop RT primers (final 2 nM each), 500 µM dNTP, and 0.5 µl Superscript III (Invitrogen, Carlsbad, CA). The pulsed RT reaction was performed as follows: 16°C for 30 min, followed by 50 cycles at 20°C for 30 s, 42°C for 30 s and 50°C for 1 s. RT products were diluted 20 fold and used as the template in a 10 µl PCR reaction containing 1X SYBR Master Mix (Applied Biosystems, Foster City, CA), 200 nM miRNA-specific forward primer, and 200 nM universal reverse primer. Real-time PCR was performed with the following conditions: 95°C for 10 min, followed by 40 cycles of 95°C for 15 s and 63°C for 32 s, and a dissociation stage. Synthetic miRNAs were reverse transcribed and amplified using the same conditions to generate standard curves for copy number calculation. All primers used are listed in the Table S1.

## Results

### EBV miRNA profiling and characterization in paired NPC tumor and adjacent normal tissues

To profile EBV-encoded miRNAs in NPC, we used the ABI SOLiD deep sequencing technology to characterize the EBV miRNAome in clinical NPC tissues. The NPC biopsy sample (T10) was taken from the nasopharynx area with clinical evidence of tumor. Clinically normal nasopharyngeal mucosa taken from the same patient was used as the control tissue (N10). A total of 1.92×10^7^ and 2.36×10^7^ qualified reads were obtained from the NPC tissue (T10) and the normal sample (N10), respectively (Table 1). Among these reads, 5.87×10^6^ (∼31%) from the T10 sample and 7.89×10^6^ (∼33%) from the N10 sample could be mapped to the human and EBV reference genome. After filtering out tRNAs, rRNAs and genomic repeats, the remaining mappable reads in the T10 sample contain 3.78×10^5^ reads corresponding to 570 known human miRNA hairpins. In the N10 sample, 1.42×10^6^ reads were mapped to 657 known human miRNA hairpins. Compared to N10, the number of reads mapped to known human miRNAs was much lower in T10. This result is consistent with our previous observation that the expression level of human miRNAs was significantly reduced in NPC samples compared to normal tissues [19]. A global downregulation of mature miRNAs in tumors compared with normal tissues has been observed in many types of human cancer [20]. The global downregulation of miRNAs in tumors is likely due to a decrease in Dicer level in tumor samples [21], [22].

**Table 1:** Overview of small RNAs detected by SOLiD sequencing.

Read Type		T10 (NPC)	N10 (normal)
Total		19,254,966	23,605,814
Mapped to human & EBV genome		5,866,413	7,891,903
Mapped to human genome		5,733,273	7,888,388
	human filters (tRNAs, rRNA, etc)	4,581,692	6,285,886
	human miRNA (miRBase 12.0)	378,017	1,418,266
	human others	1,151,581	1,602,502
Mapped to EBV genome		133,140	3,515
	EBV miRNA (miRBase 12.0)	113,901	2,027
	EBV others	19,239	1,488
human miRNAs + EBV miRNAs		491,918	1,420,293

**Figure 1:**
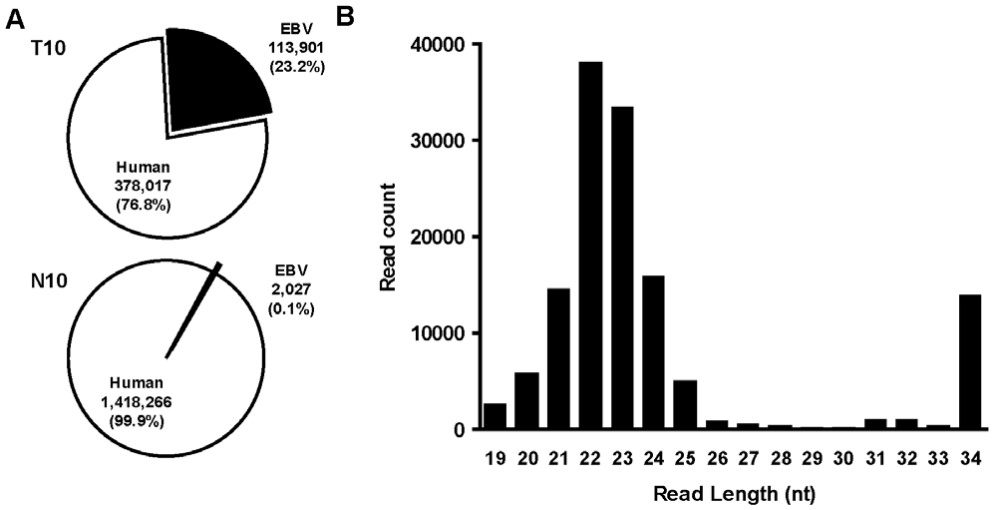
Overview of human and EBV miRNAs detected in NPC samples. (A). Proportion of human and EBV miRNAs detected in T10 and N10 libraries. Read counts represent total reads mapped to miRBase-registered precursor hairpins for human and EBV miRNAs. (B). Size distribution of EBV small RNAs detected in T10 sample.

EBV miRNA reads (1.33×10^5^ reads) constitute about 23.3% of total miRNA reads in the T10 sample, indicating that viral miRNAs take up a considerable portion of the miRNA biogenesis machinery in NPC cells (Figure 1A). Among the 1.33×10^5^ EBV miRNA reads, 85.5% of them were mapped to known EBV miRNAs. In contrast, only 2.03×10^3^ reads (approximately 0.1% of total miRNAs) from N10 were mapped to known EBV miRNAs. As the RNA samples used in this study were not obtained by laser-capture microdissection, the low level of EBV miRNAs detected in the normal tissue is likely due to minor contamination of tumor cells within the adjacent normal tissue. However, we could not exclude the possibility that EBV-infected cells were present in normal tissue adjacent to NPC. Our subsequent analysis focused mainly on EBV miRNA reads detected in the T10 NPC sample.

The EBV small RNA reads detected in the T10 sample can be separated into two distinct peaks based on the sequence length. The major peak, spanning from 20 to 25 nts and centered at 22 nt, correlated well with the size of mature miRNAs. A second peak centered at 34 nt contained more than 90% of reads derived from two EBV non-coding RNAs, EBER1 and EBER2 (Figure 1B). These reads were likely derived from contamination of EBERs in the small RNA library rather than microRNAs derived from the EBER regions. First, the read length (34 nt) was much longer than the size of mature miRNAs (∼22 nt). Second, no hairpin structure could be detected in these regions to demonstrate the structural features that define a microRNA.

Although the EBV genome can be transcribed from both directions, more than 99% of the EBV small RNA reads were mapped to positive strand transcripts. Genomic locations of all EBV small RNA reads in T10 are shown in Figure S1. These EBV small RNAs were clustered in two genomic regions, the EBER locus (6.6 kb–7.2 kb) and the BART locus (139 kb–152 kb). A few small RNA reads were mapped to previously unannotated regions, including the 70 kb and 153 kb regions of the EBV genome. No sequencing read was mapped to the region of BHRF1 transcripts. These results are in agreement with the recent study of EBV miRNAs in NPC tissues using the Roche 454 technology [9].

### Identification of novel EBV miRNAs

**Figure 2:**
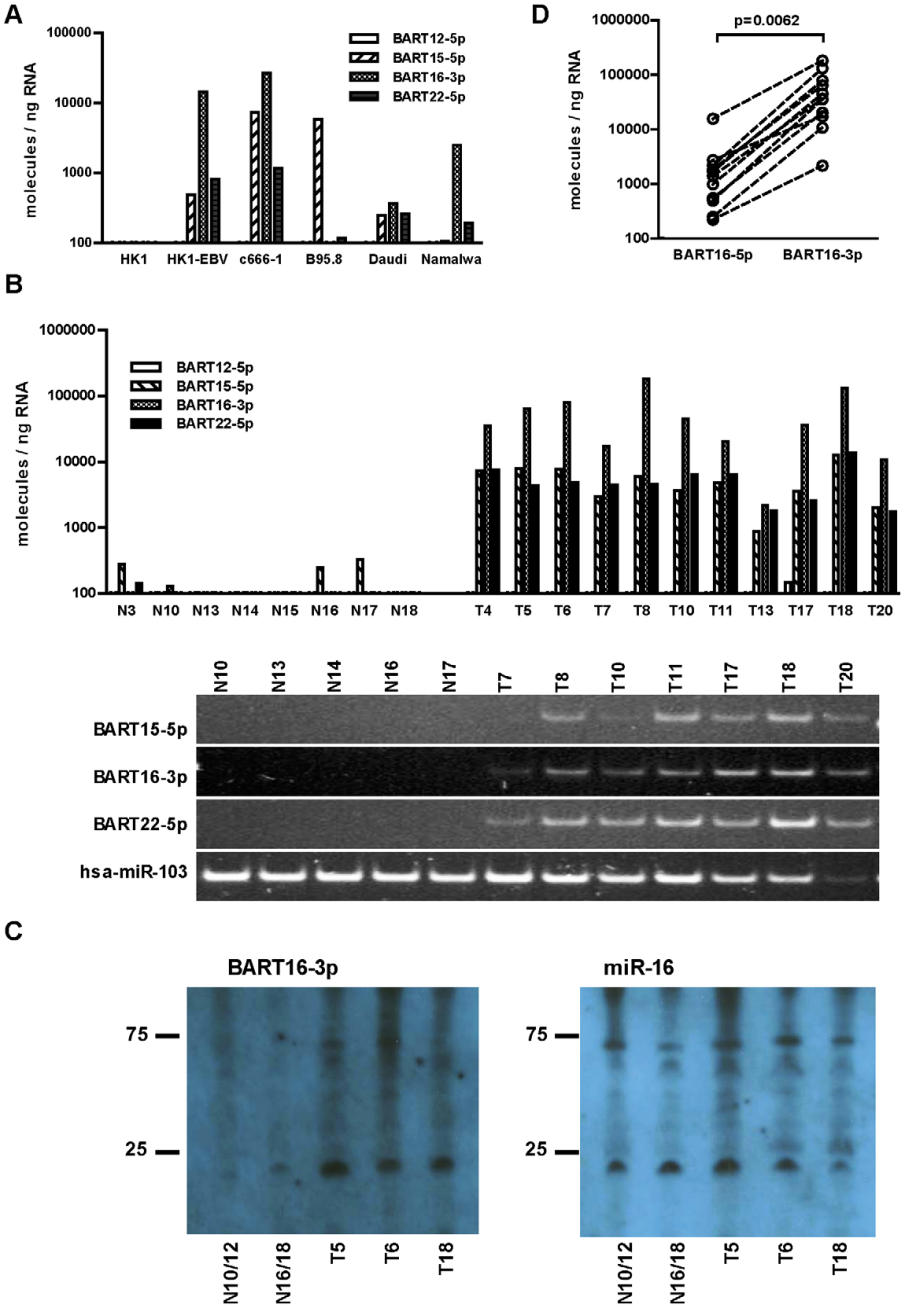
Detection and quantification of novel EBV miRNAs in cell lines and NPC tissues. (A). Expression of novel EBV miRNAs in NPC and B lymphoma cells. Expression of BART12-5p, BART15-5p, BART16-3p and BART22-5p was quantified using real-time PCR in EBV-positive cells, including HK1-EBV and c666-1 NPC carcinoma cells and B95.8, Daudi and Namalwa B lymphoma cells. HK1 is an EBV-negative NPC carcinoma cell line. Expression levels of miRNA were expressed as molecules per ng total RNA. (B). Expression of novel EBV miRNAs in normal and NPC tissues. Total RNAs from 11 NPC tissues and 8 adjacent normal tissues were analyzed for novel EBV miRNA expression using real-time PCR method. Expression levels of miRNA were expressed as molecules per ng total RNA. PCR products were analyzed using 10% polyacrylamide gel. Human miRNA hsa-miR-103, a ubiquitously expressed miRNA, was used as a loading control. (C). Northern blot detection of BART16-3p in NPC and adjacent normal tissues. Total RNA (5 µg) isolated from NPC tissues and adjacent normal tissues was separated in 12.5% denaturing polyacrylamide gels and analyzed with DIG-labeled LNA probes specific for the indicated miRNAs. RNAs from sample N10 and N12, N16 and N18 were pooled and used as normal tissues. (D). Expression level of BART16-5p and BART16-3p in NPC tissues. Expression levels of BART16-5p and BART16-3p were quantified in 11 NPC tissues using real-time PCR. P-value was calculated with paired-t-test (two-tailed).

We identified 44 mature EBV miRNAs from 22 precursor hairpins located in the BART region. Sequence information for these BART miRNAs and their read counts in T10 and N10 are summarized in Table 2. For clarity, -3p and -5p will be used to identify mature BART miRNAs and to indicate their origin on the corresponding precursor hairpin. Four mature miRNAs, including BART12-5p, BART15-5p, BART16-3p, and BART22-5p, have not been reported before. Secondary structure analysis confirmed that these four novel miRNAs are located in the stem region of their precursor hairpins (Figure S2). To confirm the presence of these novel EBV miRNAs, we used a stem-loop real-time PCR method to validate their expression in EBV-positive cell lines and NPC tissues [18]. As expected, none of these miRNAs were detected in the EBV-negative HK1 cells. In contrast, expression of BART15-5p, BART16-3p and BART22-5p was readily detected in two EBV-positive NPC cell lines, HK1-EBV and c666-1, and two EBV-positive B-lymphoma cell lines, Daudi and Namalwa. The EBV-positive B-lymphoma cells, B95.8, expressed significant levels of BART15-5p, consistent with the deletion of the majority of the BART locus in B95.8 genome [23] (Figure 2A). The low level of BART22-5p detected in B95.8 was due to background from the qPCR primer, as the gel electrophoresis did not detect a corresponding product. Similarly, expression of these three novel miRNAs was also readily detected in ten additional EBV-positive NPC tissues but was very low in abundance or completely absent in seven normal nasopharynx tissues (Figure 2B). Consistent with the read counts from the deep sequencing data for T10, the real-time PCR data indicated that expression levels of BART16-3p were higher than BART15-5p and BART22-5p in all NPC tissues. On the other hand, although BART12-5p was detected in the SOLiD sequence, the real-time PCR method could not detect its presence in majority of EBV-positive cell lines and NPC tissues. Northern blot analysis further confirmed the presence of BART16-3p in NPC tissues (Figure 2C). We were unable to detect BART12-5p, BART15-5p and BART22-5p in NPC samples with northern blotting due to their low abundance.

**Table 2:** EBV miRNAs detected by SOLiD sequencing in the T10 and the N10 samples.

EBV miRNA	miRBase Name	miRBase ^a^	Sequence ^b^	Start	End	Offset ^c^	T10 ^d^	T10 ^e^	N10 ^d^
BART3-5p	BART3*	12	AACCTAGTGTTAGTGTTGTGC	139086	139106	−1, −1	43	245	3
BART3-3p	BART3	12	CGCACCACTAGTCACCAGGTGT	139124	139145	0,0	6064	17990	120
BART4-5p	BART4	12	GACCTGATGCTGCTGGTGTGCT	139228	139249	0,0	1622	9204	33
BART4-3p	BART4*	13	CACATCACGTAGGCACCAGGTGT	139266	139288	0,0	66	128	0
BART1-5p	BART1-5p	12	TCTTAGTGGAAGTGACGTGCTGTG	139351	139374	0,0	18	462	8
BART1-3p	BART1-3p	12	TAGCACCGCTATCCACTATGTC	139387	139408	0,0	674	5551	3
BART15-5p		novel	AGGGAAACATGACCACCTGAAGTC	139519	139542	0,0	3	3	0
BART15-3p	BART15	12	GTCAGTGGTTTTGTTTCCTTGA	139553	139574	0,0	1212	1894	50
BART5-5p	BART5	12	CAAGGTGAATATAGCTGCCCATCG	139675	139698	0,0	2601	15654	64
BART5-3p	BART5*	13	GTGGGCCGCTGTTCACCTAA	139717	139736	0,2	110	300	0
BART16-5p	BART16	12	TTAGATAGAGTGGGTGTGTGCTC	139795	139817	0,−1	9	121	0
BART16-3p		novel	ATCACCACCCTCTATCCATAT	139836	139856	0,0	199	705	0
BART17-5p	BART17-5p	12	TAAGAGGACGCAGGCATACAA	139915	139935	0,−1	38	194	0
BART17-3p	BART17-3p	12	TGTATGCCTGGTGTCCCCTTAGT	139953	139975	0,0	664	1568	9
BART6-5p	BART6-5p	12	TAAGGTTGGTCCAATCCATAGG	140033	140054	0,0	15	1482	0
BART6-3p	BART6-3p	12	CGGGGATCGGACTAGCCTTAGA	140072	140093	0,0	430	1865	9
BART21-5p	BART21-5p	13	TCACTAGTGAAGGCAACTAAC	145514	145534	0,0	36	159	0
BART21-3p	BART21-3p	13	CTAGTTGTGCCCACTGGTGTTT	145548	145569	0,0	9	16	0
BART18-5p	BART18-5p	12	TCAAGTTCGCACTTCCTATACA	145962	145983	0,0	290	1853	4
BART18-3p	BART18-3p	12	TATCGGAAGTTTGGGCTTCGTC	145998	146019	0,0	8	44	0
BART7-5p	BART7*	12	CCTGGACCTTGACTATGAAACA	146439	146460	0,0	112	276	0
BART7-3p	BART7	12	CATCATAGTCCAGTGTCCAGGG	146475	146496	0,0	197	8313	5
BART8-5p	BART8	12	TACGGTTTCCTAGATTGTACAG	146772	146793	0,0	1532	2498	49
BART8-3p	BART8*	12	GTCACAATCTATGGGGTCGTAGA	146807	146829	0,0	1499	4869	26
BART9-5p	BART9*	12	TACTGGACCCTGAATTGGAAAC	146959	146980	0,0	29	191	0
BART9-3p	BART9	12	TAACACTTCATGGGTCCCGTAGT	146997	147019	0,0	5266	16398	77
BART22-5p		novel	TGCTAGACCCTGGAGTTGAACC	147169	147190	0,0	5	10	1
BART22-3p	BART22	13	TTACAAAGTCATGGTCTAGTAGT	147203	147225	0,0	167	534	6
BART10-5p	BART10*	12	GCCACCTCTTTGGTTCTGTAC	147321	147341	0,−1	81	158	3
BART10-3p	BART10	12	TACATAACCATGGAGTTGGCTGT	147356	147378	0,0	171	3633	4
BART11-5p	BART11-5p	12	TCAGACAGTTTGGTGCGCTAGTTG	147537	147560	0,0	30	350	1
BART11-3p	BART11-3p	12	ACGCACACCAGGCTGACTGCC	147575	147595	0,0	70	918	1
BART12-5p		novel	ACCCGCCCATCACCACCGGAC	147901	147921	0,0	19	82	0
BART12-3p	BART12	12	TCCTGTGGTGTTTGGTGTGGTT	147936	147957	0,0	16	142	0
BART19-5p	BART19-5p	12	ACATTCCCCGCAAACATGACATG	148215	148237	0,0	13	7713	0
BART19-3p	BART19-3p	12	TTTTGTTTGCTTGGGAATGCT	148254	148274	0,0	3	36	0
BART20-5p	BART20-5p	12	TAGCAGGCATGTCTTCATTCC	148339	148359	0,0	3	6	0
BART20-3p	BART20-3p	12	CATGAAGGCACAGCCTGTTACC	148374	148395	0,0	99	166	4
BART13-5p	BART13*	12	AACCGGCTCGTGGCTCGTACAG	148526	148547	0,0	35	333	1
BART13-3p	BART13	12	TGTAACTTGCCAGGGACGGCTGA	148563	148585	0,0	32	334	1
BART14-5p	BART14*	12	TACCCTACGCTGCCGATTTACA	148744	148765	0,0	27	360	1
BART14-3p	BART14	12	TAAATGCTGCAGTAGTAGGGAT	148778	148799	0,0	13	93	0
BART2-5p	BART2-5p	12	TATTTTCTGCATTCGCCCTTGC	152747	152768	0,0	605	1470	6
BART2-3p	BART2-3p	12	AAGGAGCGATTTGGAGAAAATAA	152783	152805	0,−1	8	12	0

a : miRBase version or novel miRNA.

b : 5′ to 3′.

c : 5′ and 3′ terminal position deviated from miRBase RefSeq.

d : Number of times the exactly matched sequence detected.

e : Number of times total sequence detected.

Interestingly, in the T10 sample, read counts of the newly identified BART16-3p were higher than for BART16-5p, the mature miRNA derived from the 5p stem of pre-miR-BART16 currently registered in miRBase [7]. To confirm the relative abundance of these two mature miRNAs, we quantified their absolute expression levels in 11 NPC tissues using real-time PCR. As shown in Figure 2D, the absolute expression level of BART16-3p is higher than that of BART16-5p in all NPC samples tested. The mean expression level of BART16-3p was approximately 20-fold higher than that of BART16-5p, similar to the read count ratio detected in T10 (Table 2). These data suggest that in NPC samples, BART16-3p is the predominant form of mature miRNA derived from the BART16 precursor hairpin.

### Length and sequence variations of EBV-encoded miRNAs

The EBV miRNA sequences obtained from deep sequencing exhibited a high degree of deviation from their miRBase “reference” sequences. Of the ∼113,000 EBV miRNA reads, only 22% of reads could be exactly aligned to the mature EBV miRNA sequence in the miRBase. Another 21% of the reads had sequence matches to the reference EBV miRNA but exhibited terminal heterogeneity at the 3′ and/or 5′ ends. The remaining 57% of reads exhibited one- to three-nucleotide variations from the reference EBV miRNA sequences in miRBase. The presence of miRNA variants, including length heterogeneity and nucleotide variations, referred to as isomiRs, has been described in previous studies using different deep sequencing technologies. The large abundance of length and sequence variants of EBV miRNA indicate that some of these isomiRs should be considered as the reference miRNA, at least in NPC tissue. We found 20 EBV miRNAs whose miRBase reference sequences represented the most abundant form of EBV miRNAs detected in the NPC tissue. In contrast, the reference sequences for six miRNAs, including BART2-3p, BART3-5p, BART5-3p and -5p, BART10-5p and BART16-5p, were not even detected. The extent of length heterogeneity and nucleotide variation observed in our EBV miRNA deep sequencing study was similar to that observed in miRNAs from Rhesus lymphocryptovirus (rLCV) [24] and Kaposi's sarcoma-associated herpesvirus (KSHV) [25], as well as in human and mouse miRNAs [17], [26], [27].

**Figure 3:**
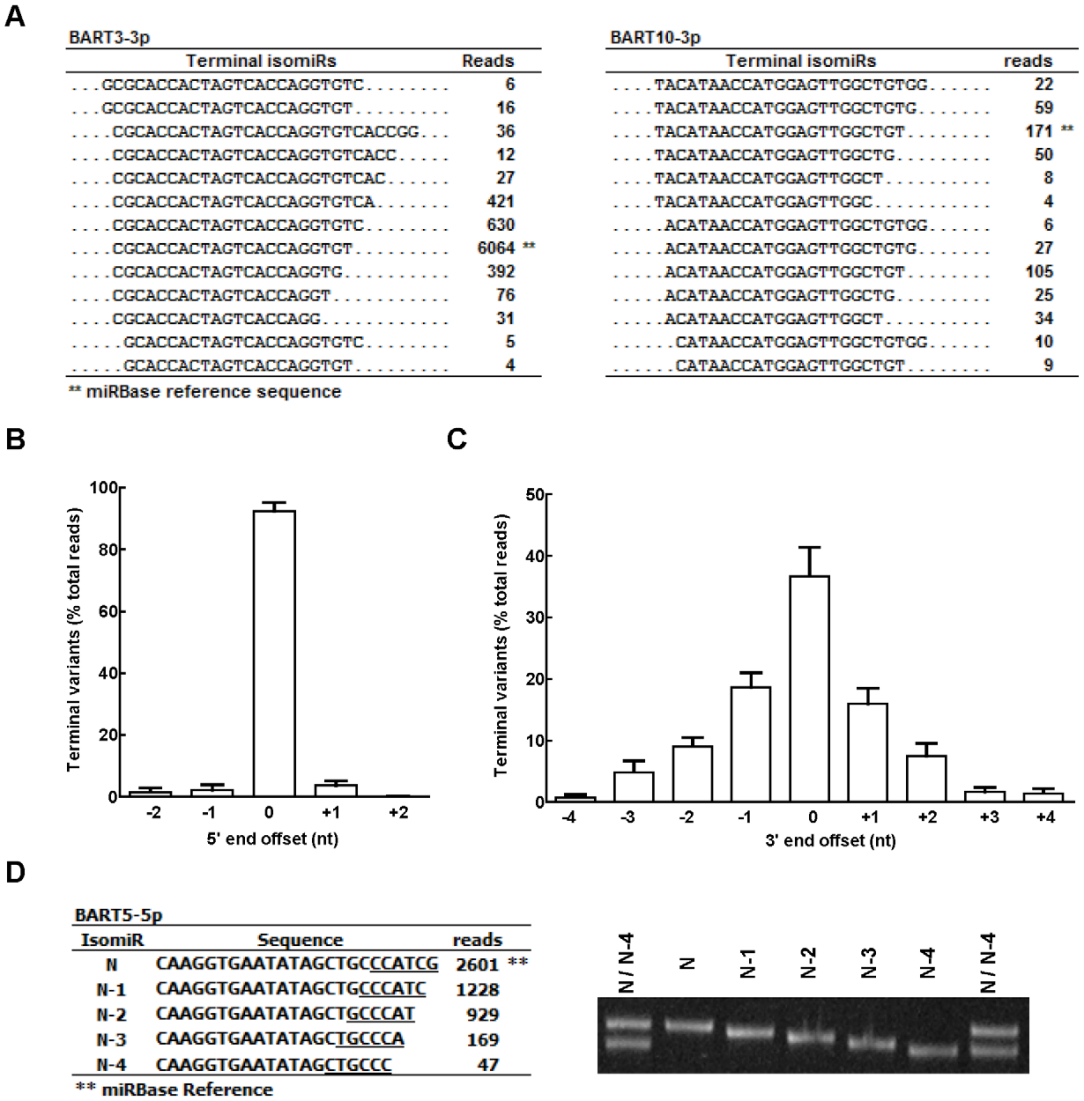
Length isomiRs of EBV BART miRNAs in T10. (A). Examples of length isomiRs of EBV miRNA BART3-3p and BART10-3p. Shown are the isomiR sequences and read counts detected in the T10 sample. (B). Distribution of 5′-end heterogeneity of EBV BART miRNA in T10. To analyze the 5′-end heterogeneity, each miRNA isomiR was aligned to its corresponding miRBase reference sequence to calculate the 5′-end nucleotide offset. IsomiRs with 5′-end nucleotide extension relative to the miRBase reference were assigned a negative offset number, while isomiRs with nucleotide deletion at the 5′-end were assigned a positive offset number. The frequency of 5′ terminal heterogeneity for each miRNA was calculated by dividing the read count at each alternative end by the total read count and was expressed as terminal variation frequency. Shown are the mean frequency ± SD calculated using 35 EBV BART miRNAs whose total reads exceed 100 in the T10 sample. (C). Distribution of 3′-end heterogeneity of EBV BART miRNAs in T10. A similar strategy was used to calculate the 3′-end nucleotide offset for each miRNA isomiR. The frequency of 3′ terminal heterogeneity for each miRNA was calculated by dividing the read count at each alternative end by the total read count and was expressed as terminal variation frequency. Shown are mean frequency ± SD calculated using 35 EBV BART miRNAs whose total reads exceed 100 in T10 sample. (D). Validation of BART5-5p 3′-end isomiRs in T10 sample. Sequence and read counts of the representative 3′ terminal isomiRs of BART5-5p are shown in the left panel. Nucleotides used to design stem-loop RT primer for each terminal isomiR (N to N-4) are underlined. PCR products of individual isomiR from T10 sample were analyzed using 15% polyacrylamide gel electrophoresis. To highlight the size difference of individual isomiRs, PCR products generated from N and N-4 primer were mixed and run on both sides to serve as size markers.

Figure 3A shows the sequences and read counts for BART3-3p and BART10-3p with alternative 3′ and 5′ ends (length isomiRs) detected in the T10 sample. The complete set of length isomiRs for all EBV miRNAs is shown in Table S2. Length variations of EBV miRNA were found at both the 5′- and 3′-ends. The frequency of 5′ end length variations for all EBV miRNAs was less than 5% and typically limited to plus or minus two nucleotides (Figure 3B). In contrast, the average frequency of 3′-end length variants for all EBV miRNAs was greater than 50% and spanned from minus 4 to plus 4 nucleotides (Figure 3C). To confirm the presence of length isomiRs for EBV miRNA in NPC, we designed a series of stem-loop RT primers, each of which recognized the last 6 nucleotides at the 3′-end of a specific length isomiR, and used RT-PCR to amplify individual length isomiRs. The PCR products were then separated on polyacrylamide gels to resolve the length heterogeneity. Synthetic miRNAs with 3′-end length heterogeneity were used to validate the sensitivity and specificity of these RT primers (Figure S3). The results shown in Figure 3D demonstrated the presence of various 3′-end length isomiRs of BART5-5p in the T10 sample.

**Figure 4:**
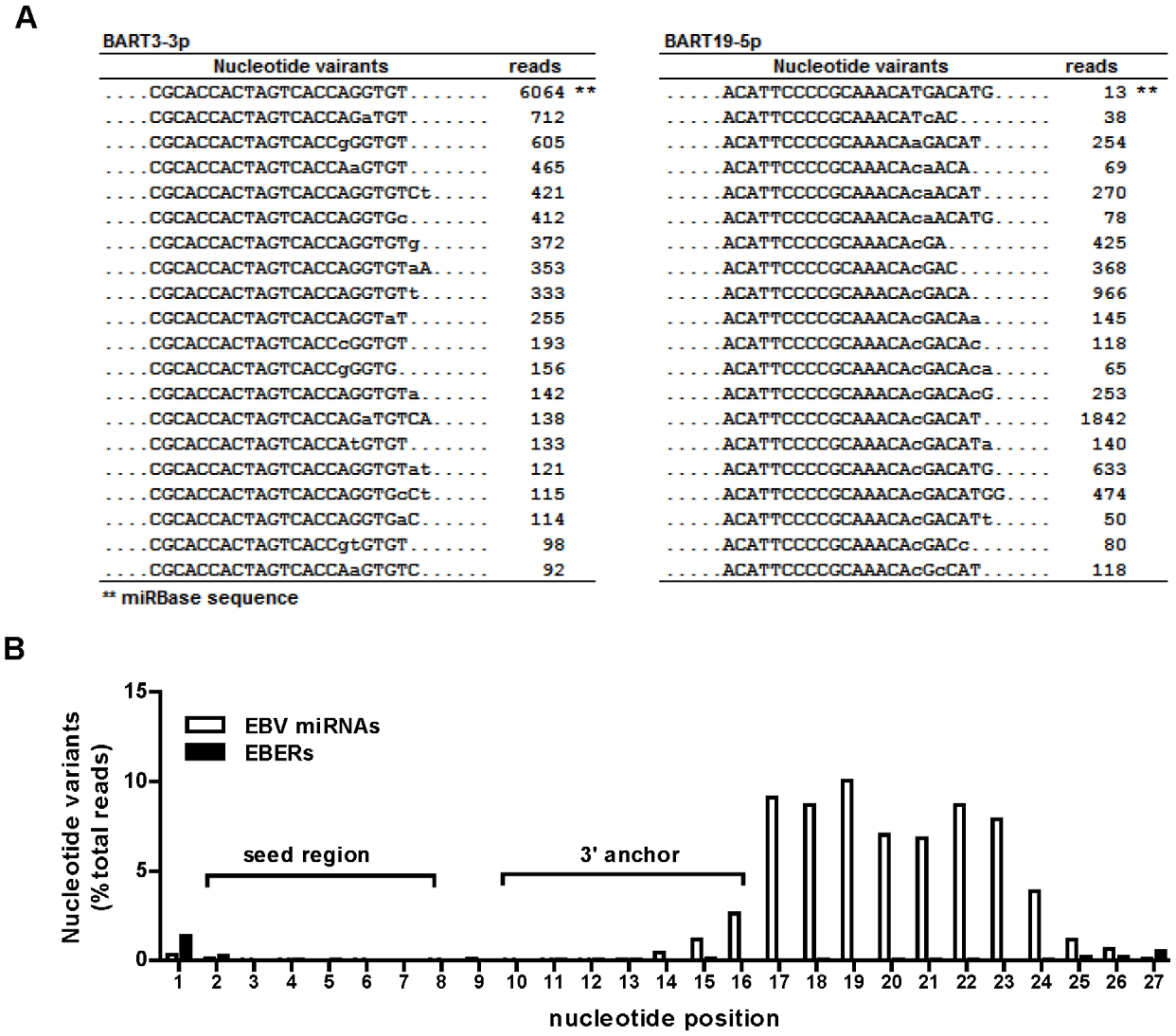
Nucleotide variations of EBV BART miRNAs. (A) Examples of nucleotide variants of BART3-3p and BART19-5p. Shown are the partial list of sequences and read counts of nucleotide variants detected in the T10 sample. Nucleotides deviated from the miRBase reference sequence are shown in lower case letters. (B) Distribution of nucleotide variation along the nucleotide positions of EBV miRNA. For individual EBV miRNAs, all sequence variants were aligned to the miRBase reference sequence to determine the nucleotide variation at each position. The variation frequency for each miRNA at each position was calculated by dividing the mismatched reads at each position by total reads. The average variation frequency of EBV miRNA at each position was calculated from 35 EBV BART miRNAs whose total reads exceed 100 in the T10 sample. A similar calculation was performed to determine the nucleotide variation frequency at each position of two EBV non-coding RNAs, EBER1 and EBER2. The frequency of nucleotide variation of EBERs and EBV miRNAs at each position was plotted on the same scale to highlight the pattern and the extent of nucleotide variation of EBV miRNAs. The nucleotide variation of EBV miRNA was clearly suppressed from nucleotides 2 to 14, covering the seed sequence and the potential miRNA:mRNA binding regions.

In addition to the length variations, more than 50% of the EBV miRNA deep sequencing reads exhibited one- to three-nucleotide mismatches to the reference sequence of EBV miRNA. Figure 4A shows a partial list of nucleotide variants of BART19-3p and BART3-3p. Similar to the length variation, the majority of nucleotide variation occurred in the 3′-end region, with significant variations observed in nt 17–23 of mature miRNAs. The average frequency of nucleotide variation for all EBV miRNA is shown in Figure 4B. Two lines of evidence suggest that the nucleotide variation observed in our current study is not an artifact of either the sequencing technology or the data-processing algorithm of the SOLiD platform. First, the nucleotide variations display a non-random pattern, with extremely low variation frequency within the miRNA seed region (nt 2–7) and low variation frequency in the miRNA cleavage/anchor region (nt 10–16). Secondly, the average variant frequency observed for the degradation products of EBERs detected from the same sequencing run is less than 1% across the entire 34-nt read length. Furthermore, the degree of nucleotide variation of EBV miRNAs in NPC detected with SOLiD is similar to the rLCV miRNAs sequencing data obtained by Riley et al. using the Illumina Solexa platform [24]. The complete set of nucleotide isomiRs for all EBV miRNAs is shown in Table S3. Table S4 summaries the sequences and read counts for major EBV miRNAs detected in the T10 sample.

**Figure 5:**
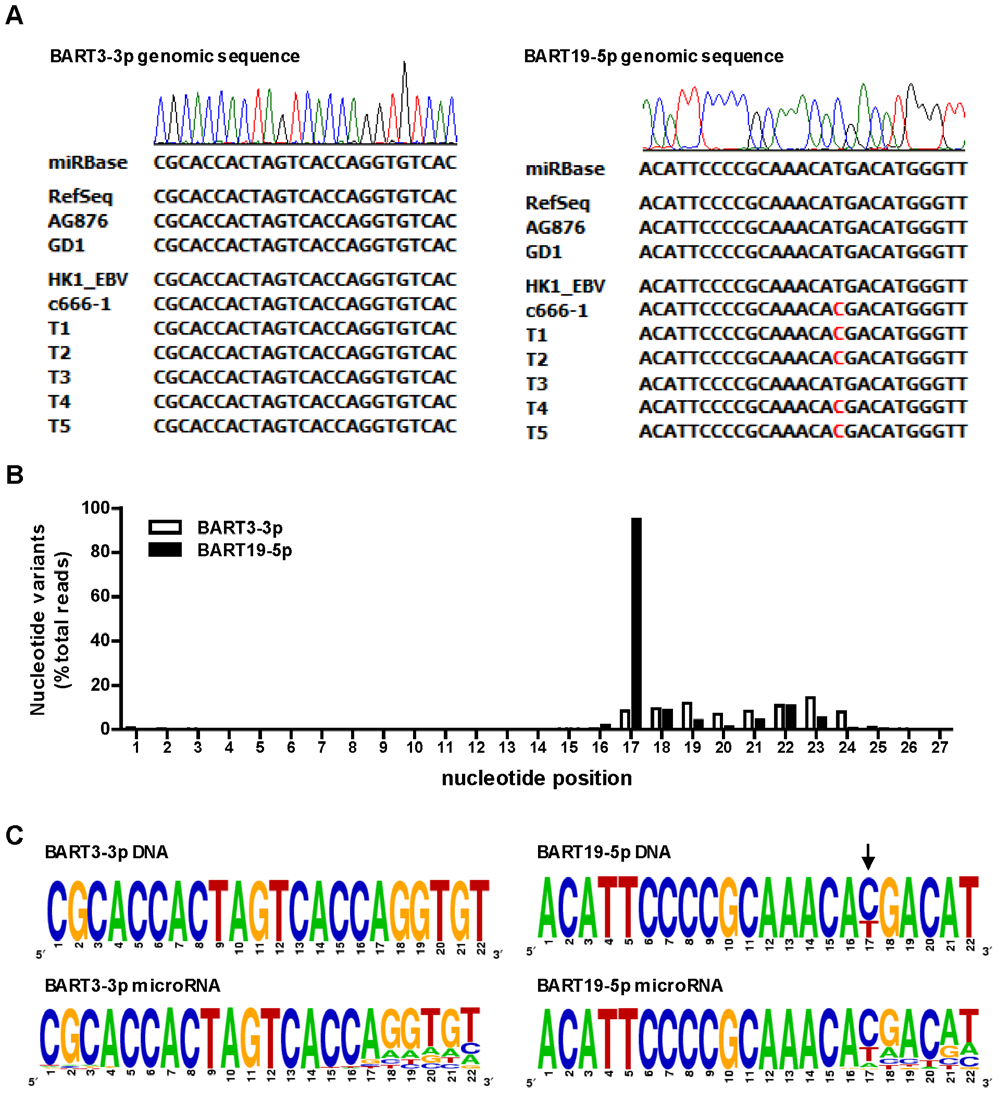
Nucleotide variations of EBV genomic DNA and BART miRNAs in NPC samples. (A) Example of genomic sequence of BART3-3p and BART19-5p in NPC cell lines and tissues. Genomic DNA from HK1-EBV, c666-1 cells and five NPC tissues was sequenced and compared to three EBV genomic sequences deposited in the GenBank, including the reference sequence of the EBV genome (RefSeq), AG876 and GD1. No nucleotide mismatch was found for BART3-3p in all NPC samples. A T to C nucleotide polymorphism at nt 17 of BART19-5p was found in c666-1 and four of the five NPC tissues. (B) Frequency of nucleotide variation in BART3-3p and BART19-5p miRNA detected in T10. For BART3-3p and BART19-5p, the frequency of nucleotide variation at each position was calculated by dividing the mismatched reads at each position by total reads. A greater than 90% variation frequency was detected at nt 17 of BART19-5p, corresponding to the T/C polymorphism found in the genomic sequence. (C) Sequence logos of BART3-3p and BART19-5p genomic DNA and miRNAs. DNA sequence logos were generated using all of the genomic sequences listed in Figure 5A, including three EBV genome sequences from GenBank and seven sequences from NPC samples. MiRNA sequence logos were generated using sequences of all unique isomiRs for BART3-3p and BART19-5p. Sequence logos represent the frequency of nucleotides detected at each position.

To clarify the source of nucleotide variations observed in EBV miRNAs, we sequenced the EBV genomic region corresponding to all BART miRNAs in five different NPC tissues and two NPC cell lines, HK1-EBV and c666-1. Given the heterogeneous nature of the tumor tissue sample, it was possible that the sequence variants detected in EBV microRNAs arose from genomic mutations in the tumor cell population. For each tumor sample, multiple clones were sequenced to address the potential heterogeneity of the EBV genome in tumor tissues. We did not find any sequence polymorphism in the genomic region of mature EBV miRNAs except for BART19-5p (Figure 5A). In four of the five NPC tissues and in c666-1 cells, we detected a T to C nucleotide change at the position of 148,231 of the EBV reference genome, corresponding to nt 17 of the mature BART19-5p. This polymorphism was reflected in the deep sequencing data with a >90% variation frequency in nt 17 of BART19-5p reads (Figure 5B). To further compare the sequence identity between the genomic and miRNA levels, we converted the aligned genome sequence as well as the miRNA sequences of BART3-3p and BART19-5p into the sequence logo [28]. As shown in Figure 5C, significant nucleotide variation occurred in the last six nucleotides of the 3′end of both mature EBV miRNAs, while no such nucleotide variation was found in the EBV genomic DNA. These data indicate that the majority of the nucleotide variants of EBV miRNAs detected in deep sequencing were the results of post-transcriptional modifications.

### Expression Levels of EBV miRNAs in NPC tissues

**Figure 6:**
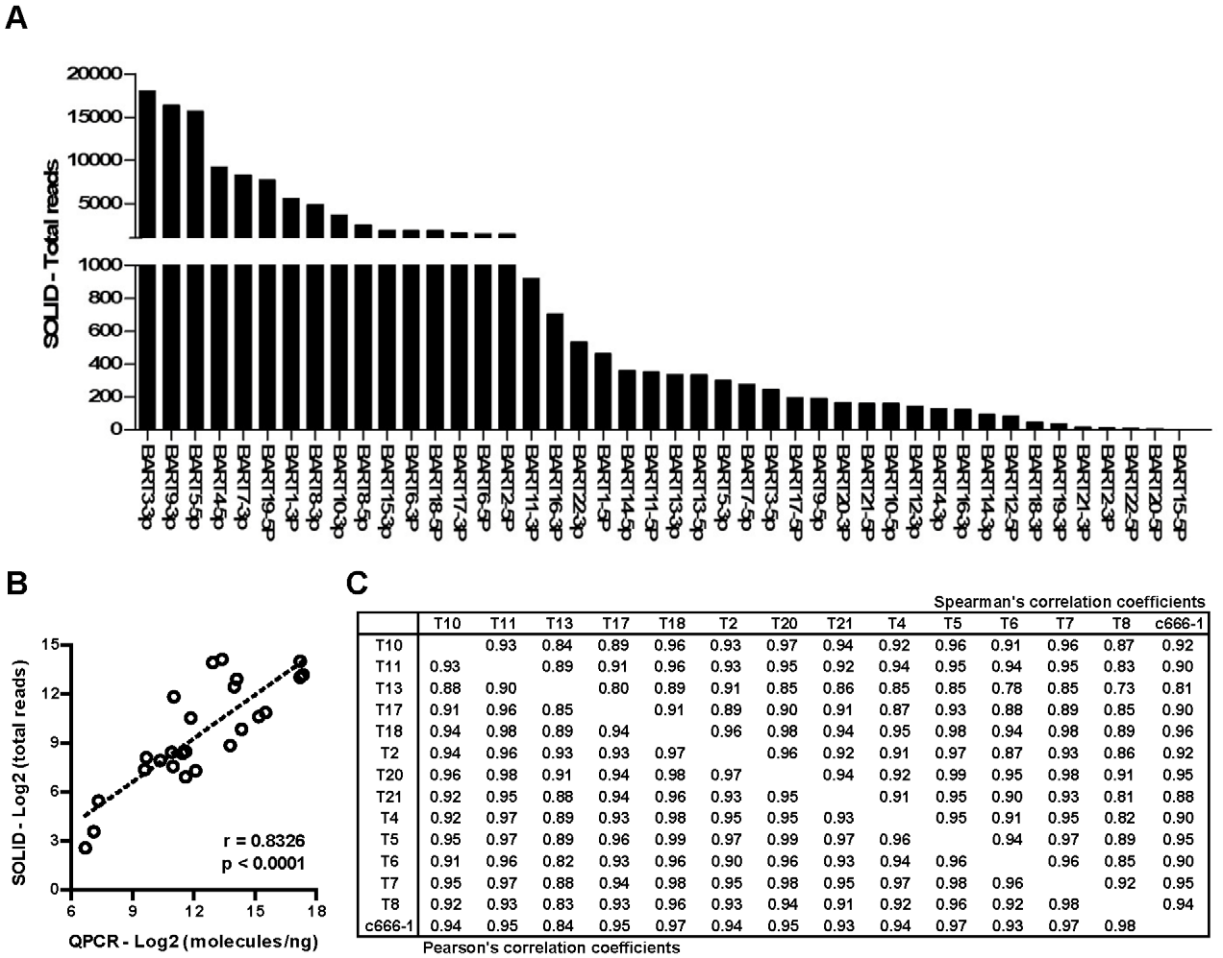
Expression levels and seed sequence conservation of EBV BART miRNAs in NPC samples. (A) Read counts of all EBV miRNAs detected in T10. Read counts for each miRNA include all terminal isomiRs and nucleotide variants. (B) Correlation between SOLiD read counts and real-time PCR expression levels of EBV miRNAs detected in T10. Twenty-five BART miRNAs in the T10 sample were quantified using real-time PCR. Expression levels of miRNA are expressed as molecules per ng total RNA. To calculate the correlation, total reads from SOLiD sequence and expression levels from real-time PCR were log2-transformed and analyzed using Person's correlation analysis. Pearson's correlation coefficient and p-value are shown in the inserts. (C) Conservation of BART miRNA expression pattern in NPC tissues and cell lines. Expression levels of 25 BART miRNAs in 13 NPC tissues and c666-1 cells were quantified using real-time PCR. Correlation analysis was performed on log2-transformed data to determine the similarity of BART miRNA expression patterns among these samples. Data shown in the lower left portion are Pearson's correlation coefficients. Values shown in the upper right portion are Spearman's correlation coefficients. Both correlation analyses show a high degree of similarity in BART miRNA expression patterns among these NPC samples and c666-1 cells.

As our sequence analysis shows that EBV miRNAs exist with multiple length and nucleotide variants, we decided to use the total read, including all length and nucleotide variants, to represent the abundance of individual EBV miRNAs in NPC tissue. As shown in Figure 6A, the read counts of individual EBV miRNAs in the T10 sample spanned from a few reads to approximately 18,000 reads. Notably, read counts of the three most abundant BART miRNAs, including BART3-3p, BART5-5p and BART9-3p, all exceeded the read count of miR-21(data not shown), a highly abundant human oncogenic miRNA. To validate the relative abundance of these EBV miRNAs derived from deep sequencing reads, we designed stem-loop real-time PCR primers for 25 EBV miRNAs, including high and low expressors, and quantified their expression levels in the T10 sample (Table S5). As shown in Figure 6B, the data from real-time PCR are highly correlated with the total reads from SOLiD sequencing (Pearson's correlation coefficient  = 0.83). Both technologies show that the expression levels of these EBV miRNAs span approximately 2,000-fold in the T10 sample. To ensure that the relative abundance of EBV miRNAs was not sample-dependent, we quantified the expression levels of these 25 BART miRNAs in 12 additional NPC tissues and c666-1 cells and analyzed the correlation of EBV miRNA expression pattern among these samples. Correlation coefficients among these NPC samples were all greater than 0.8 even though the absolute expression levels of EBV miRNAs varied substantially in individual samples (Figure 6C). These data indicate that the overall expression patterns of BART miRNAs were highly similar across all NPC tissues and cell lines.

### Highly abundant EBV miRNAs shared seed sequence with human miRNAs

**Figure 7:**
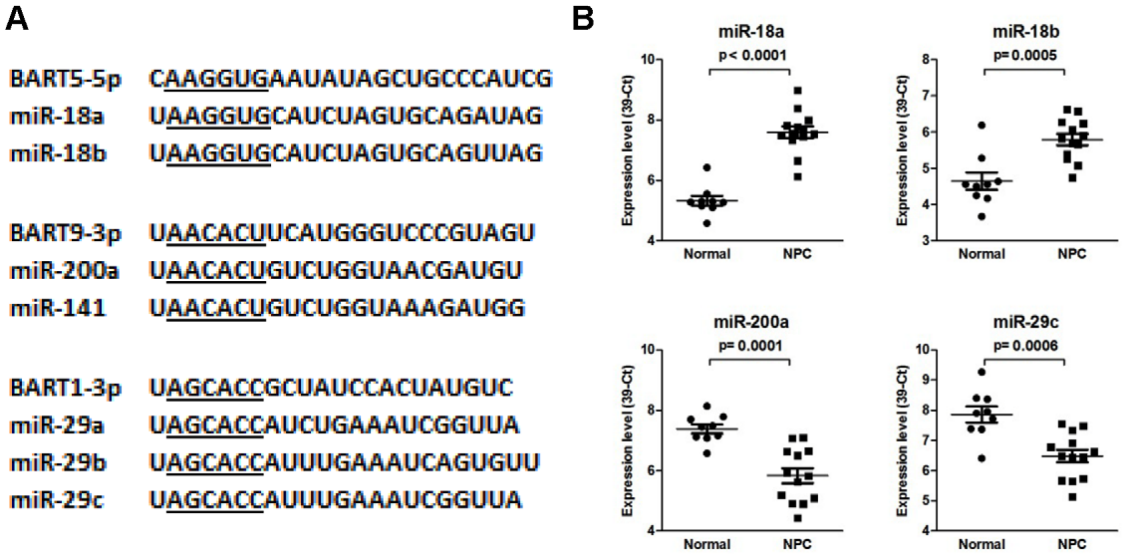
Expression levels of human miRNAs sharing seed sequences with highly abundant EBV BART miRNAs in NPC samples. (A) Sequence alignments between EBV miRNAs and human miRNAs. Seed sequences are underlined. (B) Expression levels of human miRNAs miR-18a, miR-18b, miR-200a and miR-29c in 9 normal and 13 NPC clinical tissues. The miRNA expression levels are presented as 39– Ct after normalization to u6 small RNA. P-values were calculated using t-test. Shown are the means ± sd.

The high expression levels of EBV miRNAs suggest that they may play important roles in regulating viral as well as cellular function. It is well recognized that the target spectrum of a miRNA is largely determined by its seed sequence. We hypothesized that the degree of seed-sequence homology between human and EBV miRNAs might reveal potential interaction between viral and human miRNAs. We therefore compared the seed sequences of all mature BART miRNAs to the seed sequences of all known human miRNAs (Lewis et al. 2005). There are 642 unique 6-mer seed (nt 2–7) sequences for all 988 human miRNAs registered in miRBase [29]. The expected seed-sharing probability between all EBV and all human miRNAs is 15.7%. We found that 13 of the 44 BART miRNAs shared identical seed sequences with 21 human miRNAs (Table 3). The observed seed-sharing percentage is 29.2%, about two-fold higher than the expected value. Interestingly, the seed-sharing frequency is not evenly distributed when the abundance of EBV miRNAs is taken into consideration. Six of the 10 most abundant EBV miRNAs shared their seed sequences with a total of 11 human miRNAs. In contrast, only one of the 10 least abundant EBV miRNA shared its seed sequence with 2 human miRNAs. The observed connection between seed-sequence sharing and EBV miRNA abundance suggest that seed-sequence content is an important factor in determining the differential accumulation of these EBV miRNAs. Moreover, the expression level of the miR-18 family (sharing seed with BART5-5p) was up-regulated in NPC, and expression levels of the miR-29 family (sharing seed with BART1-3p) and miR-200 family (sharing seed with BART9-3p) were down-regulated in NPC tissues (Figure 7) [19], [30]. These observations suggest that EBV miRNAs may influence critical cellular functions by either mimicking or competing with human miRNAs.

**Table 3:** EBV miRNAs sharing seed sequence with human miRNA.

EBV miRNA	Reads ^a^	Seed	Human miRNAs ^b^	Conservation ^c^	in NPC ^d^	Biological function and significance of human miRNAs
BART3-3p	17990	GCACCA	miR-767-5p	Mammals rLCV	nd ^e^	
BART9-3p	16398	AACACU	miR-141, -200a	Invertebrates rLCV	down	Inhibit EMT transition, cell growth, pluripotency & cancer progression Down-regulated in many solid tumors
BART5-5p	15645	AAGGUG	miR-18a, -18b	Vertebrates rLCV	up	Member of miR-17-92 cluster Oncogenic microRNA
BART7-3p	8313	AUCAUA	miR-154*, -487a	Mammals rLCV	nd ^e^	Expressed in human embryonic stem cells
BART1-3p	5551	AGCACC	miR-29a, -29b, -29c	Vertebrates rLCV	down	Suppress DNA methylation, apoptosis and cell migration Down-modulated in various type of cancer
BART8-3p	4869	UCACAA	miR-513b	primates	no change	Involved in IFN-r pathway
BART22-3p	534	UACAAAG ^f^	miR-520d-5p, -524	primates	nd ^e^	Expressed in undifferentiated human embryonic stem cells
BART11-5p	350	CAGACAG ^f^	miR-1324	Primates rLCV	nd ^e^	Cloned from neuroblastoma
BART7-5p	276	CUGGAC	miR-378, -422a	Mammals rLCV	no change	Promote cell survival, tumor growth and angiogenesis
BART9-5p	191	ACUGGA	miR-1243	rLCV	nd ^e^	
BART12-3p	142	CCUGUG	miR-1914	rLCV	nd ^e^	
BART4-3p	128	ACAUCAC ^f^	miR-499-3p	Vertebrates rLCV	nd ^e^	Heart-specific miRNA
BART2-3p	12	AGGAGC	miR-28-5p, -708	Mammals rLCV	no change	Dysregulated in bladder cancer
BHRF1-1 ^g^	0	AACCUG	miR-490-3p, -649	Vertebrates rLCV	nd ^e^	

a : Total read counts in the T10 sample.

b : Human miRNAs with identical seed sequence (2-7 nt).

c : Conservation scope of the seed sequence, data compiled from miRiewer.

d : Expression level of human miRNAs in NPC tissues.

e : Not determined.

f : Share 7-mer seed sequence (nt 2–8).

g : Highly abundant EBV miRNA in EBV-positive B lymphomas.

## Discussion

Previous studies have identified 25 miRNA precursor hairpins in the EBV genome, three located in the BHRF1 region and 22 in the BART region [6], [7], [8], [9]. To better understand the roles of these EBV-encoded miRNAs in the pathogenesis of NPC, we used the deep sequencing technology to interrogate the EBV miRNA transcriptome in clinical NPC tissues. Although the total reads for the BART miRNAs exceeded 110,000 in the T10 sample, no BHRF1 miRNA read was detected, consistent with the observation that BHRF1 is specifically expressed in EBV-positive B cells [9], [11]. We detected 44 mature EBV miRNAs derived from 22 distinct precursor hairpins in the BART region. Four of the mature EBV miRNAs have not been reported in previous studies using cloning or a deep sequencing method. These newly identified mature BART miRNAs, together with the 40 known BART miRNAs, established a complete BART miRNA transcriptome in NPC, with two mature miRNAs produced by each precursor hairpin. The EBV miRNA transcriptome in NPC is very similar to the viral miRNA transcriptome of the closely related rLCV [31]. One of the novel EBV miRNAs detected in the present study, BART15-5p, was previously predicted by Walz et al. based on the results from the rLCV study [31].

Our deep sequencing data revealed a wide diversity of length heterogeneity and sequence variations of EBV miRNAs in NPC tissue. These length and nucleotide variants constitute more than 50% of the total EBV miRNA reads, and many sequences show significant deviation from the miRBase reference sequence. Such length and sequence heterogeneity was not reported in a previous study that used the Roche 454 platform to sequence EBV miRNAs in two NPC tissues [9]. This discrepancy was mainly due to differences in the stringency of the data-processing algorithm. The data-processing algorithm used by Zhu et al. in the previous study did not allow any sequence deviation from the miRBase reference sequence [9]. However, similar length and sequence heterogeneity was reported in several recent studies using various technologies to sequence viral miRNAs [24], [25]. The miRNA sequences detected in these studies also show significant deviation from the reference sequence deposited in miRBase. Recently, de Hoon et al. discovered that cross-mapping may contribute to the sequence heterogeneity observed in data obtained from high-throughput sequencing libraries and proposed that a correcting algorithm might be useful to reduce potential error caused by cross-mapping [32]. However, the impact of cross-mapping on EBV miRNA sequencing data is probably minimal, as sequence similarity among EBV miRNAs is much lower than the sequence similarity observed in human miRNAs from the same miRNA family.

Length heterogeneity of EBV miRNAs was detected in both the 5′- and 3′-ends of mature miRNAs. However, the extent of length heterogeneity is significantly lower in the 5′-end, reflecting a better preservation of the 5′ sequence. The asymmetric length heterogeneity was observed in all EBV miRNAs, regardless of whether they were produced from the 3p arm or the 5p arm of the precursor hairpin. A similar phenomenon was reported in two recent studies using the Solexa platform to sequence viral miRNAs in latently KSHV-infected BC-3 B-cell line [25] and latently rLCV-infected monkey B cells [24]. Furthermore, the extent of asymmetric length heterogeneity observed in mature miRNAs encoded by KSHV, rLCV and EBV is very similar to that of human miRNAs from brain tissues [33]. Because length heterogeneity in the 5′-end will change the seed sequence and have a profound impact on the target spectrum, miRNAs are under selection pressure to maintain their seed sequences. Recent studies suggest that the conservation of miRNA 5′ termini was maintained by the selective loading of miRNA into the Argonaute complex [34].

Widespread sequence variants were detected for all 44 EBV BART miRNAs, with the counts of sequence variants for each miRNA roughly parallel to their total reads in the T10 sample. These sequence variants contain one- to three-nucleotide mismatches to the reference EBV genome, and the vast majority of nucleotide variations were detected at the 3′-end region, from nt 17 to nt 24. Extensive nucleotide variations in rLCV miRNAs have recently been reported by Riley et al. in latently rLCV-infected monkey B-cells using the Solexa sequencing platform [24]. Similar nucleotide variations have been reported on human brain miRNAs from normal samples, Huntington's disease brain regions [33] and neuroblastoma tissues [35], as well as on mouse let-7 miRNAs from ovarian tissues using both SOLiD and the Solexa sequencing platform [27]. The distribution pattern of nucleotide variations observed for EBV miRNAs in NPC is very similar to the pattern reported for human miRNAs from brain tissues, except that we did not find any increase in nucleotide variation in the bulge region (9–12 nt) [33]. This discrepancy could be due to the difference in the miRNA population studied.

Several mechanisms, including nucleotide polymorphism in the EBV genome and post-transcriptional modifications, could contribute to the sequence variations observed in EBV miRNAs. Indeed, one sequence polymorphism was detected in the BART region of the EBV genome. A T to C nucleotide polymorphism at nt 17 of BART19-5p was detected in four out of five clinical NPC samples and in the c666-1 NPC cells. While nucleotide polymorphism could account for the dramatic sequence variation observed in this particular site, the remaining sequence variations detected in EBV miRNAs probably resulted from post-transcriptional modifications. Recent studies have confirmed that RNA editing [36], [37] and non-template extension [26] are common post-transcriptional modifications, and these mechanisms could account for the wide diversity of sequence variations observed in mature EBV miRNA. Although the biological implications of these extensive modifications remain to be elucidated, adenylation at the 3′-end has been shown to enhance the stability of mature miRNAs [38].

Although all BART miRNAs were transcribed from the same BART transcripts [39], their read counts in the T10 sample spanned over 2000-fold, ranging from a few reads to close to 18,000 reads. The wide range of BART miRNA reads detected was confirmed using real-time PCR technology. This result is in agreement with two recent studies using TaqMan-based real-time PCR technology to quantify EBV miRNAs in EBV-positive cell lines [40] and NPC tissues [11]. A recent study by Pratt et al. [40] measuring the expression of 8 BART miRNAs in 17 EBV-positive cell lines also revealed that although the absolute levels of these miRNA differ among EBV-infected cells, the ranked order of the levels of individual BART miRNAs are similar among these cell lines. Therefore, the differential expression pattern of BART miRNAs seems to be independent of the cell types and the latency stages of EBV infection. The relative abundance of some EBV miRNAs detected in current study is different from the results of the study conducted by Cosmopoulos et al. [11]. This discrepancy is due to that two different methods were used to calculate the abundance for individual EBV miRNA. In the study conducted by Cosmopoulos et al., RT-PCR was used to quantify the expression level of only the miRBase RefSeq for individual EBV miRNA. However, in the present study, total read count, including all length and sequence isomiRs, was used to represent the abundance for each EBV miRNA.

Although several hypotheses have been postulated, the mechanisms governing the differential accumulation of individual BART miRNAs in EBV-infected cells remain unclear. Interestingly, we observed that six out of the ten most abundant EBV BART miRNAs share seed sequences with miRNAs that are evolutionally conserved from rLCV, to drosophila, to human. It is possible that these highly abundant EBV miRNAs may arise from a selection process driven by the virus to actively mimic human miRNAs in order to take control of critical biological functions of host cells [41], [42]; alternatively, the conserved seed sequences may play an active role in stabilizing these highly abundant BART miRNAs. This observation is consistent with a previous report that highly conserved human miRNAs are expressed at higher levels than non-conserved miRNAs [43]. In particular, the miR-200 family (shares seeds with BART9-3p), the miR-29 family (shares seeds with BART1-3p) and miR-18 (shares seeds with BART5-5p) are expressed at high levels in major types of human tissues and cell lines [44] and are highly conserved during evolution [43]. As miRNA concentration is a function of biogenesis and turnover, active miRNA degradation might also regulate miRNA accumulation. Recently, Chatterjee and Grosshans found that, in Caenorhabditis elegans, miRNAs that are not associated with targets were released from Argonaute and exposed to degradation by exonucleases [45]. These results suggest that target availability can stabilize the association of a miRNA with Argonaute and protect the miRNA from degradation. Previous studies have shown that conserved miRNAs typically were expressed more broadly and robustly than were nonconserved miRNAs [43], [46]. In addition, broadly expressed conserved miRNAs tend to have more predicted targets than conserved miRNAs with more restricted expression [46]. It is reasonable to assume that those EBV BART miRNAs that share seeds with highly conserved and broadly expressed human miRNAs would have more cellular targets and be better protected by the Argonaute complex, leading to their accumulation in the EBV-infected cells.

Read counts of the three most abundant EBV BART miRNAs, BART3-3p, BART5-5p and BART9-3p, all exceeded 15,000, similar to the level of a highly abundant human oncogenic miRNA, miR-21, in the T10 sample. BART5-5p has been shown to regulate a human pro-apoptotic target, p53 up-regulated modulator of apoptosis (PUMA), and promote the survival of NPC cells against etoposide-induced apoptosis [16]. The seed sequence of BART5-5p is identical to the seed sequence of two human miRNAs, hsa-miR-18a and miR-18b, which are members of the oncogenic miR-17-92 cluster. This oncogenic miRNA cluster is overexpressed in many types of human malignancy and promotes tumor formation when ectopically expressed in animals [47], [48]. Our previous study using real-time PCR also found that the expression levels of miR-18a and miR-18b were significantly up-modulated in NPC tissues [19].

The biological functions of BART9-3p have not been established. However, we found that the seed sequence of BART9-3p is identical to the seed sequence of the human miR-200 family, including miR-200a and miR-141. Recent studies clearly established the role of miR-200-family miRNAs in the control of epithelial-to-mesenchymal transition (EMT), a biological process involved in tumor metastasis [49], [50]. Down-regulation of the miR-200 family is observed in many solid tumors [51], [52], [53]. The expression level of miR-200a was found to be significantly reduced in NPC tissues [19]. In addition, another highly abundant EBV miRNA, BART1-3p, also shared its seed sequence with the miR-29 family, including miR-29a, -29b, and -29c. Expression levels of miR-29 family members were also significantly down-regulated in NPC tissues [19], [30], as well as in many other solid tumors. The human miR-29 family has been shown to regulate DNA methylation through DNMT3A and 3B [54] and to regulate cell survival through Mcl-1 and Tcl-1[55], [56]. Whether BART9-3p and BART1-3p also modulate the biological functions controlled by these human miRNAs remains to be elucidated. Nevertheless, these results clearly suggest a complex interplay between EBV and host miRNAs.

In the present study, we utilized the next generation sequencing technology to characterize the expression of EBV miRNAs in NPC tissue. The quantity of sequencing reads enabled us to identify several novel EBV miRNAs and uncover the extensive presence of terminal and nucleotide variants of EBV miRNAs in NPC tissue. Several highly abundant EBV miRNAs were detected at levels equivalent to the most abundant human miRNAs in NPC tissue. The extensive variants, coupled with their abundance, provide a powerful network for the virus to regulate its own biology as well as the host response.

## Supporting Information

Figure S1 Genomic localization of EBV small RNAs detected in CT10 sample. EBV small RNAs were aligned to the reference genome NC_007605. Locations of EBERs, BHRF1 and BART transcripts were highlighted. Shown are reads aligned to the sense strand of EBV genome. Segments of the EBV genome, expanding from 137,500 to 155,000, were shown in the bottom panel. Locations for BART cluster I, cluster II, and BART2 miRNAs were highlighted.
(0.20 MB PDF)

Figure S2 Secondary structure of EBV miRNAs. (A) Structure and sequences of novel EBV BART miRNAs detected in CT10. The stem-loop structure was based on the precursor hairpins predicted in miRBase. In all cases, the miRNA precursors give rise to two mature miRNAs. The mature miRNA sequences of newly discovered EBV miRNAs were indicated in red. The mature miRNA sequences in the opposite arm were shown in uppercase. (B) Comparison of miRBase standard sequence with the most abundant miRNA sequence detected in CT10. The stem- loop structure was based on the precursor hairpins predicted in miRBase. In all cases, the miRNA precursors give rise to two mature miRNAs. The mature miRNA sequences deposited in miRBase were shown in uppercase. The mature sequences of most abundant miRNAs detected in CT10 were indicated in red.
(0.31 MB PDF)

Figure S3 Validation of the specificity of RT primer for isomiRs. RNAs with sequence match to 3′-end isomiR of BART5-5p (N, N-1 and N-2 in Figure 3D) were synthesized and used as the template for isomiR detection. 1×109 copies of synthetic RNA were reverse transcribed using the indicated RT primer. Following the RT reaction, cDNA products were quantified using the universal reverse primer and the BART-5p specific forward primer. PCR products of individual reaction were analyzed using 15% polyacrylamide gel electrophoresis. Ct value for individual qPCR reaction was listed.
(0.18 MB PDF)

Table S1 Primers used for real-time PCR.
(0.02 MB PDF)

Table S2 Terminal isomiRs of EBV microRNAs in the T10 sample.
(0.07 MB PDF)

Table S3 Nucleotide variants of EBV microRNAs in the T10 sample.
(0.59 MB PDF)

Table S4 Major EBV miRNAs detected by SOLiD sequencing in T10 and N10 samples.
(0.04 MB PDF)

Table S5 Expression levels of EBV miRNAs detected by RT-PCR in 13 NPC tissues and c666-1 cells.
(0.02 MB PDF)
