# Supplementary figures and images for: Characterization of Epstein-Barr Virus miRNAome in Nasopharyngeal Carcinoma by Deep Sequencing
Source: PLoS One. 2010 Sep 20;5(9):e12745. doi: 10.1371/journal.pone.0012745 (PMC2942828; doi:10.1371/journal.pone.0012745)

Figure S1

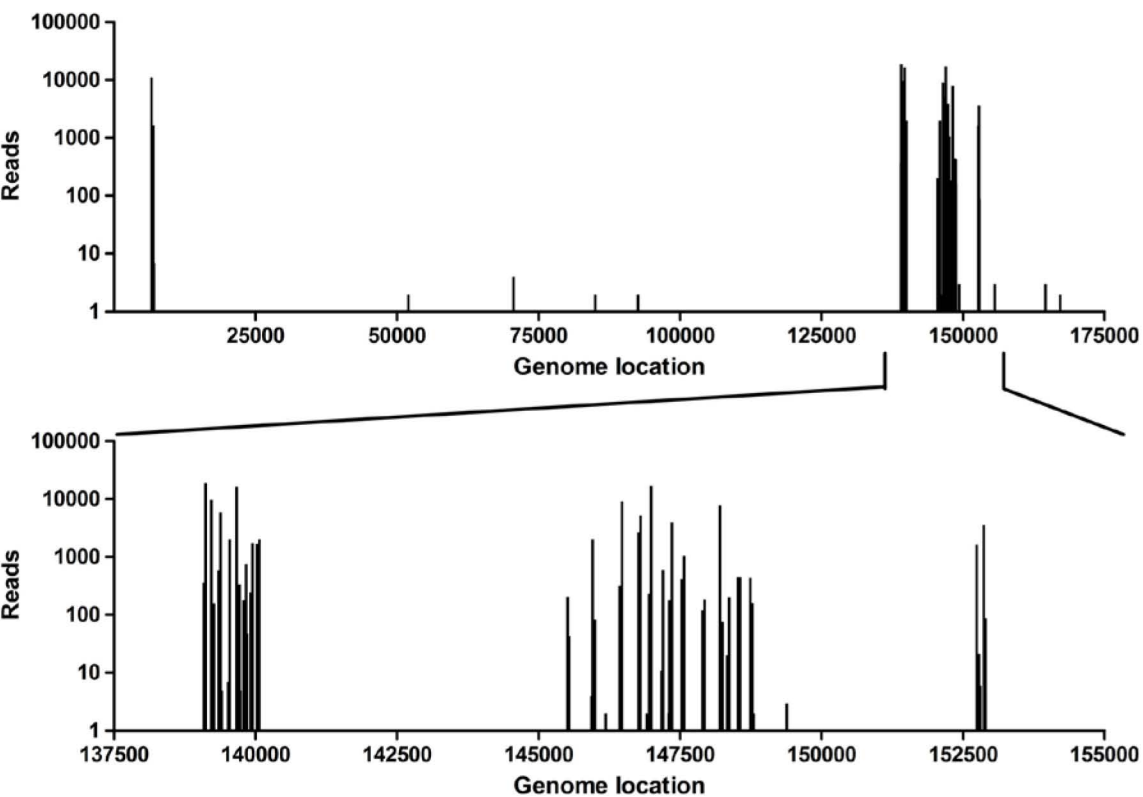

Supplement: Figure S1 — Genomic localization of EBV small RNAs detected in CT10 sample. EBV small RNAs were aligned to the reference genome NC_007605. Locations of EBERs, BHRF1 and BART transcripts were highlighted. Shown are reads aligned to the sense strand of EBV genome. Segments of the EBV genome, expanding from 137,500 to 155,000, were shown in the bottom panel. Locations for BART cluster I, cluster II, and BART2 miRNAs were highlighted. (0.20 MB PDF) [file pone.0012745.s001.pdf]

**Figure S3**

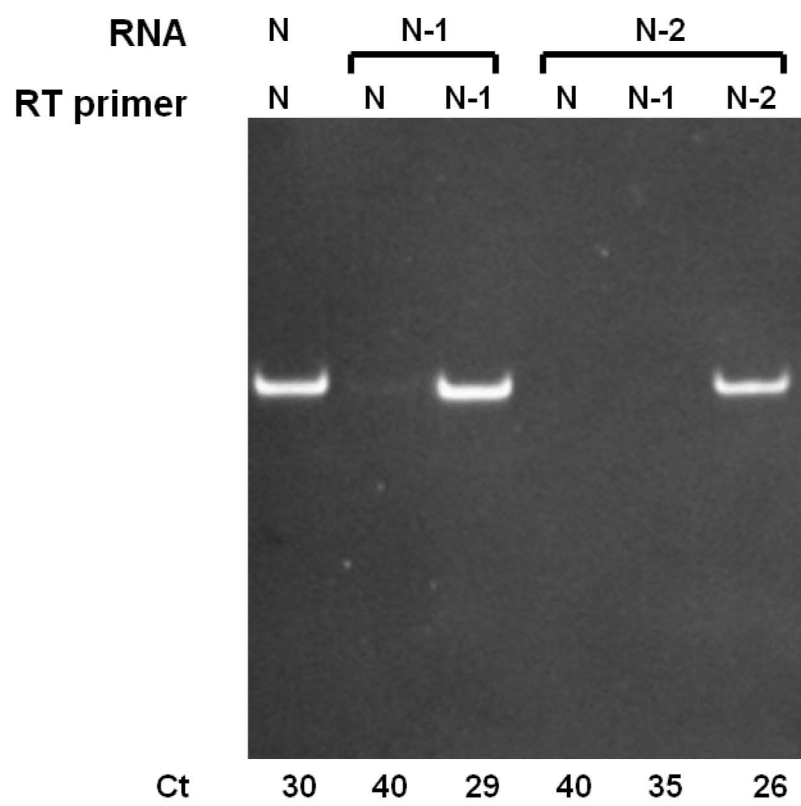

Supplement: Figure S3 — Validation of the specificity of RT primer for isomiRs. RNAs with sequence match to 3′-end isomiR of BART5-5p (N, N-1 and N-2 in Figure 3D) were synthesized and used as the template for isomiR detection. 1×10^9^ copies of synthetic RNA were reverse transcribed using the indicated RT primer. Following the RT reaction, cDNA products were quantified using the universal reverse primer and the BART-5p specific forward primer. PCR products of individual reaction were analyzed using 15% polyacrylamide gel electrophoresis. Ct value for individual qPCR reaction was listed. (0.18 MB PDF) [file pone.0012745.s003.pdf]
