# Supplementary material for: Characterization of Epstein-Barr Virus miRNAome in Nasopharyngeal Carcinoma by Deep Sequencing
Source: PLoS One. 2010 Sep 20;5(9):e12745. doi: 10.1371/journal.pone.0012745 (PMC2942828; doi:10.1371/journal.pone.0012745)
Supplement: Figure S2 — Secondary structure of EBV miRNAs. (A) Structure and sequences of novel EBV BART miRNAs detected in CT10. The stem-loop structure was based on the precursor hairpins predicted in miRBase. In all cases, the miRNA precursors give rise to two mature miRNAs. The mature miRNA sequences of newly discovered EBV miRNAs were indicated in red. The mature miRNA sequences in the opposite arm were shown in uppercase. (B) Comparison of miRBase standard sequence with the most abundant miRNA sequence detected in CT10. The stem- loop structure was based on the precursor hairpins predicted in miRBase. In all cases, the miRNA precursors give rise to two mature miRNAs. The mature miRNA sequences deposited in miRBase were shown in uppercase. The mature sequences of most abundant miRNAs detected in CT10 were indicated in red. (0.31 MB PDF) [file pone.0012745.s002.pdf]

Figure S2

A

```
>ebv-mir-BART12 (-38.50)    [ebv-miR-BART12:49-70]

   -   a     c   -    cau      c      auu
cug gug ccuaa acc cgcc   caccac ggacag   c
||| ||| ||||| ||| ||||   |||||| ||||||   u
gac cau ggguu UGG GUGG   GUGGUG CCUguu   g
   g   g     U   U    UUU      U      caa

>ebv-mir-BART15 (-29.10)    [ebv-miR-BART15:47-68]

u   c g  ugg         u      c   ag    u
 gug c cu   agggaaaca gaccac uga  ucug u
 ||| | ||   ||||||||| |||||| |||  |||| a
 cac g ga   UUCCUUUGU UUGGUG ACU  Ggac a
a   a a  uAG         U      -   --    c

>ebv-mir-BART16 (-34.80)    [ebv-miR-BART16:20-43]

   -cuu    g     a  UUA      GU     U   C     uuua
agg    ucag ugugg au   GAUAGA  GGGUG GUG UCUug    a
|||    |||| ||||| ||   ||||||  ||||| ||| |||||    u
ucc    aguu acacc ua   cuaucu  cccac cac agaac    u
   aaau    a     c  uac      --     -   u     caca

>ebv-mir-BART22 (-35.80)    [ebv-miR-BART22:43-65]

       g         c   ag   -    -  u
gucacag ugcuagacc ugg  uug aacc ag a
||||||| ||||||||| |||  ||| |||| || c
caguguU AUGAUCUGG ACU  AAC UUgg uc c
       G         U   GA   A    c  a
```

B

```
>ebv-mir-BART2 (-25.90)    [ebv-miR-BART2-5p:3-24] [ebv-miR-BART2-3p:39-62]

ac        G  -U    -       uguc
  UAUUUUCU CA  UCGC CCUUGCg    c
  |||||||| ||  |||| |||||||
  AUAAAAGA GU  AGCG GGAAcgu    a
AA        G  UU    A       uguu

>ebv-mir-BART3 (-40.70)    [ebv-miR-BART3*:12-32] [ebv-miR-BART3:49-70]

         ga    A   -       U       uaaau
ccuuuggug  ACCU GUG UUAGUG UGUGCUg      a
|||||||||  |||| ||| |||||| |||||||
ggaggccac  UGGA CAC GAUCAC ACGCgac      a
         UG    C   U       C       cugug

>ebv-mir-BART5 (-38.60)    [ebv-miR-BART5:15-38] [ebv-miR-BART5*:57-74]

  uc    g   cuCA      AU     U        ac  a  g
gc  ugug cac    AGGUGA  AUAGC GCCCAUCG  gu uc c
||  |||| |||    ||||||  ||||| ||||||||  || ||
ug  acgc gug    UCCACU  UGUCG CGGGUGgc  ca ag u
  ga    a   -aaa      --     C        --  a  g

>ebv-mir-BART10 (-49.90)    [ebv-miR-BART10*:18-39] [ebv-miR-BART10:53-75]

    -      uc   g     C    U     C      uauu
caga ggagug  ccg gGCCA CUCU UGGUU UGUACA    u
|||| ||||||  ||| ||||| |||| ||||| ||||||    u
gucu ccucac  ggU UCGGU GAGG ACCAA ACAUgu    g
    a      gu   G     U    U     U      uauu

>ebv-mir-BART16 (-34.80)    [ebv-miR-BART16:20-43]

   -cuu    g     a  UUA      GU     U   C     uuua
agg    ucag ugugg au   GAUAGA  GGGUG GUG UCUug    a
|||    |||| ||||| ||   ||||||  ||||| ||| |||||    u
ucc    aguu acacc ua   cuaucu  cccac cac agaac    u
   aaau    a     c  uac      --     -   u     caca

>ebv-mir-BART17 (-49.60)    [ebv-miR-BART17-5p:22-43] [ebv-miR-BART17-3p:60-82]

    aac   -a    gca  -     A      -            uuauu
guug   agg  ugug   cc cUAAG GGACGC AGGCAUACAAGg     a
||||   |||  ||||   || ||||| |||||| ||||||||||||
cgau   ucc  acgc   gg GAUUC CCUGUG UCCGUAUGUucc     c
    gga   gg    -ag  U     C      G            ugacc
```
