# Supplementary material for: Characterization of Epstein-Barr Virus miRNAome in Nasopharyngeal Carcinoma by Deep Sequencing
Source: PLoS One. 2010 Sep 20;5(9):e12745. doi: 10.1371/journal.pone.0012745 (PMC2942828; doi:10.1371/journal.pone.0012745)
Supplement: Table S1 — Primers used for real-time PCR. (0.02 MB PDF) [file pone.0012745.s004.pdf]

Table S1. Primers List

Gene name	Primer type	Sequence
ebv-mir-BART5 (N)	6-mer Stem-loop RT primer	CTCAACTGGTGTCGTGGAGTCGGCAATTCAGTTGAGCGATGG
ebv-mir-BART5 (N-1)	6-mer Stem-loop RT primer	CTCAACTGGTGTCGTGGAGTCGGCAATTCAGTTGAGGATGGG
ebv-mir-BART5 (N-2)	6-mer Stem-loop RT primer	CTCAACTGGTGTCGTGGAGTCGGCAATTCAGTTGAGATGGGC
ebv-mir-BART5 (N-3)	6-mer Stem-loop RT primer	CTCAACTGGTGTCGTGGAGTCGGCAATTCAGTTGAGTGGGCA
ebv-mir-BART5 (N-4)	6-mer Stem-loop RT primer	CTCAACTGGTGTCGTGGAGTCGGCAATTCAGTTGAGGGGCAG
ebv-miR-BART10	8-mer Stem-loop RT primer	CTCAACTGGTGTCGTGGAGTCGGCAATTCAGTTGAGACAGCCAA
ebv-miR-BART10*	8-mer Stem-loop RT primer	CTCAACTGGTGTCGTGGAGTCGGCAATTCAGTTGAGTGTACAGA
ebv-miR-BART11-3p	8-mer Stem-loop RT primer	CTCAACTGGTGTCGTGGAGTCGGCAATTCAGTTGAGGGCAGTCA
ebv-miR-BART11-5p	8-mer Stem-loop RT primer	CTCAACTGGTGTCGTGGAGTCGGCAATTCAGTTGAGCAACTAGC
ebv-mir-BART12-5P	8-mer Stem-loop RT primer	CTCAACTGGTGTCGTGGAGTCGGCAATTCAGTTGAGTGTCCGGT
ebv-miR-BART12-5p	8-mer Stem-loop RT primer	CTCAACTGGTGTCGTGGAGTCGGCAATTCAGTTGAGCTGTCCGG
ebv-miR-BART13	8-mer Stem-loop RT primer	CTCAACTGGTGTCGTGGAGTCGGCAATTCAGTTGAGTCAGCCGT
ebv-miR-BART1-3p	8-mer Stem-loop RT primer	CTCAACTGGTGTCGTGGAGTCGGCAATTCAGTTGAGGACATAGT
ebv-miR-BART14*	8-mer Stem-loop RT primer	CTCAACTGGTGTCGTGGAGTCGGCAATTCAGTTGAGTGTAAATC
ebv-mir-BART15-5P	8-mer Stem-loop RT primer	CTCAACTGGTGTCGTGGAGTCGGCAATTCAGTTGAGAGACTTCA
ebv-miR-BART1-5p	8-mer Stem-loop RT primer	CTCAACTGGTGTCGTGGAGTCGGCAATTCAGTTGAGCACAGCAC
ebv-miR-BART16	8-mer Stem-loop RT primer	CTCAACTGGTGTCGTGGAGTCGGCAATTCAGTTGAGAGAGCACA
ebv-mir-BART16-3P	8-mer Stem-loop RT primer	CTCAACTGGTGTCGTGGAGTCGGCAATTCAGTTGAGATATGGAT
ebv-mir-BART16-5P	8-mer Stem-loop RT primer	CTCAACTGGTGTCGTGGAGTCGGCAATTCAGTTGAGGCACACAC
ebv-miR-BART17-3p	8-mer Stem-loop RT primer	CTCAACTGGTGTCGTGGAGTCGGCAATTCAGTTGAGACTAAGGG
ebv-miR-BART18-3p	8-mer Stem-loop RT primer	CTCAACTGGTGTCGTGGAGTCGGCAATTCAGTTGAGGACGAAGC
ebv-miR-BART19-5p	8-mer Stem-loop RT primer	CTCAACTGGTGTCGTGGAGTCGGCAATTCAGTTGAGCATGTCAT
ebv-miR-BART20-3p	8-mer Stem-loop RT primer	CTCAACTGGTGTCGTGGAGTCGGCAATTCAGTTGAGGGTAACAG
ebv-miR-BART20-5p	8-mer Stem-loop RT primer	CTCAACTGGTGTCGTGGAGTCGGCAATTCAGTTGAGGGAATGAA
ebv-mir-BART21-3p	8-mer Stem-loop RT primer	CTCAACTGGTGTCGTGGAGTCGGCAATTCAGTTGAGAAACACCA
ebv-mir-BART21-5p	8-mer Stem-loop RT primer	CTCAACTGGTGTCGTGGAGTCGGCAATTCAGTTGAGGTTAGTTG
ebv-mir-BART-22	8-mer Stem-loop RT primer	CTCAACTGGTGTCGTGGAGTCGGCAATTCAGTTGAGACTACTAG
ebv-miR-BART22-5P	8-mer Stem-loop RT primer	CTCAACTGGTGTCGTGGAGTCGGCAATTCAGTTGAGGGTTCAAC
ebv-miR-BART2-3p	8-mer Stem-loop RT primer	CTCAACTGGTGTCGTGGAGTCGGCAATTCAGTTGAGTTTATTTT
ebv-miR-BART3	8-mer Stem-loop RT primer	CTCAACTGGTGTCGTGGAGTCGGCAATTCAGTTGAGACACCTGG
ebv-miR-BART3*	8-mer Stem-loop RT primer	CTCAACTGGTGTCGTGGAGTCGGCAATTCAGTTGAGAGCACAAC
ebv-miR-BART4	8-mer Stem-loop RT primer	CTCAACTGGTGTCGTGGAGTCGGCAATTCAGTTGAGAGCACACC
ebv-miR-BART4-3P	8-mer Stem-loop RT primer	CTCAACTGGTGTCGTGGAGTCGGCAATTCAGTTGAGACACCTGG
ebv-miR-BART5	8-mer Stem-loop RT primer	CTCAACTGGTGTCGTGGAGTCGGCAATTCAGTTGAGCGATGGGC
ebv-miR-BART5-3P	8-mer Stem-loop RT primer	CTCAACTGGTGTCGTGGAGTCGGCAATTCAGTTGAGAGGTGAAC
ebv-miR-BART6-3p	8-mer Stem-loop RT primer	CTCAACTGGTGTCGTGGAGTCGGCAATTCAGTTGAGTCTAAGGC
ebv-miR-BART6-5p	8-mer Stem-loop RT primer	CTCAACTGGTGTCGTGGAGTCGGCAATTCAGTTGAGCCTATGGA
ebv-miR-BART7	8-mer Stem-loop RT primer	CTCAACTGGTGTCGTGGAGTCGGCAATTCAGTTGAGCCCTGGAC
ebv-miR-BART7*	8-mer Stem-loop RT primer	CTCAACTGGTGTCGTGGAGTCGGCAATTCAGTTGAGTGTTTCAT
ebv-miR-BART9	8-mer Stem-loop RT primer	CTCAACTGGTGTCGTGGAGTCGGCAATTCAGTTGAGACTACGGG
ebv-miR-BART9*	8-mer Stem-loop RT primer	CTCAACTGGTGTCGTGGAGTCGGCAATTCAGTTGAGGTTTCCAA
ebv-miR-BART10	qPCR forward primer	CGGCGGTACATAACCATGGAGT
ebv-miR-BART10*	qPCR forward primer	CGGCGGGCCACCTCTTTGGTTC
ebv-miR-BART11-3p	qPCR forward primer	CGGCGGACGCACACCAGGCTGA
ebv-miR-BART11-5p	qPCR forward primer	CGGCGGTCAGACAGTTTGGTGC
ebv-mir-BART12-5P	qPCR forward primer	CGGCGGACCCGCCCATCACCAC
ebv-miR-BART13	qPCR forward primer	CGGCGGTGTAACTTGCCAGGGA
ebv-miR-BART1-3p	qPCR forward primer	CGGCGGTAGCACCGCTATCCAC
ebv-miR-BART14*	qPCR forward primer	CGGCGGTACCCTACGCTGCCGA
ebv-mir-BART15-5P	qPCR forward primer	CGGCGGAGGGAAACATGACCAC
ebv-miR-BART1-5p	qPCR forward primer	CGGCGGTCTTAGTGGAAGTGAC
ebv-miR-BART16	qPCR forward primer	CGGCGGTTAGATAGAGTGGGTG
ebv-mir-BART16-3P	qPCR forward primer	CGGCGGATCACCACCCTCTATC
ebv-miR-BART17-3p	qPCR forward primer	CGGCGGTGTATGCCTGGTGTCC
ebv-miR-BART18-3p	qPCR forward primer	CGGCGGTATCGGAAGTTTGGGC
ebv-miR-BART19-5p	qPCR forward primer	CGGCGGACATTCCCCGCAAACA
ebv-miR-BART20-3p	qPCR forward primer	CGGCGGCATGAAGGCACAGCCT
ebv-miR-BART20-5p	qPCR forward primer	CGGCGGTAGCAGGCATGTCTTC
ebv-mir-BART21-3p	qPCR forward primer	CGGCGGCTAGTTGTGCCCACTG
ebv-mir-BART21-5p	qPCR forward primer	CGGCGGTCACTAGTGAAGGCAA
ebv-mir-BART-22	qPCR forward primer	CGGCGGTTACAAAGTCATGGTC
ebv-miR-BART22-5P	qPCR forward primer	CGGCGGTGCTAGACCCTGGAGT
ebv-miR-BART2-3p	qPCR forward primer	CGGCGGAAGGAGCGATTTGGAG
ebv-miR-BART3	qPCR forward primer	CGGCGGCGCACCACTAGTCACC
ebv-miR-BART3*	qPCR forward primer	CGGCGGACCTAGTGTTAGTGTT

ebv-miR-BART4	qPCR forward primer	CGGCGGGACCTGATGCTGCTGG
ebv-miR-BART4-3P	qPCR forward primer	CGGCGGCACATCACGTAGGCAC
ebv-miR-BART5	qPCR forward primer	CGGCGGCAAGGTGAATATAGCT
ebv-miR-BART5-3P	qPCR forward primer	CGGCGGGTGGGCCGCTGTTCAC
ebv-miR-BART6-3p	qPCR forward primer	CGGCGGCGGGGATCGGACTAGC
ebv-miR-BART6-5p	qPCR forward primer	CGGCGGTAAGGTTGGTCCAATC
ebv-miR-BART7	qPCR forward primer	CGGCGGCATCATAGTCCAGTGT
ebv-miR-BART7*	qPCR forward primer	CGGCGGCCTGGACCTTGACTAT
ebv-miR-BART9	qPCR forward primer	CGGCGGTAACACTTCATGGGTC
ebv-miR-BART9*	qPCR forward primer	CGGCGGTACTGGACCCTGAATT
universal reverse primer	qPCR reverse primer	CTGGTGTCGTGGAGTCGGCAATTC
