# Supplementary material for: Characterization of Epstein-Barr Virus miRNAome in Nasopharyngeal Carcinoma by Deep Sequencing
Source: PLoS One. 2010 Sep 20;5(9):e12745. doi: 10.1371/journal.pone.0012745 (PMC2942828; doi:10.1371/journal.pone.0012745)
Supplement: Table S2 — Terminal isomiRs of EBV microRNAs in the T10 sample. (0.07 MB PDF) [file pone.0012745.s005.pdf]

Table S2. Terminal isomiRs of EBV microRNAs in the T10 sample

miR_Name	miRBase Name	Sequence	Start	End	Len	Read
BART3-5P	BART3-star	AACCTAGTGTTAGTGTTGT	139086	139104	19	3
BART3-5P	BART3-star	AACCTAGTGTTAGTGTTGTG	139086	139105	20	32
BART3-5P	BART3-star	AACCTAGTGTTAGTGTTGTGC	139086	139106	21	61
BART3-5P	BART3-star	AACCTAGTGTTAGTGTTGTGCT	139086	139107	22	6
BART3-5P	BART3-star	AACCTAGTGTTAGTGTTGTGCTG	139086	139108	23	21
BART3-5P	BART3-star	AACCTAGTGTTAGTGTTGTGCTGT	139086	139109	24	4
BART3-5P	BART3-star	AACCTAGTGTTAGTGTTGTGCTGTA	139086	139110	25	14
BART3-5P	BART3-star	AACCTAGTGTTAGTGTTGTGCTGTAAA	139086	139112	27	3
BART3-5P	BART3-star	ACCTAGTGTTAGTGTTGTG	139087	139105	19	7
BART3-5P	BART3-star	ACCTAGTGTTAGTGTTGTGC	139087	139106	20	3
BART3-5P	BART3-star	ACCTAGTGTTAGTGTTGTGCTG	139087	139108	22	12
BART3-5P	BART3-star	ACCTAGTGTTAGTGTTGTGCTGT	139087	139109	23	76
BART3-5P	BART3-star	ACCTAGTGTTAGTGTTGTGCTGTA	139087	139110	24	3
BART3-3P	BART3	GCGCACCACTAGTCACCAGGT	139123	139143	21	20
BART3-3P	BART3	GCGCACCACTAGTCACCAGGTGT	139123	139145	23	79
BART3-3P	BART3	GCGCACCACTAGTCACCAGGTGTC	139123	139146	24	43
BART3-3P	BART3	GCGCACCACTAGTCACCAGGTGTCA	139123	139147	25	3
BART3-3P	BART3	GCGCACCACTAGTCACCAGGTGTCAC	139123	139148	26	4
BART3-3P	BART3	CGCACCACTAGTCACCAGG	139124	139142	19	130
BART3-3P	BART3	CGCACCACTAGTCACCAGGT	139124	139143	20	299
BART3-3P	BART3	CGCACCACTAGTCACCAGGTG	139124	139144	21	1086
BART3-3P	BART3	CGCACCACTAGTCACCAGGTGT	139124	139145	22	10642
BART3-3P	BART3	CGCACCACTAGTCACCAGGTGTC	139124	139146	23	2582
BART3-3P	BART3	CGCACCACTAGTCACCAGGTGTCA	139124	139147	24	2745
BART3-3P	BART3	CGCACCACTAGTCACCAGGTGTCAC	139124	139148	25	239
BART3-3P	BART3	CGCACCACTAGTCACCAGGTGTCACC	139124	139149	26	28
BART3-3P	BART3	CGCACCACTAGTCACCAGGTGTCACCG	139124	139150	27	3
BART3-3P	BART3	CGCACCACTAGTCACCAGGTGTCACCGG	139124	139151	28	55
BART3-3P	BART3	GCACCACTAGTCACCAGGTGT	139125	139145	21	16
BART3-3P	BART3	GCACCACTAGTCACCAGGTGTC	139125	139146	22	5
BART3-3P	BART3	GCACCACTAGTCACCAGGTGTCA	139125	139147	23	11
BART4-5P	BART4	GACCTGATGCTGCTGGTGT	139228	139246	19	400
BART4-5P	BART4	GACCTGATGCTGCTGGTGTG	139228	139247	20	633
BART4-5P	BART4	GACCTGATGCTGCTGGTGTGC	139228	139248	21	2874
BART4-5P	BART4	GACCTGATGCTGCTGGTGTGCT	139228	139249	22	3365
BART4-5P	BART4	GACCTGATGCTGCTGGTGTGCTG	139228	139250	23	1481
BART4-5P	BART4	GACCTGATGCTGCTGGTGTGCTGT	139228	139251	24	270
BART4-5P	BART4	GACCTGATGCTGCTGGTGTGCTGTA	139228	139252	25	136
BART4-5P	BART4	GACCTGATGCTGCTGGTGTGCTGTAA	139228	139253	26	33
BART4-5P	BART4	GACCTGATGCTGCTGGTGTGCTGTAAA	139228	139254	27	6
BART4-5P	BART4	ACCTGATGCTGCTGGTGTGC	139229	139248	20	3
BART4-5P	BART4	ACCTGATGCTGCTGGTGTGCTG	139229	139250	22	3
BART4-3P	BART4-star	CACATCACGTAGGCACCAGGT	139266	139286	21	5
BART4-3P	BART4-star	CACATCACGTAGGCACCAGGTG	139266	139287	22	20
BART4-3P	BART4-star	CACATCACGTAGGCACCAGGTGT	139266	139288	23	91
BART4-3P	BART4-star	CACATCACGTAGGCACCAGGTGTCA	139266	139290	25	9
BART4-3P	BART4-star	CACATCACGTAGGCACCAGGTGTCACC	139266	139292	27	3
BART1-5P	BART1-5P	TCTTAGTGGAAGTGACGTG	139351	139369	19	16
BART1-5P	BART1-5P	TCTTAGTGGAAGTGACGTGC	139351	139370	20	64
BART1-5P	BART1-5P	TCTTAGTGGAAGTGACGTGCT	139351	139371	21	8
BART1-5P	BART1-5P	TCTTAGTGGAAGTGACGTGCTG	139351	139372	22	94
BART1-5P	BART1-5P	TCTTAGTGGAAGTGACGTGCTGT	139351	139373	23	104
BART1-5P	BART1-5P	TCTTAGTGGAAGTGACGTGCTGTG	139351	139374	24	86
BART1-5P	BART1-5P	TCTTAGTGGAAGTGACGTGCTGTGA	139351	139375	25	60
BART1-5P	BART1-5P	TCTTAGTGGAAGTGACGTGCTGTGAA	139351	139376	26	26
BART1-5P	BART1-5P	CTTAGTGGAAGTGACGTGCTGTG	139352	139374	23	4
BART1-3P	BART1-3P	TAGCACCGCTATCCACTAT	139387	139405	19	41
BART1-3P	BART1-3P	TAGCACCGCTATCCACTATG	139387	139406	20	147
BART1-3P	BART1-3P	TAGCACCGCTATCCACTATGT	139387	139407	21	1656
BART1-3P	BART1-3P	TAGCACCGCTATCCACTATGTC	139387	139408	22	1413
BART1-3P	BART1-3P	TAGCACCGCTATCCACTATGTCT	139387	139409	23	1949
BART1-3P	BART1-3P	TAGCACCGCTATCCACTATGTCTC	139387	139410	24	182
BART1-3P	BART1-3P	TAGCACCGCTATCCACTATGTCTCG	139387	139411	25	11
BART1-3P	BART1-3P	AGCACCGCTATCCACTATGT	139388	139407	20	51
BART1-3P	BART1-3P	AGCACCGCTATCCACTATGTC	139388	139408	21	17

BART1-3P	BART1-3P	AGCACCGCTATCCACTATGTCT	139388	139409	22	84
BART15-5P		AGGGAAACATGACCACCTGAAGTC	139519	139542	24	3
BART15-3P	BART15	GGTCAGTGGTTTTGTTTCCTTGAT	139552	139575	24	3
BART15-3P	BART15	GTCAGTGGTTTTGTTTCCTT	139553	139572	20	5
BART15-3P	BART15	GTCAGTGGTTTTGTTTCCTTG	139553	139573	21	75
BART15-3P	BART15	GTCAGTGGTTTTGTTTCCTTGA	139553	139574	22	1657
BART15-3P	BART15	GTCAGTGGTTTTGTTTCCTTGAT	139553	139575	23	137
BART15-3P	BART15	GTCAGTGGTTTTGTTTCCTTGATAG	139553	139577	25	3
BART15-3P	BART15	GTCAGTGGTTTTGTTTCCTTGATAGA	139553	139578	26	5
BART15-3P	BART15	TCAGTGGTTTTGTTTCCTTGATAG	139554	139577	24	9
BART5-5P	BART5	TCAAGGTGAATATAGCTGCCCAT	139674	139696	23	3
BART5-5P	BART5	TCAAGGTGAATATAGCTGCCCATC	139674	139697	24	14
BART5-5P	BART5	TCAAGGTGAATATAGCTGCCCATCG	139674	139698	25	15
BART5-5P	BART5	TCAAGGTGAATATAGCTGCCCATCGA	139674	139699	26	6
BART5-5P	BART5	CAAGGTGAATATAGCTGCC	139675	139693	19	116
BART5-5P	BART5	CAAGGTGAATATAGCTGCCC	139675	139694	20	81
BART5-5P	BART5	CAAGGTGAATATAGCTGCCCA	139675	139695	21	444
BART5-5P	BART5	CAAGGTGAATATAGCTGCCCAT	139675	139696	22	2746
BART5-5P	BART5	CAAGGTGAATATAGCTGCCCATC	139675	139697	23	2413
BART5-5P	BART5	CAAGGTGAATATAGCTGCCCATCG	139675	139698	24	3805
BART5-5P	BART5	CAAGGTGAATATAGCTGCCCATCGA	139675	139699	25	2114
BART5-5P	BART5	CAAGGTGAATATAGCTGCCCATCGAC	139675	139700	26	171
BART5-5P	BART5	CAAGGTGAATATAGCTGCCCATCGACG	139675	139701	27	12
BART5-5P	BART5	AAGGTGAATATAGCTGCCC	139676	139694	19	13
BART5-5P	BART5	AAGGTGAATATAGCTGCCCA	139676	139695	20	76
BART5-5P	BART5	AAGGTGAATATAGCTGCCCAT	139676	139696	21	614
BART5-5P	BART5	AAGGTGAATATAGCTGCCCATC	139676	139697	22	722
BART5-5P	BART5	AAGGTGAATATAGCTGCCCATCG	139676	139698	23	1206
BART5-5P	BART5	AAGGTGAATATAGCTGCCCATCGA	139676	139699	24	995
BART5-5P	BART5	AAGGTGAATATAGCTGCCCATCGAC	139676	139700	25	57
BART5-5P	BART5	AAGGTGAATATAGCTGCCCATCGACG	139676	139701	26	3
BART5-5P	BART5	AAGGTGAATATAGCTGCCCATCGACGT	139676	139702	27	5
BART5-5P	BART5	AGGTGAATATAGCTGCCCATCGA	139677	139699	23	14
BART5-3P	BART5-star	GTGGGCCGCTGTTCACCTA	139717	139735	19	8
BART5-3P	BART5-star	GTGGGCCGCTGTTCACCTAA	139717	139736	20	149
BART5-3P	BART5-star	GTGGGCCGCTGTTCACCTAAA	139717	139737	21	58
BART5-3P	BART5-star	GTGGGCCGCTGTTCACCTAAAG	139717	139738	22	61
BART5-3P	BART5-star	TGGGCCGCTGTTCACCTAA	139718	139736	19	9
BART5-3P	BART5-star	TGGGCCGCTGTTCACCTAAA	139718	139737	20	4
BART5-3P	BART5-star	TGGGCCGCTGTTCACCTAAAG	139718	139738	21	7
BART5-3P	BART5-star	TGGGCCGCTGTTCACCTAAAGTG	139718	139740	23	4
BART16-5P	BART16	TTAGATAGAGTGGGTGTGT	139795	139813	19	8
BART16-5P	BART16	TTAGATAGAGTGGGTGTGTG	139795	139814	20	3
BART16-5P	BART16	TTAGATAGAGTGGGTGTGTGC	139795	139815	21	53
BART16-5P	BART16	TTAGATAGAGTGGGTGTGTGCT	139795	139816	22	14
BART16-5P	BART16	TTAGATAGAGTGGGTGTGTGCTC	139795	139817	23	19
BART16-5P	BART16	TTAGATAGAGTGGGTGTGTGCTCTT	139795	139819	25	6
BART16-5P	BART16	TTAGATAGAGTGGGTGTGTGCTCTTGTT	139795	139822	28	18
BART16-3P		AGATCACCACCCTCTATCC	139834	139852	19	8
BART16-3P		AGATCACCACCCTCTATCCA	139834	139853	20	35
BART16-3P		AGATCACCACCCTCTATCCAT	139834	139854	21	168
BART16-3P		AGATCACCACCCTCTATCCATA	139834	139855	22	47
BART16-3P		AGATCACCACCCTCTATCCATAT	139834	139856	23	85
BART16-3P		GATCACCACCCTCTATCCAT	139835	139854	20	14
BART16-3P		GATCACCACCCTCTATCCATA	139835	139855	21	5
BART16-3P		GATCACCACCCTCTATCCATAT	139835	139856	22	73
BART16-3P		GATCACCACCCTCTATCCATATC	139835	139857	23	4
BART16-3P		ATCACCACCCTCTATCCAT	139836	139854	19	18
BART16-3P		ATCACCACCCTCTATCCATAT	139836	139856	21	217
BART16-3P		CCCACAATTGATAAACCTC	139857	139875	19	25
BART16-3P		CCCACAATTGATAAACCTCC	139857	139876	20	6
BART17-5P	BART17-5P	TAAGAGGACGCAGGCATAC	139915	139933	19	98
BART17-5P	BART17-5P	TAAGAGGACGCAGGCATACA	139915	139934	20	42
BART17-5P	BART17-5P	TAAGAGGACGCAGGCATACAA	139915	139935	21	43
BART17-5P	BART17-5P	TAAGAGGACGCAGGCATACAAGG	139915	139937	23	4
BART17-5P	BART17-5P	TAAGAGGACGCAGGCATACAAGGT	139915	139938	24	7
BART17-3P	BART17-3P	TTGTATGCCTGGTGTCCCCTTAGT	139952	139975	24	14
BART17-3P	BART17-3P	TGTATGCCTGGTGTCCCCT	139953	139971	19	13

BART17-3P	BART17-3P	TGTATGCCTGGTGTCCCCTT	139953	139972	20	27
BART17-3P	BART17-3P	TGTATGCCTGGTGTCCCCTTA	139953	139973	21	61
BART17-3P	BART17-3P	TGTATGCCTGGTGTCCCCTTAG	139953	139974	22	87
BART17-3P	BART17-3P	TGTATGCCTGGTGTCCCCTTAGT	139953	139975	23	894
BART17-3P	BART17-3P	TGTATGCCTGGTGTCCCCTTAGTG	139953	139976	24	288
BART17-3P	BART17-3P	TGTATGCCTGGTGTCCCCTTAGTGG	139953	139977	25	86
BART17-3P	BART17-3P	TGTATGCCTGGTGTCCCCTTAGTGGG	139953	139978	26	10
BART17-3P	BART17-3P	GTATGCCTGGTGTCCCCTTAGT	139954	139975	22	60
BART17-3P	BART17-3P	GTATGCCTGGTGTCCCCTTAGTG	139954	139976	23	16
BART17-3P	BART17-3P	TATGCCTGGTGTCCCCTTAG	139955	139974	20	3
BART17-3P	BART17-3P	TATGCCTGGTGTCCCCTTAGT	139955	139975	21	5
BART17-3P	BART17-3P	TATGCCTGGTGTCCCCTTAGTG	139955	139976	22	4
BART6-5P	BART6-5P	TAAGGTTGGTCCAATCCATA	140033	140052	20	6
BART6-5P	BART6-5P	TAAGGTTGGTCCAATCCATAG	140033	140053	21	189
BART6-5P	BART6-5P	TAAGGTTGGTCCAATCCATAGG	140033	140054	22	200
BART6-5P	BART6-5P	TAAGGTTGGTCCAATCCATAGGC	140033	140055	23	858
BART6-5P	BART6-5P	TAAGGTTGGTCCAATCCATAGGCT	140033	140056	24	185
BART6-5P	BART6-5P	TAAGGTTGGTCCAATCCATAGGCTT	140033	140057	25	20
BART6-5P	BART6-5P	TAAGGTTGGTCCAATCCATAGGCTTT	140033	140058	26	4
BART6-5P	BART6-5P	TAAGGTTGGTCCAATCCATAGGCTTTT	140033	140059	27	3
BART6-5P	BART6-5P	AAGGTTGGTCCAATCCATAGG	140034	140054	21	9
BART6-5P	BART6-5P	AAGGTTGGTCCAATCCATAGGC	140034	140055	22	8
BART6-3P	BART6-3P	CCGGGGATCGGACTAGCCTTA	140071	140091	21	4
BART6-3P	BART6-3P	CGGGGATCGGACTAGCCTT	140072	140090	19	232
BART6-3P	BART6-3P	CGGGGATCGGACTAGCCTTA	140072	140091	20	399
BART6-3P	BART6-3P	CGGGGATCGGACTAGCCTTAG	140072	140092	21	322
BART6-3P	BART6-3P	CGGGGATCGGACTAGCCTTAGA	140072	140093	22	755
BART6-3P	BART6-3P	CGGGGATCGGACTAGCCTTAGAG	140072	140094	23	134
BART6-3P	BART6-3P	CGGGGATCGGACTAGCCTTAGAGT	140072	140095	24	12
BART6-3P	BART6-3P	CGGGGATCGGACTAGCCTTAGAGTA	140072	140096	25	7
BART21-5P	BART21-5P	TCACTAGTGAAGGCAACTAAC	145514	145534	21	42
BART21-5P	BART21-5P	TCACTAGTGAAGGCAACTAACA	145514	145535	22	85
BART21-5P	BART21-5P	TCACTAGTGAAGGCAACTAACAC	145514	145536	23	25
BART21-5P	BART21-5P	TCACTAGTGAAGGCAACTAACACAG	145514	145538	25	7
BART21-3P	BART21-3P	CTAGTTGTGCCCACTGGTGTTT	145548	145569	22	16
BART18-5P	BART18-5P	TCAAGTTCGCACTTCCTAT	145962	145980	19	53
BART18-5P	BART18-5P	TCAAGTTCGCACTTCCTATA	145962	145981	20	149
BART18-5P	BART18-5P	TCAAGTTCGCACTTCCTATAC	145962	145982	21	487
BART18-5P	BART18-5P	TCAAGTTCGCACTTCCTATACA	145962	145983	22	712
BART18-5P	BART18-5P	TCAAGTTCGCACTTCCTATACAG	145962	145984	23	398
BART18-5P	BART18-5P	TCAAGTTCGCACTTCCTATACAGT	145962	145985	24	4
BART18-5P	BART18-5P	CAAGTTCGCACTTCCTATA	145963	145981	19	3
BART18-5P	BART18-5P	CAAGTTCGCACTTCCTATACA	145963	145983	21	12
BART18-5P	BART18-5P	CAAGTTCGCACTTCCTATACAG	145963	145984	22	35
BART18-3P	BART18-3P	TATCGGAAGTTTGGGCTTCGT	145998	146018	21	18
BART18-3P	BART18-3P	TATCGGAAGTTTGGGCTTCGTC	145998	146019	22	20
BART18-3P	BART18-3P	TATCGGAAGTTTGGGCTTCGTCCC	145998	146021	24	6
BART7-5P	BART7-star	CCTGGACCTTGACTATGAA	146439	146457	19	18
BART7-5P	BART7-star	CCTGGACCTTGACTATGAAA	146439	146458	20	14
BART7-5P	BART7-star	CCTGGACCTTGACTATGAAAC	146439	146459	21	80
BART7-5P	BART7-star	CCTGGACCTTGACTATGAAACA	146439	146460	22	141
BART7-5P	BART7-star	CCTGGACCTTGACTATGAAACAA	146439	146461	23	19
BART7-5P	BART7-star	CCTGGACCTTGACTATGAAACAAT	146439	146462	24	4
BART7-3P	BART7	CATCATAGTCCAGTGTCCA	146475	146493	19	151
BART7-3P	BART7	CATCATAGTCCAGTGTCCAG	146475	146494	20	1000
BART7-3P	BART7	CATCATAGTCCAGTGTCCAGG	146475	146495	21	243
BART7-3P	BART7	CATCATAGTCCAGTGTCCAGGG	146475	146496	22	582
BART7-3P	BART7	CATCATAGTCCAGTGTCCAGGGA	146475	146497	23	2435
BART7-3P	BART7	CATCATAGTCCAGTGTCCAGGGAC	146475	146498	24	2512
BART7-3P	BART7	CATCATAGTCCAGTGTCCAGGGACA	146475	146499	25	1045
BART7-3P	BART7	CATCATAGTCCAGTGTCCAGGGACAG	146475	146500	26	198
BART7-3P	BART7	CATCATAGTCCAGTGTCCAGGGACAGT	146475	146501	27	102
BART7-3P	BART7	CATCATAGTCCAGTGTCCAGGGACAGTG	146475	146502	28	32
BART7-3P	BART7	ATCATAGTCCAGTGTCCAGGGACA	146476	146499	24	13
BART8-5P	BART8	TACGGTTTCCTAGATTGTA	146772	146790	19	38
BART8-5P	BART8	TACGGTTTCCTAGATTGTAC	146772	146791	20	185
BART8-5P	BART8	TACGGTTTCCTAGATTGTACA	146772	146792	21	310
BART8-5P	BART8	TACGGTTTCCTAGATTGTACAG	146772	146793	22	1844

BART8-5P	BART8	TACGGTTTCCTAGATTGTACAGA	146772	146794	23	73
BART8-5P	BART8	TACGGTTTCCTAGATTGTACAGAT	146772	146795	24	26
BART8-5P	BART8	TACGGTTTCCTAGATTGTACAGATG	146772	146796	25	4
BART8-5P	BART8	ACGGTTTCCTAGATTGTACAG	146773	146793	21	18
BART8-3P	BART8-star	GTCACAATCTATGGGGTCG	146807	146825	19	45
BART8-3P	BART8-star	GTCACAATCTATGGGGTCGT	146807	146826	20	114
BART8-3P	BART8-star	GTCACAATCTATGGGGTCGTA	146807	146827	21	520
BART8-3P	BART8-star	GTCACAATCTATGGGGTCGTAG	146807	146828	22	665
BART8-3P	BART8-star	GTCACAATCTATGGGGTCGTAGA	146807	146829	23	2937
BART8-3P	BART8-star	GTCACAATCTATGGGGTCGTAGAC	146807	146830	24	461
BART8-3P	BART8-star	GTCACAATCTATGGGGTCGTAGACA	146807	146831	25	61
BART8-3P	BART8-star	GTCACAATCTATGGGGTCGTAGACAG	146807	146832	26	30
BART8-3P	BART8-star	TCACAATCTATGGGGTCGTA	146808	146827	20	3
BART8-3P	BART8-star	TCACAATCTATGGGGTCGTAG	146808	146828	21	11
BART8-3P	BART8-star	TCACAATCTATGGGGTCGTAGA	146808	146829	22	22
BART9-5P	BART9-star	TACTGGACCCTGAATTGGA	146959	146977	19	16
BART9-5P	BART9-star	TACTGGACCCTGAATTGGAA	146959	146978	20	18
BART9-5P	BART9-star	TACTGGACCCTGAATTGGAAA	146959	146979	21	35
BART9-5P	BART9-star	TACTGGACCCTGAATTGGAAAC	146959	146980	22	54
BART9-5P	BART9-star	TACTGGACCCTGAATTGGAAACA	146959	146981	23	50
BART9-5P	BART9-star	TACTGGACCCTGAATTGGAAACAG	146959	146982	24	10
BART9-5P	BART9-star	TACTGGACCCTGAATTGGAAACAGT	146959	146983	25	4
BART9-5P	BART9-star	ACTGGACCCTGAATTGGAAACA	146960	146981	22	4
BART9-3P	BART9	GTAACACTTCATGGGTCCCG	146996	147015	20	7
BART9-3P	BART9	GTAACACTTCATGGGTCCCGT	146996	147016	21	64
BART9-3P	BART9	GTAACACTTCATGGGTCCCGTA	146996	147017	22	145
BART9-3P	BART9	GTAACACTTCATGGGTCCCGTAG	146996	147018	23	162
BART9-3P	BART9	GTAACACTTCATGGGTCCCGTAGT	146996	147019	24	101
BART9-3P	BART9	GTAACACTTCATGGGTCCCGTAGTG	146996	147020	25	46
BART9-3P	BART9	TAACACTTCATGGGTCCCG	146997	147015	19	198
BART9-3P	BART9	TAACACTTCATGGGTCCCGT	146997	147016	20	388
BART9-3P	BART9	TAACACTTCATGGGTCCCGTA	146997	147017	21	919
BART9-3P	BART9	TAACACTTCATGGGTCCCGTAG	146997	147018	22	2974
BART9-3P	BART9	TAACACTTCATGGGTCCCGTAGT	146997	147019	23	9036
BART9-3P	BART9	TAACACTTCATGGGTCCCGTAGTG	146997	147020	24	1579
BART9-3P	BART9	TAACACTTCATGGGTCCCGTAGTGA	146997	147021	25	239
BART9-3P	BART9	TAACACTTCATGGGTCCCGTAGTGAC	146997	147022	26	37
BART9-3P	BART9	TAACACTTCATGGGTCCCGTAGTGACA	146997	147023	27	12
BART9-3P	BART9	TAACACTTCATGGGTCCCGTAGTGACAA	146997	147024	28	3
BART9-3P	BART9	AACACTTCATGGGTCCCGTA	146998	147017	20	65
BART9-3P	BART9	AACACTTCATGGGTCCCGTAG	146998	147018	21	108
BART9-3P	BART9	AACACTTCATGGGTCCCGTAGT	146998	147019	22	278
BART9-3P	BART9	AACACTTCATGGGTCCCGTAGTG	146998	147020	23	37
BART22-5P		TGCTAGACCCTGGAGTTGAAC	147169	147189	21	5
BART22-5P		TGCTAGACCCTGGAGTTGAACC	147169	147190	22	5
BART22-3P	BART22	TTACAAAGTCATGGTCTAGTA	147203	147223	21	20
BART22-3P	BART22	TTACAAAGTCATGGTCTAGTAG	147203	147224	22	136
BART22-3P	BART22	TTACAAAGTCATGGTCTAGTAGT	147203	147225	23	260
BART22-3P	BART22	TTACAAAGTCATGGTCTAGTAGTT	147203	147226	24	53
BART22-3P	BART22	TACAAAGTCATGGTCTAGTAG	147204	147224	21	9
BART22-3P	BART22	TACAAAGTCATGGTCTAGTAGT	147204	147225	22	44
BART22-3P	BART22	TACAAAGTCATGGTCTAGTAGTT	147204	147226	23	12
BART10-5P	BART10-star	GGGCCACCTCTTTGGTTCTGTA	147319	147340	22	5
BART10-5P	BART10-star	GCCACCTCTTTGGTTCTGTA	147321	147340	20	46
BART10-5P	BART10-star	GCCACCTCTTTGGTTCTGTAC	147321	147341	21	107
BART10-3P	BART10	TACATAACCATGGAGTTGG	147356	147374	19	5
BART10-3P	BART10	TACATAACCATGGAGTTGGC	147356	147375	20	23
BART10-3P	BART10	TACATAACCATGGAGTTGGCT	147356	147376	21	28
BART10-3P	BART10	TACATAACCATGGAGTTGGCTG	147356	147377	22	121
BART10-3P	BART10	TACATAACCATGGAGTTGGCTGT	147356	147378	23	1652
BART10-3P	BART10	TACATAACCATGGAGTTGGCTGTG	147356	147379	24	143
BART10-3P	BART10	TACATAACCATGGAGTTGGCTGTGG	147356	147380	25	48
BART10-3P	BART10	ACATAACCATGGAGTTGGC	147357	147375	19	6
BART10-3P	BART10	ACATAACCATGGAGTTGGCT	147357	147376	20	50
BART10-3P	BART10	ACATAACCATGGAGTTGGCTG	147357	147377	21	65
BART10-3P	BART10	ACATAACCATGGAGTTGGCTGT	147357	147378	22	1275
BART10-3P	BART10	ACATAACCATGGAGTTGGCTGTG	147357	147379	23	99
BART10-3P	BART10	ACATAACCATGGAGTTGGCTGTGG	147357	147380	24	41

BART10-3P	BART10	CATAACCATGGAGTTGGCTGT	147358	147378	21	33
BART10-3P	BART10	CATAACCATGGAGTTGGCTGTGG	147358	147380	23	21
BART10-3P	BART10	CATAACCATGGAGTTGGCTGTGGT	147358	147381	24	5
BART10-3P	BART10	ATAACCATGGAGTTGGCTGT	147359	147378	20	3
BART10-3P	BART10	ATAACCATGGAGTTGGCTGTGG	147359	147380	22	15
BART11-5P	BART11-5P	TCAGACAGTTTGGTGCGCTAG	147537	147557	21	12
BART11-5P	BART11-5P	TCAGACAGTTTGGTGCGCTAGT	147537	147558	22	99
BART11-5P	BART11-5P	TCAGACAGTTTGGTGCGCTAGTT	147537	147559	23	106
BART11-5P	BART11-5P	TCAGACAGTTTGGTGCGCTAGTTG	147537	147560	24	78
BART11-5P	BART11-5P	TCAGACAGTTTGGTGCGCTAGTTGT	147537	147561	25	38
BART11-5P	BART11-5P	TCAGACAGTTTGGTGCGCTAGTTGTG	147537	147562	26	17
BART11-3P	BART11-3P	AACGCACACCAGGCTGACTGCCT	147574	147596	23	3
BART11-3P	BART11-3P	ACGCACACCAGGCTGACTG	147575	147593	19	10
BART11-3P	BART11-3P	ACGCACACCAGGCTGACTGC	147575	147594	20	365
BART11-3P	BART11-3P	ACGCACACCAGGCTGACTGCC	147575	147595	21	194
BART11-3P	BART11-3P	ACGCACACCAGGCTGACTGCCT	147575	147596	22	258
BART11-3P	BART11-3P	ACGCACACCAGGCTGACTGCCTT	147575	147597	23	37
BART11-3P	BART11-3P	ACGCACACCAGGCTGACTGCCTTA	147575	147598	24	48
BART11-3P	BART11-3P	ACGCACACCAGGCTGACTGCCTTAGCAG	147575	147602	28	3
BART12-5P		CACCCGCCCATCACCACCGGA	147900	147920	21	12
BART12-5P		CACCCGCCCATCACCACCGGACAG	147900	147923	24	11
BART12-5P		ACCCGCCCATCACCACCGGA	147901	147920	20	5
BART12-5P		ACCCGCCCATCACCACCGGAC	147901	147921	21	22
BART12-5P		ACCCGCCCATCACCACCGGACA	147901	147922	22	13
BART12-5P		ACCCGCCCATCACCACCGGACAG	147901	147923	23	19
BART12-3P	BART12	TCCTGTGGTGTTTGGTGTGG	147936	147955	20	4
BART12-3P	BART12	TCCTGTGGTGTTTGGTGTGGT	147936	147956	21	8
BART12-3P	BART12	TCCTGTGGTGTTTGGTGTGGTT	147936	147957	22	16
BART12-3P	BART12	TCCTGTGGTGTTTGGTGTGGTTT	147936	147958	23	62
BART12-3P	BART12	TCCTGTGGTGTTTGGTGTGGTTTT	147936	147959	24	47
BART12-3P	BART12	TCCTGTGGTGTTTGGTGTGGTTTTG	147936	147960	25	5
BART19-5P	BART19-5P	CAACATTCCCCGCAAACATGA	148213	148233	21	3
BART19-5P	BART19-5P	CAACATTCCCCGCAAACATGAC	148213	148234	22	3
BART19-5P	BART19-5P	ACATTCCCCGCAAACATGA	148215	148233	19	460
BART19-5P	BART19-5P	ACATTCCCCGCAAACATGAC	148215	148234	20	447
BART19-5P	BART19-5P	ACATTCCCCGCAAACATGACA	148215	148235	21	1421
BART19-5P	BART19-5P	ACATTCCCCGCAAACATGACAT	148215	148236	22	3219
BART19-5P	BART19-5P	ACATTCCCCGCAAACATGACATG	148215	148237	23	1459
BART19-5P	BART19-5P	ACATTCCCCGCAAACATGACATGG	148215	148238	24	632
BART19-5P	BART19-5P	ACATTCCCCGCAAACATGACATGGG	148215	148239	25	54
BART19-5P	BART19-5P	CATTCCCCGCAAACATGACAT	148216	148236	21	3
BART19-5P	BART19-5P	ATTCCCCGCAAACATGACAT	148217	148236	20	9
BART19-5P	BART19-5P	ATTCCCCGCAAACATGACATG	148217	148237	21	3
BART19-3P	BART19-3P	TGTTTTGTTTGCTTGGGAATGC	148252	148273	22	3
BART19-3P	BART19-3P	TGTTTTGTTTGCTTGGGAATGCT	148252	148274	23	8
BART19-3P	BART19-3P	TGTTTTGTTTGCTTGGGAATGCTC	148252	148275	24	4
BART19-3P	BART19-3P	TTTTGTTTGCTTGGGAATGCT	148254	148274	21	3
BART19-3P	BART19-3P	TTTTGTTTGCTTGGGAATGCTC	148254	148275	22	12
BART19-3P	BART19-3P	TTTTGTTTGCTTGGGAATGCTCT	148254	148276	23	3
BART19-3P	BART19-3P	TTTTGTTTGCTTGGGAATGCTCTT	148254	148277	24	3
BART20-5P	BART20-5P	TAGCAGGCATGTCTTCATTC	148339	148358	20	3
BART20-5P	BART20-5P	TAGCAGGCATGTCTTCATTCC	148339	148359	21	3
BART20-3P	BART20-3P	CATGAAGGCACAGCCTGTTA	148374	148393	20	10
BART20-3P	BART20-3P	CATGAAGGCACAGCCTGTTAC	148374	148394	21	15
BART20-3P	BART20-3P	CATGAAGGCACAGCCTGTTACC	148374	148395	22	135
BART20-3P	BART20-3P	CATGAAGGCACAGCCTGTTACCA	148374	148396	23	3
BART20-3P	BART20-3P	CATGAAGGCACAGCCTGTTACCATTGG	148374	148400	27	3
BART13-5P	BART13-star	AACCGGCTCGTGGCTCGTA	148526	148544	19	14
BART13-5P	BART13-star	AACCGGCTCGTGGCTCGTAC	148526	148545	20	45
BART13-5P	BART13-star	AACCGGCTCGTGGCTCGTACA	148526	148546	21	76
BART13-5P	BART13-star	AACCGGCTCGTGGCTCGTACAG	148526	148547	22	57
BART13-5P	BART13-star	AACCGGCTCGTGGCTCGTACAGA	148526	148548	23	123
BART13-5P	BART13-star	AACCGGCTCGTGGCTCGTACAGAC	148526	148549	24	18
BART13-3P	BART13	TGTAACTTGCCAGGGACGGC	148563	148582	20	4
BART13-3P	BART13	TGTAACTTGCCAGGGACGGCT	148563	148583	21	7
BART13-3P	BART13	TGTAACTTGCCAGGGACGGCTG	148563	148584	22	9
BART13-3P	BART13	TGTAACTTGCCAGGGACGGCTGA	148563	148585	23	239
BART13-3P	BART13	TGTAACTTGCCAGGGACGGCTGAC	148563	148586	24	36

BART13-3P	BART13	TGTAACTTGCCAGGGACGGCTGACG	148563	148587	25	34
BART13-3P	BART13	TGTAACTTGCCAGGGACGGCTGACGAT	148563	148589	27	5
BART14-5P	BART14-star	TACCCTACGCTGCCGATTT	148744	148762	19	28
BART14-5P	BART14-star	TACCCTACGCTGCCGATTTA	148744	148763	20	32
BART14-5P	BART14-star	TACCCTACGCTGCCGATTTAC	148744	148764	21	73
BART14-5P	BART14-star	TACCCTACGCTGCCGATTTACA	148744	148765	22	123
BART14-5P	BART14-star	TACCCTACGCTGCCGATTTACAT	148744	148766	23	23
BART14-5P	BART14-star	TACCCTACGCTGCCGATTTACATA	148744	148767	24	3
BART14-5P	BART14-star	ACCCTACGCTGCCGATTTAC	148745	148764	20	46
BART14-5P	BART14-star	ACCCTACGCTGCCGATTTACA	148745	148765	21	11
BART14-5P	BART14-star	ACCCTACGCTGCCGATTTACAT	148745	148766	22	17
BART14-5P	BART14-star	CCCTACGCTGCCGATTTACAT	148746	148766	21	4
BART14-3P	BART14	TAAATGCTGCAGTAGTAGGG	148778	148797	20	4
BART14-3P	BART14	TAAATGCTGCAGTAGTAGGGA	148778	148798	21	10
BART14-3P	BART14	TAAATGCTGCAGTAGTAGGGAT	148778	148799	22	27
BART14-3P	BART14	TAAATGCTGCAGTAGTAGGGATC	148778	148800	23	31
BART14-3P	BART14	TAAATGCTGCAGTAGTAGGGATCT	148778	148801	24	8
BART14-3P	BART14	AAATGCTGCAGTAGTAGGGAT	148779	148799	21	13
BART2-5P	BART2-5P	TATTTTCTGCATTCGCCCT	152747	152765	19	12
BART2-5P	BART2-5P	TATTTTCTGCATTCGCCCTT	152747	152766	20	78
BART2-5P	BART2-5P	TATTTTCTGCATTCGCCCTTG	152747	152767	21	201
BART2-5P	BART2-5P	TATTTTCTGCATTCGCCCTTGC	152747	152768	22	1013
BART2-5P	BART2-5P	TATTTTCTGCATTCGCCCTTGCG	152747	152769	23	111
BART2-5P	BART2-5P	TATTTTCTGCATTCGCCCTTGCGT	152747	152770	24	15
BART2-5P	BART2-5P	TATTTTCTGCATTCGCCCTTGCGTGTCC	152747	152774	28	11
BART2-5P	BART2-5P	ATTTTCTGCATTCGCCCTT	152748	152766	19	5
BART2-5P	BART2-5P	ATTTTCTGCATTCGCCCTTG	152748	152767	20	4
BART2-5P	BART2-5P	ATTTTCTGCATTCGCCCTTGC	152748	152768	21	20
BART2-3P	BART2-3P	AAGGAGCGATTTGGAGAAAATA	152783	152804	22	4
BART2-3P	BART2-3P	AAGGAGCGATTTGGAGAAAATAA	152783	152805	23	8
