# Supplementary material for: Characterization of Epstein-Barr Virus miRNAome in Nasopharyngeal Carcinoma by Deep Sequencing
Source: PLoS One. 2010 Sep 20;5(9):e12745. doi: 10.1371/journal.pone.0012745 (PMC2942828; doi:10.1371/journal.pone.0012745)
Supplement: Table S3 — Nucleotide variants of EBV microRNAs in the T10 sample. (0.59 MB PDF) [file pone.0012745.s006.pdf]

Table S3. Nucleotide variants of EBV microRNAs in the T10 samples

EBV miRNA	miRBase Name	Sequence	Start	End	length	Reads
BART3-5P	BART3-star	AACCTAGTGTTAGTGTTGT	139086	139104	19	3
BART3-5P	BART3-star	AACCTAGTGTTAGTGTTGTa	139086	139105	20	15
BART3-5P	BART3-star	AACCTAGTGTTAGTGTTGTG	139086	139105	20	17
BART3-5P	BART3-star	AACCTAGTGTTAGTGaTGTGC	139086	139106	21	4
BART3-5P	BART3-star	AACCTAGTGTTAGTGgTGTGC	139086	139106	21	5
BART3-5P	BART3-star	AACCTAGTGTTAGTGTaGTGC	139086	139106	21	9
BART3-5P	BART3-star	AACCTAGTGTTAGTGTTGTGC	139086	139106	21	43
BART3-5P	BART3-star	AACCTAGTGTTAGTGTcGTGCT	139086	139107	22	3
BART3-5P	BART3-star	AACCTAGTGTTAGTGTTGTGCT	139086	139107	22	3
BART3-5P	BART3-star	AACCTAGTGTTAGTGTTGTGCTc	139086	139108	23	4
BART3-5P	BART3-star	AACCTAGTGTTAGTGTTGTGCTG	139086	139108	23	17
BART3-5P	BART3-star	AACCTAGTGTTAGTGTTGTGCTaT	139086	139109	24	4
BART3-5P	BART3-star	AACCTAGTGTTAGTGTTGTGCTaaA	139086	139110	25	4
BART3-5P	BART3-star	AACCTAGTGTTAGTGTTGTGCTtaA	139086	139110	25	10
BART3-5P	BART3-star	AACCTAGTGTTAGTGTTGTGCTGagAA	139086	139112	27	3
BART3-5P	BART3-star	ACCTAGTGTTAGTGTcGTG	139087	139105	19	4
BART3-5P	BART3-star	ACCTAGTGTTAGTGTTGTG	139087	139105	19	3
BART3-5P	BART3-star	ACCTAGTGTTAGTGTTGTGC	139087	139106	20	3
BART3-5P	BART3-star	ACCTAGTGTTAGTGTTGTGCac	139087	139108	22	4
BART3-5P	BART3-star	ACCTAGTGTTAGTGTTGTGCTG	139087	139108	22	3
BART3-5P	BART3-star	ACCTAGTGTTAGTGTTGTGtTG	139087	139108	22	5
BART3-5P	BART3-star	ACCTAGTGTTAGTGTTGacCTGT	139087	139109	23	4
BART3-5P	BART3-star	ACCTAGTGTTAGTGTTGaGCTGT	139087	139109	23	3
BART3-5P	BART3-star	ACCTAGTGTTAGTGTTGcaCTGT	139087	139109	23	18
BART3-5P	BART3-star	ACCTAGTGTTAGTGTTGccCTGT	139087	139109	23	3
BART3-5P	BART3-star	ACCTAGTGTTAGTGTTGcGCTGT	139087	139109	23	3
BART3-5P	BART3-star	ACCTAGTGTTAGTGTTGTaCTGT	139087	139109	23	9
BART3-5P	BART3-star	ACCTAGTGTTAGTGTTGTGCTGT	139087	139109	23	31
BART3-5P	BART3-star	cCCTAGTGTTAGTGTTGTGCTtT	139087	139109	23	5
BART3-5P	BART3-star	ACCTAGTGTTAGTGTTGTGCTGcA	139087	139110	24	3
BART3-3P	BART3	GCGCACCACTAGTCACCAGGc	139123	139143	21	3
BART3-3P	BART3	GCGCACCACTAGTCAgCAGGT	139123	139143	21	17
BART3-3P	BART3	GCGCACCACTAGTCACCAaGTGT	139123	139145	23	17
BART3-3P	BART3	GCGCACCACTAGTCACCAGaTaT	139123	139145	23	3
BART3-3P	BART3	GCGCACCACTAGTCACCAGaTGT	139123	139145	23	9
BART3-3P	BART3	GCGCACCACTAGTCACCAGGaGa	139123	139145	23	7
BART3-3P	BART3	GCGCACCACTAGTCACCAGGTaT	139123	139145	23	7
BART3-3P	BART3	GCGCACCACTAGTCACCAGGTGa	139123	139145	23	4
BART3-3P	BART3	GCGCACCACTAGTCACCAGGTGT	139123	139145	23	16
BART3-3P	BART3	GCGCACCACTAGTCACCAtGTGT	139123	139145	23	3
BART3-3P	BART3	GCGCACCACTAGTCACCgGGTGT	139123	139145	23	13
BART3-3P	BART3	GCGCACCACTAGTCACCAaGTGTg	139123	139146	24	10
BART3-3P	BART3	GCGCACCACTAGTCACCAGGaGTa	139123	139146	24	4
BART3-3P	BART3	GCGCACCACTAGTCACCAGGTGTa	139123	139146	24	17
BART3-3P	BART3	GCGCACCACTAGTCACCAGGTGTC	139123	139146	24	6
BART3-3P	BART3	GCGCACCACTAGTCACCAGGTGTg	139123	139146	24	3
BART3-3P	BART3	GCGCACCACTAGTCACCAGGTGTt	139123	139146	24	3
BART3-3P	BART3	GCGCACCACTAGTCACCAGGTGTat	139123	139147	25	3
BART3-3P	BART3	GCGCACCACTAGTCACCAGGTGTtAa	139123	139148	26	4
BART3-3P	BART3	CGCACCACTAGTCAaCAGa	139124	139142	19	4
BART3-3P	BART3	CGCACCACTAGTCACCAac	139124	139142	19	3
BART3-3P	BART3	CGCACCACTAGTCACCAcc	139124	139142	19	10
BART3-3P	BART3	CGCACCACTAGTCACCAcG	139124	139142	19	4
BART3-3P	BART3	CGCACCACTAGTCACCAct	139124	139142	19	5
BART3-3P	BART3	CGCACCACTAGTCACCAGa	139124	139142	19	15
BART3-3P	BART3	CGCACCACTAGTCACCAGc	139124	139142	19	6
BART3-3P	BART3	CGCACCACTAGTCACCAGG	139124	139142	19	31
BART3-3P	BART3	CGCACCACTAGTCACCAGt	139124	139142	19	8
BART3-3P	BART3	CGCACCACTAGTCACCAtc	139124	139142	19	3
BART3-3P	BART3	CGCACCACTAGTCACCAtG	139124	139142	19	5
BART3-3P	BART3	CGCACCACTAGTCACCccG	139124	139142	19	3
BART3-3P	BART3	CGCACCACTAGTCACCcGG	139124	139142	19	6
BART3-3P	BART3	CGCACCACTAGTCACCcGt	139124	139142	19	8
BART3-3P	BART3	CGCACCACTAGTCACCtGt	139124	139142	19	5
BART3-3P	BART3	CGCACCACTAGTCACtAGc	139124	139142	19	3

BART3-3P	BART3	CGCACCACTAGTCACtAGt	139124	139142	19	3
BART3-3P	BART3	CGCACCACTAGTCACtgGG	139124	139142	19	8
BART3-3P	BART3	CGCACCACTAGTCACCAcGT	139124	139143	20	10
BART3-3P	BART3	CGCACCACTAGTCACCAGac	139124	139143	20	4
BART3-3P	BART3	CGCACCACTAGTCACCAGag	139124	139143	20	9
BART3-3P	BART3	CGCACCACTAGTCACCAGaT	139124	139143	20	28
BART3-3P	BART3	CGCACCACTAGTCACCAGGa	139124	139143	20	13
BART3-3P	BART3	CGCACCACTAGTCACCAGGc	139124	139143	20	9
BART3-3P	BART3	CGCACCACTAGTCACCAGGg	139124	139143	20	3
BART3-3P	BART3	CGCACCACTAGTCACCAGGT	139124	139143	20	76
BART3-3P	BART3	CGCACCACTAGTCACCAGtT	139124	139143	20	7
BART3-3P	BART3	CGCACCACTAGTCACCAtac	139124	139143	20	10
BART3-3P	BART3	CGCACCACTAGTCACCAtGc	139124	139143	20	9
BART3-3P	BART3	CGCACCACTAGTCACCAtGT	139124	139143	20	29
BART3-3P	BART3	CGCACCACTAGTCACCccGa	139124	139143	20	4
BART3-3P	BART3	CGCACCACTAGTCACCcGGg	139124	139143	20	3
BART3-3P	BART3	CGCACCACTAGTCACCctGT	139124	139143	20	3
BART3-3P	BART3	CGCACCACTAGTCACCgtGa	139124	139143	20	4
BART3-3P	BART3	CGCACCACTAGTCACCgtGc	139124	139143	20	3
BART3-3P	BART3	CGCACCACTAGTCACCgtGT	139124	139143	20	11
BART3-3P	BART3	CGCACCACTAGTCACCtGaT	139124	139143	20	12
BART3-3P	BART3	CGCACCACTAGTCACCtGGT	139124	139143	20	16
BART3-3P	BART3	CGCACCACTAGTCACCttGc	139124	139143	20	9
BART3-3P	BART3	CGCACCACTAGTCACCttGT	139124	139143	20	14
BART3-3P	BART3	CGCACCACTAGTCACtAGGa	139124	139143	20	6
BART3-3P	BART3	CGCACCACTAGTCACtAGGT	139124	139143	20	3
BART3-3P	BART3	CGCACCACTAGTCAgCAGGT	139124	139143	20	4
BART3-3P	BART3	aGCACCACTAGTCACCAGGTG	139124	139144	21	3
BART3-3P	BART3	CGCACCACTAGTCACaAGGTG	139124	139144	21	3
BART3-3P	BART3	CGCACCACTAGTCACCAaGTG	139124	139144	21	6
BART3-3P	BART3	CGCACCACTAGTCACCAcaTG	139124	139144	21	5
BART3-3P	BART3	CGCACCACTAGTCACCAcGTc	139124	139144	21	8
BART3-3P	BART3	CGCACCACTAGTCACCAcGTG	139124	139144	21	17
BART3-3P	BART3	CGCACCACTAGTCACCAcGTt	139124	139144	21	4
BART3-3P	BART3	CGCACCACTAGTCACCActaG	139124	139144	21	3
BART3-3P	BART3	CGCACCACTAGTCACCAGaTa	139124	139144	21	6
BART3-3P	BART3	CGCACCACTAGTCACCAGaTc	139124	139144	21	6
BART3-3P	BART3	CGCACCACTAGTCACCAGaTG	139124	139144	21	43
BART3-3P	BART3	CGCACCACTAGTCACCAGcac	139124	139144	21	39
BART3-3P	BART3	CGCACCACTAGTCACCAGcaG	139124	139144	21	8
BART3-3P	BART3	CGCACCACTAGTCACCAGcTG	139124	139144	21	22
BART3-3P	BART3	CGCACCACTAGTCACCAGGac	139124	139144	21	3
BART3-3P	BART3	CGCACCACTAGTCACCAGGaG	139124	139144	21	37
BART3-3P	BART3	CGCACCACTAGTCACCAGGca	139124	139144	21	6
BART3-3P	BART3	CGCACCACTAGTCACCAGGcG	139124	139144	21	7
BART3-3P	BART3	CGCACCACTAGTCACCAGGgG	139124	139144	21	14
BART3-3P	BART3	CGCACCACTAGTCACCAGGgt	139124	139144	21	5
BART3-3P	BART3	CGCACCACTAGTCACCAGGTa	139124	139144	21	37
BART3-3P	BART3	CGCACCACTAGTCACCAGGTc	139124	139144	21	37
BART3-3P	BART3	CGCACCACTAGTCACCAGGTG	139124	139144	21	392
BART3-3P	BART3	CGCACCACTAGTCACCAGGTt	139124	139144	21	48
BART3-3P	BART3	CGCACCACTAGTCACCAGtcG	139124	139144	21	3
BART3-3P	BART3	CGCACCACTAGTCACCAGtgt	139124	139144	21	4
BART3-3P	BART3	CGCACCACTAGTCACCAGtTG	139124	139144	21	15
BART3-3P	BART3	CGCACCACTAGTCACCAtGTc	139124	139144	21	3
BART3-3P	BART3	CGCACCACTAGTCACCAtGTG	139124	139144	21	28
BART3-3P	BART3	CGCACCACTAGTCACCAtGTt	139124	139144	21	3
BART3-3P	BART3	CGCACCACTAGTCACCgaGTG	139124	139144	21	5
BART3-3P	BART3	CGCACCACTAGTCACCgcGTG	139124	139144	21	4
BART3-3P	BART3	CGCACCACTAGTCACCgGGTa	139124	139144	21	5
BART3-3P	BART3	CGCACCACTAGTCACCgGGTG	139124	139144	21	156
BART3-3P	BART3	CGCACCACTAGTCACCgtGTG	139124	139144	21	12
BART3-3P	BART3	CGCACCACTAGTCACCtGGac	139124	139144	21	14
BART3-3P	BART3	CGCACCACTAGTCACCtGGTG	139124	139144	21	32
BART3-3P	BART3	CGCACCACTAGTCACCttGTG	139124	139144	21	10
BART3-3P	BART3	CGCACCACTAGTCACgAGGTG	139124	139144	21	3
BART3-3P	BART3	CGCACCACTAGTCACtAGaTG	139124	139144	21	3
BART3-3P	BART3	CGCACCACTAGTCACtAGGTa	139124	139144	21	4

BART3-3P	BART3	CGCACCACTAGTCACtAGGTG	139124	139144	21	20
BART3-3P	BART3	tGCACCACTAGTCACCAGGTG	139124	139144	21	3
BART3-3P	BART3	aGCACCACTAGTCACCAGGTGT	139124	139145	22	28
BART3-3P	BART3	CaCACCACTAGTCACCAGaTGT	139124	139145	22	7
BART3-3P	BART3	CaCACCACTAGTCACCAGGTGT	139124	139145	22	15
BART3-3P	BART3	CGCACaACTAGTCACCAGGTGT	139124	139145	22	8
BART3-3P	BART3	CGCACCACTAGTCAaaAGGTGT	139124	139145	22	4
BART3-3P	BART3	CGCACCACTAGTCACactGTGT	139124	139145	22	5
BART3-3P	BART3	CGCACCACTAGTCACCAaacGT	139124	139145	22	16
BART3-3P	BART3	CGCACCACTAGTCACCAaaTGc	139124	139145	22	11
BART3-3P	BART3	CGCACCACTAGTCACCAaaTGT	139124	139145	22	71
BART3-3P	BART3	CGCACCACTAGTCACCAaccGT	139124	139145	22	9
BART3-3P	BART3	CGCACCACTAGTCACCAacTGa	139124	139145	22	3
BART3-3P	BART3	CGCACCACTAGTCACCAacTGT	139124	139145	22	5
BART3-3P	BART3	CGCACCACTAGTCACCAaGaGT	139124	139145	22	8
BART3-3P	BART3	CGCACCACTAGTCACCAaGcGT	139124	139145	22	10
BART3-3P	BART3	CGCACCACTAGTCACCAaGgtc	139124	139145	22	3
BART3-3P	BART3	CGCACCACTAGTCACCAaGTaT	139124	139145	22	10
BART3-3P	BART3	CGCACCACTAGTCACCAaGTca	139124	139145	22	6
BART3-3P	BART3	CGCACCACTAGTCACCAaGTGa	139124	139145	22	40
BART3-3P	BART3	CGCACCACTAGTCACCAaGTGc	139124	139145	22	24
BART3-3P	BART3	CGCACCACTAGTCACCAaGTGg	139124	139145	22	4
BART3-3P	BART3	CGCACCACTAGTCACCAaGTGT	139124	139145	22	465
BART3-3P	BART3	CGCACCACTAGTCACCAcaTGT	139124	139145	22	13
BART3-3P	BART3	CGCACCACTAGTCACCAccTca	139124	139145	22	3
BART3-3P	BART3	CGCACCACTAGTCACCAccTGT	139124	139145	22	9
BART3-3P	BART3	CGCACCACTAGTCACCAcGgtc	139124	139145	22	7
BART3-3P	BART3	CGCACCACTAGTCACCAcGTGa	139124	139145	22	4
BART3-3P	BART3	CGCACCACTAGTCACCAcGTGT	139124	139145	22	52
BART3-3P	BART3	CGCACCACTAGTCACCActaGc	139124	139145	22	4
BART3-3P	BART3	CGCACCACTAGTCACCActaGT	139124	139145	22	3
BART3-3P	BART3	CGCACCACTAGTCACCAGacGa	139124	139145	22	3
BART3-3P	BART3	CGCACCACTAGTCACCAGacGc	139124	139145	22	5
BART3-3P	BART3	CGCACCACTAGTCACCAGacGT	139124	139145	22	61
BART3-3P	BART3	CGCACCACTAGTCACCAGagGT	139124	139145	22	7
BART3-3P	BART3	CGCACCACTAGTCACCAGaTaT	139124	139145	22	13
BART3-3P	BART3	CGCACCACTAGTCACCAGaTGa	139124	139145	22	49
BART3-3P	BART3	CGCACCACTAGTCACCAGaTGc	139124	139145	22	20
BART3-3P	BART3	CGCACCACTAGTCACCAGaTGT	139124	139145	22	712
BART3-3P	BART3	CGCACCACTAGTCACCAGcaGT	139124	139145	22	8
BART3-3P	BART3	CGCACCACTAGTCACCAGccGT	139124	139145	22	10
BART3-3P	BART3	CGCACCACTAGTCACCAGcTGT	139124	139145	22	25
BART3-3P	BART3	CGCACCACTAGTCACCAGGaaa	139124	139145	22	7
BART3-3P	BART3	CGCACCACTAGTCACCAGGaaT	139124	139145	22	16
BART3-3P	BART3	CGCACCACTAGTCACCAGGaca	139124	139145	22	9
BART3-3P	BART3	CGCACCACTAGTCACCAGGacc	139124	139145	22	6
BART3-3P	BART3	CGCACCACTAGTCACCAGGacT	139124	139145	22	4
BART3-3P	BART3	CGCACCACTAGTCACCAGGaGa	139124	139145	22	25
BART3-3P	BART3	CGCACCACTAGTCACCAGGaGc	139124	139145	22	11
BART3-3P	BART3	CGCACCACTAGTCACCAGGaGT	139124	139145	22	58
BART3-3P	BART3	CGCACCACTAGTCACCAGGatg	139124	139145	22	5
BART3-3P	BART3	CGCACCACTAGTCACCAGGccT	139124	139145	22	4
BART3-3P	BART3	CGCACCACTAGTCACCAGGcGT	139124	139145	22	42
BART3-3P	BART3	CGCACCACTAGTCACCAGGcta	139124	139145	22	25
BART3-3P	BART3	CGCACCACTAGTCACCAGGgaa	139124	139145	22	3
BART3-3P	BART3	CGCACCACTAGTCACCAGGgaT	139124	139145	22	11
BART3-3P	BART3	CGCACCACTAGTCACCAGGgcc	139124	139145	22	3
BART3-3P	BART3	CGCACCACTAGTCACCAGGgGa	139124	139145	22	4
BART3-3P	BART3	CGCACCACTAGTCACCAGGgGT	139124	139145	22	19
BART3-3P	BART3	CGCACCACTAGTCACCAGGgta	139124	139145	22	16
BART3-3P	BART3	CGCACCACTAGTCACCAGGgtc	139124	139145	22	9
BART3-3P	BART3	CGCACCACTAGTCACCAGGgtg	139124	139145	22	3
BART3-3P	BART3	CGCACCACTAGTCACCAGGgtT	139124	139145	22	4
BART3-3P	BART3	CGCACCACTAGTCACCAGGTaa	139124	139145	22	69
BART3-3P	BART3	CGCACCACTAGTCACCAGGTac	139124	139145	22	23
BART3-3P	BART3	CGCACCACTAGTCACCAGGTaT	139124	139145	22	255
BART3-3P	BART3	CGCACCACTAGTCACCAGGTca	139124	139145	22	53
BART3-3P	BART3	CGCACCACTAGTCACCAGGTcc	139124	139145	22	3

BART3-3P	BART3	CGCACCACTAGTCACCAGGTcg	139124	139145	22	6
BART3-3P	BART3	CGCACCACTAGTCACCAGGTcT	139124	139145	22	4
BART3-3P	BART3	CGCACCACTAGTCACCAGGTGa	139124	139145	22	63
BART3-3P	BART3	CGCACCACTAGTCACCAGGTGc	139124	139145	22	412
BART3-3P	BART3	CGCACCACTAGTCACCAGGTGg	139124	139145	22	24
BART3-3P	BART3	CGCACCACTAGTCACCAGGTGT	139124	139145	22	6064
BART3-3P	BART3	CGCACCACTAGTCACCAGGTta	139124	139145	22	9
BART3-3P	BART3	CGCACCACTAGTCACCAGGTtc	139124	139145	22	4
BART3-3P	BART3	CGCACCACTAGTCACCAGGTtg	139124	139145	22	28
BART3-3P	BART3	CGCACCACTAGTCACCAGGTtT	139124	139145	22	12
BART3-3P	BART3	CGCACCACTAGTCACCAGtaGT	139124	139145	22	15
BART3-3P	BART3	CGCACCACTAGTCACCAGtgGT	139124	139145	22	19
BART3-3P	BART3	CGCACCACTAGTCACCAGtgtT	139124	139145	22	4
BART3-3P	BART3	CGCACCACTAGTCACCAGtTaT	139124	139145	22	9
BART3-3P	BART3	CGCACCACTAGTCACCAGtTGc	139124	139145	22	8
BART3-3P	BART3	CGCACCACTAGTCACCAGtTGT	139124	139145	22	52
BART3-3P	BART3	CGCACCACTAGTCACCAtacGT	139124	139145	22	6
BART3-3P	BART3	CGCACCACTAGTCACCAtagGT	139124	139145	22	6
BART3-3P	BART3	CGCACCACTAGTCACCAtaTGT	139124	139145	22	10
BART3-3P	BART3	CGCACCACTAGTCACCAtccGT	139124	139145	22	3
BART3-3P	BART3	CGCACCACTAGTCACCAtcTGT	139124	139145	22	6
BART3-3P	BART3	CGCACCACTAGTCACCAtGgGT	139124	139145	22	5
BART3-3P	BART3	CGCACCACTAGTCACCAtGTGc	139124	139145	22	3
BART3-3P	BART3	CGCACCACTAGTCACCAtGTGT	139124	139145	22	133
BART3-3P	BART3	CGCACCACTAGTCACCAttTGT	139124	139145	22	6
BART3-3P	BART3	CGCACCACTAGTCACCcaGTGT	139124	139145	22	7
BART3-3P	BART3	CGCACCACTAGTCACCcGaTGT	139124	139145	22	19
BART3-3P	BART3	CGCACCACTAGTCACCcGGTGa	139124	139145	22	16
BART3-3P	BART3	CGCACCACTAGTCACCcGGTGc	139124	139145	22	7
BART3-3P	BART3	CGCACCACTAGTCACCcGGTGT	139124	139145	22	193
BART3-3P	BART3	CGCACCACTAGTCACCctGTGT	139124	139145	22	15
BART3-3P	BART3	CGCACCACTAGTCACCgaGTGc	139124	139145	22	3
BART3-3P	BART3	CGCACCACTAGTCACCgaGTGT	139124	139145	22	62
BART3-3P	BART3	CGCACCACTAGTCACCgGaTGT	139124	139145	22	32
BART3-3P	BART3	CGCACCACTAGTCACCgGGTaT	139124	139145	22	11
BART3-3P	BART3	CGCACCACTAGTCACCgGGTGa	139124	139145	22	11
BART3-3P	BART3	CGCACCACTAGTCACCgGGTGc	139124	139145	22	9
BART3-3P	BART3	CGCACCACTAGTCACCgGGTGg	139124	139145	22	3
BART3-3P	BART3	CGCACCACTAGTCACCgGGTGT	139124	139145	22	605
BART3-3P	BART3	CGCACCACTAGTCACCgtGTGc	139124	139145	22	5
BART3-3P	BART3	CGCACCACTAGTCACCgtGTGT	139124	139145	22	98
BART3-3P	BART3	CGCACCACTAGTCACCtaGTGT	139124	139145	22	18
BART3-3P	BART3	CGCACCACTAGTCACCtGGTGc	139124	139145	22	4
BART3-3P	BART3	CGCACCACTAGTCACCtGGTGT	139124	139145	22	14
BART3-3P	BART3	CGCACCACTAGTCACtAGGTGT	139124	139145	22	5
BART3-3P	BART3	CGCACCACTAGTCAttAGGTGT	139124	139145	22	6
BART3-3P	BART3	CGCACCACTAGTCgCCAGGTGT	139124	139145	22	4
BART3-3P	BART3	CGCACCACTAtTCACCAGGTGT	139124	139145	22	3
BART3-3P	BART3	CGCACCAtTAGTCACCAGGTGT	139124	139145	22	8
BART3-3P	BART3	CGCACCgCTAGTCACCAGGTGT	139124	139145	22	6
BART3-3P	BART3	CGCACtACTAGTCACCAGGTGT	139124	139145	22	10
BART3-3P	BART3	CGCAgCACTAGTCACCAGGTGT	139124	139145	22	15
BART3-3P	BART3	CGCgCCACTAGTCACCAGGTGT	139124	139145	22	7
BART3-3P	BART3	CGgACCACTAGTCACCAGGTGT	139124	139145	22	3
BART3-3P	BART3	CGtACCACTAGTCACCAGGTGT	139124	139145	22	3
BART3-3P	BART3	CtaACCACTAGTCACCAGGTGT	139124	139145	22	7
BART3-3P	BART3	CtCACCACTAGTCACCAGGTGT	139124	139145	22	5
BART3-3P	BART3	gGCACCACTAGTCACCAGGTGT	139124	139145	22	36
BART3-3P	BART3	tGCACCACTAGTCACCAGGTGT	139124	139145	22	34
BART3-3P	BART3	aGCACCACTAGTCACCAGGTGTC	139124	139146	23	4
BART3-3P	BART3	CGCACCACTAGTCACCAaacGTa	139124	139146	23	3
BART3-3P	BART3	CGCACCACTAGTCACCAaaTGcC	139124	139146	23	3
BART3-3P	BART3	CGCACCACTAGTCACCAaaTGTC	139124	139146	23	5
BART3-3P	BART3	CGCACCACTAGTCACCAaGcGTC	139124	139146	23	5
BART3-3P	BART3	CGCACCACTAGTCACCAaGTaTC	139124	139146	23	4
BART3-3P	BART3	CGCACCACTAGTCACCAaGTGaC	139124	139146	23	3
BART3-3P	BART3	CGCACCACTAGTCACCAaGTGcC	139124	139146	23	17
BART3-3P	BART3	CGCACCACTAGTCACCAaGTGgC	139124	139146	23	4

BART3-3P	BART3	CGCACCACTAGTCACCAaGTGTa	139124	139146	23	24
BART3-3P	BART3	CGCACCACTAGTCACCAaGTGTC	139124	139146	23	92
BART3-3P	BART3	CGCACCACTAGTCACCAaGTGTg	139124	139146	23	13
BART3-3P	BART3	CGCACCACTAGTCACCAaGTGTt	139124	139146	23	32
BART3-3P	BART3	CGCACCACTAGTCACCAcGTGTC	139124	139146	23	6
BART3-3P	BART3	CGCACCACTAGTCACCAcGTGTg	139124	139146	23	9
BART3-3P	BART3	CGCACCACTAGTCACCAcGTGTt	139124	139146	23	19
BART3-3P	BART3	CGCACCACTAGTCACCActaGTC	139124	139146	23	5
BART3-3P	BART3	CGCACCACTAGTCACCAGaaaTC	139124	139146	23	3
BART3-3P	BART3	CGCACCACTAGTCACCAGaaGTa	139124	139146	23	4
BART3-3P	BART3	CGCACCACTAGTCACCAGacGTa	139124	139146	23	5
BART3-3P	BART3	CGCACCACTAGTCACCAGacGTg	139124	139146	23	4
BART3-3P	BART3	CGCACCACTAGTCACCAGagaTC	139124	139146	23	4
BART3-3P	BART3	CGCACCACTAGTCACCAGagGTC	139124	139146	23	6
BART3-3P	BART3	CGCACCACTAGTCACCAGaTacC	139124	139146	23	4
BART3-3P	BART3	CGCACCACTAGTCACCAGaTaTC	139124	139146	23	7
BART3-3P	BART3	CGCACCACTAGTCACCAGaTcTa	139124	139146	23	3
BART3-3P	BART3	CGCACCACTAGTCACCAGaTGaC	139124	139146	23	3
BART3-3P	BART3	CGCACCACTAGTCACCAGaTGca	139124	139146	23	9
BART3-3P	BART3	CGCACCACTAGTCACCAGaTGcC	139124	139146	23	4
BART3-3P	BART3	CGCACCACTAGTCACCAGaTGcg	139124	139146	23	3
BART3-3P	BART3	CGCACCACTAGTCACCAGaTGTa	139124	139146	23	7
BART3-3P	BART3	CGCACCACTAGTCACCAGaTGTC	139124	139146	23	43
BART3-3P	BART3	CGCACCACTAGTCACCAGaTGTg	139124	139146	23	25
BART3-3P	BART3	CGCACCACTAGTCACCAGaTGTt	139124	139146	23	48
BART3-3P	BART3	CGCACCACTAGTCACCAGcaGTC	139124	139146	23	18
BART3-3P	BART3	CGCACCACTAGTCACCAGcTaTC	139124	139146	23	4
BART3-3P	BART3	CGCACCACTAGTCACCAGcTGTa	139124	139146	23	3
BART3-3P	BART3	CGCACCACTAGTCACCAGcTGTC	139124	139146	23	3
BART3-3P	BART3	CGCACCACTAGTCACCAGGaaTa	139124	139146	23	3
BART3-3P	BART3	CGCACCACTAGTCACCAGGaaTC	139124	139146	23	4
BART3-3P	BART3	CGCACCACTAGTCACCAGGacaC	139124	139146	23	6
BART3-3P	BART3	CGCACCACTAGTCACCAGGacgC	139124	139146	23	5
BART3-3P	BART3	CGCACCACTAGTCACCAGGaGaC	139124	139146	23	5
BART3-3P	BART3	CGCACCACTAGTCACCAGGaGcC	139124	139146	23	3
BART3-3P	BART3	CGCACCACTAGTCACCAGGaGTa	139124	139146	23	17
BART3-3P	BART3	CGCACCACTAGTCACCAGGaGTC	139124	139146	23	29
BART3-3P	BART3	CGCACCACTAGTCACCAGGaGTg	139124	139146	23	20
BART3-3P	BART3	CGCACCACTAGTCACCAGGaGTt	139124	139146	23	10
BART3-3P	BART3	CGCACCACTAGTCACCAGGatgC	139124	139146	23	16
BART3-3P	BART3	CGCACCACTAGTCACCAGGcaTt	139124	139146	23	9
BART3-3P	BART3	CGCACCACTAGTCACCAGGcGTC	139124	139146	23	3
BART3-3P	BART3	CGCACCACTAGTCACCAGGcGTg	139124	139146	23	7
BART3-3P	BART3	CGCACCACTAGTCACCAGGcGTt	139124	139146	23	6
BART3-3P	BART3	CGCACCACTAGTCACCAGGgGTC	139124	139146	23	17
BART3-3P	BART3	CGCACCACTAGTCACCAGGgGTt	139124	139146	23	8
BART3-3P	BART3	CGCACCACTAGTCACCAGGgtaC	139124	139146	23	3
BART3-3P	BART3	CGCACCACTAGTCACCAGGgtgC	139124	139146	23	5
BART3-3P	BART3	CGCACCACTAGTCACCAGGgtTa	139124	139146	23	4
BART3-3P	BART3	CGCACCACTAGTCACCAGGTaaC	139124	139146	23	17
BART3-3P	BART3	CGCACCACTAGTCACCAGGTaca	139124	139146	23	5
BART3-3P	BART3	CGCACCACTAGTCACCAGGTacC	139124	139146	23	4
BART3-3P	BART3	CGCACCACTAGTCACCAGGTagC	139124	139146	23	3
BART3-3P	BART3	CGCACCACTAGTCACCAGGTaTa	139124	139146	23	44
BART3-3P	BART3	CGCACCACTAGTCACCAGGTaTC	139124	139146	23	40
BART3-3P	BART3	CGCACCACTAGTCACCAGGTaTg	139124	139146	23	7
BART3-3P	BART3	CGCACCACTAGTCACCAGGTaTt	139124	139146	23	15
BART3-3P	BART3	CGCACCACTAGTCACCAGGTcaC	139124	139146	23	12
BART3-3P	BART3	CGCACCACTAGTCACCAGGTccC	139124	139146	23	5
BART3-3P	BART3	CGCACCACTAGTCACCAGGTcTC	139124	139146	23	12
BART3-3P	BART3	CGCACCACTAGTCACCAGGTcTg	139124	139146	23	9
BART3-3P	BART3	CGCACCACTAGTCACCAGGTGaC	139124	139146	23	114
BART3-3P	BART3	CGCACCACTAGTCACCAGGTGag	139124	139146	23	7
BART3-3P	BART3	CGCACCACTAGTCACCAGGTGca	139124	139146	23	19
BART3-3P	BART3	CGCACCACTAGTCACCAGGTGcC	139124	139146	23	63
BART3-3P	BART3	CGCACCACTAGTCACCAGGTGcg	139124	139146	23	5
BART3-3P	BART3	CGCACCACTAGTCACCAGGTGct	139124	139146	23	6
BART3-3P	BART3	CGCACCACTAGTCACCAGGTGga	139124	139146	23	7

BART3-3P	BART3	CGCACCACTAGTCACCAGGTGgC	139124	139146	23	16
BART3-3P	BART3	CGCACCACTAGTCACCAGGTGTa	139124	139146	23	142
BART3-3P	BART3	CGCACCACTAGTCACCAGGTGTC	139124	139146	23	630
BART3-3P	BART3	CGCACCACTAGTCACCAGGTGTg	139124	139146	23	372
BART3-3P	BART3	CGCACCACTAGTCACCAGGTGTt	139124	139146	23	333
BART3-3P	BART3	CGCACCACTAGTCACCAGGTtaC	139124	139146	23	3
BART3-3P	BART3	CGCACCACTAGTCACCAGGTtgC	139124	139146	23	9
BART3-3P	BART3	CGCACCACTAGTCACCAGGTtTa	139124	139146	23	4
BART3-3P	BART3	CGCACCACTAGTCACCAGGTtTC	139124	139146	23	7
BART3-3P	BART3	CGCACCACTAGTCACCAGtTGaC	139124	139146	23	13
BART3-3P	BART3	CGCACCACTAGTCACCAGtTGTt	139124	139146	23	4
BART3-3P	BART3	CGCACCACTAGTCACCcttTGTC	139124	139146	23	3
BART3-3P	BART3	CtgACCACTAGTCACCAGGTGTt	139124	139146	23	7
BART3-3P	BART3	gGCACCACTAGTCACCAGGTGTt	139124	139146	23	3
BART3-3P	BART3	taCACCACTAGTCACCAaaTGTa	139124	139146	23	4
BART3-3P	BART3	aGCACCACTAGTCACCAGGTGTCc	139124	139147	24	4
BART3-3P	BART3	CGacCCACTAGTCACCAGGTGTaA	139124	139147	24	5
BART3-3P	BART3	CGCACCACcAGTCACCAGGTGTtt	139124	139147	24	4
BART3-3P	BART3	CGCACCACgcGTCACCAGGTGTCt	139124	139147	24	3
BART3-3P	BART3	CGCACCACTAGTCACCAGacaTCA	139124	139147	24	3
BART3-3P	BART3	CGCACCACTAGTCACCAGacGcCt	139124	139147	24	5
BART3-3P	BART3	CGCACCACTAGTCACCAGacGTCA	139124	139147	24	3
BART3-3P	BART3	CGCACCACTAGTCACCAGaTaTCA	139124	139147	24	3
BART3-3P	BART3	CGCACCACTAGTCACCAGaTGcac	139124	139147	24	6
BART3-3P	BART3	CGCACCACTAGTCACCAGaTGcCA	139124	139147	24	5
BART3-3P	BART3	CGCACCACTAGTCACCAGaTGTaA	139124	139147	24	23
BART3-3P	BART3	CGCACCACTAGTCACCAGaTGTag	139124	139147	24	17
BART3-3P	BART3	CGCACCACTAGTCACCAGaTGTat	139124	139147	24	4
BART3-3P	BART3	CGCACCACTAGTCACCAGaTGTCA	139124	139147	24	138
BART3-3P	BART3	CGCACCACTAGTCACCAGaTGTCc	139124	139147	24	5
BART3-3P	BART3	CGCACCACTAGTCACCAGaTGTCt	139124	139147	24	20
BART3-3P	BART3	CGCACCACTAGTCACCAGaTGTgA	139124	139147	24	4
BART3-3P	BART3	CGCACCACTAGTCACCAGaTGTtA	139124	139147	24	8
BART3-3P	BART3	CGCACCACTAGTCACCAGaTGTtc	139124	139147	24	6
BART3-3P	BART3	CGCACCACTAGTCACCAGcTaTac	139124	139147	24	30
BART3-3P	BART3	CGCACCACTAGTCACCAGcTGTCA	139124	139147	24	5
BART3-3P	BART3	CGCACCACTAGTCACCAGcTGTCt	139124	139147	24	3
BART3-3P	BART3	CGCACCACTAGTCACCAGGaacCA	139124	139147	24	4
BART3-3P	BART3	CGCACCACTAGTCACCAGGaaTaA	139124	139147	24	11
BART3-3P	BART3	CGCACCACTAGTCACCAGGaaTCA	139124	139147	24	11
BART3-3P	BART3	CGCACCACTAGTCACCAGGaaTCt	139124	139147	24	8
BART3-3P	BART3	CGCACCACTAGTCACCAGGaGatA	139124	139147	24	6
BART3-3P	BART3	CGCACCACTAGTCACCAGGaGgCA	139124	139147	24	6
BART3-3P	BART3	CGCACCACTAGTCACCAGGaGTaA	139124	139147	24	16
BART3-3P	BART3	CGCACCACTAGTCACCAGGaGTat	139124	139147	24	7
BART3-3P	BART3	CGCACCACTAGTCACCAGGaGTCA	139124	139147	24	46
BART3-3P	BART3	CGCACCACTAGTCACCAGGcacCc	139124	139147	24	3
BART3-3P	BART3	CGCACCACTAGTCACCAGGcaTag	139124	139147	24	29
BART3-3P	BART3	CGCACCACTAGTCACCAGGcaTCA	139124	139147	24	3
BART3-3P	BART3	CGCACCACTAGTCACCAGGccTac	139124	139147	24	3
BART3-3P	BART3	CGCACCACTAGTCACCAGGcGTaA	139124	139147	24	15
BART3-3P	BART3	CGCACCACTAGTCACCAGGcGTat	139124	139147	24	3
BART3-3P	BART3	CGCACCACTAGTCACCAGGcGTCA	139124	139147	24	3
BART3-3P	BART3	CGCACCACTAGTCACCAGGgGTaA	139124	139147	24	8
BART3-3P	BART3	CGCACCACTAGTCACCAGGgGTag	139124	139147	24	6
BART3-3P	BART3	CGCACCACTAGTCACCAGGgGTat	139124	139147	24	6
BART3-3P	BART3	CGCACCACTAGTCACCAGGgGTCA	139124	139147	24	33
BART3-3P	BART3	CGCACCACTAGTCACCAGGgGTCc	139124	139147	24	3
BART3-3P	BART3	CGCACCACTAGTCACCAGGTaagA	139124	139147	24	4
BART3-3P	BART3	CGCACCACTAGTCACCAGGTacCA	139124	139147	24	11
BART3-3P	BART3	CGCACCACTAGTCACCAGGTacCg	139124	139147	24	10
BART3-3P	BART3	CGCACCACTAGTCACCAGGTacCt	139124	139147	24	4
BART3-3P	BART3	CGCACCACTAGTCACCAGGTagaA	139124	139147	24	14
BART3-3P	BART3	CGCACCACTAGTCACCAGGTaTaA	139124	139147	24	12
BART3-3P	BART3	CGCACCACTAGTCACCAGGTaTat	139124	139147	24	3
BART3-3P	BART3	CGCACCACTAGTCACCAGGTaTCA	139124	139147	24	23
BART3-3P	BART3	CGCACCACTAGTCACCAGGTaTCt	139124	139147	24	47
BART3-3P	BART3	CGCACCACTAGTCACCAGGTaTgt	139124	139147	24	9

BART3-3P	BART3	CGCACCACTAGTCACCAGGTaTtA	139124	139147	24	9
BART3-3P	BART3	CGCACCACTAGTCACCAGGTaTtc	139124	139147	24	7
BART3-3P	BART3	CGCACCACTAGTCACCAGGTcagA	139124	139147	24	7
BART3-3P	BART3	CGCACCACTAGTCACCAGGTcTCA	139124	139147	24	3
BART3-3P	BART3	CGCACCACTAGTCACCAGGTcTCt	139124	139147	24	5
BART3-3P	BART3	CGCACCACTAGTCACCAGGTGaaA	139124	139147	24	31
BART3-3P	BART3	CGCACCACTAGTCACCAGGTGaat	139124	139147	24	12
BART3-3P	BART3	CGCACCACTAGTCACCAGGTGaCA	139124	139147	24	10
BART3-3P	BART3	CGCACCACTAGTCACCAGGTGagA	139124	139147	24	4
BART3-3P	BART3	CGCACCACTAGTCACCAGGTGagt	139124	139147	24	4
BART3-3P	BART3	CGCACCACTAGTCACCAGGTGatA	139124	139147	24	3
BART3-3P	BART3	CGCACCACTAGTCACCAGGTGatc	139124	139147	24	9
BART3-3P	BART3	CGCACCACTAGTCACCAGGTGcaA	139124	139147	24	40
BART3-3P	BART3	CGCACCACTAGTCACCAGGTGcac	139124	139147	24	3
BART3-3P	BART3	CGCACCACTAGTCACCAGGTGcCA	139124	139147	24	42
BART3-3P	BART3	CGCACCACTAGTCACCAGGTGcCc	139124	139147	24	3
BART3-3P	BART3	CGCACCACTAGTCACCAGGTGcCg	139124	139147	24	13
BART3-3P	BART3	CGCACCACTAGTCACCAGGTGcCt	139124	139147	24	115
BART3-3P	BART3	CGCACCACTAGTCACCAGGTGcgt	139124	139147	24	3
BART3-3P	BART3	CGCACCACTAGTCACCAGGTGctc	139124	139147	24	6
BART3-3P	BART3	CGCACCACTAGTCACCAGGTGgaA	139124	139147	24	5
BART3-3P	BART3	CGCACCACTAGTCACCAGGTGgag	139124	139147	24	3
BART3-3P	BART3	CGCACCACTAGTCACCAGGTGgat	139124	139147	24	8
BART3-3P	BART3	CGCACCACTAGTCACCAGGTGgCA	139124	139147	24	5
BART3-3P	BART3	CGCACCACTAGTCACCAGGTGggt	139124	139147	24	7
BART3-3P	BART3	CGCACCACTAGTCACCAGGTGgtc	139124	139147	24	5
BART3-3P	BART3	CGCACCACTAGTCACCAGGTGTaA	139124	139147	24	353
BART3-3P	BART3	CGCACCACTAGTCACCAGGTGTac	139124	139147	24	20
BART3-3P	BART3	CGCACCACTAGTCACCAGGTGTag	139124	139147	24	56
BART3-3P	BART3	CGCACCACTAGTCACCAGGTGTat	139124	139147	24	121
BART3-3P	BART3	CGCACCACTAGTCACCAGGTGTCA	139124	139147	24	421
BART3-3P	BART3	CGCACCACTAGTCACCAGGTGTCc	139124	139147	24	42
BART3-3P	BART3	CGCACCACTAGTCACCAGGTGTCg	139124	139147	24	25
BART3-3P	BART3	CGCACCACTAGTCACCAGGTGTCt	139124	139147	24	421
BART3-3P	BART3	CGCACCACTAGTCACCAGGTGTgA	139124	139147	24	12
BART3-3P	BART3	CGCACCACTAGTCACCAGGTGTgg	139124	139147	24	4
BART3-3P	BART3	CGCACCACTAGTCACCAGGTGTgt	139124	139147	24	44
BART3-3P	BART3	CGCACCACTAGTCACCAGGTGTtA	139124	139147	24	55
BART3-3P	BART3	CGCACCACTAGTCACCAGGTGTtc	139124	139147	24	8
BART3-3P	BART3	CGCACCACTAGTCACCAGGTGTtg	139124	139147	24	6
BART3-3P	BART3	CGCACCACTAGTCACCAGGTGTtt	139124	139147	24	57
BART3-3P	BART3	CGCACCACTAGTCACCAGGTtcaA	139124	139147	24	4
BART3-3P	BART3	CGCACCACTAGTCACCAGGTtgCt	139124	139147	24	25
BART3-3P	BART3	CGCACCACTAGTCACCAGtaGTCA	139124	139147	24	3
BART3-3P	BART3	CGCACCACTAGTCACCAGtgGTCA	139124	139147	24	3
BART3-3P	BART3	CGCACCACTAGTCACCAGtgGTCt	139124	139147	24	4
BART3-3P	BART3	CGCACCACTAGTCACCAGtTGTCA	139124	139147	24	6
BART3-3P	BART3	CGCACCACTAGTCACCAGtTGTCt	139124	139147	24	6
BART3-3P	BART3	CGCACCACTAGTCACCgaGTGTCA	139124	139147	24	3
BART3-3P	BART3	CGCcCCACTAGTCACCAGGTGgat	139124	139147	24	3
BART3-3P	BART3	gGCACCACTAGTCACCAGGTaTCA	139124	139147	24	3
BART3-3P	BART3	taCACCACTAGTCACCAGGaGTag	139124	139147	24	4
BART3-3P	BART3	tGCACCACTAGTCACCAGGTGTCA	139124	139147	24	3
BART3-3P	BART3	CGCACCACTAGTCACCAGaTGTtca	139124	139148	25	4
BART3-3P	BART3	CGCACCACTAGTCACCAGGaGTCgC	139124	139148	25	4
BART3-3P	BART3	CGCACCACTAGTCACCAGGcGTCAa	139124	139148	25	3
BART3-3P	BART3	CGCACCACTAGTCACCAGGcGTCAC	139124	139148	25	3
BART3-3P	BART3	CGCACCACTAGTCACCAGGgGTCtt	139124	139148	25	3
BART3-3P	BART3	CGCACCACTAGTCACCAGGgGTgca	139124	139148	25	3
BART3-3P	BART3	CGCACCACTAGTCACCAGGTaTact	139124	139148	25	5
BART3-3P	BART3	CGCACCACTAGTCACCAGGTGcCAa	139124	139148	25	5
BART3-3P	BART3	CGCACCACTAGTCACCAGGTGcCAt	139124	139148	25	5
BART3-3P	BART3	CGCACCACTAGTCACCAGGTGcCga	139124	139148	25	3
BART3-3P	BART3	CGCACCACTAGTCACCAGGTGgCAC	139124	139148	25	4
BART3-3P	BART3	CGCACCACTAGTCACCAGGTGTaAa	139124	139148	25	8
BART3-3P	BART3	CGCACCACTAGTCACCAGGTGTaAC	139124	139148	25	19
BART3-3P	BART3	CGCACCACTAGTCACCAGGTGTaAt	139124	139148	25	8
BART3-3P	BART3	CGCACCACTAGTCACCAGGTGTact	139124	139148	25	8

BART3-3P	BART3	CGCACCACTAGTCACCAGGTGTaga	139124	139148	25	8
BART3-3P	BART3	CGCACCACTAGTCACCAGGTGTata	139124	139148	25	13
BART3-3P	BART3	CGCACCACTAGTCACCAGGTGTCAa	139124	139148	25	11
BART3-3P	BART3	CGCACCACTAGTCACCAGGTGTCAC	139124	139148	25	27
BART3-3P	BART3	CGCACCACTAGTCACCAGGTGTCAg	139124	139148	25	4
BART3-3P	BART3	CGCACCACTAGTCACCAGGTGTCAt	139124	139148	25	17
BART3-3P	BART3	CGCACCACTAGTCACCAGGTGTCca	139124	139148	25	7
BART3-3P	BART3	CGCACCACTAGTCACCAGGTGTCcC	139124	139148	25	4
BART3-3P	BART3	CGCACCACTAGTCACCAGGTGTCct	139124	139148	25	15
BART3-3P	BART3	CGCACCACTAGTCACCAGGTGTCta	139124	139148	25	18
BART3-3P	BART3	CGCACCACTAGTCACCAGGTGTCtC	139124	139148	25	4
BART3-3P	BART3	CGCACCACTAGTCACCAGGTGTCtg	139124	139148	25	3
BART3-3P	BART3	CGCACCACTAGTCACCAGGTGTCtt	139124	139148	25	3
BART3-3P	BART3	CGCACCACTAGTCACCAGGTGTgAC	139124	139148	25	4
BART3-3P	BART3	CGCACCACTAGTCACCAGGTGTgct	139124	139148	25	4
BART3-3P	BART3	CGCACCACTAGTCACCAGGTGTgtt	139124	139148	25	3
BART3-3P	BART3	CGCACCACTAGTCACCAGGTGTtga	139124	139148	25	3
BART3-3P	BART3	CGCACCACTAGTCACCAGGTGTttC	139124	139148	25	6
BART3-3P	BART3	CGCACCACTAGTCACCAGGTGTCACC	139124	139149	26	12
BART3-3P	BART3	CGCACCACTAGTCACCAGGTGTCACg	139124	139149	26	5
BART3-3P	BART3	CGCACCACTAGTCACCAGGTGTCACt	139124	139149	26	4
BART3-3P	BART3	CGCACCACTAGTCACCAGGTGTCAta	139124	139149	26	4
BART3-3P	BART3	CGCACCACTAGTCACCAGGTGTCgCC	139124	139149	26	3
BART3-3P	BART3	CGCACCACTAGTCACCAGGTGTCtCCG	139124	139150	27	3
BART3-3P	BART3	CGCACCACTAGTCACCAGGTGTCACCcc	139124	139151	28	9
BART3-3P	BART3	CGCACCACTAGTCACCAGGTGTCACCGG	139124	139151	28	36
BART3-3P	BART3	CGCACCACTAGTCACCAGGTGTCgttGG	139124	139151	28	7
BART3-3P	BART3	CGCACCACTAGTCACCtGGTGTCACCGG	139124	139151	28	3
BART3-3P	BART3	atACCACTAGTCACCAGGTGT	139125	139145	21	5
BART3-3P	BART3	caACCACTAGTCACCAGGTGT	139125	139145	21	7
BART3-3P	BART3	GCACCACTAGTCACCAGGTGT	139125	139145	21	4
BART3-3P	BART3	GCACCACTAGTCACCAGGTGTC	139125	139146	22	5
BART3-3P	BART3	GCACCACTAGTCACCAGGcagCt	139125	139147	23	6
BART3-3P	BART3	GCACCACTAGTCACCAGGTGTCt	139125	139147	23	5
BART4-5P	BART4	GACCTGATGCTGCaGGTGT	139228	139246	19	13
BART4-5P	BART4	GACCTGATGCTGCcaGTGT	139228	139246	19	10
BART4-5P	BART4	GACCTGATGCTGCcGGTGT	139228	139246	19	46
BART4-5P	BART4	GACCTGATGCTGCgGGTGT	139228	139246	19	8
BART4-5P	BART4	GACCTGATGCTGCTaaTGT	139228	139246	19	5
BART4-5P	BART4	GACCTGATGCTGCTaGTGT	139228	139246	19	24
BART4-5P	BART4	GACCTGATGCTGCTcGTGT	139228	139246	19	4
BART4-5P	BART4	GACCTGATGCTGCTGaTGT	139228	139246	19	33
BART4-5P	BART4	GACCTGATGCTGCTGGcaT	139228	139246	19	15
BART4-5P	BART4	GACCTGATGCTGCTGGgGT	139228	139246	19	4
BART4-5P	BART4	GACCTGATGCTGCTGGTaT	139228	139246	19	3
BART4-5P	BART4	GACCTGATGCTGCTGGTGc	139228	139246	19	6
BART4-5P	BART4	GACCTGATGCTGCTGGTGT	139228	139246	19	204
BART4-5P	BART4	GACCTGATGCTGCTtGTGT	139228	139246	19	20
BART4-5P	BART4	GgCCTGATGCTGCTGGTGT	139228	139246	19	5
BART4-5P	BART4	GACCTGATGCTGCTaGTGac	139228	139247	20	4
BART4-5P	BART4	GACCTGATGCTGCTaGTGTa	139228	139247	20	21
BART4-5P	BART4	GACCTGATGCTGCTaGTGTc	139228	139247	20	3
BART4-5P	BART4	GACCTGATGCTGCTaGTGTG	139228	139247	20	61
BART4-5P	BART4	GACCTGATGCTGCTcGTGTG	139228	139247	20	15
BART4-5P	BART4	GACCTGATGCTGCTGaaGTG	139228	139247	20	15
BART4-5P	BART4	GACCTGATGCTGCTGaTact	139228	139247	20	3
BART4-5P	BART4	GACCTGATGCTGCTGaTGca	139228	139247	20	8
BART4-5P	BART4	GACCTGATGCTGCTGaTGcc	139228	139247	20	3
BART4-5P	BART4	GACCTGATGCTGCTGaTGct	139228	139247	20	5
BART4-5P	BART4	GACCTGATGCTGCTGaTGTG	139228	139247	20	12
BART4-5P	BART4	GACCTGATGCTGCTGGaGTG	139228	139247	20	11
BART4-5P	BART4	GACCTGATGCTGCTGGcaTG	139228	139247	20	5
BART4-5P	BART4	GACCTGATGCTGCTGGcGTa	139228	139247	20	7
BART4-5P	BART4	GACCTGATGCTGCTGGcGTG	139228	139247	20	3
BART4-5P	BART4	GACCTGATGCTGCTGGgGTG	139228	139247	20	3
BART4-5P	BART4	GACCTGATGCTGCTGGTact	139228	139247	20	32
BART4-5P	BART4	GACCTGATGCTGCTGGTaTa	139228	139247	20	4
BART4-5P	BART4	GACCTGATGCTGCTGGTaTG	139228	139247	20	6

BART4-5P	BART4	GACCTGATGCTGCTGGTcaG	139228	139247	20	7
BART4-5P	BART4	GACCTGATGCTGCTGGTGaG	139228	139247	20	25
BART4-5P	BART4	GACCTGATGCTGCTGGTGca	139228	139247	20	14
BART4-5P	BART4	GACCTGATGCTGCTGGTGct	139228	139247	20	19
BART4-5P	BART4	GACCTGATGCTGCTGGTGTa	139228	139247	20	25
BART4-5P	BART4	GACCTGATGCTGCTGGTGTc	139228	139247	20	21
BART4-5P	BART4	GACCTGATGCTGCTGGTGTG	139228	139247	20	267
BART4-5P	BART4	GACCTGATGCTGCTGGTGTt	139228	139247	20	4
BART4-5P	BART4	GACCTGATGCTGCTGGTtca	139228	139247	20	8
BART4-5P	BART4	GACCTGATGCTGCTGGTtgc	139228	139247	20	22
BART4-5P	BART4	aACCTGATGCTGCTGGTGTGC	139228	139248	21	4
BART4-5P	BART4	GACCgtcTGCTGCTGGTGTGC	139228	139248	21	3
BART4-5P	BART4	GACCTGATGCTGCTGaaGTGC	139228	139248	21	6
BART4-5P	BART4	GACCTGATGCTGCTGacaTGC	139228	139248	21	7
BART4-5P	BART4	GACCTGATGCTGCTGacGTGa	139228	139248	21	4
BART4-5P	BART4	GACCTGATGCTGCTGacGTGC	139228	139248	21	10
BART4-5P	BART4	GACCTGATGCTGCTGagGTGa	139228	139248	21	6
BART4-5P	BART4	GACCTGATGCTGCTGagGTGC	139228	139248	21	48
BART4-5P	BART4	GACCTGATGCTGCTGaTGaGC	139228	139248	21	14
BART4-5P	BART4	GACCTGATGCTGCTGaTGcGC	139228	139248	21	14
BART4-5P	BART4	GACCTGATGCTGCTGaTGTGa	139228	139248	21	18
BART4-5P	BART4	GACCTGATGCTGCTGaTGTGC	139228	139248	21	298
BART4-5P	BART4	GACCTGATGCTGCTGaTGTGt	139228	139248	21	19
BART4-5P	BART4	GACCTGATGCTGCTGccGTGC	139228	139248	21	8
BART4-5P	BART4	GACCTGATGCTGCTGcTGTGC	139228	139248	21	8
BART4-5P	BART4	GACCTGATGCTGCTGGaaTct	139228	139248	21	5
BART4-5P	BART4	GACCTGATGCTGCTGGaaTGC	139228	139248	21	18
BART4-5P	BART4	GACCTGATGCTGCTGGaGTGa	139228	139248	21	39
BART4-5P	BART4	GACCTGATGCTGCTGGaGTGC	139228	139248	21	42
BART4-5P	BART4	GACCTGATGCTGCTGGaGTGt	139228	139248	21	6
BART4-5P	BART4	GACCTGATGCTGCTGGcaTct	139228	139248	21	3
BART4-5P	BART4	GACCTGATGCTGCTGGcaTGC	139228	139248	21	13
BART4-5P	BART4	GACCTGATGCTGCTGGcGTGa	139228	139248	21	11
BART4-5P	BART4	GACCTGATGCTGCTGGcGTGC	139228	139248	21	15
BART4-5P	BART4	GACCTGATGCTGCTGGcGTGt	139228	139248	21	3
BART4-5P	BART4	GACCTGATGCTGCTGGgaTGa	139228	139248	21	3
BART4-5P	BART4	GACCTGATGCTGCTGGgaTGC	139228	139248	21	17
BART4-5P	BART4	GACCTGATGCTGCTGGgGTaa	139228	139248	21	3
BART4-5P	BART4	GACCTGATGCTGCTGGgGTGa	139228	139248	21	61
BART4-5P	BART4	GACCTGATGCTGCTGGgGTGC	139228	139248	21	180
BART4-5P	BART4	GACCTGATGCTGCTGGgGTGt	139228	139248	21	15
BART4-5P	BART4	GACCTGATGCTGCTGGTacGC	139228	139248	21	10
BART4-5P	BART4	GACCTGATGCTGCTGGTaTct	139228	139248	21	10
BART4-5P	BART4	GACCTGATGCTGCTGGTaTGa	139228	139248	21	20
BART4-5P	BART4	GACCTGATGCTGCTGGTaTGC	139228	139248	21	119
BART4-5P	BART4	GACCTGATGCTGCTGGTaTGt	139228	139248	21	12
BART4-5P	BART4	GACCTGATGCTGCTGGTccaC	139228	139248	21	4
BART4-5P	BART4	GACCTGATGCTGCTGGTcTGC	139228	139248	21	4
BART4-5P	BART4	GACCTGATGCTGCTGGTGaGa	139228	139248	21	9
BART4-5P	BART4	GACCTGATGCTGCTGGTGaGC	139228	139248	21	105
BART4-5P	BART4	GACCTGATGCTGCTGGTGaGt	139228	139248	21	8
BART4-5P	BART4	GACCTGATGCTGCTGGTGatC	139228	139248	21	17
BART4-5P	BART4	GACCTGATGCTGCTGGTGcaC	139228	139248	21	4
BART4-5P	BART4	GACCTGATGCTGCTGGTGcGa	139228	139248	21	4
BART4-5P	BART4	GACCTGATGCTGCTGGTGcGC	139228	139248	21	64
BART4-5P	BART4	GACCTGATGCTGCTGGTGcGt	139228	139248	21	3
BART4-5P	BART4	GACCTGATGCTGCTGGTGgat	139228	139248	21	4
BART4-5P	BART4	GACCTGATGCTGCTGGTGgtC	139228	139248	21	13
BART4-5P	BART4	GACCTGATGCTGCTGGTGTaa	139228	139248	21	17
BART4-5P	BART4	GACCTGATGCTGCTGGTGTaC	139228	139248	21	23
BART4-5P	BART4	GACCTGATGCTGCTGGTGTag	139228	139248	21	5
BART4-5P	BART4	GACCTGATGCTGCTGGTGTat	139228	139248	21	16
BART4-5P	BART4	GACCTGATGCTGCTGGTGTcC	139228	139248	21	3
BART4-5P	BART4	GACCTGATGCTGCTGGTGTct	139228	139248	21	55
BART4-5P	BART4	GACCTGATGCTGCTGGTGTGa	139228	139248	21	101
BART4-5P	BART4	GACCTGATGCTGCTGGTGTGC	139228	139248	21	1159
BART4-5P	BART4	GACCTGATGCTGCTGGTGTGg	139228	139248	21	37
BART4-5P	BART4	GACCTGATGCTGCTGGTGTGt	139228	139248	21	118

BART4-5P	BART4	GACCTGATGCTGCTGGTtcGC	139228	139248	21	4
BART4-5P	BART4	GACCTGATGCTGCTGtTGTGC	139228	139248	21	16
BART4-5P	BART4	GACCTGATGCTGCTtGTGTGa	139228	139248	21	5
BART4-5P	BART4	GcaCTGATGCTGCTGGTGTGC	139228	139248	21	4
BART4-5P	BART4	aACCTGATGCTGCTGGTGTaCT	139228	139249	22	8
BART4-5P	BART4	aACCTGATGCTGCTGGTGTGCT	139228	139249	22	14
BART4-5P	BART4	cACCTGATGCTGCTGGTGTGCT	139228	139249	22	6
BART4-5P	BART4	cgCCTGATGCTGCTGGTGTGCT	139228	139249	22	11
BART4-5P	BART4	GACCcGATGCTGCTGGTGTGCT	139228	139249	22	6
BART4-5P	BART4	GACCTGATGaTGCTGGTGTGCT	139228	139249	22	3
BART4-5P	BART4	GACCTGATGCTaCTGGTGTGCT	139228	139249	22	6
BART4-5P	BART4	GACCTGATGCTGCTaacGTGCT	139228	139249	22	4
BART4-5P	BART4	GACCTGATGCTGCTGGaaTGCT	139228	139249	22	6
BART4-5P	BART4	GACCTGATGCTGCTGGacTGCT	139228	139249	22	3
BART4-5P	BART4	GACCTGATGCTGCTGGaGTaCT	139228	139249	22	14
BART4-5P	BART4	GACCTGATGCTGCTGGaGTGaT	139228	139249	22	3
BART4-5P	BART4	GACCTGATGCTGCTGGaGTGCT	139228	139249	22	326
BART4-5P	BART4	GACCTGATGCTGCTGGaGTGga	139228	139249	22	5
BART4-5P	BART4	GACCTGATGCTGCTGGcaTaCT	139228	139249	22	3
BART4-5P	BART4	GACCTGATGCTGCTGGcaTGCT	139228	139249	22	17
BART4-5P	BART4	GACCTGATGCTGCTGGccTGCT	139228	139249	22	15
BART4-5P	BART4	GACCTGATGCTGCTGGcGTaCT	139228	139249	22	64
BART4-5P	BART4	GACCTGATGCTGCTGGcGTGCc	139228	139249	22	3
BART4-5P	BART4	GACCTGATGCTGCTGGcGTGCT	139228	139249	22	178
BART4-5P	BART4	GACCTGATGCTGCTGGgaTGCT	139228	139249	22	23
BART4-5P	BART4	GACCTGATGCTGCTGGgcTaCT	139228	139249	22	3
BART4-5P	BART4	GACCTGATGCTGCTGGgGTaCT	139228	139249	22	35
BART4-5P	BART4	GACCTGATGCTGCTGGgGTGaT	139228	139249	22	11
BART4-5P	BART4	GACCTGATGCTGCTGGgGTGCc	139228	139249	22	4
BART4-5P	BART4	GACCTGATGCTGCTGGgGTGCT	139228	139249	22	290
BART4-5P	BART4	GACCTGATGCTGCTGGgGTGta	139228	139249	22	3
BART4-5P	BART4	GACCTGATGCTGCTGGgtTGCT	139228	139249	22	6
BART4-5P	BART4	GACCTGATGCTGCTGGTaaGCT	139228	139249	22	6
BART4-5P	BART4	GACCTGATGCTGCTGGTacaCT	139228	139249	22	7
BART4-5P	BART4	GACCTGATGCTGCTGGTaTaCT	139228	139249	22	20
BART4-5P	BART4	GACCTGATGCTGCTGGTaTGCT	139228	139249	22	185
BART4-5P	BART4	GACCTGATGCTGCTGGTccaCT	139228	139249	22	4
BART4-5P	BART4	GACCTGATGCTGCTGGTcTaCT	139228	139249	22	10
BART4-5P	BART4	GACCTGATGCTGCTGGTcTGCT	139228	139249	22	12
BART4-5P	BART4	GACCTGATGCTGCTGGTGaaCT	139228	139249	22	12
BART4-5P	BART4	GACCTGATGCTGCTGGTGaGCa	139228	139249	22	5
BART4-5P	BART4	GACCTGATGCTGCTGGTGaGCT	139228	139249	22	32
BART4-5P	BART4	GACCTGATGCTGCTGGTGcaCT	139228	139249	22	26
BART4-5P	BART4	GACCTGATGCTGCTGGTGgaCT	139228	139249	22	8
BART4-5P	BART4	GACCTGATGCTGCTGGTGTaaT	139228	139249	22	3
BART4-5P	BART4	GACCTGATGCTGCTGGTGTaCc	139228	139249	22	7
BART4-5P	BART4	GACCTGATGCTGCTGGTGTaCg	139228	139249	22	6
BART4-5P	BART4	GACCTGATGCTGCTGGTGTaCT	139228	139249	22	211
BART4-5P	BART4	GACCTGATGCTGCTGGTGTagT	139228	139249	22	5
BART4-5P	BART4	GACCTGATGCTGCTGGTGTcCT	139228	139249	22	9
BART4-5P	BART4	GACCTGATGCTGCTGGTGTGac	139228	139249	22	8
BART4-5P	BART4	GACCTGATGCTGCTGGTGTGaT	139228	139249	22	12
BART4-5P	BART4	GACCTGATGCTGCTGGTGTGCa	139228	139249	22	12
BART4-5P	BART4	GACCTGATGCTGCTGGTGTGCc	139228	139249	22	31
BART4-5P	BART4	GACCTGATGCTGCTGGTGTGCg	139228	139249	22	6
BART4-5P	BART4	GACCTGATGCTGCTGGTGTGCT	139228	139249	22	1622
BART4-5P	BART4	GACCTGATGCTGCTGGTGTGgT	139228	139249	22	4
BART4-5P	BART4	GACCTGATGCTGCTGGTGTGta	139228	139249	22	5
BART4-5P	BART4	GACCTGATGCTGCTGGTGTGtT	139228	139249	22	3
BART4-5P	BART4	GACCTGATGCTGCTGGTtaGCT	139228	139249	22	3
BART4-5P	BART4	GACCTGATGCTGCTGGTtTGCT	139228	139249	22	6
BART4-5P	BART4	GACCTGATGtTGCTGGTGTGCT	139228	139249	22	3
BART4-5P	BART4	GtCCTGATGCTGCTGGTGTGCT	139228	139249	22	4
BART4-5P	BART4	tACCTGATGCTGCTGGTGTGCT	139228	139249	22	8
BART4-5P	BART4	aACCTGATGCTGCTGGTGTGCTG	139228	139250	23	4
BART4-5P	BART4	GACCTGATGCTGCTGGgGTGCTG	139228	139250	23	4
BART4-5P	BART4	GACCTGATGCTGCTGGTacGCTG	139228	139250	23	7
BART4-5P	BART4	GACCTGATGCTGCTGGTaTGCcG	139228	139250	23	8

BART4-5P	BART4	GACCTGATGCTGCTGGTaTGCTc	139228	139250	23	5
BART4-5P	BART4	GACCTGATGCTGCTGGTaTGCTG	139228	139250	23	60
BART4-5P	BART4	GACCTGATGCTGCTGGTGaaCTG	139228	139250	23	16
BART4-5P	BART4	GACCTGATGCTGCTGGTGacCTc	139228	139250	23	3
BART4-5P	BART4	GACCTGATGCTGCTGGTGaGCTc	139228	139250	23	7
BART4-5P	BART4	GACCTGATGCTGCTGGTGaGCTG	139228	139250	23	34
BART4-5P	BART4	GACCTGATGCTGCTGGTGcaCTc	139228	139250	23	3
BART4-5P	BART4	GACCTGATGCTGCTGGTGcaCTG	139228	139250	23	10
BART4-5P	BART4	GACCTGATGCTGCTGGTGcGCTG	139228	139250	23	13
BART4-5P	BART4	GACCTGATGCTGCTGGTGctCTc	139228	139250	23	5
BART4-5P	BART4	GACCTGATGCTGCTGGTGgGCTG	139228	139250	23	5
BART4-5P	BART4	GACCTGATGCTGCTGGTGTaCgG	139228	139250	23	3
BART4-5P	BART4	GACCTGATGCTGCTGGTGTaCTc	139228	139250	23	7
BART4-5P	BART4	GACCTGATGCTGCTGGTGTaCTG	139228	139250	23	63
BART4-5P	BART4	GACCTGATGCTGCTGGTGTaCTt	139228	139250	23	3
BART4-5P	BART4	GACCTGATGCTGCTGGTGTcCcG	139228	139250	23	3
BART4-5P	BART4	GACCTGATGCTGCTGGTGTcCTG	139228	139250	23	5
BART4-5P	BART4	GACCTGATGCTGCTGGTGTGaTc	139228	139250	23	3
BART4-5P	BART4	GACCTGATGCTGCTGGTGTGaTG	139228	139250	23	5
BART4-5P	BART4	GACCTGATGCTGCTGGTGTGCac	139228	139250	23	5
BART4-5P	BART4	GACCTGATGCTGCTGGTGTGCaG	139228	139250	23	91
BART4-5P	BART4	GACCTGATGCTGCTGGTGTGCca	139228	139250	23	6
BART4-5P	BART4	GACCTGATGCTGCTGGTGTGCcG	139228	139250	23	16
BART4-5P	BART4	GACCTGATGCTGCTGGTGTGCgG	139228	139250	23	13
BART4-5P	BART4	GACCTGATGCTGCTGGTGTGCTa	139228	139250	23	4
BART4-5P	BART4	GACCTGATGCTGCTGGTGTGCTc	139228	139250	23	201
BART4-5P	BART4	GACCTGATGCTGCTGGTGTGCTG	139228	139250	23	829
BART4-5P	BART4	GACCTGATGCTGCTGGTGTGCTt	139228	139250	23	29
BART4-5P	BART4	GACCTGATGCTGCTGGTGTGgTG	139228	139250	23	4
BART4-5P	BART4	tACCTGATGCTGCTGGTGTGCTG	139228	139250	23	7
BART4-5P	BART4	GACCTGATGCTGCTGGTGaGCTaT	139228	139251	24	43
BART4-5P	BART4	GACCTGATGCTGCTGGTGaGCTcT	139228	139251	24	3
BART4-5P	BART4	GACCTGATGCTGCTGGTGaGCTGg	139228	139251	24	9
BART4-5P	BART4	GACCTGATGCTGCTGGTGaGCTtT	139228	139251	24	4
BART4-5P	BART4	GACCTGATGCTGCTGGTGcaCTaT	139228	139251	24	3
BART4-5P	BART4	GACCTGATGCTGCTGGTGcaCTGg	139228	139251	24	7
BART4-5P	BART4	GACCTGATGCTGCTGGTGcGCTaT	139228	139251	24	16
BART4-5P	BART4	GACCTGATGCTGCTGGTGcGCTcT	139228	139251	24	3
BART4-5P	BART4	GACCTGATGCTGCTGGTGcGCTGg	139228	139251	24	10
BART4-5P	BART4	GACCTGATGCTGCTGGTGgGCTaT	139228	139251	24	13
BART4-5P	BART4	GACCTGATGCTGCTGGTGTaCTaT	139228	139251	24	21
BART4-5P	BART4	GACCTGATGCTGCTGGTGTaCTGg	139228	139251	24	4
BART4-5P	BART4	GACCTGATGCTGCTGGTGTaCTGT	139228	139251	24	3
BART4-5P	BART4	GACCTGATGCTGCTGGTGTGaTGT	139228	139251	24	3
BART4-5P	BART4	GACCTGATGCTGCTGGTGTGCcaT	139228	139251	24	3
BART4-5P	BART4	GACCTGATGCTGCTGGTGTGCcGa	139228	139251	24	4
BART4-5P	BART4	GACCTGATGCTGCTGGTGTGCTaT	139228	139251	24	57
BART4-5P	BART4	GACCTGATGCTGCTGGTGTGCTGa	139228	139251	24	5
BART4-5P	BART4	GACCTGATGCTGCTGGTGTGCTGg	139228	139251	24	20
BART4-5P	BART4	GACCTGATGCTGCTGGTGTGCTGT	139228	139251	24	14
BART4-5P	BART4	GACCTGATGCTGCTGGTGTGCTta	139228	139251	24	5
BART4-5P	BART4	GACCTGATGCTGCTGGTGTGCTtT	139228	139251	24	20
BART4-5P	BART4	GACCTGATGCTGCTGGTGTGCacaA	139228	139252	25	3
BART4-5P	BART4	GACCTGATGCTGCTGGTGTGCacTg	139228	139252	25	22
BART4-5P	BART4	GACCTGATGCTGCTGGTGTGCctTA	139228	139252	25	4
BART4-5P	BART4	GACCTGATGCTGCTGGTGTGCTaaA	139228	139252	25	21
BART4-5P	BART4	GACCTGATGCTGCTGGTGTGCTacA	139228	139252	25	3
BART4-5P	BART4	GACCTGATGCTGCTGGTGTGCTagA	139228	139252	25	11
BART4-5P	BART4	GACCTGATGCTGCTGGTGTGCTaTA	139228	139252	25	9
BART4-5P	BART4	GACCTGATGCTGCTGGTGTGCTcaA	139228	139252	25	6
BART4-5P	BART4	GACCTGATGCTGCTGGTGTGCTcTA	139228	139252	25	3
BART4-5P	BART4	GACCTGATGCTGCTGGTGTGCTGag	139228	139252	25	3
BART4-5P	BART4	GACCTGATGCTGCTGGTGTGCTGcc	139228	139252	25	3
BART4-5P	BART4	GACCTGATGCTGCTGGTGTGCTGcg	139228	139252	25	7
BART4-5P	BART4	GACCTGATGCTGCTGGTGTGCTGgc	139228	139252	25	13
BART4-5P	BART4	GACCTGATGCTGCTGGTGTGCTGgg	139228	139252	25	12
BART4-5P	BART4	GACCTGATGCTGCTGGTGTGCTGTA	139228	139252	25	13
BART4-5P	BART4	GACCTGATGCTGCTGGTGTGCTGTt	139228	139252	25	3

BART4-5P	BART4	GACCTGATGCTGCTGGTGTGCTaagA	139228	139253	26	5
BART4-5P	BART4	GACCTGATGCTGCTGGTGTGCTacAA	139228	139253	26	5
BART4-5P	BART4	GACCTGATGCTGCTGGTGTGCTGTAA	139228	139253	26	5
BART4-5P	BART4	GACCTGATGCTGCTGGTGTGCTGTtg	139228	139253	26	5
BART4-5P	BART4	GACCTGATGCTGCTGGTGTGCTtaAA	139228	139253	26	3
BART4-5P	BART4	tACCTGATGCTGCTGGTGTGCTacAA	139228	139253	26	10
BART4-5P	BART4	GACCTGATGCTGCTGGTGTGCTaTAAg	139228	139254	27	3
BART4-5P	BART4	GACCTGATGCTGCTGGTGTGCTGTAgg	139228	139254	27	3
BART4-5P	BART4	cCCTGATGCTGCTGGTGTGC	139229	139248	20	3
BART4-5P	BART4	ACtTGATGCTGCTGGTGTGCTc	139229	139250	22	3
BART4-3P	BART4-star	CACATCACGTAGGCACCAGGT	139266	139286	21	5
BART4-3P	BART4-star	CACATCACGTAGGCACCAGGTG	139266	139287	22	20
BART4-3P	BART4-star	CACATCACGTAGGCACCAaGTGT	139266	139288	23	4
BART4-3P	BART4-star	CACATCACGTAGGCACCAGaTGc	139266	139288	23	3
BART4-3P	BART4-star	CACATCACGTAGGCACCAGGTaa	139266	139288	23	5
BART4-3P	BART4-star	CACATCACGTAGGCACCAGGTaT	139266	139288	23	5
BART4-3P	BART4-star	CACATCACGTAGGCACCAGGTGa	139266	139288	23	3
BART4-3P	BART4-star	CACATCACGTAGGCACCAGGTGT	139266	139288	23	66
BART4-3P	BART4-star	CACATCACGTAGGCACCAGGTtg	139266	139288	23	5
BART4-3P	BART4-star	CACATCACGTAGGCACCAGGTGTCA	139266	139290	25	6
BART4-3P	BART4-star	CACATCACGTAGGCACCAGGTGTtt	139266	139290	25	3
BART4-3P	BART4-star	CACATCACGTAGGCACCAGGTGTCACC	139266	139292	27	3
BART1-5P	BART1-5P	TCTTAGTGGAAGTGACGTG	139351	139369	19	16
BART1-5P	BART1-5P	TCTTAGTGGAAGTGACGTaa	139351	139370	20	24
BART1-5P	BART1-5P	TCTTAGTGGAAGTGACGTGa	139351	139370	20	5
BART1-5P	BART1-5P	TCTTAGTGGAAGTGACGTGC	139351	139370	20	4
BART1-5P	BART1-5P	TCTTAGTGGAAGTGcaGTGC	139351	139370	20	5
BART1-5P	BART1-5P	TCTTAGTGGAAGTGcCGTGC	139351	139370	20	18
BART1-5P	BART1-5P	TCTTAGTGGAAGTGcCGTGt	139351	139370	20	8
BART1-5P	BART1-5P	TCTTAGTGGAAGTGACcTGCT	139351	139371	21	4
BART1-5P	BART1-5P	TCTTAGTGGAAGTGACGTGCT	139351	139371	21	4
BART1-5P	BART1-5P	TCTTAGTGGAAGTGACaTGCaG	139351	139372	22	6
BART1-5P	BART1-5P	TCTTAGTGGAAGTGACaTGCTc	139351	139372	22	12
BART1-5P	BART1-5P	TCTTAGTGGAAGTGACaTGCTG	139351	139372	22	3
BART1-5P	BART1-5P	TCTTAGTGGAAGTGACGcaCaa	139351	139372	22	3
BART1-5P	BART1-5P	TCTTAGTGGAAGTGACGgtaTG	139351	139372	22	8
BART1-5P	BART1-5P	TCTTAGTGGAAGTGACGTaCca	139351	139372	22	3
BART1-5P	BART1-5P	TCTTAGTGGAAGTGACGTaCTG	139351	139372	22	10
BART1-5P	BART1-5P	TCTTAGTGGAAGTGACGTGCac	139351	139372	22	9
BART1-5P	BART1-5P	TCTTAGTGGAAGTGACGTGCcG	139351	139372	22	20
BART1-5P	BART1-5P	TCTTAGTGGAAGTGACGTGCTG	139351	139372	22	20
BART1-5P	BART1-5P	TCTTAGTGGAAGTGACGaGCcGT	139351	139373	23	3
BART1-5P	BART1-5P	TCTTAGTGGAAGTGACGaGCTGT	139351	139373	23	5
BART1-5P	BART1-5P	TCTTAGTGGAAGTGACGcaCTGT	139351	139373	23	3
BART1-5P	BART1-5P	TCTTAGTGGAAGTGACGcGCTaT	139351	139373	23	6
BART1-5P	BART1-5P	TCTTAGTGGAAGTGACGcGCTGT	139351	139373	23	12
BART1-5P	BART1-5P	TCTTAGTGGAAGTGACGTaCTGT	139351	139373	23	25
BART1-5P	BART1-5P	TCTTAGTGGAAGTGACGTatTGT	139351	139373	23	5
BART1-5P	BART1-5P	TCTTAGTGGAAGTGACGTGCaGT	139351	139373	23	13
BART1-5P	BART1-5P	TCTTAGTGGAAGTGACGTGCcGT	139351	139373	23	4
BART1-5P	BART1-5P	TCTTAGTGGAAGTGACGTGCTaT	139351	139373	23	3
BART1-5P	BART1-5P	TCTTAGTGGAAGTGACGTGCTGT	139351	139373	23	25
BART1-5P	BART1-5P	TCTTAGTGGAAGTGACGTaCTcTG	139351	139374	24	5
BART1-5P	BART1-5P	TCTTAGTGGAAGTGACGTaCTGTG	139351	139374	24	25
BART1-5P	BART1-5P	TCTTAGTGGAAGTGACGTGCaGTG	139351	139374	24	17
BART1-5P	BART1-5P	TCTTAGTGGAAGTGACGTGCcGTG	139351	139374	24	3
BART1-5P	BART1-5P	TCTTAGTGGAAGTGACGTGCTaTG	139351	139374	24	9
BART1-5P	BART1-5P	TCTTAGTGGAAGTGACGTGCTGTG	139351	139374	24	18
BART1-5P	BART1-5P	TCTTAGTGGAAGTGACGTGtTaTG	139351	139374	24	9
BART1-5P	BART1-5P	TCTTAGTGGAAGTGACGTGCaGTGA	139351	139375	25	16
BART1-5P	BART1-5P	TCTTAGTGGAAGTGACGTGCaGTGt	139351	139375	25	3
BART1-5P	BART1-5P	TCTTAGTGGAAGTGACGTGCgGTGA	139351	139375	25	4
BART1-5P	BART1-5P	TCTTAGTGGAAGTGACGTGCTaTGA	139351	139375	25	5
BART1-5P	BART1-5P	TCTTAGTGGAAGTGACGTGCTGaaA	139351	139375	25	7
BART1-5P	BART1-5P	TCTTAGTGGAAGTGACGTGCTGTGA	139351	139375	25	12
BART1-5P	BART1-5P	TCTTAGTGGAAGTGACGTGCTGTGt	139351	139375	25	9
BART1-5P	BART1-5P	TCTTAGTGGAAGTGACGTGtTGTGA	139351	139375	25	4
BART1-5P	BART1-5P	TCTTAGTGGAAGTGACGTGCTGTacA	139351	139376	26	5

BART1-5P	BART1-5P	TCTTAGTGGAAGTGACGTGCTGTGAA	139351	139376	26	7
BART1-5P	BART1-5P	TCTTAGTGGAAGTGACGTGCTGTGAc	139351	139376	26	7
BART1-5P	BART1-5P	TCTTAGTGGAAGTGACGTGCTGTGAt	139351	139376	26	7
BART1-5P	BART1-5P	CTTAGTGGAAGTGACGTGCTGTG	139352	139374	23	4
BART1-3P	BART1-3P	TAGCACCGCTATCCACgAa	139387	139405	19	3
BART1-3P	BART1-3P	TAGCACCGCTATCCACTAa	139387	139405	19	7
BART1-3P	BART1-3P	TAGCACCGCTATCCACTAc	139387	139405	19	6
BART1-3P	BART1-3P	TAGCACCGCTATCCACTAT	139387	139405	19	10
BART1-3P	BART1-3P	TAGCACCGCTATCCACTga	139387	139405	19	7
BART1-3P	BART1-3P	TAGCACCGCTATCCACTgT	139387	139405	19	8
BART1-3P	BART1-3P	TAGCACCGCTATCCAaTATG	139387	139406	20	3
BART1-3P	BART1-3P	TAGCACCGCTATCCAaTATt	139387	139406	20	5
BART1-3P	BART1-3P	TAGCACCGCTATCCACaATa	139387	139406	20	7
BART1-3P	BART1-3P	TAGCACCGCTATCCACaATG	139387	139406	20	6
BART1-3P	BART1-3P	TAGCACCGCTATCCACatTG	139387	139406	20	3
BART1-3P	BART1-3P	TAGCACCGCTATCCACcATG	139387	139406	20	3
BART1-3P	BART1-3P	TAGCACCGCTATCCACcATt	139387	139406	20	3
BART1-3P	BART1-3P	TAGCACCGCTATCCACcgTG	139387	139406	20	8
BART1-3P	BART1-3P	TAGCACCGCTATCCACTAca	139387	139406	20	42
BART1-3P	BART1-3P	TAGCACCGCTATCCACTATa	139387	139406	20	4
BART1-3P	BART1-3P	TAGCACCGCTATCCACTATG	139387	139406	20	28
BART1-3P	BART1-3P	TAGCACCGCTATCCACTATt	139387	139406	20	7
BART1-3P	BART1-3P	TAGCACCGCTATCCACTcac	139387	139406	20	9
BART1-3P	BART1-3P	TAGCACCGCTATCCACTcat	139387	139406	20	3
BART1-3P	BART1-3P	TAGCACCGCTATCCACTcgG	139387	139406	20	8
BART1-3P	BART1-3P	TAGCACCGCTATCCACTgTG	139387	139406	20	8
BART1-3P	BART1-3P	cAGCACCGCTATCCACTATGT	139387	139407	21	5
BART1-3P	BART1-3P	TAGCACCGCTATCacaTATGT	139387	139407	21	5
BART1-3P	BART1-3P	TAGCACCGCTATCCAaTATGT	139387	139407	21	23
BART1-3P	BART1-3P	TAGCACCGCTATCCACaATcT	139387	139407	21	3
BART1-3P	BART1-3P	TAGCACCGCTATCCACcATaT	139387	139407	21	7
BART1-3P	BART1-3P	TAGCACCGCTATCCACcATGT	139387	139407	21	22
BART1-3P	BART1-3P	TAGCACCGCTATCCACcgTGT	139387	139407	21	3
BART1-3P	BART1-3P	TAGCACCGCTATCCACgATGT	139387	139407	21	28
BART1-3P	BART1-3P	TAGCACCGCTATCCACTAaaT	139387	139407	21	4
BART1-3P	BART1-3P	TAGCACCGCTATCCACTAacT	139387	139407	21	10
BART1-3P	BART1-3P	TAGCACCGCTATCCACTAatc	139387	139407	21	3
BART1-3P	BART1-3P	TAGCACCGCTATCCACTAcaT	139387	139407	21	5
BART1-3P	BART1-3P	TAGCACCGCTATCCACTAcGT	139387	139407	21	14
BART1-3P	BART1-3P	TAGCACCGCTATCCACTAgac	139387	139407	21	3
BART1-3P	BART1-3P	TAGCACCGCTATCCACTAgtc	139387	139407	21	33
BART1-3P	BART1-3P	TAGCACCGCTATCCACTATaT	139387	139407	21	38
BART1-3P	BART1-3P	TAGCACCGCTATCCACTATcT	139387	139407	21	29
BART1-3P	BART1-3P	TAGCACCGCTATCCACTATGa	139387	139407	21	25
BART1-3P	BART1-3P	TAGCACCGCTATCCACTATGc	139387	139407	21	76
BART1-3P	BART1-3P	TAGCACCGCTATCCACTATGg	139387	139407	21	4
BART1-3P	BART1-3P	TAGCACCGCTATCCACTATGT	139387	139407	21	1204
BART1-3P	BART1-3P	TAGCACCGCTATCCACTATtg	139387	139407	21	3
BART1-3P	BART1-3P	TAGCACCGCTATCCACTcTGT	139387	139407	21	7
BART1-3P	BART1-3P	TAGCACCGCTATCCACTgacT	139387	139407	21	9
BART1-3P	BART1-3P	TAGCACCGCTATCCACTgcGT	139387	139407	21	4
BART1-3P	BART1-3P	TAGCACCGCTATCCACTgTca	139387	139407	21	6
BART1-3P	BART1-3P	TAGCACCGCTATCCACTgTGc	139387	139407	21	4
BART1-3P	BART1-3P	TAGCACCGCTATCCACTgTGT	139387	139407	21	5
BART1-3P	BART1-3P	TAGCACCGCTATCCACTtatT	139387	139407	21	3
BART1-3P	BART1-3P	TAGCACCGCTATCCAgTATGT	139387	139407	21	34
BART1-3P	BART1-3P	TAGCACCGCTATCCAtcATGT	139387	139407	21	7
BART1-3P	BART1-3P	TAGCACCGCTATCCAtTATGT	139387	139407	21	30
BART1-3P	BART1-3P	cAGCACCGCTATCCACTATaTC	139387	139408	22	3
BART1-3P	BART1-3P	cAGCACCGCTATCCACTATGTC	139387	139408	22	3
BART1-3P	BART1-3P	TAGCACCaCTATCCACTATGTC	139387	139408	22	10
BART1-3P	BART1-3P	TAGCACCGCTATCCACaATGcC	139387	139408	22	4
BART1-3P	BART1-3P	TAGCACCGCTATCCACaATGTC	139387	139408	22	40
BART1-3P	BART1-3P	TAGCACCGCTATCCACagcGTC	139387	139408	22	3
BART1-3P	BART1-3P	TAGCACCGCTATCCACcATacC	139387	139408	22	3
BART1-3P	BART1-3P	TAGCACCGCTATCCACcATGcC	139387	139408	22	11
BART1-3P	BART1-3P	TAGCACCGCTATCCACcATGTC	139387	139408	22	73
BART1-3P	BART1-3P	TAGCACCGCTATCCACcATGTg	139387	139408	22	24

BART1-3P	BART1-3P	TAGCACCGCTATCCACgATGcC	139387	139408	22	9
BART1-3P	BART1-3P	TAGCACCGCTATCCACgATGTC	139387	139408	22	70
BART1-3P	BART1-3P	TAGCACCGCTATCCACTAacTC	139387	139408	22	16
BART1-3P	BART1-3P	TAGCACCGCTATCCACTAaGTC	139387	139408	22	24
BART1-3P	BART1-3P	TAGCACCGCTATCCACTAcaTC	139387	139408	22	10
BART1-3P	BART1-3P	TAGCACCGCTATCCACTAcGcC	139387	139408	22	4
BART1-3P	BART1-3P	TAGCACCGCTATCCACTAcGTC	139387	139408	22	24
BART1-3P	BART1-3P	TAGCACCGCTATCCACTAgcTC	139387	139408	22	3
BART1-3P	BART1-3P	TAGCACCGCTATCCACTAgGTC	139387	139408	22	7
BART1-3P	BART1-3P	TAGCACCGCTATCCACTAgtcC	139387	139408	22	3
BART1-3P	BART1-3P	TAGCACCGCTATCCACTATaTC	139387	139408	22	30
BART1-3P	BART1-3P	TAGCACCGCTATCCACTATcTC	139387	139408	22	5
BART1-3P	BART1-3P	TAGCACCGCTATCCACTATGaC	139387	139408	22	23
BART1-3P	BART1-3P	TAGCACCGCTATCCACTATGcC	139387	139408	22	249
BART1-3P	BART1-3P	TAGCACCGCTATCCACTATGga	139387	139408	22	19
BART1-3P	BART1-3P	TAGCACCGCTATCCACTATGgC	139387	139408	22	3
BART1-3P	BART1-3P	TAGCACCGCTATCCACTATGTa	139387	139408	22	13
BART1-3P	BART1-3P	TAGCACCGCTATCCACTATGTC	139387	139408	22	674
BART1-3P	BART1-3P	TAGCACCGCTATCCACTATGTg	139387	139408	22	5
BART1-3P	BART1-3P	TAGCACCGCTATCCACTcTGTC	139387	139408	22	5
BART1-3P	BART1-3P	TAGCACCGCTATCCACTgaGTC	139387	139408	22	6
BART1-3P	BART1-3P	TAGCACCGCTATCCACTgTaTC	139387	139408	22	4
BART1-3P	BART1-3P	TAGCACCGCTATCCACTgTGcC	139387	139408	22	9
BART1-3P	BART1-3P	TAGCACCGCTATCCACTgTGTC	139387	139408	22	13
BART1-3P	BART1-3P	TAGCACCGCTATCCACTtaGTC	139387	139408	22	8
BART1-3P	BART1-3P	TAGCACCGCTATCCgCTATGcC	139387	139408	22	3
BART1-3P	BART1-3P	cAGCACCGCTATCCACTATGTCT	139387	139409	23	7
BART1-3P	BART1-3P	TAGCACCGCTATCacaTATGTCa	139387	139409	23	3
BART1-3P	BART1-3P	TAGCACCGCTATCCACTAaaaCc	139387	139409	23	11
BART1-3P	BART1-3P	TAGCACCGCTATCCACTAaaTCT	139387	139409	23	6
BART1-3P	BART1-3P	TAGCACCGCTATCCACTAaGgaa	139387	139409	23	3
BART1-3P	BART1-3P	TAGCACCGCTATCCACTAaGgCT	139387	139409	23	5
BART1-3P	BART1-3P	TAGCACCGCTATCCACTAaGTCa	139387	139409	23	15
BART1-3P	BART1-3P	TAGCACCGCTATCCACTAaGTCc	139387	139409	23	8
BART1-3P	BART1-3P	TAGCACCGCTATCCACTAatcCT	139387	139409	23	4
BART1-3P	BART1-3P	TAGCACCGCTATCCACTAcaaCT	139387	139409	23	7
BART1-3P	BART1-3P	TAGCACCGCTATCCACTAcaTCa	139387	139409	23	3
BART1-3P	BART1-3P	TAGCACCGCTATCCACTAcaTCT	139387	139409	23	25
BART1-3P	BART1-3P	TAGCACCGCTATCCACTAcGTCa	139387	139409	23	20
BART1-3P	BART1-3P	TAGCACCGCTATCCACTAcGTCT	139387	139409	23	6
BART1-3P	BART1-3P	TAGCACCGCTATCCACTAgcTCT	139387	139409	23	3
BART1-3P	BART1-3P	TAGCACCGCTATCCACTAgGcCT	139387	139409	23	9
BART1-3P	BART1-3P	TAGCACCGCTATCCACTAgGTCa	139387	139409	23	14
BART1-3P	BART1-3P	TAGCACCGCTATCCACTAgGTCT	139387	139409	23	12
BART1-3P	BART1-3P	TAGCACCGCTATCCACTATacCa	139387	139409	23	19
BART1-3P	BART1-3P	TAGCACCGCTATCCACTATagCc	139387	139409	23	5
BART1-3P	BART1-3P	TAGCACCGCTATCCACTATaTag	139387	139409	23	7
BART1-3P	BART1-3P	TAGCACCGCTATCCACTATaTCa	139387	139409	23	4
BART1-3P	BART1-3P	TAGCACCGCTATCCACTATaTCT	139387	139409	23	32
BART1-3P	BART1-3P	TAGCACCGCTATCCACTATGaac	139387	139409	23	5
BART1-3P	BART1-3P	TAGCACCGCTATCCACTATGaCc	139387	139409	23	13
BART1-3P	BART1-3P	TAGCACCGCTATCCACTATGaCT	139387	139409	23	22
BART1-3P	BART1-3P	TAGCACCGCTATCCACTATGagc	139387	139409	23	4
BART1-3P	BART1-3P	TAGCACCGCTATCCACTATGatc	139387	139409	23	4
BART1-3P	BART1-3P	TAGCACCGCTATCCACTATGcaa	139387	139409	23	4
BART1-3P	BART1-3P	TAGCACCGCTATCCACTATGcac	139387	139409	23	5
BART1-3P	BART1-3P	TAGCACCGCTATCCACTATGcag	139387	139409	23	3
BART1-3P	BART1-3P	TAGCACCGCTATCCACTATGcaT	139387	139409	23	5
BART1-3P	BART1-3P	TAGCACCGCTATCCACTATGcCa	139387	139409	23	26
BART1-3P	BART1-3P	TAGCACCGCTATCCACTATGcCc	139387	139409	23	19
BART1-3P	BART1-3P	TAGCACCGCTATCCACTATGcCg	139387	139409	23	9
BART1-3P	BART1-3P	TAGCACCGCTATCCACTATGcCT	139387	139409	23	148
BART1-3P	BART1-3P	TAGCACCGCTATCCACTATGcgc	139387	139409	23	16
BART1-3P	BART1-3P	TAGCACCGCTATCCACTATGctc	139387	139409	23	4
BART1-3P	BART1-3P	TAGCACCGCTATCCACTATGgac	139387	139409	23	4
BART1-3P	BART1-3P	TAGCACCGCTATCCACTATGgCT	139387	139409	23	3
BART1-3P	BART1-3P	TAGCACCGCTATCCACTATGgtc	139387	139409	23	34
BART1-3P	BART1-3P	TAGCACCGCTATCCACTATGgtg	139387	139409	23	10

BART1-3P	BART1-3P	TAGCACCGCTATCCACTATGTaT	139387	139409	23	8
BART1-3P	BART1-3P	TAGCACCGCTATCCACTATGTCa	139387	139409	23	221
BART1-3P	BART1-3P	TAGCACCGCTATCCACTATGTCc	139387	139409	23	127
BART1-3P	BART1-3P	TAGCACCGCTATCCACTATGTCg	139387	139409	23	45
BART1-3P	BART1-3P	TAGCACCGCTATCCACTATGTCT	139387	139409	23	881
BART1-3P	BART1-3P	TAGCACCGCTATCCACTATGTgc	139387	139409	23	4
BART1-3P	BART1-3P	TAGCACCGCTATCCACTATGTtc	139387	139409	23	5
BART1-3P	BART1-3P	TAGCACCGCTATCCACTcTGTCT	139387	139409	23	3
BART1-3P	BART1-3P	TAGCACCGCTATCCACTcTGTtT	139387	139409	23	4
BART1-3P	BART1-3P	TAGCACCGCTATCCACTgTaTCT	139387	139409	23	4
BART1-3P	BART1-3P	TAGCACCGCTATCCACTgTGcCT	139387	139409	23	3
BART1-3P	BART1-3P	TAGCACCGCTATCCACTgTGTCa	139387	139409	23	4
BART1-3P	BART1-3P	TAGCACCGCTATCCACTgTGTCc	139387	139409	23	5
BART1-3P	BART1-3P	TAGCACCGCTATCCACTgTGTCT	139387	139409	23	52
BART1-3P	BART1-3P	TAGCACCGCTATCCACTtTGTCT	139387	139409	23	6
BART1-3P	BART1-3P	TAGCAtCGCTATCCACTATGTCT	139387	139409	23	6
BART1-3P	BART1-3P	TgGCACCGCTATCCACTATGTCT	139387	139409	23	5
BART1-3P	BART1-3P	TAGCACCGCTATCCACTAcGTCTa	139387	139410	24	3
BART1-3P	BART1-3P	TAGCACCGCTATCCACTAcGTCTt	139387	139410	24	5
BART1-3P	BART1-3P	TAGCACCGCTATCCACTAgGTCaC	139387	139410	24	3
BART1-3P	BART1-3P	TAGCACCGCTATCCACTAgGTCTa	139387	139410	24	5
BART1-3P	BART1-3P	TAGCACCGCTATCCACTAgGTCTC	139387	139410	24	5
BART1-3P	BART1-3P	TAGCACCGCTATCCACTATaTCTt	139387	139410	24	3
BART1-3P	BART1-3P	TAGCACCGCTATCCACTATGcCTC	139387	139410	24	9
BART1-3P	BART1-3P	TAGCACCGCTATCCACTATGcCTt	139387	139410	24	4
BART1-3P	BART1-3P	TAGCACCGCTATCCACTATGTaga	139387	139410	24	11
BART1-3P	BART1-3P	TAGCACCGCTATCCACTATGTCaC	139387	139410	24	3
BART1-3P	BART1-3P	TAGCACCGCTATCCACTATGTCga	139387	139410	24	10
BART1-3P	BART1-3P	TAGCACCGCTATCCACTATGTCTa	139387	139410	24	33
BART1-3P	BART1-3P	TAGCACCGCTATCCACTATGTCTC	139387	139410	24	73
BART1-3P	BART1-3P	TAGCACCGCTATCCACTATGTCTg	139387	139410	24	7
BART1-3P	BART1-3P	TAGCACCGCTATCCACTATGTCTt	139387	139410	24	8
BART1-3P	BART1-3P	TAGCACCGCTATCCACTATGTCTCt	139387	139411	25	3
BART1-3P	BART1-3P	TAGCACCGCTATCCACTATGTCTgG	139387	139411	25	8
BART1-3P	BART1-3P	AGCACCGCTATCCACcATcc	139388	139407	20	5
BART1-3P	BART1-3P	AGCACCGCTATCCACcATGT	139388	139407	20	5
BART1-3P	BART1-3P	AGCACCGCTATCCACTATGc	139388	139407	20	3
BART1-3P	BART1-3P	AGCACCGCTATCCACTATGT	139388	139407	20	30
BART1-3P	BART1-3P	AGCACCGCTATCCACTgTca	139388	139407	20	5
BART1-3P	BART1-3P	AGCACCGCTATCCAgTATGT	139388	139407	20	3
BART1-3P	BART1-3P	AGCACCGCTATCCACTAcGTC	139388	139408	21	4
BART1-3P	BART1-3P	AGCACCGCTATCCACTATGTC	139388	139408	21	13
BART1-3P	BART1-3P	AGCACCGCTATCCACTAgGTCT	139388	139409	22	4
BART1-3P	BART1-3P	AGCACCGCTATCCACTATGcCg	139388	139409	22	6
BART1-3P	BART1-3P	AGCACCGCTATCCACTATGcCT	139388	139409	22	5
BART1-3P	BART1-3P	AGCACCGCTATCCACTATGTCa	139388	139409	22	17
BART1-3P	BART1-3P	AGCACCGCTATCCACTATGTCT	139388	139409	22	52
BART15-5P		AGGGAAACATGACCACCTGAAGTC	139519	139542	24	3
BART15-3P	BART15	GGTCAGTGGTTTTGTTTCCTTGta	139552	139575	24	3
BART15-3P	BART15	GTCAGTGGTTTTGTTTCCTT	139553	139572	20	5
BART15-3P	BART15	GTCAGTGGTTTTGTTaCCTTc	139553	139573	21	5
BART15-3P	BART15	GTCAGTGGTTTTGTTTCCTaG	139553	139573	21	4
BART15-3P	BART15	GTCAGTGGTTTTGTTTCCTcc	139553	139573	21	4
BART15-3P	BART15	GTCAGTGGTTTTGTTTCCTcG	139553	139573	21	6
BART15-3P	BART15	GTCAGTGGTTTTGTTTCCTTc	139553	139573	21	10
BART15-3P	BART15	GTCAGTGGTTTTGTTTCCTTG	139553	139573	21	43
BART15-3P	BART15	GTCAGTGGTTTTGTTTCtTTc	139553	139573	21	3
BART15-3P	BART15	aTCAGTGGTTTTGTTTCCTTGA	139553	139574	22	6
BART15-3P	BART15	cTCAGTGGTTTTGTTTCCTTGA	139553	139574	22	3
BART15-3P	BART15	GTCAGTGGcTTTGTTTCCTTGA	139553	139574	22	20
BART15-3P	BART15	GTCAGTGGTTTTGTcTCCTTGA	139553	139574	22	11
BART15-3P	BART15	GTCAGTGGTTTTGTTTaCaTGA	139553	139574	22	6
BART15-3P	BART15	GTCAGTGGTTTTGTTTaCTTGA	139553	139574	22	102
BART15-3P	BART15	GTCAGTGGTTTTGTTTCagTGA	139553	139574	22	7
BART15-3P	BART15	GTCAGTGGTTTTGTTTCaTaGA	139553	139574	22	4
BART15-3P	BART15	GTCAGTGGTTTTGTTTCaTgGA	139553	139574	22	3
BART15-3P	BART15	GTCAGTGGTTTTGTTTCaTTGA	139553	139574	22	29
BART15-3P	BART15	GTCAGTGGTTTTGTTTCCaTGA	139553	139574	22	34

BART15-3P	BART15	GTCAGTGGTTTTGTTTCCcatA	139553	139574	22	3
BART15-3P	BART15	GTCAGTGGTTTTGTTTCCccGA	139553	139574	22	5
BART15-3P	BART15	GTCAGTGGTTTTGTTTCCcTGA	139553	139574	22	62
BART15-3P	BART15	GTCAGTGGTTTTGTTTCCTaGA	139553	139574	22	21
BART15-3P	BART15	GTCAGTGGTTTTGTTTCCTcGA	139553	139574	22	26
BART15-3P	BART15	GTCAGTGGTTTTGTTTCCTTaA	139553	139574	22	17
BART15-3P	BART15	GTCAGTGGTTTTGTTTCCTTcA	139553	139574	22	3
BART15-3P	BART15	GTCAGTGGTTTTGTTTCCTTGA	139553	139574	22	1212
BART15-3P	BART15	GTCAGTGGTTTTGTTTCgaTGA	139553	139574	22	3
BART15-3P	BART15	GTCAGTGGTTTTGTTTCgTTGA	139553	139574	22	6
BART15-3P	BART15	GTCAGTGGTTTTGTTTCtcTGA	139553	139574	22	4
BART15-3P	BART15	GTCAGTGGTTTTGTTTCtTTGA	139553	139574	22	7
BART15-3P	BART15	GTCAGTGGTTTTGTTTgCTTGA	139553	139574	22	23
BART15-3P	BART15	GTCAGTGGTTTTGTTTtCTTGA	139553	139574	22	32
BART15-3P	BART15	tgCAGTGGTTTTGTTTCCTTGA	139553	139574	22	5
BART15-3P	BART15	tTCAGTGGTTTTGTTTCCTTGA	139553	139574	22	3
BART15-3P	BART15	GTCAGTGGTTTTGTTTCCaTGAa	139553	139575	23	7
BART15-3P	BART15	GTCAGTGGTTTTGTTTCCaTGga	139553	139575	23	5
BART15-3P	BART15	GTCAGTGGTTTTGTTTCCTaGta	139553	139575	23	3
BART15-3P	BART15	GTCAGTGGTTTTGTTTCCTccAg	139553	139575	23	4
BART15-3P	BART15	GTCAGTGGTTTTGTTTCCTTacT	139553	139575	23	8
BART15-3P	BART15	GTCAGTGGTTTTGTTTCCTTctc	139553	139575	23	5
BART15-3P	BART15	GTCAGTGGTTTTGTTTCCTTGAa	139553	139575	23	57
BART15-3P	BART15	GTCAGTGGTTTTGTTTCCTTGAc	139553	139575	23	10
BART15-3P	BART15	GTCAGTGGTTTTGTTTCCTTGAg	139553	139575	23	3
BART15-3P	BART15	GTCAGTGGTTTTGTTTCCTTGAT	139553	139575	23	15
BART15-3P	BART15	GTCAGTGGTTTTGTTTCCTTGcT	139553	139575	23	3
BART15-3P	BART15	GTCAGTGGTTTTGTTTCCTTGga	139553	139575	23	6
BART15-3P	BART15	GTCAGTGGTTTTGTTTCCTTGgT	139553	139575	23	3
BART15-3P	BART15	GTCAGTGGTTTTGTTTCCTTGtc	139553	139575	23	4
BART15-3P	BART15	GTCAGTGGTTTTGTTTCCTTGtT	139553	139575	23	4
BART15-3P	BART15	GTCAGTGGTTTTGTTTCCTTaATAt	139553	139577	25	3
BART15-3P	BART15	GTCAGTGGTTTTGTTTCCTTGATAcA	139553	139578	26	5
BART15-3P	BART15	gCAGTGGTTTTGTTTCCTTGAaAc	139554	139577	24	9
BART5-5P	BART5	TCAAGGTGAATATAGCTGCCCAT	139674	139696	23	3
BART5-5P	BART5	TCAAGGTGAATATAGCTGCCCATC	139674	139697	24	14
BART5-5P	BART5	TCAAGGTGAATATAGCTGCCCATCG	139674	139698	25	12
BART5-5P	BART5	TCAAGGTGAATATAGCTGCtCATCG	139674	139698	25	3
BART5-5P	BART5	gCAAGGTGAATATAGCTGCCCATCGA	139674	139699	26	3
BART5-5P	BART5	TCAAGGTGAATATAGCTGCCCATCGA	139674	139699	26	3
BART5-5P	BART5	CAAGGTGAATATAGCcaCC	139675	139693	19	5
BART5-5P	BART5	CAAGGTGAATATAGCTGaC	139675	139693	19	35
BART5-5P	BART5	CAAGGTGAATATAGCTGCC	139675	139693	19	60
BART5-5P	BART5	CAAGGTGAATATAGCTGgt	139675	139693	19	16
BART5-5P	BART5	CAAGGTGAATATAGaTGCCa	139675	139694	20	6
BART5-5P	BART5	CAAGGTGAATATAGCcGCaC	139675	139694	20	3
BART5-5P	BART5	CAAGGTGAATATAGCTaCCg	139675	139694	20	4
BART5-5P	BART5	CAAGGTGAATATAGCTcCCg	139675	139694	20	6
BART5-5P	BART5	CAAGGTGAATATAGCTGCCa	139675	139694	20	5
BART5-5P	BART5	CAAGGTGAATATAGCTGCCC	139675	139694	20	47
BART5-5P	BART5	CAAGGTGAATATAGCTGCCg	139675	139694	20	4
BART5-5P	BART5	CAAGGTGAATATAGCTGCCt	139675	139694	20	3
BART5-5P	BART5	CAAGGTGAATATAGtTGCCt	139675	139694	20	3
BART5-5P	BART5	CAAGGTGAATATAGCaaCCCA	139675	139695	21	3
BART5-5P	BART5	CAAGGTGAATATAGCaGCCCA	139675	139695	21	38
BART5-5P	BART5	CAAGGTGAATATAGCcaCCCA	139675	139695	21	5
BART5-5P	BART5	CAAGGTGAATATAGCcGCCCA	139675	139695	21	10
BART5-5P	BART5	CAAGGTGAATATAGCcGCCCg	139675	139695	21	3
BART5-5P	BART5	CAAGGTGAATATAGCgGCCCA	139675	139695	21	4
BART5-5P	BART5	CAAGGTGAATATAGCTaCCaA	139675	139695	21	3
BART5-5P	BART5	CAAGGTGAATATAGCTaCCCA	139675	139695	21	108
BART5-5P	BART5	CAAGGTGAATATAGCTaCCCg	139675	139695	21	7
BART5-5P	BART5	CAAGGTGAATATAGCTcCCCA	139675	139695	21	9
BART5-5P	BART5	CAAGGTGAATATAGCTGaCCA	139675	139695	21	4
BART5-5P	BART5	CAAGGTGAATATAGCTGatCA	139675	139695	21	3
BART5-5P	BART5	CAAGGTGAATATAGCTGCaCA	139675	139695	21	3
BART5-5P	BART5	CAAGGTGAATATAGCTGCCaA	139675	139695	21	11
BART5-5P	BART5	CAAGGTGAATATAGCTGCCCA	139675	139695	21	169

BART5-5P	BART5	CAAGGTGAATATAGCTGCCCg	139675	139695	21	11
BART5-5P	BART5	CAAGGTGAATATAGCTGCCCt	139675	139695	21	10
BART5-5P	BART5	CAAGGTGAATATAGCTGCCgA	139675	139695	21	16
BART5-5P	BART5	CAAGGTGAATATAGCTGCtCA	139675	139695	21	3
BART5-5P	BART5	CAAGGTGAATATAGCTtCCCA	139675	139695	21	24
BART5-5P	BART5	agAGGTGAATATAGCTGCCCca	139675	139696	22	7
BART5-5P	BART5	atAGGTGAATATAGCTGCCCAT	139675	139696	22	3
BART5-5P	BART5	CAAcGTGAATATAGCTGCCCtT	139675	139696	22	7
BART5-5P	BART5	CAAGGTGAAgcgAGCTGCCCAT	139675	139696	22	4
BART5-5P	BART5	CAAGGTGAATATAGCTaaCCAa	139675	139696	22	14
BART5-5P	BART5	CAAGGTGAATATAGCTaaCCAc	139675	139696	22	3
BART5-5P	BART5	CAAGGTGAATATAGCTaaCCAT	139675	139696	22	78
BART5-5P	BART5	CAAGGTGAATATAGCTaCaCAT	139675	139696	22	6
BART5-5P	BART5	CAAGGTGAATATAGCTaCCaAc	139675	139696	22	3
BART5-5P	BART5	CAAGGTGAATATAGCTaCCaAT	139675	139696	22	17
BART5-5P	BART5	CAAGGTGAATATAGCTaCCaca	139675	139696	22	14
BART5-5P	BART5	CAAGGTGAATATAGCTaCCCAa	139675	139696	22	46
BART5-5P	BART5	CAAGGTGAATATAGCTaCCCAc	139675	139696	22	65
BART5-5P	BART5	CAAGGTGAATATAGCTaCCCAg	139675	139696	22	6
BART5-5P	BART5	CAAGGTGAATATAGCTaCCCAT	139675	139696	22	270
BART5-5P	BART5	CAAGGTGAATATAGCTaCCCca	139675	139696	22	14
BART5-5P	BART5	CAAGGTGAATATAGCTaCCCcg	139675	139696	22	11
BART5-5P	BART5	CAAGGTGAATATAGCTaCCCcT	139675	139696	22	18
BART5-5P	BART5	CAAGGTGAATATAGCTaCCCga	139675	139696	22	4
BART5-5P	BART5	CAAGGTGAATATAGCTaCCCgT	139675	139696	22	5
BART5-5P	BART5	CAAGGTGAATATAGCTaCCCtc	139675	139696	22	5
BART5-5P	BART5	CAAGGTGAATATAGCTaCCCtT	139675	139696	22	10
BART5-5P	BART5	CAAGGTGAATATAGCTaCCtAT	139675	139696	22	9
BART5-5P	BART5	CAAGGTGAATATAGCTaCtCAT	139675	139696	22	3
BART5-5P	BART5	CAAGGTGAATATAGCTatCCAa	139675	139696	22	3
BART5-5P	BART5	CAAGGTGAATATAGCTatCCAc	139675	139696	22	3
BART5-5P	BART5	CAAGGTGAATATAGCTatCCAT	139675	139696	22	73
BART5-5P	BART5	CAAGGTGAATATAGCTcCCCAc	139675	139696	22	4
BART5-5P	BART5	CAAGGTGAATATAGCTcCCCAT	139675	139696	22	11
BART5-5P	BART5	CAAGGTGAATATAGCTGaCCAa	139675	139696	22	7
BART5-5P	BART5	CAAGGTGAATATAGCTGaCCAc	139675	139696	22	20
BART5-5P	BART5	CAAGGTGAATATAGCTGaCCAT	139675	139696	22	51
BART5-5P	BART5	CAAGGTGAATATAGCTGCaagT	139675	139696	22	3
BART5-5P	BART5	CAAGGTGAATATAGCTGCaCAa	139675	139696	22	5
BART5-5P	BART5	CAAGGTGAATATAGCTGCaCAc	139675	139696	22	6
BART5-5P	BART5	CAAGGTGAATATAGCTGCaCAT	139675	139696	22	13
BART5-5P	BART5	CAAGGTGAATATAGCTGCCaAa	139675	139696	22	5
BART5-5P	BART5	CAAGGTGAATATAGCTGCCaAT	139675	139696	22	18
BART5-5P	BART5	CAAGGTGAATATAGCTGCCaca	139675	139696	22	3
BART5-5P	BART5	CAAGGTGAATATAGCTGCCacc	139675	139696	22	31
BART5-5P	BART5	CAAGGTGAATATAGCTGCCacg	139675	139696	22	6
BART5-5P	BART5	CAAGGTGAATATAGCTGCCacT	139675	139696	22	12
BART5-5P	BART5	CAAGGTGAATATAGCTGCCata	139675	139696	22	15
BART5-5P	BART5	CAAGGTGAATATAGCTGCCatc	139675	139696	22	57
BART5-5P	BART5	CAAGGTGAATATAGCTGCCCAa	139675	139696	22	76
BART5-5P	BART5	CAAGGTGAATATAGCTGCCCAc	139675	139696	22	416
BART5-5P	BART5	CAAGGTGAATATAGCTGCCCAT	139675	139696	22	929
BART5-5P	BART5	CAAGGTGAATATAGCTGCCCca	139675	139696	22	12
BART5-5P	BART5	CAAGGTGAATATAGCTGCCCcc	139675	139696	22	7
BART5-5P	BART5	CAAGGTGAATATAGCTGCCCcg	139675	139696	22	19
BART5-5P	BART5	CAAGGTGAATATAGCTGCCCcT	139675	139696	22	28
BART5-5P	BART5	CAAGGTGAATATAGCTGCCCga	139675	139696	22	27
BART5-5P	BART5	CAAGGTGAATATAGCTGCCCgc	139675	139696	22	6
BART5-5P	BART5	CAAGGTGAATATAGCTGCCCgg	139675	139696	22	3
BART5-5P	BART5	CAAGGTGAATATAGCTGCCCgT	139675	139696	22	23
BART5-5P	BART5	CAAGGTGAATATAGCTGCCCta	139675	139696	22	23
BART5-5P	BART5	CAAGGTGAATATAGCTGCCCtc	139675	139696	22	28
BART5-5P	BART5	CAAGGTGAATATAGCTGCCCtg	139675	139696	22	11
BART5-5P	BART5	CAAGGTGAATATAGCTGCCCtT	139675	139696	22	21
BART5-5P	BART5	CAAGGTGAATATAGCTGCCgAc	139675	139696	22	11
BART5-5P	BART5	CAAGGTGAATATAGCTGCCgAT	139675	139696	22	19
BART5-5P	BART5	CAAGGTGAATATAGCTGCCgca	139675	139696	22	4
BART5-5P	BART5	CAAGGTGAATATAGCTGCCgcT	139675	139696	22	6

BART5-5P	BART5	CAAGGTGAATATAGCTGCCggT	139675	139696	22	3
BART5-5P	BART5	CAAGGTGAATATAGCTGCCgtT	139675	139696	22	3
BART5-5P	BART5	CAAGGTGAATATAGCTGCCtAT	139675	139696	22	16
BART5-5P	BART5	CAAGGTGAATATAGCTGCtCAT	139675	139696	22	4
BART5-5P	BART5	CAAGGTGAATATAGCTGtCCAa	139675	139696	22	6
BART5-5P	BART5	CAAGGTGAATATAGCTGtCCAc	139675	139696	22	15
BART5-5P	BART5	CAAGGTGAATATAGCTGtCCAT	139675	139696	22	34
BART5-5P	BART5	CAAGGTGAATATAGCTGtCCcT	139675	139696	22	3
BART5-5P	BART5	CAAGGTGAATATAGCTtCCCAT	139675	139696	22	8
BART5-5P	BART5	CAAGGTGAATATctCTGCCCAT	139675	139696	22	3
BART5-5P	BART5	CAAGGTGtATATAGCTGCCCAT	139675	139696	22	3
BART5-5P	BART5	CcAGGTGAATATAGCTGCCCtT	139675	139696	22	3
BART5-5P	BART5	tAAGGTGAATATAGCTGCCCAc	139675	139696	22	5
BART5-5P	BART5	tAAGGTGAATATAGCTGCCCAT	139675	139696	22	3
BART5-5P	BART5	CAAGGTGAATATAGCcGCCCATC	139675	139697	23	4
BART5-5P	BART5	CAAGGTGAATATAGCTaCCCATC	139675	139697	23	3
BART5-5P	BART5	CAAGGTGAATATAGCTGaCCAcC	139675	139697	23	19
BART5-5P	BART5	CAAGGTGAATATAGCTGaCCATa	139675	139697	23	27
BART5-5P	BART5	CAAGGTGAATATAGCTGaCCATC	139675	139697	23	114
BART5-5P	BART5	CAAGGTGAATATAGCTGaCCATt	139675	139697	23	13
BART5-5P	BART5	CAAGGTGAATATAGCTGCaCAcC	139675	139697	23	4
BART5-5P	BART5	CAAGGTGAATATAGCTGCaCATC	139675	139697	23	12
BART5-5P	BART5	CAAGGTGAATATAGCTGCCCAaC	139675	139697	23	85
BART5-5P	BART5	CAAGGTGAATATAGCTGCCCAag	139675	139697	23	7
BART5-5P	BART5	CAAGGTGAATATAGCTGCCCAat	139675	139697	23	3
BART5-5P	BART5	CAAGGTGAATATAGCTGCCCAcC	139675	139697	23	229
BART5-5P	BART5	CAAGGTGAATATAGCTGCCCAgC	139675	139697	23	23
BART5-5P	BART5	CAAGGTGAATATAGCTGCCCAgt	139675	139697	23	6
BART5-5P	BART5	CAAGGTGAATATAGCTGCCCATa	139675	139697	23	266
BART5-5P	BART5	CAAGGTGAATATAGCTGCCCATC	139675	139697	23	1228
BART5-5P	BART5	CAAGGTGAATATAGCTGCCCATg	139675	139697	23	62
BART5-5P	BART5	CAAGGTGAATATAGCTGCCCATt	139675	139697	23	112
BART5-5P	BART5	CAAGGTGAATATAGCTGCCCcaC	139675	139697	23	9
BART5-5P	BART5	CAAGGTGAATATAGCTGCCCcgC	139675	139697	23	4
BART5-5P	BART5	CAAGGTGAATATAGCTGCCCcTC	139675	139697	23	10
BART5-5P	BART5	CAAGGTGAATATAGCTGCCCgcC	139675	139697	23	4
BART5-5P	BART5	CAAGGTGAATATAGCTGCCCgTC	139675	139697	23	3
BART5-5P	BART5	CAAGGTGAATATAGCTGCCCtat	139675	139697	23	9
BART5-5P	BART5	CAAGGTGAATATAGCTGCCCtgC	139675	139697	23	7
BART5-5P	BART5	CAAGGTGAATATAGCTGCCCtTC	139675	139697	23	9
BART5-5P	BART5	CAAGGTGAATATAGCTGCCgATt	139675	139697	23	3
BART5-5P	BART5	CAAGGTGAATATAGCTGCCtATC	139675	139697	23	4
BART5-5P	BART5	CAAGGTGAATATAGCTGCgCAcC	139675	139697	23	4
BART5-5P	BART5	CAAGGTGAATATAGCTGCgCATC	139675	139697	23	23
BART5-5P	BART5	CAAGGTGAATATAGCTGCtCATa	139675	139697	23	4
BART5-5P	BART5	CAAGGTGAATATAGCTGCtCATC	139675	139697	23	8
BART5-5P	BART5	CAAGGTGAATATAGCTGgCCATC	139675	139697	23	5
BART5-5P	BART5	CAAGGTGAATATAGCTGtCCAcC	139675	139697	23	14
BART5-5P	BART5	CAAGGTGAATATAGCTGtCCATa	139675	139697	23	10
BART5-5P	BART5	CAAGGTGAATATAGCTGtCCATC	139675	139697	23	48
BART5-5P	BART5	CAAGGTGAATATAGCTGtCCATg	139675	139697	23	3
BART5-5P	BART5	CAAGGTGAATATAtCTGCCCATC	139675	139697	23	5
BART5-5P	BART5	tAAGGTGAATATAGCTGCCCATa	139675	139697	23	3
BART5-5P	BART5	tAAGGTGAATATAGCTGCCCATC	139675	139697	23	7
BART5-5P	BART5	CAAGGTGAATATAGCTGCaCAcCG	139675	139698	24	8
BART5-5P	BART5	CAAGGTGAATATAGCTGCaCATCG	139675	139698	24	160
BART5-5P	BART5	CAAGGTGAATATAGCTGCCaATCG	139675	139698	24	8
BART5-5P	BART5	CAAGGTGAATATAGCTGCCCAaCa	139675	139698	24	3
BART5-5P	BART5	CAAGGTGAATATAGCTGCCCAaCG	139675	139698	24	45
BART5-5P	BART5	CAAGGTGAATATAGCTGCCCAcac	139675	139698	24	29
BART5-5P	BART5	CAAGGTGAATATAGCTGCCCAcCa	139675	139698	24	8
BART5-5P	BART5	CAAGGTGAATATAGCTGCCCAcCG	139675	139698	24	299
BART5-5P	BART5	CAAGGTGAATATAGCTGCCCAcgc	139675	139698	24	6
BART5-5P	BART5	CAAGGTGAATATAGCTGCCCAcgG	139675	139698	24	29
BART5-5P	BART5	CAAGGTGAATATAGCTGCCCActG	139675	139698	24	7
BART5-5P	BART5	CAAGGTGAATATAGCTGCCCAgCa	139675	139698	24	7
BART5-5P	BART5	CAAGGTGAATATAGCTGCCCAgCG	139675	139698	24	30
BART5-5P	BART5	CAAGGTGAATATAGCTGCCCAgCt	139675	139698	24	5

BART5-5P	BART5	CAAGGTGAATATAGCTGCCCATaa	139675	139698	24	12
BART5-5P	BART5	CAAGGTGAATATAGCTGCCCATac	139675	139698	24	23
BART5-5P	BART5	CAAGGTGAATATAGCTGCCCATaG	139675	139698	24	17
BART5-5P	BART5	CAAGGTGAATATAGCTGCCCATat	139675	139698	24	9
BART5-5P	BART5	CAAGGTGAATATAGCTGCCCATCa	139675	139698	24	129
BART5-5P	BART5	CAAGGTGAATATAGCTGCCCATCc	139675	139698	24	43
BART5-5P	BART5	CAAGGTGAATATAGCTGCCCATCG	139675	139698	24	2601
BART5-5P	BART5	CAAGGTGAATATAGCTGCCCATCt	139675	139698	24	17
BART5-5P	BART5	CAAGGTGAATATAGCTGCCCATga	139675	139698	24	11
BART5-5P	BART5	CAAGGTGAATATAGCTGCCCATgG	139675	139698	24	11
BART5-5P	BART5	CAAGGTGAATATAGCTGCCCATgt	139675	139698	24	5
BART5-5P	BART5	CAAGGTGAATATAGCTGCCCATtG	139675	139698	24	6
BART5-5P	BART5	CAAGGTGAATATAGCTGCCCcTCG	139675	139698	24	18
BART5-5P	BART5	CAAGGTGAATATAGCTGCCCgaCG	139675	139698	24	5
BART5-5P	BART5	CAAGGTGAATATAGCTGCCCgTCG	139675	139698	24	7
BART5-5P	BART5	CAAGGTGAATATAGCTGCCCtTCG	139675	139698	24	5
BART5-5P	BART5	CAAGGTGAATATAGCTGCCgATCG	139675	139698	24	11
BART5-5P	BART5	CAAGGTGAATATAGCTGCCgtgCG	139675	139698	24	3
BART5-5P	BART5	CAAGGTGAATATAGCTGCCtATCG	139675	139698	24	14
BART5-5P	BART5	CAAGGTGAATATAGCTGCCtgTCG	139675	139698	24	3
BART5-5P	BART5	CAAGGTGAATATAGCTGCgCAcCG	139675	139698	24	10
BART5-5P	BART5	CAAGGTGAATATAGCTGCgCATCa	139675	139698	24	7
BART5-5P	BART5	CAAGGTGAATATAGCTGCgCATCG	139675	139698	24	77
BART5-5P	BART5	CAAGGTGAATATAGCTGCtaATCG	139675	139698	24	11
BART5-5P	BART5	CAAGGTGAATATAGCTGCtCATaG	139675	139698	24	3
BART5-5P	BART5	CAAGGTGAATATAGCTGCtCATCG	139675	139698	24	42
BART5-5P	BART5	CAAGGTGAATATAGCTGtCCATCG	139675	139698	24	3
BART5-5P	BART5	CAAGGTGAATgTAGCTGCCCATCG	139675	139698	24	3
BART5-5P	BART5	CAAGGTGAcTATAGCTGCCCATCG	139675	139698	24	6
BART5-5P	BART5	CAAGGTGAgTATAGCTGCCCATCG	139675	139698	24	3
BART5-5P	BART5	CAgGGTGAATATAGCTGCCCATCG	139675	139698	24	7
BART5-5P	BART5	CgAGGTGAATATAGCTGCCCATCG	139675	139698	24	9
BART5-5P	BART5	CgAGGTGAATATAGCTGCCCcTCG	139675	139698	24	3
BART5-5P	BART5	gAAGGTGAATATAGCTGCCCATCG	139675	139698	24	6
BART5-5P	BART5	tAAGGTGAATATAGCTGCCCATCG	139675	139698	24	21
BART5-5P	BART5	agAGGTGAATATAGCTGCCCATCGA	139675	139699	25	5
BART5-5P	BART5	CAAGGTGAAcATAGCTGCCCATCat	139675	139699	25	3
BART5-5P	BART5	CAAGGTGAATATAGCTGCCaAcCGA	139675	139699	25	3
BART5-5P	BART5	CAAGGTGAATATAGCTGCCaATCGA	139675	139699	25	22
BART5-5P	BART5	CAAGGTGAATATAGCTGCCaATCGt	139675	139699	25	25
BART5-5P	BART5	CAAGGTGAATATAGCTGCCCAaCGA	139675	139699	25	5
BART5-5P	BART5	CAAGGTGAATATAGCTGCCCAaCGc	139675	139699	25	7
BART5-5P	BART5	CAAGGTGAATATAGCTGCCCAaCGt	139675	139699	25	14
BART5-5P	BART5	CAAGGTGAATATAGCTGCCCAatcA	139675	139699	25	4
BART5-5P	BART5	CAAGGTGAATATAGCTGCCCAcCaA	139675	139699	25	3
BART5-5P	BART5	CAAGGTGAATATAGCTGCCCAcCat	139675	139699	25	3
BART5-5P	BART5	CAAGGTGAATATAGCTGCCCAcCcA	139675	139699	25	4
BART5-5P	BART5	CAAGGTGAATATAGCTGCCCAcCcc	139675	139699	25	17
BART5-5P	BART5	CAAGGTGAATATAGCTGCCCAcCGA	139675	139699	25	74
BART5-5P	BART5	CAAGGTGAATATAGCTGCCCAcCGc	139675	139699	25	37
BART5-5P	BART5	CAAGGTGAATATAGCTGCCCAcCGg	139675	139699	25	7
BART5-5P	BART5	CAAGGTGAATATAGCTGCCCAcCGt	139675	139699	25	144
BART5-5P	BART5	CAAGGTGAATATAGCTGCCCAcCtt	139675	139699	25	7
BART5-5P	BART5	CAAGGTGAATATAGCTGCCCAcgcA	139675	139699	25	7
BART5-5P	BART5	CAAGGTGAATATAGCTGCCCActcA	139675	139699	25	4
BART5-5P	BART5	CAAGGTGAATATAGCTGCCCAgCcA	139675	139699	25	3
BART5-5P	BART5	CAAGGTGAATATAGCTGCCCAgCGA	139675	139699	25	5
BART5-5P	BART5	CAAGGTGAATATAGCTGCCCATacA	139675	139699	25	29
BART5-5P	BART5	CAAGGTGAATATAGCTGCCCATacg	139675	139699	25	10
BART5-5P	BART5	CAAGGTGAATATAGCTGCCCATaGA	139675	139699	25	14
BART5-5P	BART5	CAAGGTGAATATAGCTGCCCATaGt	139675	139699	25	3
BART5-5P	BART5	CAAGGTGAATATAGCTGCCCATatA	139675	139699	25	5
BART5-5P	BART5	CAAGGTGAATATAGCTGCCCATCaA	139675	139699	25	47
BART5-5P	BART5	CAAGGTGAATATAGCTGCCCATCac	139675	139699	25	4
BART5-5P	BART5	CAAGGTGAATATAGCTGCCCATCag	139675	139699	25	4
BART5-5P	BART5	CAAGGTGAATATAGCTGCCCATCat	139675	139699	25	72
BART5-5P	BART5	CAAGGTGAATATAGCTGCCCATCcA	139675	139699	25	13
BART5-5P	BART5	CAAGGTGAATATAGCTGCCCATCcc	139675	139699	25	9

BART5-5P	BART5	CAAGGTGAATATAGCTGCCCATCcg	139675	139699	25	8
BART5-5P	BART5	CAAGGTGAATATAGCTGCCCATCGA	139675	139699	25	1027
BART5-5P	BART5	CAAGGTGAATATAGCTGCCCATCGc	139675	139699	25	148
BART5-5P	BART5	CAAGGTGAATATAGCTGCCCATCGg	139675	139699	25	23
BART5-5P	BART5	CAAGGTGAATATAGCTGCCCATCGt	139675	139699	25	84
BART5-5P	BART5	CAAGGTGAATATAGCTGCCCATCtA	139675	139699	25	16
BART5-5P	BART5	CAAGGTGAATATAGCTGCCCATCtg	139675	139699	25	13
BART5-5P	BART5	CAAGGTGAATATAGCTGCCCATgcA	139675	139699	25	25
BART5-5P	BART5	CAAGGTGAATATAGCTGCCCATgGt	139675	139699	25	3
BART5-5P	BART5	CAAGGTGAATATAGCTGCCCATtcA	139675	139699	25	15
BART5-5P	BART5	CAAGGTGAATATAGCTGCCCATtcg	139675	139699	25	5
BART5-5P	BART5	CAAGGTGAATATAGCTGCCCATtGA	139675	139699	25	4
BART5-5P	BART5	CAAGGTGAATATAGCTGCCCATtGg	139675	139699	25	3
BART5-5P	BART5	CAAGGTGAATATAGCTGCCCATtGt	139675	139699	25	8
BART5-5P	BART5	CAAGGTGAATATAGCTGCCCcTCGA	139675	139699	25	15
BART5-5P	BART5	CAAGGTGAATATAGCTGCCCgaCGA	139675	139699	25	8
BART5-5P	BART5	CAAGGTGAATATAGCTGCCCgcCGA	139675	139699	25	4
BART5-5P	BART5	CAAGGTGAATATAGCTGCCCgTCGt	139675	139699	25	3
BART5-5P	BART5	CAAGGTGAATATAGCTGCCgATCGA	139675	139699	25	32
BART5-5P	BART5	CAAGGTGAATATAGCTGCCgATCGg	139675	139699	25	3
BART5-5P	BART5	CAAGGTGAATATAGCTGCCgATCGt	139675	139699	25	6
BART5-5P	BART5	CAAGGTGAATATAGCTGCCtATCGA	139675	139699	25	20
BART5-5P	BART5	CAAGGTGAATATAGCTGCCtATCGt	139675	139699	25	7
BART5-5P	BART5	CAtGGTGAATATAGCTGCCCAaCGt	139675	139699	25	3
BART5-5P	BART5	CgAGGTGAATATAGCTGCCCATCGc	139675	139699	25	3
BART5-5P	BART5	CgAGGTGAATATAGCTGCCCATCGt	139675	139699	25	3
BART5-5P	BART5	tAAGGTGAATATAGCTGCCCATCGA	139675	139699	25	12
BART5-5P	BART5	CAAGGTGAATATAGCTGCCCAaCGca	139675	139700	26	4
BART5-5P	BART5	CAAGGTGAATATAGCTGCCCAcCGAC	139675	139700	26	4
BART5-5P	BART5	CAAGGTGAATATAGCTGCCCAgCtAC	139675	139700	26	7
BART5-5P	BART5	CAAGGTGAATATAGCTGCCCATaGca	139675	139700	26	6
BART5-5P	BART5	CAAGGTGAATATAGCTGCCCATaGcg	139675	139700	26	4
BART5-5P	BART5	CAAGGTGAATATAGCTGCCCATaGct	139675	139700	26	28
BART5-5P	BART5	CAAGGTGAATATAGCTGCCCATatgC	139675	139700	26	6
BART5-5P	BART5	CAAGGTGAATATAGCTGCCCATCaAC	139675	139700	26	5
BART5-5P	BART5	CAAGGTGAATATAGCTGCCCATCacC	139675	139700	26	6
BART5-5P	BART5	CAAGGTGAATATAGCTGCCCATCGAa	139675	139700	26	6
BART5-5P	BART5	CAAGGTGAATATAGCTGCCCATCGAC	139675	139700	26	20
BART5-5P	BART5	CAAGGTGAATATAGCTGCCCATCGAt	139675	139700	26	27
BART5-5P	BART5	CAAGGTGAATATAGCTGCCCATCGca	139675	139700	26	12
BART5-5P	BART5	CAAGGTGAATATAGCTGCCCATCGct	139675	139700	26	5
BART5-5P	BART5	CAAGGTGAATATAGCTGCCCATCGta	139675	139700	26	6
BART5-5P	BART5	CAAGGTGAATATAGCTGCCCATCGtC	139675	139700	26	7
BART5-5P	BART5	CAAGGTGAATATAGCTGCCCATCtAC	139675	139700	26	12
BART5-5P	BART5	CAAGGTGAATATAGCTGCCCATgtAC	139675	139700	26	6
BART5-5P	BART5	CAAGGTGAATATAGCTGCCCAcCGACG	139675	139701	27	4
BART5-5P	BART5	CAAGGTGAATATAGCTGCCCATCGACG	139675	139701	27	4
BART5-5P	BART5	CAAGGTGAATATAGCTGCCCATCGcaG	139675	139701	27	4
BART5-5P	BART5	AAGGTGAATATAGCTGCCC	139676	139694	19	13
BART5-5P	BART5	AAGGTGAATATAGCaGCCCA	139676	139695	20	14
BART5-5P	BART5	AAGGTGAATATAGCTaCCCA	139676	139695	20	8
BART5-5P	BART5	AAGGTGAATATAGCTcCCCt	139676	139695	20	8
BART5-5P	BART5	AAGGTGAATATAGCTGCCCA	139676	139695	20	46
BART5-5P	BART5	AAGGTGAATATAGCTaaCCAT	139676	139696	21	13
BART5-5P	BART5	AAGGTGAATATAGCTaCCCAa	139676	139696	21	6
BART5-5P	BART5	AAGGTGAATATAGCTaCCCAc	139676	139696	21	12
BART5-5P	BART5	AAGGTGAATATAGCTaCCCAT	139676	139696	21	111
BART5-5P	BART5	AAGGTGAATATAGCTGaCCAT	139676	139696	21	6
BART5-5P	BART5	AAGGTGAATATAGCTGaCCca	139676	139696	21	4
BART5-5P	BART5	AAGGTGAATATAGCTGatCAT	139676	139696	21	3
BART5-5P	BART5	AAGGTGAATATAGCTGCatAa	139676	139696	21	6
BART5-5P	BART5	AAGGTGAATATAGCTGCCaAT	139676	139696	21	6
BART5-5P	BART5	AAGGTGAATATAGCTGCCacT	139676	139696	21	9
BART5-5P	BART5	AAGGTGAATATAGCTGCCata	139676	139696	21	13
BART5-5P	BART5	AAGGTGAATATAGCTGCCatc	139676	139696	21	11
BART5-5P	BART5	AAGGTGAATATAGCTGCCatT	139676	139696	21	3
BART5-5P	BART5	AAGGTGAATATAGCTGCCCAa	139676	139696	21	35
BART5-5P	BART5	AAGGTGAATATAGCTGCCCAc	139676	139696	21	63

BART5-5P	BART5	AAGGTGAATATAGCTGCCCAT	139676	139696	21	260
BART5-5P	BART5	AAGGTGAATATAGCTGCCCcT	139676	139696	21	7
BART5-5P	BART5	AAGGTGAATATAGCTGCCCgT	139676	139696	21	6
BART5-5P	BART5	AAGGTGAATATAGCTGCCgAc	139676	139696	21	4
BART5-5P	BART5	AAGGTGAATATAGCTGCCgAT	139676	139696	21	3
BART5-5P	BART5	AAGGTGAATATAGCTGCCgca	139676	139696	21	6
BART5-5P	BART5	AAGGTGAATATAGCTGCCtAT	139676	139696	21	5
BART5-5P	BART5	AAGGTGAATATAGCTGtCCAT	139676	139696	21	5
BART5-5P	BART5	AAGGTGAATATAGCTtCCCAT	139676	139696	21	6
BART5-5P	BART5	gAGGTGAATATAGCTGCCCAT	139676	139696	21	11
BART5-5P	BART5	AAGGTGAATATAGCTGaCCAcC	139676	139697	22	21
BART5-5P	BART5	AAGGTGAATATAGCTGaCCATa	139676	139697	22	6
BART5-5P	BART5	AAGGTGAATATAGCTGaCCATC	139676	139697	22	38
BART5-5P	BART5	AAGGTGAATATAGCTGCaCATa	139676	139697	22	5
BART5-5P	BART5	AAGGTGAATATAGCTGCaCATC	139676	139697	22	6
BART5-5P	BART5	AAGGTGAATATAGCTGCCCAaC	139676	139697	22	10
BART5-5P	BART5	AAGGTGAATATAGCTGCCCAcC	139676	139697	22	44
BART5-5P	BART5	AAGGTGAATATAGCTGCCCAgC	139676	139697	22	5
BART5-5P	BART5	AAGGTGAATATAGCTGCCCATa	139676	139697	22	64
BART5-5P	BART5	AAGGTGAATATAGCTGCCCATC	139676	139697	22	386
BART5-5P	BART5	AAGGTGAATATAGCTGCCCATg	139676	139697	22	12
BART5-5P	BART5	AAGGTGAATATAGCTGCCCATt	139676	139697	22	40
BART5-5P	BART5	AAGGTGAATATAGCTGCCCcgg	139676	139697	22	4
BART5-5P	BART5	AAGGTGAATATAGCTGCCCcTC	139676	139697	22	3
BART5-5P	BART5	AAGGTGAATATAGCTGCCCcTt	139676	139697	22	9
BART5-5P	BART5	AAGGTGAATATAGCTGCCCgTC	139676	139697	22	4
BART5-5P	BART5	AAGGTGAATATAGCTGCCCtag	139676	139697	22	3
BART5-5P	BART5	AAGGTGAATATAGCTGCCCtTC	139676	139697	22	16
BART5-5P	BART5	AAGGTGAATATAGCTGCgCATC	139676	139697	22	3
BART5-5P	BART5	AAGGTGAATATAGCTGCtCAcC	139676	139697	22	6
BART5-5P	BART5	AAGGTGAATATAGCTGtCCATa	139676	139697	22	6
BART5-5P	BART5	AAGGTGAATATAGCTGtCCATC	139676	139697	22	13
BART5-5P	BART5	AAGGTGAATATAGCTGttCATC	139676	139697	22	3
BART5-5P	BART5	AAtGTGAATATAGCTGCCCATa	139676	139697	22	12
BART5-5P	BART5	cAGGTGAATATAGCTGCCCATC	139676	139697	22	3
BART5-5P	BART5	AAGGTGAATATAGCTGCaCAcCG	139676	139698	23	39
BART5-5P	BART5	AAGGTGAATATAGCTGCaCATCc	139676	139698	23	4
BART5-5P	BART5	AAGGTGAATATAGCTGCaCATCG	139676	139698	23	45
BART5-5P	BART5	AAGGTGAATATAGCTGCCCAaCG	139676	139698	23	8
BART5-5P	BART5	AAGGTGAATATAGCTGCCCAcCa	139676	139698	23	8
BART5-5P	BART5	AAGGTGAATATAGCTGCCCAcCc	139676	139698	23	4
BART5-5P	BART5	AAGGTGAATATAGCTGCCCAcCG	139676	139698	23	62
BART5-5P	BART5	AAGGTGAATATAGCTGCCCAcCt	139676	139698	23	4
BART5-5P	BART5	AAGGTGAATATAGCTGCCCAcgG	139676	139698	23	3
BART5-5P	BART5	AAGGTGAATATAGCTGCCCAgCG	139676	139698	23	6
BART5-5P	BART5	AAGGTGAATATAGCTGCCCAgCt	139676	139698	23	6
BART5-5P	BART5	AAGGTGAATATAGCTGCCCATaa	139676	139698	23	3
BART5-5P	BART5	AAGGTGAATATAGCTGCCCATac	139676	139698	23	12
BART5-5P	BART5	AAGGTGAATATAGCTGCCCATaG	139676	139698	23	16
BART5-5P	BART5	AAGGTGAATATAGCTGCCCATCa	139676	139698	23	22
BART5-5P	BART5	AAGGTGAATATAGCTGCCCATCc	139676	139698	23	39
BART5-5P	BART5	AAGGTGAATATAGCTGCCCATCG	139676	139698	23	791
BART5-5P	BART5	AAGGTGAATATAGCTGCCCATCt	139676	139698	23	8
BART5-5P	BART5	AAGGTGAATATAGCTGCCCATga	139676	139698	23	4
BART5-5P	BART5	AAGGTGAATATAGCTGCCCATgG	139676	139698	23	6
BART5-5P	BART5	AAGGTGAATATAGCTGCCCATgt	139676	139698	23	3
BART5-5P	BART5	AAGGTGAATATAGCTGCCCATtG	139676	139698	23	7
BART5-5P	BART5	AAGGTGAATATAGCTGCCCcgaG	139676	139698	23	14
BART5-5P	BART5	AAGGTGAATATAGCTGCCCcTCG	139676	139698	23	8
BART5-5P	BART5	AAGGTGAATATAGCTGCCCtcCG	139676	139698	23	3
BART5-5P	BART5	AAGGTGAATATAGCTGCCgATCc	139676	139698	23	3
BART5-5P	BART5	AAGGTGAATATAGCTGCCtATCG	139676	139698	23	4
BART5-5P	BART5	AAGGTGAATATAGCTGCgCATCG	139676	139698	23	19
BART5-5P	BART5	AAGGTGAATATAGCTGCtCATCG	139676	139698	23	28
BART5-5P	BART5	AgaGTGAATATAGCTGCCCATCG	139676	139698	23	8
BART5-5P	BART5	cAGGTGAATATAGCTGCCCATCG	139676	139698	23	12
BART5-5P	BART5	tAGGTGAATATAGCTGCCCATCG	139676	139698	23	7
BART5-5P	BART5	AAGGTGAATATAGCTGCCaATCGA	139676	139699	24	22

BART5-5P	BART5	AAGGTGAATATAGCTGCCCAaCGA	139676	139699	24	13
BART5-5P	BART5	AAGGTGAATATAGCTGCCCAaCGt	139676	139699	24	11
BART5-5P	BART5	AAGGTGAATATAGCTGCCCAcCat	139676	139699	24	3
BART5-5P	BART5	AAGGTGAATATAGCTGCCCAcCGA	139676	139699	24	21
BART5-5P	BART5	AAGGTGAATATAGCTGCCCAcCGc	139676	139699	24	5
BART5-5P	BART5	AAGGTGAATATAGCTGCCCAcCGt	139676	139699	24	6
BART5-5P	BART5	AAGGTGAATATAGCTGCCCAgCcA	139676	139699	24	5
BART5-5P	BART5	AAGGTGAATATAGCTGCCCAgCGA	139676	139699	24	4
BART5-5P	BART5	AAGGTGAATATAGCTGCCCATacA	139676	139699	24	11
BART5-5P	BART5	AAGGTGAATATAGCTGCCCATacg	139676	139699	24	7
BART5-5P	BART5	AAGGTGAATATAGCTGCCCATCaA	139676	139699	24	29
BART5-5P	BART5	AAGGTGAATATAGCTGCCCATCat	139676	139699	24	9
BART5-5P	BART5	AAGGTGAATATAGCTGCCCATCcA	139676	139699	24	9
BART5-5P	BART5	AAGGTGAATATAGCTGCCCATCct	139676	139699	24	3
BART5-5P	BART5	AAGGTGAATATAGCTGCCCATCGA	139676	139699	24	651
BART5-5P	BART5	AAGGTGAATATAGCTGCCCATCGc	139676	139699	24	49
BART5-5P	BART5	AAGGTGAATATAGCTGCCCATCGg	139676	139699	24	3
BART5-5P	BART5	AAGGTGAATATAGCTGCCCATCGt	139676	139699	24	29
BART5-5P	BART5	AAGGTGAATATAGCTGCCCATCtg	139676	139699	24	8
BART5-5P	BART5	AAGGTGAATATAGCTGCCCATgcA	139676	139699	24	4
BART5-5P	BART5	AAGGTGAATATAGCTGCCCATtcA	139676	139699	24	7
BART5-5P	BART5	AAGGTGAATATAGCTGCCCATtct	139676	139699	24	3
BART5-5P	BART5	AAGGTGAATATAGCTGCCCATtGA	139676	139699	24	5
BART5-5P	BART5	AAGGTGAATATAGCTGCCCcTCGA	139676	139699	24	5
BART5-5P	BART5	AAGGTGAATATAGCTGCCCgaaGA	139676	139699	24	4
BART5-5P	BART5	AAGGTGAATATAGCTGCCCtaCGA	139676	139699	24	3
BART5-5P	BART5	AAGGTGAATATAGCTGCCgATCGA	139676	139699	24	21
BART5-5P	BART5	AAGGTGAATATAGCTGCCtATCGA	139676	139699	24	27
BART5-5P	BART5	AgGGTGAATATAGCTGCCCATCGA	139676	139699	24	4
BART5-5P	BART5	gAGGTGAATATAGCTGCCCATCGA	139676	139699	24	6
BART5-5P	BART5	tAGGTGAATATAGCTGCCCATCGA	139676	139699	24	8
BART5-5P	BART5	AAGGTGAATATAGCTGCCCAcCGct	139676	139700	25	3
BART5-5P	BART5	AAGGTGAATATAGCTGCCCATaGct	139676	139700	25	6
BART5-5P	BART5	AAGGTGAATATAGCTGCCCATCGAa	139676	139700	25	3
BART5-5P	BART5	AAGGTGAATATAGCTGCCCATCGAC	139676	139700	25	23
BART5-5P	BART5	AAGGTGAATATAGCTGCCCATCGAt	139676	139700	25	6
BART5-5P	BART5	AAGGTGAATATAGCTGCCCATCGct	139676	139700	25	13
BART5-5P	BART5	AAGGTGAATATAGCTGCCCgaCGAC	139676	139700	25	3
BART5-5P	BART5	AAGGTGAATATAGCTGCCCATCGACG	139676	139701	26	3
BART5-5P	BART5	AAGGTGAATATAGCTGCCCATCGAaGc	139676	139702	27	5
BART5-5P	BART5	AGGTGAATATAGCTGCCCAcCGA	139677	139699	23	3
BART5-5P	BART5	AGGTGAATATAGCTGCCCATCGA	139677	139699	23	11
BART5-3P	BART5-star	GTGGGCCGCTGTTCACCTA	139717	139735	19	8
BART5-3P	BART5-star	GTGGGCCGCTGTTCACCaAt	139717	139736	20	3
BART5-3P	BART5-star	GTGGGCCGCTGTTCACCTAA	139717	139736	20	110
BART5-3P	BART5-star	GTGGGCCGCTGTTCACCTAt	139717	139736	20	19
BART5-3P	BART5-star	GTGGGCCGCTGTTCACgcAA	139717	139736	20	4
BART5-3P	BART5-star	GTGGGCCGCTGTTCcCCTAA	139717	139736	20	13
BART5-3P	BART5-star	GTGGGCCGCTGTTCACCcAAA	139717	139737	21	6
BART5-3P	BART5-star	GTGGGCCGCTGTTCACCctAA	139717	139737	21	6
BART5-3P	BART5-star	GTGGGCCGCTGTTCACCTAAA	139717	139737	21	46
BART5-3P	BART5-star	GTGGGCCGCTGTTCACCTAAAa	139717	139738	22	7
BART5-3P	BART5-star	GTGGGCCGCTGTTCACCTAAAt	139717	139738	22	17
BART5-3P	BART5-star	GTGGGCCGCTGTTCACCTAAtc	139717	139738	22	27
BART5-3P	BART5-star	GTGGGCCGCTGTTCACCTAtAa	139717	139738	22	10
BART5-3P	BART5-star	TGGGCCGCTGTTCACCTAA	139718	139736	19	9
BART5-3P	BART5-star	TGGGCCGCTGTTCACCTAAA	139718	139737	20	4
BART5-3P	BART5-star	TGGGCCGCTGTTCACCTAAAt	139718	139738	21	3
BART5-3P	BART5-star	TGGGCCGCTGTTCACCTAAct	139718	139738	21	4
BART5-3P	BART5-star	TGGGCCGCTGTTCACCTAAAtTG	139718	139740	23	4
BART16-5P	BART16	TTAGATAGAGTGGGTGTtT	139795	139813	19	8
BART16-5P	BART16	TTAGATAGAGTGGGTGTGTa	139795	139814	20	3
BART16-5P	BART16	TTAGATAGAGTGGGcGTGTGC	139795	139815	21	6
BART16-5P	BART16	TTAGATAGAGTGGGTaTGcGC	139795	139815	21	4
BART16-5P	BART16	TTAGATAGAGTGGGTaTGTGC	139795	139815	21	4
BART16-5P	BART16	TTAGATAGAGTGGGTGgGTGC	139795	139815	21	12
BART16-5P	BART16	TTAGATAGAGTGGGTGTcTGC	139795	139815	21	4
BART16-5P	BART16	TTAGATAGAGTGGGTGTGTGC	139795	139815	21	23

BART16-5P	BART16	TTAGATAGAGTGGGTGcGTGgc	139795	139816	22	5
BART16-5P	BART16	TTAGATAGAGTGGGTGTGTaCT	139795	139816	22	4
BART16-5P	BART16	TTAGATAGAGTGGGTGTGTGCT	139795	139816	22	5
BART16-5P	BART16	TTAGATAGAGTGGGTGTGTaCTg	139795	139817	23	7
BART16-5P	BART16	TTAGATAGAGTGGGTGTGTGCTC	139795	139817	23	9
BART16-5P	BART16	TTAGATAGAGTGGGTGTGTGCTt	139795	139817	23	3
BART16-5P	BART16	TTAGATAGAGTGGGTGTGTaCcCTT	139795	139819	25	6
BART16-5P	BART16	TTAGATAGAGTGGGTGTGTGCTCTTaTT	139795	139822	28	18
BART16-3P		AGATCACCACCCTCTATCC	139834	139852	19	8
BART16-3P		AGATCACCACCCTCaATCCA	139834	139853	20	3
BART16-3P		AGATCACCACCCTCgATCCA	139834	139853	20	5
BART16-3P		AGATCACCACCCTCTAcCCA	139834	139853	20	12
BART16-3P		AGATCACCACCCTCTATCCA	139834	139853	20	15
BART16-3P		AcATCACCACCCTCTATCCAT	139834	139854	21	3
BART16-3P		AGATCACCACCCTCTAcCCAT	139834	139854	21	6
BART16-3P		AGATCACCACCCTCTAgCCAT	139834	139854	21	15
BART16-3P		AGATCACCACCCTCTATCCAT	139834	139854	21	120
BART16-3P		AGATCACCACCCTCTcTCCAT	139834	139854	21	21
BART16-3P		AGATCACCACCCTCTtTCCAT	139834	139854	21	3
BART16-3P		AGATCACCACCCTCTAcCCATA	139834	139855	22	6
BART16-3P		AGATCACCACCCTCTATCCATA	139834	139855	22	22
BART16-3P		AGATCACCACCCTCTATCCATc	139834	139855	22	3
BART16-3P		AGATCACCACCCTCTATCCATt	139834	139855	22	16
BART16-3P		AGATCACCACCCTCTATCCATAc	139834	139856	23	3
BART16-3P		AGATCACCACCCTCTATCCATAT	139834	139856	23	72
BART16-3P		AGATCACCACCCTCTATCCATgT	139834	139856	23	7
BART16-3P		AGATCACCACCCTCTATCCATta	139834	139856	23	3
BART16-3P		GATCACCACCCTCTATCCAT	139835	139854	20	14
BART16-3P		GATCACCACCCTCTATCCAcA	139835	139855	21	5
BART16-3P		GATCACCACCCTCTATCCATAa	139835	139856	22	3
BART16-3P		GATCACCACCCTCTATCCATAT	139835	139856	22	64
BART16-3P		GATCACCACCCTCTATCCtcAT	139835	139856	22	6
BART16-3P		GATCACCACCCTCTATCCAgAat	139835	139857	23	4
BART16-3P		ATCACCACCCTCTATCCAT	139836	139854	19	9
BART16-3P		ATCACCACCCTCTATCCtT	139836	139854	19	6
BART16-3P		ATCACCACCCTCTcTCCAT	139836	139854	19	3
BART16-3P		ATCACCACCCTCTATCCATAa	139836	139856	21	6
BART16-3P		ATCACCACCCTCTATCCATAc	139836	139856	21	6
BART16-3P		ATCACCACCCTCTATCCATAT	139836	139856	21	199
BART16-3P		ATCACCACCCTCTATgCATAT	139836	139856	21	3
BART16-3P		gTCACCACCCTCTATCCATAT	139836	139856	21	3
BART16-3P		CCCACAATTGATAAACCTx	139857	139875	19	25
BART16-3P		CCCACAATTGATAAACCTxx	139857	139876	20	6
BART17-5P	BART17-5P	TAAGAGGACGCAGaCATAC	139915	139933	19	15
BART17-5P	BART17-5P	TAAGAGGACGCAGGCAaAC	139915	139933	19	6
BART17-5P	BART17-5P	TAAGAGGACGCAGGCAcAC	139915	139933	19	6
BART17-5P	BART17-5P	TAAGAGGACGCAGGCATAC	139915	139933	19	63
BART17-5P	BART17-5P	TAAGAGGACGCAGGCATtg	139915	139933	19	8
BART17-5P	BART17-5P	TAAGAGGACGCAGGCATACA	139915	139934	20	34
BART17-5P	BART17-5P	TAAGAGGACGCAGGCATtCA	139915	139934	20	8
BART17-5P	BART17-5P	TAAGAGGACGCAGGCATACAA	139915	139935	21	38
BART17-5P	BART17-5P	TAAGAGGACGCAGGCATAtAA	139915	139935	21	5
BART17-5P	BART17-5P	TAAGAGGACGCAGGCATACgAaG	139915	139937	23	4
BART17-5P	BART17-5P	TAAGAGGACGCAGGCATACAAGaT	139915	139938	24	3
BART17-5P	BART17-5P	TAAGAGGACGCAGGCATACAAGGT	139915	139938	24	4
BART17-3P	BART17-3P	TTGTATGCCTGGTGTCCCCTTAGT	139952	139975	24	14
BART17-3P	BART17-3P	TGTATGCCTGGTGTCCCCT	139953	139971	19	9
BART17-3P	BART17-3P	TGTATGCCTGGTGTCCCgT	139953	139971	19	4
BART17-3P	BART17-3P	TGTATGCCTGGTGTCaCCTc	139953	139972	20	4
BART17-3P	BART17-3P	TGTATGCCTGGTGTCCCCaT	139953	139972	20	6
BART17-3P	BART17-3P	TGTATGCCTGGTGTCCCCgT	139953	139972	20	3
BART17-3P	BART17-3P	TGTATGCCTGGTGTCCCCTa	139953	139972	20	6
BART17-3P	BART17-3P	TGTATGCCTGGTGTCCCCTT	139953	139972	20	8
BART17-3P	BART17-3P	TGTATGCCTGGTGTCCCCTaA	139953	139973	21	3
BART17-3P	BART17-3P	TGTATGCCTGGTGTCCCCTTA	139953	139973	21	19
BART17-3P	BART17-3P	TGTATGCCTGGTGTCCCCTTg	139953	139973	21	39
BART17-3P	BART17-3P	TGTATGCCTGGTGTCCCCaTgt	139953	139974	22	4
BART17-3P	BART17-3P	TGTATGCCTGGTGTCCCCcTAG	139953	139974	22	6

BART17-3P	BART17-3P	TGTATGCCTGGTGTCCCCTTAa	139953	139974	22	13
BART17-3P	BART17-3P	TGTATGCCTGGTGTCCCCTTAc	139953	139974	22	8
BART17-3P	BART17-3P	TGTATGCCTGGTGTCCCCTTAG	139953	139974	22	44
BART17-3P	BART17-3P	TGTATGCCTGGTGTCCCCTTgt	139953	139974	22	12
BART17-3P	BART17-3P	aGTATGCCTGGTGTCCCCTTAGT	139953	139975	23	9
BART17-3P	BART17-3P	cGTATGCCTGGTGTCCCCTTAGT	139953	139975	23	5
BART17-3P	BART17-3P	cGTATGCCTGGTGTCCCCTTgGT	139953	139975	23	6
BART17-3P	BART17-3P	TGTATaCCTGGTGTCCCCTTAGT	139953	139975	23	11
BART17-3P	BART17-3P	TGTATGCaTGGTGTCCCCTTAGT	139953	139975	23	5
BART17-3P	BART17-3P	TGTATGCCTGGTGTCCCaTTAGT	139953	139975	23	33
BART17-3P	BART17-3P	TGTATGCCTGGTGTCCCCaacGT	139953	139975	23	6
BART17-3P	BART17-3P	TGTATGCCTGGTGTCCCCacAGT	139953	139975	23	5
BART17-3P	BART17-3P	TGTATGCCTGGTGTCCCCaTAGa	139953	139975	23	6
BART17-3P	BART17-3P	TGTATGCCTGGTGTCCCCgTAGT	139953	139975	23	11
BART17-3P	BART17-3P	TGTATGCCTGGTGTCCCCTacGT	139953	139975	23	3
BART17-3P	BART17-3P	TGTATGCCTGGTGTCCCCTagGT	139953	139975	23	3
BART17-3P	BART17-3P	TGTATGCCTGGTGTCCCCTatGT	139953	139975	23	4
BART17-3P	BART17-3P	TGTATGCCTGGTGTCCCCTctGa	139953	139975	23	3
BART17-3P	BART17-3P	TGTATGCCTGGTGTCCCCTgAGT	139953	139975	23	3
BART17-3P	BART17-3P	TGTATGCCTGGTGTCCCCTTAaa	139953	139975	23	3
BART17-3P	BART17-3P	TGTATGCCTGGTGTCCCCTTAac	139953	139975	23	3
BART17-3P	BART17-3P	TGTATGCCTGGTGTCCCCTTAaT	139953	139975	23	5
BART17-3P	BART17-3P	TGTATGCCTGGTGTCCCCTTAGa	139953	139975	23	39
BART17-3P	BART17-3P	TGTATGCCTGGTGTCCCCTTAGc	139953	139975	23	22
BART17-3P	BART17-3P	TGTATGCCTGGTGTCCCCTTAGT	139953	139975	23	664
BART17-3P	BART17-3P	TGTATGCCTGGTGTCCCCTTAtT	139953	139975	23	4
BART17-3P	BART17-3P	TGTATGCCTGGTGTCCCCTTgGT	139953	139975	23	18
BART17-3P	BART17-3P	TGTATGCCTGGTGTCCCCTTtca	139953	139975	23	7
BART17-3P	BART17-3P	TGTATGCCTGGTGTCCCCTTtGT	139953	139975	23	7
BART17-3P	BART17-3P	TGTATGCCTGGTGTCCCtTTAGT	139953	139975	23	6
BART17-3P	BART17-3P	TGTATGCCTtGTGTCCCCTTAGT	139953	139975	23	3
BART17-3P	BART17-3P	TGTATGCCTGGTGTCCCCaTAGTa	139953	139976	24	11
BART17-3P	BART17-3P	TGTATGCCTGGTGTCCCCaTAGTG	139953	139976	24	10
BART17-3P	BART17-3P	TGTATGCCTGGTGTCCCCcTAGaa	139953	139976	24	4
BART17-3P	BART17-3P	TGTATGCCTGGTGTCCCCcTAGTa	139953	139976	24	5
BART17-3P	BART17-3P	TGTATGCCTGGTGTCCCCTcAGat	139953	139976	24	3
BART17-3P	BART17-3P	TGTATGCCTGGTGTCCCCTTAGaa	139953	139976	24	5
BART17-3P	BART17-3P	TGTATGCCTGGTGTCCCCTTAGac	139953	139976	24	3
BART17-3P	BART17-3P	TGTATGCCTGGTGTCCCCTTAGat	139953	139976	24	11
BART17-3P	BART17-3P	TGTATGCCTGGTGTCCCCTTAGca	139953	139976	24	19
BART17-3P	BART17-3P	TGTATGCCTGGTGTCCCCTTAGgt	139953	139976	24	69
BART17-3P	BART17-3P	TGTATGCCTGGTGTCCCCTTAGTa	139953	139976	24	22
BART17-3P	BART17-3P	TGTATGCCTGGTGTCCCCTTAGTc	139953	139976	24	8
BART17-3P	BART17-3P	TGTATGCCTGGTGTCCCCTTAGTG	139953	139976	24	71
BART17-3P	BART17-3P	TGTATGCCTGGTGTCCCCTTAGTt	139953	139976	24	26
BART17-3P	BART17-3P	TGTATGCCTGGTGTCCCCTTAtca	139953	139976	24	17
BART17-3P	BART17-3P	TGTATGCCTGGTGTCCCCTTgGTc	139953	139976	24	4
BART17-3P	BART17-3P	TGTATGCCTGGTGTCCCCTaAGTGG	139953	139977	25	3
BART17-3P	BART17-3P	TGTATGCCTGGTGTCCCCTcAGTGc	139953	139977	25	3
BART17-3P	BART17-3P	TGTATGCCTGGTGTCCCCTcAGTGG	139953	139977	25	13
BART17-3P	BART17-3P	TGTATGCCTGGTGTCCCCTTAGaaG	139953	139977	25	25
BART17-3P	BART17-3P	TGTATGCCTGGTGTCCCCTTAGTGc	139953	139977	25	21
BART17-3P	BART17-3P	TGTATGCCTGGTGTCCCCTTAGTGG	139953	139977	25	21
BART17-3P	BART17-3P	TGTATGCCTGGTGTCCCCTTAGTGGG	139953	139978	26	6
BART17-3P	BART17-3P	TGTATGCCTGGTGTCCCCTTAGTGtt	139953	139978	26	4
BART17-3P	BART17-3P	GTATGCCcGGTGTCCCCTTAGT	139954	139975	22	3
BART17-3P	BART17-3P	GTATGCCTGGTGTCCCCTTAGT	139954	139975	22	57
BART17-3P	BART17-3P	GTATGCCTGGTGTCCCCaTAGTa	139954	139976	23	4
BART17-3P	BART17-3P	GTATGCCTGGTGTCCCCTTAGTa	139954	139976	23	3
BART17-3P	BART17-3P	GTATGCCTGGTGTCCCCTTAGTt	139954	139976	23	9
BART17-3P	BART17-3P	TATGCCTGGTGTCCCCTTAG	139955	139974	20	3
BART17-3P	BART17-3P	TATGCCTGGTGTCCCCTTAGT	139955	139975	21	5
BART17-3P	BART17-3P	TATGCCTGGTGTCCCCTTAGct	139955	139976	22	4
BART6-5P	BART6-5P	TAAGGTTGGTCCAATCCATA	140033	140052	20	6
BART6-5P	BART6-5P	TAAGGTTGGTCCAATaCATAG	140033	140053	21	42
BART6-5P	BART6-5P	TAAGGTTGGTCCAATCCAcAG	140033	140053	21	6
BART6-5P	BART6-5P	TAAGGTTGGTCCAATCCATAa	140033	140053	21	5
BART6-5P	BART6-5P	TAAGGTTGGTCCAATCCATAG	140033	140053	21	83

BART6-5P	BART6-5P	TAAGGTTGGTCCAATgCATAG	140033	140053	21	6
BART6-5P	BART6-5P	TAAGGTTGGTCCAATtCATAa	140033	140053	21	3
BART6-5P	BART6-5P	TAAGGTTGGTCCAATtCATAG	140033	140053	21	44
BART6-5P	BART6-5P	TAAGGTTGGTCCAATCCATAaG	140033	140054	22	4
BART6-5P	BART6-5P	TAAGGTTGGTCCAATCCATAGa	140033	140054	22	104
BART6-5P	BART6-5P	TAAGGTTGGTCCAATCCATAGc	140033	140054	22	11
BART6-5P	BART6-5P	TAAGGTTGGTCCAATCCATAGG	140033	140054	22	15
BART6-5P	BART6-5P	TAAGGTTGGTCCAATCCATAta	140033	140054	22	3
BART6-5P	BART6-5P	TAAGGTTGGTCCAATCCATAtt	140033	140054	22	3
BART6-5P	BART6-5P	TAAGGTTGGTCCAATCCATcGa	140033	140054	22	3
BART6-5P	BART6-5P	TAAGGTTGGTCCAATCCATgaa	140033	140054	22	7
BART6-5P	BART6-5P	TAAGGTTGGTCCAATCCATgGa	140033	140054	22	8
BART6-5P	BART6-5P	TAAGGTTGGTCCAATCCATgGc	140033	140054	22	8
BART6-5P	BART6-5P	TAAGGTTGGTCCAATCCATgGt	140033	140054	22	11
BART6-5P	BART6-5P	TAAGGTTGGTCCAATCCgTAGG	140033	140054	22	19
BART6-5P	BART6-5P	TAAGGTTGGTCCAATCCtagGt	140033	140054	22	4
BART6-5P	BART6-5P	agAGGTTGGTCCAATCCATAGGC	140033	140055	23	8
BART6-5P	BART6-5P	TAAGGTTGGgCCAATCCATAGGC	140033	140055	23	3
BART6-5P	BART6-5P	TAAGGTTGGTCCAATCCAaAGGC	140033	140055	23	5
BART6-5P	BART6-5P	TAAGGTTGGTCCAATCCAgAGGC	140033	140055	23	16
BART6-5P	BART6-5P	TAAGGTTGGTCCAATCCATAaaC	140033	140055	23	9
BART6-5P	BART6-5P	TAAGGTTGGTCCAATCCATAaat	140033	140055	23	14
BART6-5P	BART6-5P	TAAGGTTGGTCCAATCCATAaGC	140033	140055	23	4
BART6-5P	BART6-5P	TAAGGTTGGTCCAATCCATAcGC	140033	140055	23	5
BART6-5P	BART6-5P	TAAGGTTGGTCCAATCCATAGat	140033	140055	23	5
BART6-5P	BART6-5P	TAAGGTTGGTCCAATCCATAGGa	140033	140055	23	3
BART6-5P	BART6-5P	TAAGGTTGGTCCAATCCATAGGC	140033	140055	23	709
BART6-5P	BART6-5P	TAAGGTTGGTCCAATCCATAGGt	140033	140055	23	22
BART6-5P	BART6-5P	TAAGGTTGGTCCAATCCATgGGC	140033	140055	23	3
BART6-5P	BART6-5P	TAAGGTTGGTCCAATCCATtGGC	140033	140055	23	3
BART6-5P	BART6-5P	TAAGGTTGGTCCAATCCcTAGGC	140033	140055	23	15
BART6-5P	BART6-5P	TAAGGTTGGTCCAATCCgTAGGC	140033	140055	23	31
BART6-5P	BART6-5P	TAAGGTTGGTCCAATCCgTAGGt	140033	140055	23	3
BART6-5P	BART6-5P	TAAGGTTGGTCCAATCCAaAGGCg	140033	140056	24	4
BART6-5P	BART6-5P	TAAGGTTGGTCCAATCCAcAGGCg	140033	140056	24	6
BART6-5P	BART6-5P	TAAGGTTGGTCCAATCCAgAGGCg	140033	140056	24	3
BART6-5P	BART6-5P	TAAGGTTGGTCCAATCCAgAGGCT	140033	140056	24	3
BART6-5P	BART6-5P	TAAGGTTGGTCCAATCCATAGaCT	140033	140056	24	19
BART6-5P	BART6-5P	TAAGGTTGGTCCAATCCATAGGaT	140033	140056	24	14
BART6-5P	BART6-5P	TAAGGTTGGTCCAATCCATAGGCa	140033	140056	24	31
BART6-5P	BART6-5P	TAAGGTTGGTCCAATCCATAGGCc	140033	140056	24	5
BART6-5P	BART6-5P	TAAGGTTGGTCCAATCCATAGGCg	140033	140056	24	12
BART6-5P	BART6-5P	TAAGGTTGGTCCAATCCATAGGCT	140033	140056	24	51
BART6-5P	BART6-5P	TAAGGTTGGTCCAATCCATAGGgT	140033	140056	24	4
BART6-5P	BART6-5P	TAAGGTTGGTCCAATCCATAGGta	140033	140056	24	5
BART6-5P	BART6-5P	TAAGGTTGGTCCAATCCATAGGtg	140033	140056	24	6
BART6-5P	BART6-5P	TAAGGTTGGTCCAATCCATAGGtT	140033	140056	24	15
BART6-5P	BART6-5P	TAAGGTTGGTCCAATCCATgGGCa	140033	140056	24	7
BART6-5P	BART6-5P	TAAGGTTGGTCCAATCCATAGGCgg	140033	140057	25	3
BART6-5P	BART6-5P	TAAGGTTGGTCCAATCCATAGGCTg	140033	140057	25	3
BART6-5P	BART6-5P	TAAGGTTGGTCCAATCCATAGGCTT	140033	140057	25	11
BART6-5P	BART6-5P	TAAGGTTGGTCCAATCCATAGGtaT	140033	140057	25	3
BART6-5P	BART6-5P	TAAGGTTGGTCCAATCCATAGGCTTT	140033	140058	26	4
BART6-5P	BART6-5P	TAAGGTTGGTCCAATCCATAGGCTcTT	140033	140059	27	3
BART6-5P	BART6-5P	AAGGTTGGTCCAATCCATAGa	140034	140054	21	5
BART6-5P	BART6-5P	AgaGTTGGTCCAATCCATAGa	140034	140054	21	4
BART6-5P	BART6-5P	AAGGTTGGTCCAATCCATAGGC	140034	140055	22	8
BART6-3P	BART6-3P	CCGGGGATCGGACTAaCCTcA	140071	140091	21	4
BART6-3P	BART6-3P	CGGGGATCGGACTAaCCaa	140072	140090	19	5
BART6-3P	BART6-3P	CGGGGATCGGACTAaCCTg	140072	140090	19	3
BART6-3P	BART6-3P	CGGGGATCGGACTAaCCTT	140072	140090	19	3
BART6-3P	BART6-3P	CGGGGATCGGACTAGCCaa	140072	140090	19	4
BART6-3P	BART6-3P	CGGGGATCGGACTAGCCag	140072	140090	19	4
BART6-3P	BART6-3P	CGGGGATCGGACTAGCCaT	140072	140090	19	13
BART6-3P	BART6-3P	CGGGGATCGGACTAGCCcg	140072	140090	19	6
BART6-3P	BART6-3P	CGGGGATCGGACTAGCCcT	140072	140090	19	4
BART6-3P	BART6-3P	CGGGGATCGGACTAGCCga	140072	140090	19	115
BART6-3P	BART6-3P	CGGGGATCGGACTAGCCTa	140072	140090	19	12

BART6-3P	BART6-3P	CGGGGATCGGACTAGCCTc	140072	140090	19	19
BART6-3P	BART6-3P	CGGGGATCGGACTAGCCTg	140072	140090	19	8
BART6-3P	BART6-3P	CGGGGATCGGACTAGCCTT	140072	140090	19	36
BART6-3P	BART6-3P	aGGGGATCGGACTAGCCTTA	140072	140091	20	6
BART6-3P	BART6-3P	CGGGGATCGGACTAaCCTTA	140072	140091	20	61
BART6-3P	BART6-3P	CGGGGATCGGACTAcCCTTA	140072	140091	20	6
BART6-3P	BART6-3P	CGGGGATCGGACTAGCaTgA	140072	140091	20	3
BART6-3P	BART6-3P	CGGGGATCGGACTAGCCaaA	140072	140091	20	3
BART6-3P	BART6-3P	CGGGGATCGGACTAGCCagA	140072	140091	20	14
BART6-3P	BART6-3P	CGGGGATCGGACTAGCCaTA	140072	140091	20	9
BART6-3P	BART6-3P	CGGGGATCGGACTAGCCcaA	140072	140091	20	9
BART6-3P	BART6-3P	CGGGGATCGGACTAGCCcgA	140072	140091	20	12
BART6-3P	BART6-3P	CGGGGATCGGACTAGCCcTA	140072	140091	20	4
BART6-3P	BART6-3P	CGGGGATCGGACTAGCCggA	140072	140091	20	5
BART6-3P	BART6-3P	CGGGGATCGGACTAGCCTaA	140072	140091	20	18
BART6-3P	BART6-3P	CGGGGATCGGACTAGCCTgA	140072	140091	20	12
BART6-3P	BART6-3P	CGGGGATCGGACTAGCCTgg	140072	140091	20	14
BART6-3P	BART6-3P	CGGGGATCGGACTAGCCTTA	140072	140091	20	185
BART6-3P	BART6-3P	CGGGGATCGGACTAGCCTTc	140072	140091	20	18
BART6-3P	BART6-3P	CGGGGATCGGACTAGCCTTt	140072	140091	20	7
BART6-3P	BART6-3P	CGGGGATCGGACTAGCtagA	140072	140091	20	6
BART6-3P	BART6-3P	CGGGGATCGGACTAGCtTTc	140072	140091	20	7
BART6-3P	BART6-3P	CGGGGATCGGACTAGaCTTAG	140072	140092	21	22
BART6-3P	BART6-3P	CGGGGATCGGACTAGCCcTAG	140072	140092	21	5
BART6-3P	BART6-3P	CGGGGATCGGACTAGCCTcAa	140072	140092	21	4
BART6-3P	BART6-3P	CGGGGATCGGACTAGCCTgAG	140072	140092	21	9
BART6-3P	BART6-3P	CGGGGATCGGACTAGCCTTAa	140072	140092	21	28
BART6-3P	BART6-3P	CGGGGATCGGACTAGCCTTAG	140072	140092	21	195
BART6-3P	BART6-3P	CGGGGATCGGACTAGCCTTAt	140072	140092	21	10
BART6-3P	BART6-3P	CGGGGATCGGACTAGCCTTcG	140072	140092	21	4
BART6-3P	BART6-3P	CGGGGATCGGACTAGCCTTga	140072	140092	21	22
BART6-3P	BART6-3P	CGGGGATCGGACTAGCCTTtc	140072	140092	21	7
BART6-3P	BART6-3P	CGGGGATCGGACTAGCCTTtG	140072	140092	21	7
BART6-3P	BART6-3P	CGGGGATCGGACTAGCtTTAG	140072	140092	21	3
BART6-3P	BART6-3P	CGGGGATCGGACTAGtCTTAG	140072	140092	21	6
BART6-3P	BART6-3P	CGGGGATCGGACTAGCaaTAGA	140072	140093	22	4
BART6-3P	BART6-3P	CGGGGATCGGACTAGCacTAGA	140072	140093	22	4
BART6-3P	BART6-3P	CGGGGATCGGACTAGCaTaAGA	140072	140093	22	9
BART6-3P	BART6-3P	CGGGGATCGGACTAGCaTTAGA	140072	140093	22	20
BART6-3P	BART6-3P	CGGGGATCGGACTAGCaTTgGA	140072	140093	22	11
BART6-3P	BART6-3P	CGGGGATCGGACTAGCCaaAGA	140072	140093	22	4
BART6-3P	BART6-3P	CGGGGATCGGACTAGCCaacGA	140072	140093	22	5
BART6-3P	BART6-3P	CGGGGATCGGACTAGCCaatGA	140072	140093	22	3
BART6-3P	BART6-3P	CGGGGATCGGACTAGCCacAGA	140072	140093	22	4
BART6-3P	BART6-3P	CGGGGATCGGACTAGCCacgGA	140072	140093	22	11
BART6-3P	BART6-3P	CGGGGATCGGACTAGCCagAGA	140072	140093	22	3
BART6-3P	BART6-3P	CGGGGATCGGACTAGCCagtGA	140072	140093	22	7
BART6-3P	BART6-3P	CGGGGATCGGACTAGCCaTAGA	140072	140093	22	41
BART6-3P	BART6-3P	CGGGGATCGGACTAGCCaTcGA	140072	140093	22	3
BART6-3P	BART6-3P	CGGGGATCGGACTAGCCaTgGA	140072	140093	22	6
BART6-3P	BART6-3P	CGGGGATCGGACTAGCCcgAGA	140072	140093	22	3
BART6-3P	BART6-3P	CGGGGATCGGACTAGCCcgAGc	140072	140093	22	3
BART6-3P	BART6-3P	CGGGGATCGGACTAGCCcTAGA	140072	140093	22	21
BART6-3P	BART6-3P	CGGGGATCGGACTAGCCgaAGA	140072	140093	22	5
BART6-3P	BART6-3P	CGGGGATCGGACTAGCCgagGA	140072	140093	22	3
BART6-3P	BART6-3P	CGGGGATCGGACTAGCCgccGA	140072	140093	22	6
BART6-3P	BART6-3P	CGGGGATCGGACTAGCCggAGA	140072	140093	22	4
BART6-3P	BART6-3P	CGGGGATCGGACTAGCCgTAGA	140072	140093	22	8
BART6-3P	BART6-3P	CGGGGATCGGACTAGCCgTtGA	140072	140093	22	3
BART6-3P	BART6-3P	CGGGGATCGGACTAGCCTaAGA	140072	140093	22	6
BART6-3P	BART6-3P	CGGGGATCGGACTAGCCTacGA	140072	140093	22	5
BART6-3P	BART6-3P	CGGGGATCGGACTAGCCTagGA	140072	140093	22	3
BART6-3P	BART6-3P	CGGGGATCGGACTAGCCTatGA	140072	140093	22	6
BART6-3P	BART6-3P	CGGGGATCGGACTAGCCTcAGA	140072	140093	22	6
BART6-3P	BART6-3P	CGGGGATCGGACTAGCCTcgGA	140072	140093	22	3
BART6-3P	BART6-3P	CGGGGATCGGACTAGCCTctGA	140072	140093	22	11
BART6-3P	BART6-3P	CGGGGATCGGACTAGCCTgAGA	140072	140093	22	7
BART6-3P	BART6-3P	CGGGGATCGGACTAGCCTTAaA	140072	140093	22	9

BART6-3P	BART6-3P	CGGGGATCGGACTAGCCTTAGA	140072	140093	22	430
BART6-3P	BART6-3P	CGGGGATCGGACTAGCCTTAtc	140072	140093	22	3
BART6-3P	BART6-3P	CGGGGATCGGACTAGCCTTcGA	140072	140093	22	8
BART6-3P	BART6-3P	CGGGGATCGGACTAGCCTTgGA	140072	140093	22	8
BART6-3P	BART6-3P	CGGGGATCGGACTAGCCTTtct	140072	140093	22	20
BART6-3P	BART6-3P	CGGGGATCGGACTAGCCTTtGA	140072	140093	22	3
BART6-3P	BART6-3P	CGGGGATCGGACTAGCgTTAGA	140072	140093	22	26
BART6-3P	BART6-3P	CGGGGATCGGACTAGCtTTAGA	140072	140093	22	4
BART6-3P	BART6-3P	tGGGGATCGGACTAGCCTTAGA	140072	140093	22	6
BART6-3P	BART6-3P	CGGGGATCGGACTAGCCaaAGAG	140072	140094	23	5
BART6-3P	BART6-3P	CGGGGATCGGACTAGCCTaAGAG	140072	140094	23	11
BART6-3P	BART6-3P	CGGGGATCGGACTAGCCTcAGAG	140072	140094	23	9
BART6-3P	BART6-3P	CGGGGATCGGACTAGCCTTAGAc	140072	140094	23	4
BART6-3P	BART6-3P	CGGGGATCGGACTAGCCTTAGAG	140072	140094	23	85
BART6-3P	BART6-3P	CGGGGATCGGACTAGCCTTAGgG	140072	140094	23	7
BART6-3P	BART6-3P	CGGGGATCGGACTAGCCTTAGtc	140072	140094	23	3
BART6-3P	BART6-3P	CGGGGATCGGACTAGCCTTcGAG	140072	140094	23	7
BART6-3P	BART6-3P	CGGGGATCGGACTAGCCTTgGAG	140072	140094	23	3
BART6-3P	BART6-3P	CGGGGATCGGACTAGCCTTAagGa	140072	140095	24	4
BART6-3P	BART6-3P	CGGGGATCGGACTAGCCTTAGAGT	140072	140095	24	5
BART6-3P	BART6-3P	CGGGGATCGGACTAGCCTTAGcGa	140072	140095	24	3
BART6-3P	BART6-3P	CGGGGATCGGACTAGCCTTAGAGcg	140072	140096	25	4
BART6-3P	BART6-3P	CGGGGATCGGACTAGCCTTAGAGTg	140072	140096	25	3
BART21-5P	BART21-5P	TCACTAGTGAAGGCAACTAAC	145514	145534	21	36
BART21-5P	BART21-5P	TCACTAGTGAAGGCAgCTAAC	145514	145534	21	6
BART21-5P	BART21-5P	TCACTAGTGAAGGCAAaTAACA	145514	145535	22	9
BART21-5P	BART21-5P	TCACTAGTGAAGGCAACcAACA	145514	145535	22	12
BART21-5P	BART21-5P	TCACTAGTGAAGGCAACTAACA	145514	145535	22	58
BART21-5P	BART21-5P	TCACTAGTGAAGGCAACTAgCA	145514	145535	22	6
BART21-5P	BART21-5P	TCACTAGTGAAGGCAACTAACAa	145514	145536	23	6
BART21-5P	BART21-5P	TCACTAGTGAAGGCAACTAACAC	145514	145536	23	15
BART21-5P	BART21-5P	TCACTAGTGAAGGCAACTAACca	145514	145536	23	4
BART21-5P	BART21-5P	TCACTAGTGAAGGCAACTAACACAc	145514	145538	25	7
BART21-3P	BART21-3P	CTAGTTGTGCCCACTGGTGaaT	145548	145569	22	3
BART21-3P	BART21-3P	CTAGTTGTGCCCACTGGTGcaT	145548	145569	22	4
BART21-3P	BART21-3P	CTAGTTGTGCCCACTGGTGTTT	145548	145569	22	9
BART18-5P	BART18-5P	TCAAGTTCGCACTaCCTAT	145962	145980	19	8
BART18-5P	BART18-5P	TCAAGTTCGCACTcCCTAT	145962	145980	19	3
BART18-5P	BART18-5P	TCAAGTTCGCACTTCCTAT	145962	145980	19	36
BART18-5P	BART18-5P	TCAAGTTCGCACTTCCTgT	145962	145980	19	6
BART18-5P	BART18-5P	TCAAGTTCGCACTTCCaAcA	145962	145981	20	5
BART18-5P	BART18-5P	TCAAGTTCGCACTTCCaccA	145962	145981	20	4
BART18-5P	BART18-5P	TCAAGTTCGCACTTCCatcA	145962	145981	20	3
BART18-5P	BART18-5P	TCAAGTTCGCACTTCCcAcA	145962	145981	20	4
BART18-5P	BART18-5P	TCAAGTTCGCACTTCCgAac	145962	145981	20	4
BART18-5P	BART18-5P	TCAAGTTCGCACTTCCgAcA	145962	145981	20	3
BART18-5P	BART18-5P	TCAAGTTCGCACTTCCTAaA	145962	145981	20	41
BART18-5P	BART18-5P	TCAAGTTCGCACTTCCTAcA	145962	145981	20	10
BART18-5P	BART18-5P	TCAAGTTCGCACTTCCTAcc	145962	145981	20	8
BART18-5P	BART18-5P	TCAAGTTCGCACTTCCTATA	145962	145981	20	46
BART18-5P	BART18-5P	TCAAGTTCGCACTTCCTATg	145962	145981	20	4
BART18-5P	BART18-5P	TCAAGTTCGCACTTCCTATt	145962	145981	20	4
BART18-5P	BART18-5P	TCAAGTTCGCACTTCCTtac	145962	145981	20	7
BART18-5P	BART18-5P	TCAAGTTCGCACTTCgTAcA	145962	145981	20	6
BART18-5P	BART18-5P	cCAAGTTCGCACTTCCTATAC	145962	145982	21	8
BART18-5P	BART18-5P	TCAAGTTCGCACTTCaTATAC	145962	145982	21	28
BART18-5P	BART18-5P	TCAAGTTCGCACTTCCaATAC	145962	145982	21	19
BART18-5P	BART18-5P	TCAAGTTCGCACTTCCcATAC	145962	145982	21	25
BART18-5P	BART18-5P	TCAAGTTCGCACTTCCgATAC	145962	145982	21	11
BART18-5P	BART18-5P	TCAAGTTCGCACTTCCTAaAC	145962	145982	21	3
BART18-5P	BART18-5P	TCAAGTTCGCACTTCCTAcAa	145962	145982	21	5
BART18-5P	BART18-5P	TCAAGTTCGCACTTCCTAcAC	145962	145982	21	36
BART18-5P	BART18-5P	TCAAGTTCGCACTTCCTAgAC	145962	145982	21	6
BART18-5P	BART18-5P	TCAAGTTCGCACTTCCTATAa	145962	145982	21	16
BART18-5P	BART18-5P	TCAAGTTCGCACTTCCTATAC	145962	145982	21	296
BART18-5P	BART18-5P	TCAAGTTCGCACTTCCTATAg	145962	145982	21	4
BART18-5P	BART18-5P	TCAAGTTCGCACTTCCTATAt	145962	145982	21	5
BART18-5P	BART18-5P	TCAAGTTCGCACTTCCTATca	145962	145982	21	6

BART18-5P	BART18-5P	TCAAGTTCGCACTTCgTATAC	145962	145982	21	15
BART18-5P	BART18-5P	TCAAGTTCGCACTTCtTATAC	145962	145982	21	4
BART18-5P	BART18-5P	agAAGTTCGCACTTCCTATACA	145962	145983	22	9
BART18-5P	BART18-5P	cCAAGTTCGCACTTCCTATACA	145962	145983	22	7
BART18-5P	BART18-5P	TacAGTTCGCACTTCCTATACA	145962	145983	22	3
BART18-5P	BART18-5P	TCAAGTTCGCACTTCCaAcACA	145962	145983	22	9
BART18-5P	BART18-5P	TCAAGTTCGCACTTCCaATACA	145962	145983	22	94
BART18-5P	BART18-5P	TCAAGTTCGCACTTCCagcACA	145962	145983	22	6
BART18-5P	BART18-5P	TCAAGTTCGCACTTCCcAcACA	145962	145983	22	10
BART18-5P	BART18-5P	TCAAGTTCGCACTTCCcATACA	145962	145983	22	42
BART18-5P	BART18-5P	TCAAGTTCGCACTTCCcATtCA	145962	145983	22	3
BART18-5P	BART18-5P	TCAAGTTCGCACTTCCgATACA	145962	145983	22	116
BART18-5P	BART18-5P	TCAAGTTCGCACTTCCTAaACA	145962	145983	22	7
BART18-5P	BART18-5P	TCAAGTTCGCACTTCCTAacCA	145962	145983	22	8
BART18-5P	BART18-5P	TCAAGTTCGCACTTCCTAagCA	145962	145983	22	10
BART18-5P	BART18-5P	TCAAGTTCGCACTTCCTAcACA	145962	145983	22	27
BART18-5P	BART18-5P	TCAAGTTCGCACTTCCTAccCA	145962	145983	22	3
BART18-5P	BART18-5P	TCAAGTTCGCACTTCCTAcgCA	145962	145983	22	4
BART18-5P	BART18-5P	TCAAGTTCGCACTTCCTActCA	145962	145983	22	25
BART18-5P	BART18-5P	TCAAGTTCGCACTTCCTATACA	145962	145983	22	290
BART18-5P	BART18-5P	TCAAGTTCGCACTTCCTATACg	145962	145983	22	6
BART18-5P	BART18-5P	TCAAGTTCGCACTTCCTATAtg	145962	145983	22	5
BART18-5P	BART18-5P	TCAAGTTCGCACTTCCTATcCA	145962	145983	22	3
BART18-5P	BART18-5P	TCAAGTTCGCACTTCCTATgCA	145962	145983	22	3
BART18-5P	BART18-5P	TCAAGTTCGCACTTCCTATtCA	145962	145983	22	3
BART18-5P	BART18-5P	TCAAGTTCGCACTTCCTcaACA	145962	145983	22	4
BART18-5P	BART18-5P	TCAAGTTCGCACTTCCTcTACA	145962	145983	22	5
BART18-5P	BART18-5P	TCAAGTTCGCACTTCCTgaACA	145962	145983	22	4
BART18-5P	BART18-5P	TCAAGTTCGCACTTCCTgTACA	145962	145983	22	6
BART18-5P	BART18-5P	TCAAGTTCGCACTTCCTAaACAG	145962	145984	23	12
BART18-5P	BART18-5P	TCAAGTTCGCACTTCCTATACAa	145962	145984	23	73
BART18-5P	BART18-5P	TCAAGTTCGCACTTCCTATACAc	145962	145984	23	19
BART18-5P	BART18-5P	TCAAGTTCGCACTTCCTATACAG	145962	145984	23	105
BART18-5P	BART18-5P	TCAAGTTCGCACTTCCTATACAt	145962	145984	23	5
BART18-5P	BART18-5P	TCAAGTTCGCACTTCCTATACca	145962	145984	23	52
BART18-5P	BART18-5P	TCAAGTTCGCACTTCCTATACcc	145962	145984	23	20
BART18-5P	BART18-5P	TCAAGTTCGCACTTCCTATACcG	145962	145984	23	8
BART18-5P	BART18-5P	TCAAGTTCGCACTTCCTATACga	145962	145984	23	38
BART18-5P	BART18-5P	TCAAGTTCGCACTTCCTATACgc	145962	145984	23	11
BART18-5P	BART18-5P	TCAAGTTCGCACTTCCTATACgt	145962	145984	23	3
BART18-5P	BART18-5P	TCAAGTTCGCACTTCCTATACta	145962	145984	23	4
BART18-5P	BART18-5P	TCAAGTTCGCACTTCCTATACtc	145962	145984	23	9
BART18-5P	BART18-5P	TCAAGTTCGCACTTCCTATACtG	145962	145984	23	18
BART18-5P	BART18-5P	TCAAGTTCGCACTTCCTATACtt	145962	145984	23	3
BART18-5P	BART18-5P	TCAAGTTCGCACTTCCTATAgtG	145962	145984	23	6
BART18-5P	BART18-5P	TCAAGTTCGCACTTCCTATAtAc	145962	145984	23	7
BART18-5P	BART18-5P	TCAAGTTCGCACTTCCTATtgtG	145962	145984	23	5
BART18-5P	BART18-5P	TCAAGTTCGCACTTCCTATACAGT	145962	145985	24	4
BART18-5P	BART18-5P	CAAGTTCGCACTTCCTAcA	145963	145981	19	3
BART18-5P	BART18-5P	CAAGTTCGCACTTCCgATACA	145963	145983	21	12
BART18-5P	BART18-5P	CAAGTTCGCACTTCCTATACAa	145963	145984	22	25
BART18-5P	BART18-5P	CAAGTTCGCACTTCCTATACAG	145963	145984	22	10
BART18-3P	BART18-3P	TATCGGAAGTTTGGGaTTCGT	145998	146018	21	4
BART18-3P	BART18-3P	TATCGGAAGTTTGGGCaTCGa	145998	146018	21	3
BART18-3P	BART18-3P	TATCGGAAGTTTGGGCTcCGT	145998	146018	21	5
BART18-3P	BART18-3P	TATCGGAAGTTTGGGCTTCGT	145998	146018	21	6
BART18-3P	BART18-3P	TATCGGAAGTTTGGGCTTCaTC	145998	146019	22	6
BART18-3P	BART18-3P	TATCGGAAGTTTGGGCTTCGcC	145998	146019	22	3
BART18-3P	BART18-3P	TATCGGAAGTTTGGGCTTCGTC	145998	146019	22	8
BART18-3P	BART18-3P	TATCGGAAGTTTGGGCTTgcTC	145998	146019	22	3
BART18-3P	BART18-3P	TATCGGAAGTTTGGGCTTCGgaCa	145998	146021	24	3
BART18-3P	BART18-3P	TATCGGAAGTTTGGGCTTCGTCat	145998	146021	24	3
BART7-5P	BART7-star	CCTGGACCTTGACTAaGAA	146439	146457	19	5
BART7-5P	BART7-star	CCTGGACCTTGACTAcaAA	146439	146457	19	6
BART7-5P	BART7-star	CCTGGACCTTGACTAcacA	146439	146457	19	4
BART7-5P	BART7-star	CCTGGACCTTGACTATGAA	146439	146457	19	3
BART7-5P	BART7-star	CCTGGACCTTGACTATcAAc	146439	146458	20	4
BART7-5P	BART7-star	CCTGGACCTTGACTATGAAA	146439	146458	20	4

BART7-5P	BART7-star	CCTGGACCTTGACTATGAAc	146439	146458	20	6
BART7-5P	BART7-star	CCTGGACCTTGACTAaGAAAC	146439	146459	21	15
BART7-5P	BART7-star	CCTGGACCTTGACTAcGAAAC	146439	146459	21	5
BART7-5P	BART7-star	CCTGGACCTTGACTAgGAAAC	146439	146459	21	4
BART7-5P	BART7-star	CCTGGACCTTGACTATGAAAC	146439	146459	21	51
BART7-5P	BART7-star	CCTGGACCTTGACTATGcAAC	146439	146459	21	5
BART7-5P	BART7-star	CCTGGACCTTGACTATaAAACA	146439	146460	22	23
BART7-5P	BART7-star	CCTGGACCTTGACTATGAAACA	146439	146460	22	112
BART7-5P	BART7-star	CCTGGACCTTGACTATGgAACA	146439	146460	22	3
BART7-5P	BART7-star	CgTGGACCTTGACTATGAAACA	146439	146460	22	3
BART7-5P	BART7-star	CCTGGACCTTGACTATGAAACAA	146439	146461	23	11
BART7-5P	BART7-star	CCTGGACCTTGACTATGAAAgcA	146439	146461	23	5
BART7-5P	BART7-star	CCTGGACCTTGACTATGAAcacA	146439	146461	23	3
BART7-5P	BART7-star	CCTGGACCTTGACTATGAAACAAg	146439	146462	24	4
BART7-3P	BART7	CATCATAGTCCAGaGTCCA	146475	146493	19	12
BART7-3P	BART7	CATCATAGTCCAGcGTCCA	146475	146493	19	4
BART7-3P	BART7	CATCATAGTCCAGgGTCCA	146475	146493	19	13
BART7-3P	BART7	CATCATAGTCCAGTaTCCA	146475	146493	19	5
BART7-3P	BART7	CATCATAGTCCAGTGcCac	146475	146493	19	3
BART7-3P	BART7	CATCATAGTCCAGTGcCCA	146475	146493	19	7
BART7-3P	BART7	CATCATAGTCCAGTGgaCc	146475	146493	19	4
BART7-3P	BART7	CATCATAGTCCAGTGTCaA	146475	146493	19	4
BART7-3P	BART7	CATCATAGTCCAGTGTCac	146475	146493	19	12
BART7-3P	BART7	CATCATAGTCCAGTGTCCA	146475	146493	19	52
BART7-3P	BART7	CATCATAGTCCAGTGTCCc	146475	146493	19	4
BART7-3P	BART7	CATCATAGTCCAGTGTCCg	146475	146493	19	11
BART7-3P	BART7	CATCATAGTCCAGTGTCCt	146475	146493	19	5
BART7-3P	BART7	CATCATAGTCCAGTGTCtg	146475	146493	19	6
BART7-3P	BART7	CATCATAGTCCAGTGTgCA	146475	146493	19	9
BART7-3P	BART7	CATCATAGTCCAGTacCCAG	146475	146494	20	7
BART7-3P	BART7	CATCATAGTCCAGTagCCAG	146475	146494	20	17
BART7-3P	BART7	CATCATAGTCCAGTaTCCAa	146475	146494	20	6
BART7-3P	BART7	CATCATAGTCCAGTaTCCAG	146475	146494	20	95
BART7-3P	BART7	CATCATAGTCCAGTaTCCAt	146475	146494	20	9
BART7-3P	BART7	CATCATAGTCCAGTaTCCgG	146475	146494	20	15
BART7-3P	BART7	CATCATAGTCCAGTcTCCAG	146475	146494	20	4
BART7-3P	BART7	CATCATAGTCCAGTGaaCAG	146475	146494	20	3
BART7-3P	BART7	CATCATAGTCCAGTGaCCAa	146475	146494	20	5
BART7-3P	BART7	CATCATAGTCCAGTGaCCAG	146475	146494	20	6
BART7-3P	BART7	CATCATAGTCCAGTGaCCAt	146475	146494	20	11
BART7-3P	BART7	CATCATAGTCCAGTGaCCga	146475	146494	20	3
BART7-3P	BART7	CATCATAGTCCAGTGaCCgG	146475	146494	20	8
BART7-3P	BART7	CATCATAGTCCAGTGcCCAa	146475	146494	20	8
BART7-3P	BART7	CATCATAGTCCAGTGcCCAG	146475	146494	20	7
BART7-3P	BART7	CATCATAGTCCAGTGcCCAt	146475	146494	20	6
BART7-3P	BART7	CATCATAGTCCAGTGcCCga	146475	146494	20	3
BART7-3P	BART7	CATCATAGTCCAGTGcCCtt	146475	146494	20	13
BART7-3P	BART7	CATCATAGTCCAGTGTaCca	146475	146494	20	3
BART7-3P	BART7	CATCATAGTCCAGTGTaggG	146475	146494	20	13
BART7-3P	BART7	CATCATAGTCCAGTGTCCAa	146475	146494	20	38
BART7-3P	BART7	CATCATAGTCCAGTGTCCAc	146475	146494	20	27
BART7-3P	BART7	CATCATAGTCCAGTGTCCAG	146475	146494	20	573
BART7-3P	BART7	CATCATAGTCCAGTGTCCAt	146475	146494	20	33
BART7-3P	BART7	CATCATAGTCCAGTGTCCca	146475	146494	20	8
BART7-3P	BART7	CATCATAGTCCAGTGTCCcG	146475	146494	20	8
BART7-3P	BART7	CATCATAGTCCAGTGTCCct	146475	146494	20	8
BART7-3P	BART7	CATCATAGTCCAGTGTCCga	146475	146494	20	3
BART7-3P	BART7	CATCATAGTCCAGTGTCCgG	146475	146494	20	8
BART7-3P	BART7	CATCATAGTCCAGTGTCCgt	146475	146494	20	4
BART7-3P	BART7	CATCATAGTCCAGTGTCCta	146475	146494	20	3
BART7-3P	BART7	CATCATAGTCCAGTGTCCtG	146475	146494	20	7
BART7-3P	BART7	CATCATAGTCCAGTGTCCtt	146475	146494	20	17
BART7-3P	BART7	CATCATAGTCCAGTGTCtAG	146475	146494	20	9
BART7-3P	BART7	CATCATAGTCCAGTGTCtgG	146475	146494	20	5
BART7-3P	BART7	CATCATAGTCCAGTGTtCAG	146475	146494	20	4
BART7-3P	BART7	CtTCATAGTCCAGTGTCCAG	146475	146494	20	3
BART7-3P	BART7	CATCATAGTCCAGTGaCCAGa	146475	146495	21	10
BART7-3P	BART7	CATCATAGTCCAGTGcCCAGa	146475	146495	21	28

BART7-3P	BART7	CATCATAGTCCAGTGgCCAGa	146475	146495	21	5
BART7-3P	BART7	CATCATAGTCCAGTGTCacaG	146475	146495	21	5
BART7-3P	BART7	CATCATAGTCCAGTGTCCAcG	146475	146495	21	4
BART7-3P	BART7	CATCATAGTCCAGTGTCCAGa	146475	146495	21	66
BART7-3P	BART7	CATCATAGTCCAGTGTCCAGc	146475	146495	21	19
BART7-3P	BART7	CATCATAGTCCAGTGTCCAGG	146475	146495	21	32
BART7-3P	BART7	CATCATAGTCCAGTGTCCAGt	146475	146495	21	9
BART7-3P	BART7	CATCATAGTCCAGTGTCCAta	146475	146495	21	5
BART7-3P	BART7	CATCATAGTCCAGTGTCCcaG	146475	146495	21	4
BART7-3P	BART7	CATCATAGTCCAGTGTCCcGc	146475	146495	21	3
BART7-3P	BART7	CATCATAGTCCAGTGTCCcGG	146475	146495	21	4
BART7-3P	BART7	CATCATAGTCCAGTGTCCcGt	146475	146495	21	4
BART7-3P	BART7	CATCATAGTCCAGTGTCCgaG	146475	146495	21	3
BART7-3P	BART7	CATCATAGTCCAGTGTCCgGG	146475	146495	21	7
BART7-3P	BART7	CATCATAGTCCAGTGTCCgGt	146475	146495	21	7
BART7-3P	BART7	CATCATAGTCCAGTGTCCtGa	146475	146495	21	4
BART7-3P	BART7	CATCATAGTCCAGTGTCCtGG	146475	146495	21	14
BART7-3P	BART7	CATCATAGTCCAGTGTCCtGt	146475	146495	21	3
BART7-3P	BART7	CATCATAGTCCAGTGTCggGt	146475	146495	21	3
BART7-3P	BART7	CATCATAGTCCAGTGTCttGG	146475	146495	21	4
BART7-3P	BART7	CATCATAGTCCAGTGTaCAGGG	146475	146496	22	10
BART7-3P	BART7	CATCATAGTCCAGTGTCCAacG	146475	146496	22	6
BART7-3P	BART7	CATCATAGTCCAGTGTCCAaGG	146475	146496	22	4
BART7-3P	BART7	CATCATAGTCCAGTGTCCAaGt	146475	146496	22	6
BART7-3P	BART7	CATCATAGTCCAGTGTCCAcaG	146475	146496	22	4
BART7-3P	BART7	CATCATAGTCCAGTGTCCAcGa	146475	146496	22	5
BART7-3P	BART7	CATCATAGTCCAGTGTCCAcGG	146475	146496	22	36
BART7-3P	BART7	CATCATAGTCCAGTGTCCAGaG	146475	146496	22	7
BART7-3P	BART7	CATCATAGTCCAGTGTCCAGat	146475	146496	22	4
BART7-3P	BART7	CATCATAGTCCAGTGTCCAGca	146475	146496	22	5
BART7-3P	BART7	CATCATAGTCCAGTGTCCAGcc	146475	146496	22	8
BART7-3P	BART7	CATCATAGTCCAGTGTCCAGcG	146475	146496	22	32
BART7-3P	BART7	CATCATAGTCCAGTGTCCAGGa	146475	146496	22	17
BART7-3P	BART7	CATCATAGTCCAGTGTCCAGGc	146475	146496	22	13
BART7-3P	BART7	CATCATAGTCCAGTGTCCAGGG	146475	146496	22	197
BART7-3P	BART7	CATCATAGTCCAGTGTCCAGGt	146475	146496	22	14
BART7-3P	BART7	CATCATAGTCCAGTGTCCAGta	146475	146496	22	11
BART7-3P	BART7	CATCATAGTCCAGTGTCCAGtG	146475	146496	22	7
BART7-3P	BART7	CATCATAGTCCAGTGTCCAGtt	146475	146496	22	10
BART7-3P	BART7	CATCATAGTCCAGTGTCCAtGa	146475	146496	22	8
BART7-3P	BART7	CATCATAGTCCAGTGTCCAtGG	146475	146496	22	5
BART7-3P	BART7	CATCATAGTCCAGTGTCCccGG	146475	146496	22	5
BART7-3P	BART7	CATCATAGTCCAGTGTCCcGGG	146475	146496	22	24
BART7-3P	BART7	CATCATAGTCCAGTGTCCctaG	146475	146496	22	4
BART7-3P	BART7	CATCATAGTCCAGTGTCCctGG	146475	146496	22	8
BART7-3P	BART7	CATCATAGTCCAGTGTCCgGGG	146475	146496	22	23
BART7-3P	BART7	CATCATAGTCCAGTGTCCgtGG	146475	146496	22	8
BART7-3P	BART7	CATCATAGTCCAGTGTCCtaGG	146475	146496	22	7
BART7-3P	BART7	CATCATAGTCCAGTGTCCtcGG	146475	146496	22	28
BART7-3P	BART7	CATCATAGTCCAGTGTCCtGaG	146475	146496	22	3
BART7-3P	BART7	CATCATAGTCCAGTGTCCtGGa	146475	146496	22	3
BART7-3P	BART7	CATCATAGTCCAGTGTCCtGGG	146475	146496	22	17
BART7-3P	BART7	CATCATAGTCCAGTGTCCttGG	146475	146496	22	7
BART7-3P	BART7	CATCATAGTCCAGTGTCgAGGG	146475	146496	22	4
BART7-3P	BART7	CATCATAGTCCAGTGTCtAGGG	146475	146496	22	10
BART7-3P	BART7	CATCATAGTCCAGTGTgCAGGG	146475	146496	22	7
BART7-3P	BART7	CATCATAGTCCAGTGTtCAGGG	146475	146496	22	15
BART7-3P	BART7	CATCATAGTCCAGcGTCCAaGGA	146475	146497	23	6
BART7-3P	BART7	CATCATAGTCCAGTGTCaAcGGt	146475	146497	23	3
BART7-3P	BART7	CATCATAGTCCAGTGTCaAGGaA	146475	146497	23	5
BART7-3P	BART7	CATCATAGTCCAGTGTCaAGGGA	146475	146497	23	25
BART7-3P	BART7	CATCATAGTCCAGTGTCaAGGGt	146475	146497	23	14
BART7-3P	BART7	CATCATAGTCCAGTGTCCAacaA	146475	146497	23	5
BART7-3P	BART7	CATCATAGTCCAGTGTCCAacGt	146475	146497	23	5
BART7-3P	BART7	CATCATAGTCCAGTGTCCAaGGA	146475	146497	23	17
BART7-3P	BART7	CATCATAGTCCAGTGTCCAaGGt	146475	146497	23	7
BART7-3P	BART7	CATCATAGTCCAGTGTCCAaGtA	146475	146497	23	17
BART7-3P	BART7	CATCATAGTCCAGTGTCCAataA	146475	146497	23	4

BART7-3P	BART7	CATCATAGTCCAGTGTCCAatGA	146475	146497	23	5
BART7-3P	BART7	CATCATAGTCCAGTGTCCAattA	146475	146497	23	10
BART7-3P	BART7	CATCATAGTCCAGTGTCCAcatA	146475	146497	23	7
BART7-3P	BART7	CATCATAGTCCAGTGTCCAccaA	146475	146497	23	4
BART7-3P	BART7	CATCATAGTCCAGTGTCCAccGt	146475	146497	23	9
BART7-3P	BART7	CATCATAGTCCAGTGTCCAcGac	146475	146497	23	4
BART7-3P	BART7	CATCATAGTCCAGTGTCCAcGat	146475	146497	23	4
BART7-3P	BART7	CATCATAGTCCAGTGTCCAcGGA	146475	146497	23	8
BART7-3P	BART7	CATCATAGTCCAGTGTCCAcGGc	146475	146497	23	3
BART7-3P	BART7	CATCATAGTCCAGTGTCCAcGGt	146475	146497	23	6
BART7-3P	BART7	CATCATAGTCCAGTGTCCActaA	146475	146497	23	4
BART7-3P	BART7	CATCATAGTCCAGTGTCCActGA	146475	146497	23	11
BART7-3P	BART7	CATCATAGTCCAGTGTCCActGc	146475	146497	23	4
BART7-3P	BART7	CATCATAGTCCAGTGTCCActGt	146475	146497	23	20
BART7-3P	BART7	CATCATAGTCCAGTGTCCAGaaA	146475	146497	23	28
BART7-3P	BART7	CATCATAGTCCAGTGTCCAGaat	146475	146497	23	38
BART7-3P	BART7	CATCATAGTCCAGTGTCCAGacA	146475	146497	23	4
BART7-3P	BART7	CATCATAGTCCAGTGTCCAGaGA	146475	146497	23	41
BART7-3P	BART7	CATCATAGTCCAGTGTCCAGaGc	146475	146497	23	3
BART7-3P	BART7	CATCATAGTCCAGTGTCCAGaGt	146475	146497	23	8
BART7-3P	BART7	CATCATAGTCCAGTGTCCAGatA	146475	146497	23	4
BART7-3P	BART7	CATCATAGTCCAGTGTCCAGcaA	146475	146497	23	5
BART7-3P	BART7	CATCATAGTCCAGTGTCCAGccc	146475	146497	23	3
BART7-3P	BART7	CATCATAGTCCAGTGTCCAGcct	146475	146497	23	3
BART7-3P	BART7	CATCATAGTCCAGTGTCCAGcGc	146475	146497	23	3
BART7-3P	BART7	CATCATAGTCCAGTGTCCAGcGt	146475	146497	23	5
BART7-3P	BART7	CATCATAGTCCAGTGTCCAGctA	146475	146497	23	4
BART7-3P	BART7	CATCATAGTCCAGTGTCCAGGaA	146475	146497	23	128
BART7-3P	BART7	CATCATAGTCCAGTGTCCAGGac	146475	146497	23	39
BART7-3P	BART7	CATCATAGTCCAGTGTCCAGGag	146475	146497	23	9
BART7-3P	BART7	CATCATAGTCCAGTGTCCAGGat	146475	146497	23	216
BART7-3P	BART7	CATCATAGTCCAGTGTCCAGGcA	146475	146497	23	21
BART7-3P	BART7	CATCATAGTCCAGTGTCCAGGGA	146475	146497	23	539
BART7-3P	BART7	CATCATAGTCCAGTGTCCAGGGc	146475	146497	23	120
BART7-3P	BART7	CATCATAGTCCAGTGTCCAGGGg	146475	146497	23	15
BART7-3P	BART7	CATCATAGTCCAGTGTCCAGGGt	146475	146497	23	112
BART7-3P	BART7	CATCATAGTCCAGTGTCCAGGtA	146475	146497	23	35
BART7-3P	BART7	CATCATAGTCCAGTGTCCAGGtc	146475	146497	23	10
BART7-3P	BART7	CATCATAGTCCAGTGTCCAGGtg	146475	146497	23	4
BART7-3P	BART7	CATCATAGTCCAGTGTCCAGGtt	146475	146497	23	35
BART7-3P	BART7	CATCATAGTCCAGTGTCCAGtaA	146475	146497	23	8
BART7-3P	BART7	CATCATAGTCCAGTGTCCAGtat	146475	146497	23	15
BART7-3P	BART7	CATCATAGTCCAGTGTCCAGtcA	146475	146497	23	3
BART7-3P	BART7	CATCATAGTCCAGTGTCCAGtGA	146475	146497	23	13
BART7-3P	BART7	CATCATAGTCCAGTGTCCAGtGc	146475	146497	23	7
BART7-3P	BART7	CATCATAGTCCAGTGTCCAGtGt	146475	146497	23	19
BART7-3P	BART7	CATCATAGTCCAGTGTCCAGttA	146475	146497	23	29
BART7-3P	BART7	CATCATAGTCCAGTGTCCAtaGA	146475	146497	23	8
BART7-3P	BART7	CATCATAGTCCAGTGTCCAtcGA	146475	146497	23	6
BART7-3P	BART7	CATCATAGTCCAGTGTCCAtGaA	146475	146497	23	3
BART7-3P	BART7	CATCATAGTCCAGTGTCCAtGat	146475	146497	23	11
BART7-3P	BART7	CATCATAGTCCAGTGTCCAtGGA	146475	146497	23	15
BART7-3P	BART7	CATCATAGTCCAGTGTCCAtGGc	146475	146497	23	3
BART7-3P	BART7	CATCATAGTCCAGTGTCCAtGGt	146475	146497	23	37
BART7-3P	BART7	CATCATAGTCCAGTGTCCAtGtA	146475	146497	23	3
BART7-3P	BART7	CATCATAGTCCAGTGTCCAtGtt	146475	146497	23	7
BART7-3P	BART7	CATCATAGTCCAGTGTCCAttGA	146475	146497	23	7
BART7-3P	BART7	CATCATAGTCCAGTGTCCAttGt	146475	146497	23	3
BART7-3P	BART7	CATCATAGTCCAGTGTCCcGaGA	146475	146497	23	17
BART7-3P	BART7	CATCATAGTCCAGTGTCCcGGaA	146475	146497	23	5
BART7-3P	BART7	CATCATAGTCCAGTGTCCcGGGA	146475	146497	23	3
BART7-3P	BART7	CATCATAGTCCAGTGTCCcGGtA	146475	146497	23	3
BART7-3P	BART7	CATCATAGTCCAGTGTCCgaGaA	146475	146497	23	5
BART7-3P	BART7	CATCATAGTCCAGTGTCCgaGGA	146475	146497	23	5
BART7-3P	BART7	CATCATAGTCCAGTGTCCgatGt	146475	146497	23	6
BART7-3P	BART7	CATCATAGTCCAGTGTCCgcaGt	146475	146497	23	5
BART7-3P	BART7	CATCATAGTCCAGTGTCCgGaaA	146475	146497	23	4
BART7-3P	BART7	CATCATAGTCCAGTGTCCgGaGA	146475	146497	23	8

BART7-3P	BART7	CATCATAGTCCAGTGTCCgGGaA	146475	146497	23	29
BART7-3P	BART7	CATCATAGTCCAGTGTCCgGGat	146475	146497	23	4
BART7-3P	BART7	CATCATAGTCCAGTGTCCgGGGA	146475	146497	23	32
BART7-3P	BART7	CATCATAGTCCAGTGTCCgGGGt	146475	146497	23	4
BART7-3P	BART7	CATCATAGTCCAGTGTCCgGGtA	146475	146497	23	3
BART7-3P	BART7	CATCATAGTCCAGTGTCCgGGtc	146475	146497	23	3
BART7-3P	BART7	CATCATAGTCCAGTGTCCgGtaA	146475	146497	23	3
BART7-3P	BART7	CATCATAGTCCAGTGTCCgGtGt	146475	146497	23	6
BART7-3P	BART7	CATCATAGTCCAGTGTCCgGttA	146475	146497	23	6
BART7-3P	BART7	CATCATAGTCCAGTGTCCgtaGt	146475	146497	23	6
BART7-3P	BART7	CATCATAGTCCAGTGTCCgtGGt	146475	146497	23	15
BART7-3P	BART7	CATCATAGTCCAGTGTCCgtGtg	146475	146497	23	3
BART7-3P	BART7	CATCATAGTCCAGTGTCCgttGA	146475	146497	23	3
BART7-3P	BART7	CATCATAGTCCAGTGTCCtacGA	146475	146497	23	9
BART7-3P	BART7	CATCATAGTCCAGTGTCCtaGaA	146475	146497	23	4
BART7-3P	BART7	CATCATAGTCCAGTGTCCtaGag	146475	146497	23	4
BART7-3P	BART7	CATCATAGTCCAGTGTCCtaGGt	146475	146497	23	6
BART7-3P	BART7	CATCATAGTCCAGTGTCCtaGtc	146475	146497	23	4
BART7-3P	BART7	CATCATAGTCCAGTGTCCtcGat	146475	146497	23	4
BART7-3P	BART7	CATCATAGTCCAGTGTCCtGaGA	146475	146497	23	3
BART7-3P	BART7	CATCATAGTCCAGTGTCCtGaGt	146475	146497	23	12
BART7-3P	BART7	CATCATAGTCCAGTGTCCtGGaA	146475	146497	23	6
BART7-3P	BART7	CATCATAGTCCAGTGTCCtGGat	146475	146497	23	11
BART7-3P	BART7	CATCATAGTCCAGTGTCCtGGcA	146475	146497	23	4
BART7-3P	BART7	CATCATAGTCCAGTGTCCtGGGA	146475	146497	23	8
BART7-3P	BART7	CATCATAGTCCAGTGTCCtGGGt	146475	146497	23	63
BART7-3P	BART7	CATCATAGTCCAGTGTCCtGGtt	146475	146497	23	20
BART7-3P	BART7	CATCATAGTCCAGTGTCCttGat	146475	146497	23	15
BART7-3P	BART7	CATCATAGTCCAGTGTCCttGGt	146475	146497	23	3
BART7-3P	BART7	CATCATAGTCCAGTGTCCttGtt	146475	146497	23	12
BART7-3P	BART7	CATCATAGTCCAGTGTCgAGGGA	146475	146497	23	16
BART7-3P	BART7	CATCATAGTCCAGTGTCgAGGGt	146475	146497	23	24
BART7-3P	BART7	CATCATAGTCCAGTGTCtAcGGt	146475	146497	23	4
BART7-3P	BART7	CATCATAGTCCAGTGTCtAGaGt	146475	146497	23	14
BART7-3P	BART7	CATCATAGTCCAGTGTCtAGGaA	146475	146497	23	5
BART7-3P	BART7	CATCATAGTCCAGTGTCtAGGat	146475	146497	23	6
BART7-3P	BART7	CATCATAGTCCAGTGTCtAGGGA	146475	146497	23	29
BART7-3P	BART7	CATCATAGTCCAGTGTCtAGGGc	146475	146497	23	6
BART7-3P	BART7	CATCATAGTCCAGTGTCtAGGGt	146475	146497	23	91
BART7-3P	BART7	CATCATAGTCCAGTGTCtAGGtg	146475	146497	23	12
BART7-3P	BART7	CATCATAGTCCAGTGTCtAGtGt	146475	146497	23	10
BART7-3P	BART7	CATCATAGTCCAGTGTCttGGGt	146475	146497	23	5
BART7-3P	BART7	CgTCATAGTCCAGTGTCCtGGGt	146475	146497	23	7
BART7-3P	BART7	tATCATAGTCCAGTGTCCAGGGA	146475	146497	23	4
BART7-3P	BART7	CAcCATAGTCCAGTGTCCAGGGAt	146475	146498	24	3
BART7-3P	BART7	CATCATAGTCCAGTGTCCAaacAt	146475	146498	24	4
BART7-3P	BART7	CATCATAGTCCAGTGTCCAaaGAa	146475	146498	24	5
BART7-3P	BART7	CATCATAGTCCAGTGTCCAaaGAC	146475	146498	24	3
BART7-3P	BART7	CATCATAGTCCAGTGTCCAaaGAt	146475	146498	24	10
BART7-3P	BART7	CATCATAGTCCAGTGTCCAacaAt	146475	146498	24	24
BART7-3P	BART7	CATCATAGTCCAGTGTCCAacGAt	146475	146498	24	4
BART7-3P	BART7	CATCATAGTCCAGTGTCCAacGgt	146475	146498	24	4
BART7-3P	BART7	CATCATAGTCCAGTGTCCAaGaAt	146475	146498	24	4
BART7-3P	BART7	CATCATAGTCCAGTGTCCAaGcAa	146475	146498	24	5
BART7-3P	BART7	CATCATAGTCCAGTGTCCAaGcAg	146475	146498	24	4
BART7-3P	BART7	CATCATAGTCCAGTGTCCAaGcAt	146475	146498	24	9
BART7-3P	BART7	CATCATAGTCCAGTGTCCAaGGAa	146475	146498	24	36
BART7-3P	BART7	CATCATAGTCCAGTGTCCAaGGAC	146475	146498	24	12
BART7-3P	BART7	CATCATAGTCCAGTGTCCAaGGAg	146475	146498	24	10
BART7-3P	BART7	CATCATAGTCCAGTGTCCAaGGAt	146475	146498	24	39
BART7-3P	BART7	CATCATAGTCCAGTGTCCAaGGcC	146475	146498	24	4
BART7-3P	BART7	CATCATAGTCCAGTGTCCAaGGga	146475	146498	24	20
BART7-3P	BART7	CATCATAGTCCAGTGTCCAaGGgC	146475	146498	24	5
BART7-3P	BART7	CATCATAGTCCAGTGTCCAaGGtC	146475	146498	24	3
BART7-3P	BART7	CATCATAGTCCAGTGTCCAaGGtt	146475	146498	24	8
BART7-3P	BART7	CATCATAGTCCAGTGTCCAatGAt	146475	146498	24	3
BART7-3P	BART7	CATCATAGTCCAGTGTCCAcaGtt	146475	146498	24	3
BART7-3P	BART7	CATCATAGTCCAGTGTCCAccaAa	146475	146498	24	10

BART7-3P	BART7	CATCATAGTCCAGTGTCCAcccAt	146475	146498	24	3
BART7-3P	BART7	CATCATAGTCCAGTGTCCAcGcAa	146475	146498	24	3
BART7-3P	BART7	CATCATAGTCCAGTGTCCAcGGAa	146475	146498	24	11
BART7-3P	BART7	CATCATAGTCCAGTGTCCAcGGgC	146475	146498	24	3
BART7-3P	BART7	CATCATAGTCCAGTGTCCActGAt	146475	146498	24	38
BART7-3P	BART7	CATCATAGTCCAGTGTCCAGaaAa	146475	146498	24	4
BART7-3P	BART7	CATCATAGTCCAGTGTCCAGaaAC	146475	146498	24	4
BART7-3P	BART7	CATCATAGTCCAGTGTCCAGaaAt	146475	146498	24	5
BART7-3P	BART7	CATCATAGTCCAGTGTCCAGacAt	146475	146498	24	6
BART7-3P	BART7	CATCATAGTCCAGTGTCCAGaGAa	146475	146498	24	9
BART7-3P	BART7	CATCATAGTCCAGTGTCCAGaGAg	146475	146498	24	3
BART7-3P	BART7	CATCATAGTCCAGTGTCCAGaGAt	146475	146498	24	32
BART7-3P	BART7	CATCATAGTCCAGTGTCCAGaGca	146475	146498	24	5
BART7-3P	BART7	CATCATAGTCCAGTGTCCAGaGga	146475	146498	24	17
BART7-3P	BART7	CATCATAGTCCAGTGTCCAGaGgt	146475	146498	24	16
BART7-3P	BART7	CATCATAGTCCAGTGTCCAGaGta	146475	146498	24	11
BART7-3P	BART7	CATCATAGTCCAGTGTCCAGaGtg	146475	146498	24	7
BART7-3P	BART7	CATCATAGTCCAGTGTCCAGaGtt	146475	146498	24	10
BART7-3P	BART7	CATCATAGTCCAGTGTCCAGcaAt	146475	146498	24	14
BART7-3P	BART7	CATCATAGTCCAGTGTCCAGccAa	146475	146498	24	8
BART7-3P	BART7	CATCATAGTCCAGTGTCCAGccAC	146475	146498	24	4
BART7-3P	BART7	CATCATAGTCCAGTGTCCAGccAt	146475	146498	24	4
BART7-3P	BART7	CATCATAGTCCAGTGTCCAGcGAa	146475	146498	24	8
BART7-3P	BART7	CATCATAGTCCAGTGTCCAGcGga	146475	146498	24	3
BART7-3P	BART7	CATCATAGTCCAGTGTCCAGcGtt	146475	146498	24	3
BART7-3P	BART7	CATCATAGTCCAGTGTCCAGGaAa	146475	146498	24	51
BART7-3P	BART7	CATCATAGTCCAGTGTCCAGGaAC	146475	146498	24	21
BART7-3P	BART7	CATCATAGTCCAGTGTCCAGGaAg	146475	146498	24	6
BART7-3P	BART7	CATCATAGTCCAGTGTCCAGGaAt	146475	146498	24	88
BART7-3P	BART7	CATCATAGTCCAGTGTCCAGGagC	146475	146498	24	14
BART7-3P	BART7	CATCATAGTCCAGTGTCCAGGatC	146475	146498	24	6
BART7-3P	BART7	CATCATAGTCCAGTGTCCAGGcAC	146475	146498	24	4
BART7-3P	BART7	CATCATAGTCCAGTGTCCAGGcAg	146475	146498	24	6
BART7-3P	BART7	CATCATAGTCCAGTGTCCAGGcAt	146475	146498	24	3
BART7-3P	BART7	CATCATAGTCCAGTGTCCAGGctg	146475	146498	24	4
BART7-3P	BART7	CATCATAGTCCAGTGTCCAGGGAa	146475	146498	24	317
BART7-3P	BART7	CATCATAGTCCAGTGTCCAGGGAC	146475	146498	24	184
BART7-3P	BART7	CATCATAGTCCAGTGTCCAGGGAg	146475	146498	24	47
BART7-3P	BART7	CATCATAGTCCAGTGTCCAGGGAt	146475	146498	24	307
BART7-3P	BART7	CATCATAGTCCAGTGTCCAGGGca	146475	146498	24	35
BART7-3P	BART7	CATCATAGTCCAGTGTCCAGGGcC	146475	146498	24	26
BART7-3P	BART7	CATCATAGTCCAGTGTCCAGGGga	146475	146498	24	46
BART7-3P	BART7	CATCATAGTCCAGTGTCCAGGGgC	146475	146498	24	22
BART7-3P	BART7	CATCATAGTCCAGTGTCCAGGGgg	146475	146498	24	7
BART7-3P	BART7	CATCATAGTCCAGTGTCCAGGGgt	146475	146498	24	28
BART7-3P	BART7	CATCATAGTCCAGTGTCCAGGGta	146475	146498	24	51
BART7-3P	BART7	CATCATAGTCCAGTGTCCAGGGtC	146475	146498	24	53
BART7-3P	BART7	CATCATAGTCCAGTGTCCAGGGtg	146475	146498	24	107
BART7-3P	BART7	CATCATAGTCCAGTGTCCAGGGtt	146475	146498	24	53
BART7-3P	BART7	CATCATAGTCCAGTGTCCAGGtAa	146475	146498	24	12
BART7-3P	BART7	CATCATAGTCCAGTGTCCAGGtAC	146475	146498	24	6
BART7-3P	BART7	CATCATAGTCCAGTGTCCAGGtAg	146475	146498	24	6
BART7-3P	BART7	CATCATAGTCCAGTGTCCAGGtAt	146475	146498	24	7
BART7-3P	BART7	CATCATAGTCCAGTGTCCAGGtgC	146475	146498	24	23
BART7-3P	BART7	CATCATAGTCCAGTGTCCAGtagC	146475	146498	24	8
BART7-3P	BART7	CATCATAGTCCAGTGTCCAGtGAa	146475	146498	24	8
BART7-3P	BART7	CATCATAGTCCAGTGTCCAGtGAC	146475	146498	24	6
BART7-3P	BART7	CATCATAGTCCAGTGTCCAGtGAt	146475	146498	24	7
BART7-3P	BART7	CATCATAGTCCAGTGTCCAGtGga	146475	146498	24	5
BART7-3P	BART7	CATCATAGTCCAGTGTCCAGtGgt	146475	146498	24	17
BART7-3P	BART7	CATCATAGTCCAGTGTCCAGttgC	146475	146498	24	3
BART7-3P	BART7	CATCATAGTCCAGTGTCCAtaGAt	146475	146498	24	4
BART7-3P	BART7	CATCATAGTCCAGTGTCCAtaGgt	146475	146498	24	10
BART7-3P	BART7	CATCATAGTCCAGTGTCCAtGcAa	146475	146498	24	10
BART7-3P	BART7	CATCATAGTCCAGTGTCCAtGGAa	146475	146498	24	4
BART7-3P	BART7	CATCATAGTCCAGTGTCCAtGGAC	146475	146498	24	7
BART7-3P	BART7	CATCATAGTCCAGTGTCCAtGGAg	146475	146498	24	9
BART7-3P	BART7	CATCATAGTCCAGTGTCCAtGGAt	146475	146498	24	14

BART7-3P	BART7	CATCATAGTCCAGTGTCCAtGGga	146475	146498	24	31
BART7-3P	BART7	CATCATAGTCCAGTGTCCAtGGgC	146475	146498	24	6
BART7-3P	BART7	CATCATAGTCCAGTGTCCAtGGgg	146475	146498	24	3
BART7-3P	BART7	CATCATAGTCCAGTGTCCAtGGgt	146475	146498	24	26
BART7-3P	BART7	CATCATAGTCCAGTGTCCAtGGtg	146475	146498	24	7
BART7-3P	BART7	CATCATAGTCCAGTGTCCcGGGAa	146475	146498	24	11
BART7-3P	BART7	CATCATAGTCCAGTGTCCcGGGAt	146475	146498	24	3
BART7-3P	BART7	CATCATAGTCCAGTGTCCcGGGca	146475	146498	24	3
BART7-3P	BART7	CATCATAGTCCAGTGTCCgaGagC	146475	146498	24	5
BART7-3P	BART7	CATCATAGTCCAGTGTCCgaGGAa	146475	146498	24	6
BART7-3P	BART7	CATCATAGTCCAGTGTCCgaGGAt	146475	146498	24	13
BART7-3P	BART7	CATCATAGTCCAGTGTCCgGGatC	146475	146498	24	5
BART7-3P	BART7	CATCATAGTCCAGTGTCCgGGGAa	146475	146498	24	65
BART7-3P	BART7	CATCATAGTCCAGTGTCCgGGGAg	146475	146498	24	3
BART7-3P	BART7	CATCATAGTCCAGTGTCCgGGGAt	146475	146498	24	35
BART7-3P	BART7	CATCATAGTCCAGTGTCCgGGGta	146475	146498	24	5
BART7-3P	BART7	CATCATAGTCCAGTGTCCgGGGtg	146475	146498	24	4
BART7-3P	BART7	CATCATAGTCCAGTGTCCgGGGtt	146475	146498	24	12
BART7-3P	BART7	CATCATAGTCCAGTGTCCgtGGAg	146475	146498	24	34
BART7-3P	BART7	CATCATAGTCCAGTGTCCtGGagC	146475	146498	24	3
BART7-3P	BART7	CATCATAGTCCAGTGTCCtGGGAa	146475	146498	24	22
BART7-3P	BART7	CATCATAGTCCAGTGTCCtGGGAg	146475	146498	24	5
BART7-3P	BART7	CATCATAGTCCAGTGTCCtGGGAt	146475	146498	24	23
BART7-3P	BART7	CATCATAGTCCAGTGTCCtGGGga	146475	146498	24	4
BART7-3P	BART7	CATCATAGTCCAGTGTCCtGGGtt	146475	146498	24	6
BART7-3P	BART7	CATCATAGTCCAGTGTCCttGGAt	146475	146498	24	4
BART7-3P	BART7	CATCATAGTCCAGTGTCtAGGGAa	146475	146498	24	27
BART7-3P	BART7	CgTCATAGTCCAGTGTCCAGGGAt	146475	146498	24	3
BART7-3P	BART7	gATCATAGTCCAGTGTCCAGGGAt	146475	146498	24	13
BART7-3P	BART7	tATCATAGTCCAGTGTCCAGGGAa	146475	146498	24	7
BART7-3P	BART7	tATCATAGTCCAGTGTCCAGGGAt	146475	146498	24	3
BART7-3P	BART7	CATCATAGTCCAGTGTCCAaGGAaA	146475	146499	25	12
BART7-3P	BART7	CATCATAGTCCAGTGTCCAaGGACA	146475	146499	25	3
BART7-3P	BART7	CATCATAGTCCAGTGTCCAaGGACg	146475	146499	25	8
BART7-3P	BART7	CATCATAGTCCAGTGTCCAGaGAaA	146475	146499	25	3
BART7-3P	BART7	CATCATAGTCCAGTGTCCAGaGACA	146475	146499	25	12
BART7-3P	BART7	CATCATAGTCCAGTGTCCAGaGACg	146475	146499	25	9
BART7-3P	BART7	CATCATAGTCCAGTGTCCAGaGAtA	146475	146499	25	3
BART7-3P	BART7	CATCATAGTCCAGTGTCCAGaGtaA	146475	146499	25	3
BART7-3P	BART7	CATCATAGTCCAGTGTCCAGaGttA	146475	146499	25	5
BART7-3P	BART7	CATCATAGTCCAGTGTCCAGccAaA	146475	146499	25	3
BART7-3P	BART7	CATCATAGTCCAGTGTCCAGGaAaA	146475	146499	25	18
BART7-3P	BART7	CATCATAGTCCAGTGTCCAGGaACg	146475	146499	25	24
BART7-3P	BART7	CATCATAGTCCAGTGTCCAGGagaA	146475	146499	25	15
BART7-3P	BART7	CATCATAGTCCAGTGTCCAGGattA	146475	146499	25	3
BART7-3P	BART7	CATCATAGTCCAGTGTCCAGGcAaA	146475	146499	25	10
BART7-3P	BART7	CATCATAGTCCAGTGTCCAGGcAgA	146475	146499	25	3
BART7-3P	BART7	CATCATAGTCCAGTGTCCAGGGAaA	146475	146499	25	214
BART7-3P	BART7	CATCATAGTCCAGTGTCCAGGGAag	146475	146499	25	3
BART7-3P	BART7	CATCATAGTCCAGTGTCCAGGGAat	146475	146499	25	7
BART7-3P	BART7	CATCATAGTCCAGTGTCCAGGGACA	146475	146499	25	76
BART7-3P	BART7	CATCATAGTCCAGTGTCCAGGGACc	146475	146499	25	24
BART7-3P	BART7	CATCATAGTCCAGTGTCCAGGGACg	146475	146499	25	131
BART7-3P	BART7	CATCATAGTCCAGTGTCCAGGGACt	146475	146499	25	17
BART7-3P	BART7	CATCATAGTCCAGTGTCCAGGGAgA	146475	146499	25	16
BART7-3P	BART7	CATCATAGTCCAGTGTCCAGGGAtA	146475	146499	25	224
BART7-3P	BART7	CATCATAGTCCAGTGTCCAGGGAtc	146475	146499	25	11
BART7-3P	BART7	CATCATAGTCCAGTGTCCAGGGAtg	146475	146499	25	5
BART7-3P	BART7	CATCATAGTCCAGTGTCCAGGGcaA	146475	146499	25	6
BART7-3P	BART7	CATCATAGTCCAGTGTCCAGGGcCA	146475	146499	25	7
BART7-3P	BART7	CATCATAGTCCAGTGTCCAGGGgaA	146475	146499	25	11
BART7-3P	BART7	CATCATAGTCCAGTGTCCAGGGgCA	146475	146499	25	3
BART7-3P	BART7	CATCATAGTCCAGTGTCCAGGGgtA	146475	146499	25	6
BART7-3P	BART7	CATCATAGTCCAGTGTCCAGGGtaA	146475	146499	25	49
BART7-3P	BART7	CATCATAGTCCAGTGTCCAGGGtCA	146475	146499	25	20
BART7-3P	BART7	CATCATAGTCCAGTGTCCAGGGtCg	146475	146499	25	3
BART7-3P	BART7	CATCATAGTCCAGTGTCCAGGGtCt	146475	146499	25	3
BART7-3P	BART7	CATCATAGTCCAGTGTCCAGGGtgA	146475	146499	25	11

BART7-3P	BART7	CATCATAGTCCAGTGTCCAGGGttA	146475	146499	25	17
BART7-3P	BART7	CATCATAGTCCAGTGTCCAGGtACA	146475	146499	25	4
BART7-3P	BART7	CATCATAGTCCAGTGTCCAGGtACg	146475	146499	25	5
BART7-3P	BART7	CATCATAGTCCAGTGTCCAGGtAtA	146475	146499	25	3
BART7-3P	BART7	CATCATAGTCCAGTGTCCAGGtcaA	146475	146499	25	3
BART7-3P	BART7	CATCATAGTCCAGTGTCCAGtGAaA	146475	146499	25	19
BART7-3P	BART7	CATCATAGTCCAGTGTCCAGtGACg	146475	146499	25	13
BART7-3P	BART7	CATCATAGTCCAGTGTCCAGaGAaAa	146475	146500	26	4
BART7-3P	BART7	CATCATAGTCCAGTGTCCAGaGAtAa	146475	146500	26	3
BART7-3P	BART7	CATCATAGTCCAGTGTCCAGGaAaAG	146475	146500	26	4
BART7-3P	BART7	CATCATAGTCCAGTGTCCAGGaACAG	146475	146500	26	3
BART7-3P	BART7	CATCATAGTCCAGTGTCCAGGagCga	146475	146500	26	3
BART7-3P	BART7	CATCATAGTCCAGTGTCCAGGcAgAG	146475	146500	26	4
BART7-3P	BART7	CATCATAGTCCAGTGTCCAGGGAaAa	146475	146500	26	5
BART7-3P	BART7	CATCATAGTCCAGTGTCCAGGGAaAG	146475	146500	26	24
BART7-3P	BART7	CATCATAGTCCAGTGTCCAGGGAaAt	146475	146500	26	4
BART7-3P	BART7	CATCATAGTCCAGTGTCCAGGGACAa	146475	146500	26	3
BART7-3P	BART7	CATCATAGTCCAGTGTCCAGGGACAc	146475	146500	26	8
BART7-3P	BART7	CATCATAGTCCAGTGTCCAGGGACAG	146475	146500	26	35
BART7-3P	BART7	CATCATAGTCCAGTGTCCAGGGACcG	146475	146500	26	8
BART7-3P	BART7	CATCATAGTCCAGTGTCCAGGGACtc	146475	146500	26	6
BART7-3P	BART7	CATCATAGTCCAGTGTCCAGGGACtG	146475	146500	26	3
BART7-3P	BART7	CATCATAGTCCAGTGTCCAGGGAgAG	146475	146500	26	3
BART7-3P	BART7	CATCATAGTCCAGTGTCCAGGGAtAa	146475	146500	26	8
BART7-3P	BART7	CATCATAGTCCAGTGTCCAGGGAtAG	146475	146500	26	41
BART7-3P	BART7	CATCATAGTCCAGTGTCCAGGGcaAt	146475	146500	26	3
BART7-3P	BART7	CATCATAGTCCAGTGTCCAGGGgaAG	146475	146500	26	5
BART7-3P	BART7	CATCATAGTCCAGTGTCCAGGGtaAa	146475	146500	26	5
BART7-3P	BART7	CATCATAGTCCAGTGTCCAGGGtaAG	146475	146500	26	12
BART7-3P	BART7	CATCATAGTCCAGTGTCCAGGGttAG	146475	146500	26	4
BART7-3P	BART7	CATCATAGTCCAGTGTCCAGGaAaAGT	146475	146501	27	4
BART7-3P	BART7	CATCATAGTCCAGTGTCCAGGGAaAGT	146475	146501	27	11
BART7-3P	BART7	CATCATAGTCCAGTGTCCAGGGACAaT	146475	146501	27	27
BART7-3P	BART7	CATCATAGTCCAGTGTCCAGGGACAGT	146475	146501	27	14
BART7-3P	BART7	CATCATAGTCCAGTGTCCAGGGACAtT	146475	146501	27	4
BART7-3P	BART7	CATCATAGTCCAGTGTCCAGGGAgAaT	146475	146501	27	4
BART7-3P	BART7	CATCATAGTCCAGTGTCCAGGGAtAGT	146475	146501	27	7
BART7-3P	BART7	CATCATAGTCCAGTGTCCAGGGAtAta	146475	146501	27	3
BART7-3P	BART7	CATCATAGTCCAGTGTCCAGGGtaAGT	146475	146501	27	8
BART7-3P	BART7	CATCATAGTCCAGTGTCCAGGGtagGT	146475	146501	27	3
BART7-3P	BART7	CATCATAGTCCAGTGTCCAGGGttAGg	146475	146501	27	17
BART7-3P	BART7	CATCATAGTCCAGTGTCCAGGGAaAaaa	146475	146502	28	4
BART7-3P	BART7	CATCATAGTCCAGTGTCCAGGGAaAact	146475	146502	28	4
BART7-3P	BART7	CATCATAGTCCAGTGTCCAGGGAaAcaG	146475	146502	28	3
BART7-3P	BART7	CATCATAGTCCAGTGTCCAGGGAaAGac	146475	146502	28	21
BART7-3P	BART7	ATCATAGTCCAGTGTCCAGGGAaA	146476	146499	24	9
BART7-3P	BART7	ATCATAGTCCAGTGTCCAGGGAtA	146476	146499	24	4
BART8-5P	BART8	TACGGTTTCCTAGATaacA	146772	146790	19	4
BART8-5P	BART8	TACGGTTTCCTAGATTccA	146772	146790	19	8
BART8-5P	BART8	TACGGTTTCCTAGATTGcA	146772	146790	19	16
BART8-5P	BART8	TACGGTTTCCTAGATTGTA	146772	146790	19	10
BART8-5P	BART8	TACGGTTTCCTAGAaTGTAC	146772	146791	20	7
BART8-5P	BART8	TACGGTTTCCTAGAcTGTAC	146772	146791	20	4
BART8-5P	BART8	TACGGTTTCCTAGAgTGTAC	146772	146791	20	4
BART8-5P	BART8	TACGGTTTCCTAGATaGTAC	146772	146791	20	3
BART8-5P	BART8	TACGGTTTCCTAGATgGTAC	146772	146791	20	24
BART8-5P	BART8	TACGGTTTCCTAGATTacAC	146772	146791	20	4
BART8-5P	BART8	TACGGTTTCCTAGATTacAg	146772	146791	20	15
BART8-5P	BART8	TACGGTTTCCTAGATTaTAC	146772	146791	20	4
BART8-5P	BART8	TACGGTTTCCTAGATTGTAC	146772	146791	20	107
BART8-5P	BART8	TACGGTTTCCTAGATTGTca	146772	146791	20	13
BART8-5P	BART8	TACGGTTTCCTAGATaGTACA	146772	146792	21	59
BART8-5P	BART8	TACGGTTTCCTAGATcaTACA	146772	146792	21	8
BART8-5P	BART8	TACGGTTTCCTAGATcGTACA	146772	146792	21	19
BART8-5P	BART8	TACGGTTTCCTAGATgGTACA	146772	146792	21	15
BART8-5P	BART8	TACGGTTTCCTAGATTaaACA	146772	146792	21	5
BART8-5P	BART8	TACGGTTTCCTAGATTacACA	146772	146792	21	8
BART8-5P	BART8	TACGGTTTCCTAGATTaccCA	146772	146792	21	3

BART8-5P	BART8	TACGGTTTCCTAGATTagACA	146772	146792	21	4
BART8-5P	BART8	TACGGTTTCCTAGATTaTACA	146772	146792	21	8
BART8-5P	BART8	TACGGTTTCCTAGATTaTcCA	146772	146792	21	24
BART8-5P	BART8	TACGGTTTCCTAGATTccACA	146772	146792	21	4
BART8-5P	BART8	TACGGTTTCCTAGATTGaACA	146772	146792	21	3
BART8-5P	BART8	TACGGTTTCCTAGATTGacCA	146772	146792	21	3
BART8-5P	BART8	TACGGTTTCCTAGATTGatCA	146772	146792	21	3
BART8-5P	BART8	TACGGTTTCCTAGATTGcACA	146772	146792	21	10
BART8-5P	BART8	TACGGTTTCCTAGATTGgACA	146772	146792	21	3
BART8-5P	BART8	TACGGTTTCCTAGATTGTAaA	146772	146792	21	3
BART8-5P	BART8	TACGGTTTCCTAGATTGTACA	146772	146792	21	104
BART8-5P	BART8	TACGGTTTCCTAGATTGTAgA	146772	146792	21	3
BART8-5P	BART8	TACGGTTTCCTAGATTGTgtc	146772	146792	21	21
BART8-5P	BART8	TACGGTTTCCTAGATTagACAG	146772	146793	22	3
BART8-5P	BART8	TACGGTTTCCTAGATTaTACAG	146772	146793	22	90
BART8-5P	BART8	TACGGTTTCCTAGATTGaACAG	146772	146793	22	56
BART8-5P	BART8	TACGGTTTCCTAGATTGcACAc	146772	146793	22	13
BART8-5P	BART8	TACGGTTTCCTAGATTGcACAG	146772	146793	22	6
BART8-5P	BART8	TACGGTTTCCTAGATTGgACAG	146772	146793	22	12
BART8-5P	BART8	TACGGTTTCCTAGATTGgACtG	146772	146793	22	3
BART8-5P	BART8	TACGGTTTCCTAGATTGTAact	146772	146793	22	10
BART8-5P	BART8	TACGGTTTCCTAGATTGTACAc	146772	146793	22	64
BART8-5P	BART8	TACGGTTTCCTAGATTGTACAG	146772	146793	22	1532
BART8-5P	BART8	TACGGTTTCCTAGATTGTACAt	146772	146793	22	23
BART8-5P	BART8	TACGGTTTCCTAGATTGTACtG	146772	146793	22	6
BART8-5P	BART8	TACGGTTTCCTAGATTGTAgtG	146772	146793	22	6
BART8-5P	BART8	TACGGTTTCCTAGATTGTgCAG	146772	146793	22	3
BART8-5P	BART8	TACGGTTTCCTAGATTGTgCtc	146772	146793	22	3
BART8-5P	BART8	TACGGTTTCCTAGATTGTgtAG	146772	146793	22	7
BART8-5P	BART8	TACGGTTTCCTAGATTGTtCAc	146772	146793	22	3
BART8-5P	BART8	TcCGGTTTCCTAGATTGTACAG	146772	146793	22	4
BART8-5P	BART8	TACGGTTTCCTAGATTGgACAGt	146772	146794	23	6
BART8-5P	BART8	TACGGTTTCCTAGATTGTAagcA	146772	146794	23	3
BART8-5P	BART8	TACGGTTTCCTAGATTGTACAcA	146772	146794	23	3
BART8-5P	BART8	TACGGTTTCCTAGATTGTACAGA	146772	146794	23	41
BART8-5P	BART8	TACGGTTTCCTAGATTGTACAGc	146772	146794	23	3
BART8-5P	BART8	TACGGTTTCCTAGATTGTACAGt	146772	146794	23	10
BART8-5P	BART8	TACGGTTTCCTAGATTGTACtcA	146772	146794	23	7
BART8-5P	BART8	TACGGTTTCCTAGATTGTACAcAg	146772	146795	24	4
BART8-5P	BART8	TACGGTTTCCTAGATTGTACAccT	146772	146795	24	12
BART8-5P	BART8	TACGGTTTCCTAGATTGTACActT	146772	146795	24	5
BART8-5P	BART8	TACGGTTTCCTAGATTGTACAGAg	146772	146795	24	5
BART8-5P	BART8	TACGGTTTCCTAGATTGTACAGATG	146772	146796	25	4
BART8-5P	BART8	ACGGTTTCCTAGATTGTACAG	146773	146793	21	18
BART8-3P	BART8-star	GTCACAATCTATGGGGTCG	146807	146825	19	30
BART8-3P	BART8-star	GTCACAATCTATGGGGTCt	146807	146825	19	15
BART8-3P	BART8-star	GTCACAATCTATGGGacCGT	146807	146826	20	3
BART8-3P	BART8-star	GTCACAATCTATGGGaTCGT	146807	146826	20	5
BART8-3P	BART8-star	GTCACAATCTATGGGGaaGa	146807	146826	20	8
BART8-3P	BART8-star	GTCACAATCTATGGGGaCag	146807	146826	20	11
BART8-3P	BART8-star	GTCACAATCTATGGGGcCGa	146807	146826	20	4
BART8-3P	BART8-star	GTCACAATCTATGGGGcCGT	146807	146826	20	10
BART8-3P	BART8-star	GTCACAATCTATGGGGgaGa	146807	146826	20	4
BART8-3P	BART8-star	GTCACAATCTATGGGGTaGa	146807	146826	20	3
BART8-3P	BART8-star	GTCACAATCTATGGGGTCaT	146807	146826	20	6
BART8-3P	BART8-star	GTCACAATCTATGGGGTCGT	146807	146826	20	35
BART8-3P	BART8-star	GTCACAATCTATGGGGTgcT	146807	146826	20	25
BART8-3P	BART8-star	GTCACAATCTATGGGaTCagA	146807	146827	21	5
BART8-3P	BART8-star	GTCACAATCTATGGGaTCaTA	146807	146827	21	12
BART8-3P	BART8-star	GTCACAATCTATGGGGaCagA	146807	146827	21	27
BART8-3P	BART8-star	GTCACAATCTATGGGGaCGgA	146807	146827	21	9
BART8-3P	BART8-star	GTCACAATCTATGGGGcCagA	146807	146827	21	9
BART8-3P	BART8-star	GTCACAATCTATGGGGcCGTA	146807	146827	21	9
BART8-3P	BART8-star	GTCACAATCTATGGGGcCtaA	146807	146827	21	6
BART8-3P	BART8-star	GTCACAATCTATGGGGcCtgA	146807	146827	21	5
BART8-3P	BART8-star	GTCACAATCTATGGGGgCagA	146807	146827	21	6
BART8-3P	BART8-star	GTCACAATCTATGGGGTaagA	146807	146827	21	14
BART8-3P	BART8-star	GTCACAATCTATGGGGTCagA	146807	146827	21	21

BART8-3P	BART8-star	GTCACAATCTATGGGGTCaTA	146807	146827	21	23
BART8-3P	BART8-star	GTCACAATCTATGGGGTCaTc	146807	146827	21	26
BART8-3P	BART8-star	GTCACAATCTATGGGGTCcaA	146807	146827	21	3
BART8-3P	BART8-star	GTCACAATCTATGGGGTCcgA	146807	146827	21	13
BART8-3P	BART8-star	GTCACAATCTATGGGGTCcTA	146807	146827	21	39
BART8-3P	BART8-star	GTCACAATCTATGGGGTCGaA	146807	146827	21	7
BART8-3P	BART8-star	GTCACAATCTATGGGGTCGag	146807	146827	21	13
BART8-3P	BART8-star	GTCACAATCTATGGGGTCGcA	146807	146827	21	7
BART8-3P	BART8-star	GTCACAATCTATGGGGTCGct	146807	146827	21	3
BART8-3P	BART8-star	GTCACAATCTATGGGGTCGgA	146807	146827	21	3
BART8-3P	BART8-star	GTCACAATCTATGGGGTCGTA	146807	146827	21	81
BART8-3P	BART8-star	GTCACAATCTATGGGGTCGTc	146807	146827	21	13
BART8-3P	BART8-star	GTCACAATCTATGGGGTCGTg	146807	146827	21	6
BART8-3P	BART8-star	GTCACAATCTATGGGGTCtct	146807	146827	21	7
BART8-3P	BART8-star	GTCACAATCTATGGGGTCtgA	146807	146827	21	4
BART8-3P	BART8-star	GTCACAATCTATGGGGTgagA	146807	146827	21	8
BART8-3P	BART8-star	GTCACAATCTATGGGGTgcTA	146807	146827	21	114
BART8-3P	BART8-star	GTCACAATCTATGGGGTtagA	146807	146827	21	24
BART8-3P	BART8-star	GTCACAATCTATGGGGTtGgA	146807	146827	21	3
BART8-3P	BART8-star	GTCACAATCTATGGGGaCGTAc	146807	146828	22	19
BART8-3P	BART8-star	GTCACAATCTATGGGGaCGTAG	146807	146828	22	22
BART8-3P	BART8-star	GTCACAATCTATGGGGaCGTct	146807	146828	22	3
BART8-3P	BART8-star	GTCACAATCTATGGGGagGTAG	146807	146828	22	5
BART8-3P	BART8-star	GTCACAATCTATGGGGcCGatc	146807	146828	22	3
BART8-3P	BART8-star	GTCACAATCTATGGGGcCGTAc	146807	146828	22	10
BART8-3P	BART8-star	GTCACAATCTATGGGGcCGTAG	146807	146828	22	23
BART8-3P	BART8-star	GTCACAATCTATGGGGcCGTAt	146807	146828	22	3
BART8-3P	BART8-star	GTCACAATCTATGGGGcCGTct	146807	146828	22	3
BART8-3P	BART8-star	GTCACAATCTATGGGGcCGTtc	146807	146828	22	3
BART8-3P	BART8-star	GTCACAATCTATGGGGgCGTAc	146807	146828	22	8
BART8-3P	BART8-star	GTCACAATCTATGGGGTaaTAG	146807	146828	22	3
BART8-3P	BART8-star	GTCACAATCTATGGGGTaGTAc	146807	146828	22	27
BART8-3P	BART8-star	GTCACAATCTATGGGGTCacAc	146807	146828	22	3
BART8-3P	BART8-star	GTCACAATCTATGGGGTCacgG	146807	146828	22	3
BART8-3P	BART8-star	GTCACAATCTATGGGGTCagAG	146807	146828	22	5
BART8-3P	BART8-star	GTCACAATCTATGGGGTCaTAc	146807	146828	22	19
BART8-3P	BART8-star	GTCACAATCTATGGGGTCaTAG	146807	146828	22	8
BART8-3P	BART8-star	GTCACAATCTATGGGGTCcatG	146807	146828	22	3
BART8-3P	BART8-star	GTCACAATCTATGGGGTCGaAc	146807	146828	22	5
BART8-3P	BART8-star	GTCACAATCTATGGGGTCGaAG	146807	146828	22	4
BART8-3P	BART8-star	GTCACAATCTATGGGGTCGTAc	146807	146828	22	316
BART8-3P	BART8-star	GTCACAATCTATGGGGTCGTAG	146807	146828	22	76
BART8-3P	BART8-star	GTCACAATCTATGGGGTCGTAt	146807	146828	22	18
BART8-3P	BART8-star	GTCACAATCTATGGGGTCGTca	146807	146828	22	5
BART8-3P	BART8-star	GTCACAATCTATGGGGTCGTct	146807	146828	22	5
BART8-3P	BART8-star	GTCACAATCTATGGGGTCGTgt	146807	146828	22	3
BART8-3P	BART8-star	GTCACAATCTATGGGGTCGTtc	146807	146828	22	3
BART8-3P	BART8-star	GTCACAATCTATGGGGTCGTtG	146807	146828	22	4
BART8-3P	BART8-star	GTCACAATCTATGGGGTCtTAc	146807	146828	22	4
BART8-3P	BART8-star	GTCACAATCTATGGGGTgGTAc	146807	146828	22	21
BART8-3P	BART8-star	GTCACAATCTATGGGGTgGTAG	146807	146828	22	6
BART8-3P	BART8-star	GTCACAATCTATGGGGTtGctc	146807	146828	22	4
BART8-3P	BART8-star	GTCACAATCTATGGGGTtGTAc	146807	146828	22	18
BART8-3P	BART8-star	aTCACAATCTATGGGGTCaTAGA	146807	146829	23	9
BART8-3P	BART8-star	aTCACAATCTATGGGGTCGcAGA	146807	146829	23	4
BART8-3P	BART8-star	aTCACAATCTATGGGGTCGTAGA	146807	146829	23	5
BART8-3P	BART8-star	GcCACAATCTATGGGGTCGTAGA	146807	146829	23	3
BART8-3P	BART8-star	GTCACAATCTATGGGGTaGaAGA	146807	146829	23	3
BART8-3P	BART8-star	GTCACAATCTATGGGGTaGcAGA	146807	146829	23	8
BART8-3P	BART8-star	GTCACAATCTATGGGGTaGTAGA	146807	146829	23	246
BART8-3P	BART8-star	GTCACAATCTATGGGGTaGTAGt	146807	146829	23	19
BART8-3P	BART8-star	GTCACAATCTATGGGGTCaaAGA	146807	146829	23	5
BART8-3P	BART8-star	GTCACAATCTATGGGGTCacAGA	146807	146829	23	32
BART8-3P	BART8-star	GTCACAATCTATGGGGTCacAGt	146807	146829	23	3
BART8-3P	BART8-star	GTCACAATCTATGGGGTCaccGA	146807	146829	23	5
BART8-3P	BART8-star	GTCACAATCTATGGGGTCagAGA	146807	146829	23	30
BART8-3P	BART8-star	GTCACAATCTATGGGGTCaTAaA	146807	146829	23	5
BART8-3P	BART8-star	GTCACAATCTATGGGGTCaTAGA	146807	146829	23	148

BART8-3P	BART8-star	GTCACAATCTATGGGGTCaTcGA	146807	146829	23	5
BART8-3P	BART8-star	GTCACAATCTATGGGGTCaTtGA	146807	146829	23	9
BART8-3P	BART8-star	GTCACAATCTATGGGGTCccAGA	146807	146829	23	9
BART8-3P	BART8-star	GTCACAATCTATGGGGTCcctGA	146807	146829	23	4
BART8-3P	BART8-star	GTCACAATCTATGGGGTCcTAGA	146807	146829	23	35
BART8-3P	BART8-star	GTCACAATCTATGGGGTCGaAGA	146807	146829	23	41
BART8-3P	BART8-star	GTCACAATCTATGGGGTCGacaA	146807	146829	23	3
BART8-3P	BART8-star	GTCACAATCTATGGGGTCGagGA	146807	146829	23	15
BART8-3P	BART8-star	GTCACAATCTATGGGGTCGatGA	146807	146829	23	14
BART8-3P	BART8-star	GTCACAATCTATGGGGTCGcAGA	146807	146829	23	81
BART8-3P	BART8-star	GTCACAATCTATGGGGTCGccGA	146807	146829	23	4
BART8-3P	BART8-star	GTCACAATCTATGGGGTCGcgaA	146807	146829	23	8
BART8-3P	BART8-star	GTCACAATCTATGGGGTCGgAGA	146807	146829	23	51
BART8-3P	BART8-star	GTCACAATCTATGGGGTCGTAaA	146807	146829	23	10
BART8-3P	BART8-star	GTCACAATCTATGGGGTCGTAag	146807	146829	23	43
BART8-3P	BART8-star	GTCACAATCTATGGGGTCGTAat	146807	146829	23	4
BART8-3P	BART8-star	GTCACAATCTATGGGGTCGTAcA	146807	146829	23	5
BART8-3P	BART8-star	GTCACAATCTATGGGGTCGTAct	146807	146829	23	4
BART8-3P	BART8-star	GTCACAATCTATGGGGTCGTAGA	146807	146829	23	1499
BART8-3P	BART8-star	GTCACAATCTATGGGGTCGTAGc	146807	146829	23	42
BART8-3P	BART8-star	GTCACAATCTATGGGGTCGTAGg	146807	146829	23	18
BART8-3P	BART8-star	GTCACAATCTATGGGGTCGTAGt	146807	146829	23	196
BART8-3P	BART8-star	GTCACAATCTATGGGGTCGTAtc	146807	146829	23	4
BART8-3P	BART8-star	GTCACAATCTATGGGGTCGTcGA	146807	146829	23	5
BART8-3P	BART8-star	GTCACAATCTATGGGGTCGTgag	146807	146829	23	15
BART8-3P	BART8-star	GTCACAATCTATGGGGTCGTgGA	146807	146829	23	10
BART8-3P	BART8-star	GTCACAATCTATGGGGTCGTtGA	146807	146829	23	24
BART8-3P	BART8-star	GTCACAATCTATGGGGTCtaAGA	146807	146829	23	11
BART8-3P	BART8-star	GTCACAATCTATGGGGTCtgAGA	146807	146829	23	19
BART8-3P	BART8-star	GTCACAATCTATGGGGTCtgAGc	146807	146829	23	4
BART8-3P	BART8-star	GTCACAATCTATGGGGTCtTAGA	146807	146829	23	14
BART8-3P	BART8-star	GTCACAATCTATGGGGTCtTcGA	146807	146829	23	7
BART8-3P	BART8-star	GTCACAATCTATGGGGTgGTAGA	146807	146829	23	19
BART8-3P	BART8-star	GTCACAATCTATGGGGTtaTAGA	146807	146829	23	12
BART8-3P	BART8-star	GTCACAATCTATGGGGTtGaAGA	146807	146829	23	15
BART8-3P	BART8-star	GTCACAATCTATGGGGTtGcAGA	146807	146829	23	7
BART8-3P	BART8-star	GTCACAATCTATGGGGTtGTAcA	146807	146829	23	3
BART8-3P	BART8-star	GTCACAATCTATGGGGTtGTAGA	146807	146829	23	114
BART8-3P	BART8-star	GTCACAATCTATGGGGTtGTAGt	146807	146829	23	23
BART8-3P	BART8-star	GTCACAATCTtTGGGGTCGTAGA	146807	146829	23	3
BART8-3P	BART8-star	GTCAtAATCTATGGGGTCGTAGA	146807	146829	23	3
BART8-3P	BART8-star	GTCACAATCTATGGGGTCaTAGga	146807	146830	24	12
BART8-3P	BART8-star	GTCACAATCTATGGGGTCaTAGgt	146807	146830	24	3
BART8-3P	BART8-star	GTCACAATCTATGGGGTCGaActC	146807	146830	24	5
BART8-3P	BART8-star	GTCACAATCTATGGGGTCGaAGAa	146807	146830	24	9
BART8-3P	BART8-star	GTCACAATCTATGGGGTCGcAGga	146807	146830	24	3
BART8-3P	BART8-star	GTCACAATCTATGGGGTCGcAGgt	146807	146830	24	5
BART8-3P	BART8-star	GTCACAATCTATGGGGTCGTAaAC	146807	146830	24	3
BART8-3P	BART8-star	GTCACAATCTATGGGGTCGTAcAC	146807	146830	24	9
BART8-3P	BART8-star	GTCACAATCTATGGGGTCGTAcAg	146807	146830	24	5
BART8-3P	BART8-star	GTCACAATCTATGGGGTCGTAccC	146807	146830	24	6
BART8-3P	BART8-star	GTCACAATCTATGGGGTCGTActa	146807	146830	24	8
BART8-3P	BART8-star	GTCACAATCTATGGGGTCGTActC	146807	146830	24	24
BART8-3P	BART8-star	GTCACAATCTATGGGGTCGTActg	146807	146830	24	4
BART8-3P	BART8-star	GTCACAATCTATGGGGTCGTAGAa	146807	146830	24	94
BART8-3P	BART8-star	GTCACAATCTATGGGGTCGTAGAC	146807	146830	24	56
BART8-3P	BART8-star	GTCACAATCTATGGGGTCGTAGAg	146807	146830	24	39
BART8-3P	BART8-star	GTCACAATCTATGGGGTCGTAGAt	146807	146830	24	17
BART8-3P	BART8-star	GTCACAATCTATGGGGTCGTAGca	146807	146830	24	7
BART8-3P	BART8-star	GTCACAATCTATGGGGTCGTAGcC	146807	146830	24	3
BART8-3P	BART8-star	GTCACAATCTATGGGGTCGTAGga	146807	146830	24	98
BART8-3P	BART8-star	GTCACAATCTATGGGGTCGTAGgt	146807	146830	24	9
BART8-3P	BART8-star	GTCACAATCTATGGGGTCGTAGtC	146807	146830	24	31
BART8-3P	BART8-star	GTCACAATCTATGGGGTCGTAGtt	146807	146830	24	11
BART8-3P	BART8-star	GTCACAATCTATGGGGTCGTAGAaA	146807	146831	25	7
BART8-3P	BART8-star	GTCACAATCTATGGGGTCGTAGACA	146807	146831	25	17
BART8-3P	BART8-star	GTCACAATCTATGGGGTCGTAGACg	146807	146831	25	11
BART8-3P	BART8-star	GTCACAATCTATGGGGTCGTAGAgA	146807	146831	25	17

BART8-3P	BART8-star	GTCACAATCTATGGGGTCGTAGAtA	146807	146831	25	3
BART8-3P	BART8-star	GTCACAATCTATGGGGTCGTAGcgA	146807	146831	25	3
BART8-3P	BART8-star	GTCACAATCTATGGGGTCGTAGtgA	146807	146831	25	3
BART8-3P	BART8-star	GTCACAATCTATGGGGTCGTAGACAG	146807	146832	26	8
BART8-3P	BART8-star	GTCACAATCTATGGGGTCGTAGACga	146807	146832	26	9
BART8-3P	BART8-star	GTCACAATCTATGGGGTCGTAGACtc	146807	146832	26	7
BART8-3P	BART8-star	GTCACAATCTATGGGGTCGTAGAgAG	146807	146832	26	6
BART8-3P	BART8-star	TCACAATCTATGGGGTCaTA	146808	146827	20	3
BART8-3P	BART8-star	TCACAATCTATGGGGTCGTAG	146808	146828	21	8
BART8-3P	BART8-star	TCACAATCTATGGGGTgGTAG	146808	146828	21	3
BART8-3P	BART8-star	cCACAATCTATGGGGTCGaAGA	146808	146829	22	3
BART8-3P	BART8-star	TCACAATCTATGGGGTCaTAGA	146808	146829	22	3
BART8-3P	BART8-star	TCACAATCTATGGGGTCGTAGA	146808	146829	22	12
BART8-3P	BART8-star	TCACAATCTATGGGGTtGTAGA	146808	146829	22	4
BART9-5P	BART9-star	TACTGGACCCTGAATTGGA	146959	146977	19	8
BART9-5P	BART9-star	TACTGGACCCTGAATTGtc	146959	146977	19	8
BART9-5P	BART9-star	TACTGGACCCTGAATaGGAA	146959	146978	20	4
BART9-5P	BART9-star	TACTGGACCCTGAATTcGAA	146959	146978	20	3
BART9-5P	BART9-star	TACTGGACCCTGAATTGGAA	146959	146978	20	11
BART9-5P	BART9-star	TACTGGACCCTGAATaGGAAA	146959	146979	21	3
BART9-5P	BART9-star	TACTGGACCCTGAATgaGAAA	146959	146979	21	8
BART9-5P	BART9-star	TACTGGACCCTGAATgGGAAA	146959	146979	21	3
BART9-5P	BART9-star	TACTGGACCCTGAATTGGAAA	146959	146979	21	21
BART9-5P	BART9-star	TACTGGACCCTGAATTGGAAAa	146959	146980	22	16
BART9-5P	BART9-star	TACTGGACCCTGAATTGGAAAC	146959	146980	22	29
BART9-5P	BART9-star	TACTGGACCCTGAATTGGAgAC	146959	146980	22	3
BART9-5P	BART9-star	TACTGGACCCTGAATTGGttgC	146959	146980	22	6
BART9-5P	BART9-star	TACTGGACCCTGAATTGaAAACA	146959	146981	23	20
BART9-5P	BART9-star	TACTGGACCCTGAATTGaAAgCA	146959	146981	23	3
BART9-5P	BART9-star	TACTGGACCCTGAATTGGAAACA	146959	146981	23	23
BART9-5P	BART9-star	TACTGGACCCTGAATTGGgAACA	146959	146981	23	4
BART9-5P	BART9-star	TACTGGACCCTGAATTGGAAACAG	146959	146982	24	5
BART9-5P	BART9-star	TACTGGACCCTGAATTGGAgACAG	146959	146982	24	5
BART9-5P	BART9-star	TACTGGACCCTGAATTGGAAACAGg	146959	146983	25	4
BART9-5P	BART9-star	ACTGGACCCTGAATTGGAAACA	146960	146981	22	4
BART9-3P	BART9	GTAACACTTCATGGGTCCCG	146996	147015	20	7
BART9-3P	BART9	GTAACACTTCATGGGaCCCGT	146996	147016	21	7
BART9-3P	BART9	GTAACACTTCATGGGcaCCGT	146996	147016	21	4
BART9-3P	BART9	GTAACACTTCATGGGcCCCGT	146996	147016	21	9
BART9-3P	BART9	GTAACACTTCATGGGTCCCcT	146996	147016	21	6
BART9-3P	BART9	GTAACACTTCATGGGTCCCGc	146996	147016	21	7
BART9-3P	BART9	GTAACACTTCATGGGTCCCGT	146996	147016	21	31
BART9-3P	BART9	GTAACACTTCATGGGTaCCGTA	146996	147017	22	4
BART9-3P	BART9	GTAACACTTCATGGGTCCCacA	146996	147017	22	4
BART9-3P	BART9	GTAACACTTCATGGGTCCCaTA	146996	147017	22	39
BART9-3P	BART9	GTAACACTTCATGGGTCCCaTc	146996	147017	22	3
BART9-3P	BART9	GTAACACTTCATGGGTCCCcat	146996	147017	22	3
BART9-3P	BART9	GTAACACTTCATGGGTCCCGaA	146996	147017	22	20
BART9-3P	BART9	GTAACACTTCATGGGTCCCGcA	146996	147017	22	13
BART9-3P	BART9	GTAACACTTCATGGGTCCCGTA	146996	147017	22	45
BART9-3P	BART9	GTAACACTTCATGGGTCCgGTA	146996	147017	22	10
BART9-3P	BART9	GTAACACTTCATGGGTgCCGTA	146996	147017	22	4
BART9-3P	BART9	aTAACACTTCATGGGTCCCGTAG	146996	147018	23	15
BART9-3P	BART9	GTAACACTTCATGGGTCCCaTAa	146996	147018	23	4
BART9-3P	BART9	GTAACACTTCATGGGTCCCGaAa	146996	147018	23	3
BART9-3P	BART9	GTAACACTTCATGGGTCCCGTAa	146996	147018	23	56
BART9-3P	BART9	GTAACACTTCATGGGTCCCGTAc	146996	147018	23	3
BART9-3P	BART9	GTAACACTTCATGGGTCCCGTAG	146996	147018	23	32
BART9-3P	BART9	GTAACACTTCATGGGTCCCGTAt	146996	147018	23	26
BART9-3P	BART9	GTAACACTTCATGGGTCgCGTAG	146996	147018	23	5
BART9-3P	BART9	GTAACACTTCATGGGTCgCGTAt	146996	147018	23	6
BART9-3P	BART9	GTAACACTTCATGGGTCtCGTAG	146996	147018	23	7
BART9-3P	BART9	GTAACACTTCATGGtgaCCGTAG	146996	147018	23	5
BART9-3P	BART9	aTAACACTTCATGGGTCCCGTAGT	146996	147019	24	6
BART9-3P	BART9	GTAACACTTCATGGGTCCCGcAGT	146996	147019	24	8
BART9-3P	BART9	GTAACACTTCATGGGTCCCGTAaT	146996	147019	24	4
BART9-3P	BART9	GTAACACTTCATGGGTCCCGTAGa	146996	147019	24	4
BART9-3P	BART9	GTAACACTTCATGGGTCCCGTAGc	146996	147019	24	7

BART9-3P	BART9	GTAACACTTCATGGGTCCCGTAGT	146996	147019	24	72
BART9-3P	BART9	GTAACACTTCATGGGTCCCaTAGTG	146996	147020	25	3
BART9-3P	BART9	GTAACACTTCATGGGTCCCGTAGTG	146996	147020	25	43
BART9-3P	BART9	TAACACTTCATGGaTCCCG	146997	147015	19	21
BART9-3P	BART9	TAACACTTCATGGGaCCCG	146997	147015	19	12
BART9-3P	BART9	TAACACTTCATGGGcCCCG	146997	147015	19	8
BART9-3P	BART9	TAACACTTCATGGGTaCCG	146997	147015	19	5
BART9-3P	BART9	TAACACTTCATGGGTCCCa	146997	147015	19	22
BART9-3P	BART9	TAACACTTCATGGGTCCCG	146997	147015	19	126
BART9-3P	BART9	TAACACTTCATGGtTCCCG	146997	147015	19	4
BART9-3P	BART9	TAACACTTCATGGGaCCCGT	146997	147016	20	31
BART9-3P	BART9	TAACACTTCATGGGatCCGT	146997	147016	20	5
BART9-3P	BART9	TAACACTTCATGGGcCCCaT	146997	147016	20	4
BART9-3P	BART9	TAACACTTCATGGGcCCCGT	146997	147016	20	10
BART9-3P	BART9	TAACACTTCATGGGgCCCGa	146997	147016	20	3
BART9-3P	BART9	TAACACTTCATGGGgCCCGT	146997	147016	20	21
BART9-3P	BART9	TAACACTTCATGGGTaCCac	146997	147016	20	3
BART9-3P	BART9	TAACACTTCATGGGTaCCGT	146997	147016	20	6
BART9-3P	BART9	TAACACTTCATGGGTCCCaa	146997	147016	20	26
BART9-3P	BART9	TAACACTTCATGGGTCCCag	146997	147016	20	7
BART9-3P	BART9	TAACACTTCATGGGTCCCaT	146997	147016	20	11
BART9-3P	BART9	TAACACTTCATGGGTCCCca	146997	147016	20	13
BART9-3P	BART9	TAACACTTCATGGGTCCCcc	146997	147016	20	5
BART9-3P	BART9	TAACACTTCATGGGTCCCGa	146997	147016	20	32
BART9-3P	BART9	TAACACTTCATGGGTCCCGc	146997	147016	20	33
BART9-3P	BART9	TAACACTTCATGGGTCCCGg	146997	147016	20	10
BART9-3P	BART9	TAACACTTCATGGGTCCCGT	146997	147016	20	147
BART9-3P	BART9	TAACACTTCATGGGTCCCta	146997	147016	20	5
BART9-3P	BART9	TAACACTTCATGGGTCCCtT	146997	147016	20	6
BART9-3P	BART9	TAACACTTCATGGGTCCtGT	146997	147016	20	10
BART9-3P	BART9	TAACACTTCATGGGTaCCGcA	146997	147017	21	6
BART9-3P	BART9	TAACACTTCATGGGTaCCGTA	146997	147017	21	15
BART9-3P	BART9	TAACACTTCATGGGTCaaaTg	146997	147017	21	4
BART9-3P	BART9	TAACACTTCATGGGTCaCGaA	146997	147017	21	3
BART9-3P	BART9	TAACACTTCATGGGTCaCGcA	146997	147017	21	3
BART9-3P	BART9	TAACACTTCATGGGTCaCGTg	146997	147017	21	3
BART9-3P	BART9	TAACACTTCATGGGTCCaGTA	146997	147017	21	5
BART9-3P	BART9	TAACACTTCATGGGTCCCaaA	146997	147017	21	7
BART9-3P	BART9	TAACACTTCATGGGTCCCagA	146997	147017	21	11
BART9-3P	BART9	TAACACTTCATGGGTCCCaTA	146997	147017	21	41
BART9-3P	BART9	TAACACTTCATGGGTCCCcgA	146997	147017	21	9
BART9-3P	BART9	TAACACTTCATGGGTCCCcTA	146997	147017	21	6
BART9-3P	BART9	TAACACTTCATGGGTCCCGaA	146997	147017	21	61
BART9-3P	BART9	TAACACTTCATGGGTCCCGag	146997	147017	21	4
BART9-3P	BART9	TAACACTTCATGGGTCCCGcA	146997	147017	21	252
BART9-3P	BART9	TAACACTTCATGGGTCCCGcg	146997	147017	21	7
BART9-3P	BART9	TAACACTTCATGGGTCCCGgA	146997	147017	21	56
BART9-3P	BART9	TAACACTTCATGGGTCCCGgc	146997	147017	21	5
BART9-3P	BART9	TAACACTTCATGGGTCCCGTA	146997	147017	21	235
BART9-3P	BART9	TAACACTTCATGGGTCCCGTc	146997	147017	21	11
BART9-3P	BART9	TAACACTTCATGGGTCCCGTg	146997	147017	21	94
BART9-3P	BART9	TAACACTTCATGGGTCCCGTt	146997	147017	21	7
BART9-3P	BART9	TAACACTTCATGGGTCCCtcA	146997	147017	21	36
BART9-3P	BART9	TAACACTTCATGGGTCCCtgA	146997	147017	21	6
BART9-3P	BART9	TAACACTTCATGGGTCCCtTA	146997	147017	21	13
BART9-3P	BART9	TAACACTTCATGGGTCtCGTA	146997	147017	21	19
BART9-3P	BART9	aAACACTTCATGGGTCCCGTAa	146997	147018	22	13
BART9-3P	BART9	cAACACTTCATGGGTCCCGTAa	146997	147018	22	6
BART9-3P	BART9	cAACACTTCATGGGTCCCGTgt	146997	147018	22	3
BART9-3P	BART9	TAACACTTCATGGGTCaaGTAt	146997	147018	22	3
BART9-3P	BART9	TAACACTTCATGGGTCaCGTAa	146997	147018	22	27
BART9-3P	BART9	TAACACTTCATGGGTCaCGTAG	146997	147018	22	106
BART9-3P	BART9	TAACACTTCATGGGTCaCGTAt	146997	147018	22	10
BART9-3P	BART9	TAACACTTCATGGGTCCaGTAa	146997	147018	22	3
BART9-3P	BART9	TAACACTTCATGGGTCCaGTAG	146997	147018	22	22
BART9-3P	BART9	TAACACTTCATGGGTCCaGTgt	146997	147018	22	3
BART9-3P	BART9	TAACACTTCATGGGTCCCacAt	146997	147018	22	10
BART9-3P	BART9	TAACACTTCATGGGTCCCacgG	146997	147018	22	11

BART9-3P	BART9	TAACACTTCATGGGTCCCaTAa	146997	147018	22	53
BART9-3P	BART9	TAACACTTCATGGGTCCCaTAG	146997	147018	22	22
BART9-3P	BART9	TAACACTTCATGGGTCCCaTAt	146997	147018	22	30
BART9-3P	BART9	TAACACTTCATGGGTCCCaTgt	146997	147018	22	7
BART9-3P	BART9	TAACACTTCATGGGTCCCaTtc	146997	147018	22	3
BART9-3P	BART9	TAACACTTCATGGGTCCCcaAt	146997	147018	22	5
BART9-3P	BART9	TAACACTTCATGGGTCCCccgG	146997	147018	22	8
BART9-3P	BART9	TAACACTTCATGGGTCCCcTAa	146997	147018	22	4
BART9-3P	BART9	TAACACTTCATGGGTCCCcTAG	146997	147018	22	19
BART9-3P	BART9	TAACACTTCATGGGTCCCcTga	146997	147018	22	8
BART9-3P	BART9	TAACACTTCATGGGTCCCcTgt	146997	147018	22	5
BART9-3P	BART9	TAACACTTCATGGGTCCCGaAa	146997	147018	22	12
BART9-3P	BART9	TAACACTTCATGGGTCCCGaAG	146997	147018	22	12
BART9-3P	BART9	TAACACTTCATGGGTCCCGacc	146997	147018	22	3
BART9-3P	BART9	TAACACTTCATGGGTCCCGacG	146997	147018	22	6
BART9-3P	BART9	TAACACTTCATGGGTCCCGatG	146997	147018	22	26
BART9-3P	BART9	TAACACTTCATGGGTCCCGcAa	146997	147018	22	16
BART9-3P	BART9	TAACACTTCATGGGTCCCGcAG	146997	147018	22	13
BART9-3P	BART9	TAACACTTCATGGGTCCCGcga	146997	147018	22	3
BART9-3P	BART9	TAACACTTCATGGGTCCCGcgG	146997	147018	22	19
BART9-3P	BART9	TAACACTTCATGGGTCCCGgAa	146997	147018	22	5
BART9-3P	BART9	TAACACTTCATGGGTCCCGgAc	146997	147018	22	3
BART9-3P	BART9	TAACACTTCATGGGTCCCGgAG	146997	147018	22	17
BART9-3P	BART9	TAACACTTCATGGGTCCCGgcG	146997	147018	22	7
BART9-3P	BART9	TAACACTTCATGGGTCCCGTAa	146997	147018	22	608
BART9-3P	BART9	TAACACTTCATGGGTCCCGTAc	146997	147018	22	82
BART9-3P	BART9	TAACACTTCATGGGTCCCGTAG	146997	147018	22	1066
BART9-3P	BART9	TAACACTTCATGGGTCCCGTAt	146997	147018	22	262
BART9-3P	BART9	TAACACTTCATGGGTCCCGTca	146997	147018	22	5
BART9-3P	BART9	TAACACTTCATGGGTCCCGTcG	146997	147018	22	13
BART9-3P	BART9	TAACACTTCATGGGTCCCGTga	146997	147018	22	31
BART9-3P	BART9	TAACACTTCATGGGTCCCGTgc	146997	147018	22	4
BART9-3P	BART9	TAACACTTCATGGGTCCCGTgG	146997	147018	22	4
BART9-3P	BART9	TAACACTTCATGGGTCCCGTgt	146997	147018	22	64
BART9-3P	BART9	TAACACTTCATGGGTCCCGTta	146997	147018	22	6
BART9-3P	BART9	TAACACTTCATGGGTCCCGTtG	146997	147018	22	5
BART9-3P	BART9	TAACACTTCATGGGTCCCtcAa	146997	147018	22	9
BART9-3P	BART9	TAACACTTCATGGGTCCCtcAt	146997	147018	22	4
BART9-3P	BART9	TAACACTTCATGGGTCCCtgtG	146997	147018	22	3
BART9-3P	BART9	TAACACTTCATGGGTCCCtTAa	146997	147018	22	6
BART9-3P	BART9	TAACACTTCATGGGTCCCtTAG	146997	147018	22	10
BART9-3P	BART9	TAACACTTCATGGGTCCCtTgt	146997	147018	22	12
BART9-3P	BART9	TAACACTTCATGGGTCCgGTAa	146997	147018	22	16
BART9-3P	BART9	TAACACTTCATGGGTCCgGTAG	146997	147018	22	65
BART9-3P	BART9	TAACACTTCATGGGTCCgGTAt	146997	147018	22	3
BART9-3P	BART9	TAACACTTCATGGGTCCtGTAa	146997	147018	22	4
BART9-3P	BART9	TAACACTTCATGGGTCCtGTAc	146997	147018	22	4
BART9-3P	BART9	TAACACTTCATGGGTCCtGTAG	146997	147018	22	12
BART9-3P	BART9	TAACACTTCATGGGTCCtGTAt	146997	147018	22	3
BART9-3P	BART9	TAACACTTCATGGGTCgCGTAa	146997	147018	22	4
BART9-3P	BART9	TAACACTTCATGGGTCgCGTAG	146997	147018	22	18
BART9-3P	BART9	TAACACTTCATGGGTCgCGTAt	146997	147018	22	13
BART9-3P	BART9	TAACACTTCATGGGTCtCGTAa	146997	147018	22	6
BART9-3P	BART9	TAACACTTCATGGGTCtCGTAG	146997	147018	22	100
BART9-3P	BART9	TAACACTTCATGGGTCtCGTAt	146997	147018	22	5
BART9-3P	BART9	TAACACTTCATGGGTtCCGTAG	146997	147018	22	4
BART9-3P	BART9	aAACACTTCATGGGTCCCGTAGT	146997	147019	23	15
BART9-3P	BART9	acACACTTCATGGGTCCCGTAGT	146997	147019	23	4
BART9-3P	BART9	agACACTTCATGGGTCCCGTAGT	146997	147019	23	4
BART9-3P	BART9	cAACACTTCATGGGTCCCGTAGT	146997	147019	23	23
BART9-3P	BART9	gAACACTTCATGGGTCCCGTAGT	146997	147019	23	35
BART9-3P	BART9	TAACACcTCATGGGTCCCGTAGT	146997	147019	23	5
BART9-3P	BART9	TAACACTTCATGGaTCCCGTAGT	146997	147019	23	18
BART9-3P	BART9	TAACACTTCATGGGcCCCGTAGT	146997	147019	23	10
BART9-3P	BART9	TAACACTTCATGGGTCCaaTAGT	146997	147019	23	10
BART9-3P	BART9	TAACACTTCATGGGTCCaGaAGa	146997	147019	23	3
BART9-3P	BART9	TAACACTTCATGGGTCCaGcAGT	146997	147019	23	18
BART9-3P	BART9	TAACACTTCATGGGTCCaGTAaT	146997	147019	23	12

BART9-3P	BART9	TAACACTTCATGGGTCCaGTAGa	146997	147019	23	23
BART9-3P	BART9	TAACACTTCATGGGTCCaGTAGc	146997	147019	23	16
BART9-3P	BART9	TAACACTTCATGGGTCCaGTAGT	146997	147019	23	328
BART9-3P	BART9	TAACACTTCATGGGTCCaGTgGT	146997	147019	23	3
BART9-3P	BART9	TAACACTTCATGGGTCCatTAGT	146997	147019	23	4
BART9-3P	BART9	TAACACTTCATGGGTCCCaacGT	146997	147019	23	5
BART9-3P	BART9	TAACACTTCATGGGTCCCacAaT	146997	147019	23	5
BART9-3P	BART9	TAACACTTCATGGGTCCCacAGa	146997	147019	23	6
BART9-3P	BART9	TAACACTTCATGGGTCCCacAGT	146997	147019	23	13
BART9-3P	BART9	TAACACTTCATGGGTCCCaccGT	146997	147019	23	29
BART9-3P	BART9	TAACACTTCATGGGTCCCacgGc	146997	147019	23	5
BART9-3P	BART9	TAACACTTCATGGGTCCCacgGg	146997	147019	23	4
BART9-3P	BART9	TAACACTTCATGGGTCCCactGT	146997	147019	23	22
BART9-3P	BART9	TAACACTTCATGGGTCCCagAGa	146997	147019	23	6
BART9-3P	BART9	TAACACTTCATGGGTCCCagtGT	146997	147019	23	8
BART9-3P	BART9	TAACACTTCATGGGTCCCaTAaT	146997	147019	23	26
BART9-3P	BART9	TAACACTTCATGGGTCCCaTAGa	146997	147019	23	37
BART9-3P	BART9	TAACACTTCATGGGTCCCaTAGc	146997	147019	23	8
BART9-3P	BART9	TAACACTTCATGGGTCCCaTAGT	146997	147019	23	54
BART9-3P	BART9	TAACACTTCATGGGTCCCaTgcT	146997	147019	23	3
BART9-3P	BART9	TAACACTTCATGGGTCCCaTgGT	146997	147019	23	20
BART9-3P	BART9	TAACACTTCATGGGTCCCaTtGT	146997	147019	23	8
BART9-3P	BART9	TAACACTTCATGGGTCCCaTttT	146997	147019	23	6
BART9-3P	BART9	TAACACTTCATGGGTCCCcaAGa	146997	147019	23	7
BART9-3P	BART9	TAACACTTCATGGGTCCCcaAtc	146997	147019	23	6
BART9-3P	BART9	TAACACTTCATGGGTCCCcaAtT	146997	147019	23	3
BART9-3P	BART9	TAACACTTCATGGGTCCCcTAca	146997	147019	23	5
BART9-3P	BART9	TAACACTTCATGGGTCCCcTAGa	146997	147019	23	3
BART9-3P	BART9	TAACACTTCATGGGTCCCcTAGT	146997	147019	23	18
BART9-3P	BART9	TAACACTTCATGGGTCCCcTAtc	146997	147019	23	9
BART9-3P	BART9	TAACACTTCATGGGTCCCcTgaT	146997	147019	23	5
BART9-3P	BART9	TAACACTTCATGGGTCCCcTgGc	146997	147019	23	4
BART9-3P	BART9	TAACACTTCATGGGTCCCcTgGT	146997	147019	23	3
BART9-3P	BART9	TAACACTTCATGGGTCCCcTtGT	146997	147019	23	3
BART9-3P	BART9	TAACACTTCATGGGTCCCGaAGa	146997	147019	23	13
BART9-3P	BART9	TAACACTTCATGGGTCCCGaAGc	146997	147019	23	3
BART9-3P	BART9	TAACACTTCATGGGTCCCGaAGT	146997	147019	23	6
BART9-3P	BART9	TAACACTTCATGGGTCCCGacGa	146997	147019	23	3
BART9-3P	BART9	TAACACTTCATGGGTCCCGacGT	146997	147019	23	42
BART9-3P	BART9	TAACACTTCATGGGTCCCGagtT	146997	147019	23	5
BART9-3P	BART9	TAACACTTCATGGGTCCCGatGa	146997	147019	23	8
BART9-3P	BART9	TAACACTTCATGGGTCCCGatGT	146997	147019	23	30
BART9-3P	BART9	TAACACTTCATGGGTCCCGcAaa	146997	147019	23	5
BART9-3P	BART9	TAACACTTCATGGGTCCCGcAaT	146997	147019	23	15
BART9-3P	BART9	TAACACTTCATGGGTCCCGcAGa	146997	147019	23	40
BART9-3P	BART9	TAACACTTCATGGGTCCCGcAGc	146997	147019	23	11
BART9-3P	BART9	TAACACTTCATGGGTCCCGcAGg	146997	147019	23	3
BART9-3P	BART9	TAACACTTCATGGGTCCCGcAGT	146997	147019	23	257
BART9-3P	BART9	TAACACTTCATGGGTCCCGccGT	146997	147019	23	39
BART9-3P	BART9	TAACACTTCATGGGTCCCGcgaT	146997	147019	23	10
BART9-3P	BART9	TAACACTTCATGGGTCCCGcgGc	146997	147019	23	5
BART9-3P	BART9	TAACACTTCATGGGTCCCGctGT	146997	147019	23	5
BART9-3P	BART9	TAACACTTCATGGGTCCCGgAGa	146997	147019	23	26
BART9-3P	BART9	TAACACTTCATGGGTCCCGgAGc	146997	147019	23	10
BART9-3P	BART9	TAACACTTCATGGGTCCCGgAGg	146997	147019	23	4
BART9-3P	BART9	TAACACTTCATGGGTCCCGgAGT	146997	147019	23	21
BART9-3P	BART9	TAACACTTCATGGGTCCCGgcGT	146997	147019	23	4
BART9-3P	BART9	TAACACTTCATGGGTCCCGgctT	146997	147019	23	6
BART9-3P	BART9	TAACACTTCATGGGTCCCGggGT	146997	147019	23	7
BART9-3P	BART9	TAACACTTCATGGGTCCCGgtaT	146997	147019	23	5
BART9-3P	BART9	TAACACTTCATGGGTCCCGTAaa	146997	147019	23	6
BART9-3P	BART9	TAACACTTCATGGGTCCCGTAac	146997	147019	23	5
BART9-3P	BART9	TAACACTTCATGGGTCCCGTAaT	146997	147019	23	271
BART9-3P	BART9	TAACACTTCATGGGTCCCGTAca	146997	147019	23	4
BART9-3P	BART9	TAACACTTCATGGGTCCCGTAcT	146997	147019	23	4
BART9-3P	BART9	TAACACTTCATGGGTCCCGTAGa	146997	147019	23	476
BART9-3P	BART9	TAACACTTCATGGGTCCCGTAGc	146997	147019	23	503
BART9-3P	BART9	TAACACTTCATGGGTCCCGTAGg	146997	147019	23	120

BART9-3P	BART9	TAACACTTCATGGGTCCCGTAGT	146997	147019	23	5266
BART9-3P	BART9	TAACACTTCATGGGTCCCGTAtc	146997	147019	23	7
BART9-3P	BART9	TAACACTTCATGGGTCCCGTAtT	146997	147019	23	43
BART9-3P	BART9	TAACACTTCATGGGTCCCGTcaT	146997	147019	23	12
BART9-3P	BART9	TAACACTTCATGGGTCCCGTcGg	146997	147019	23	9
BART9-3P	BART9	TAACACTTCATGGGTCCCGTcGT	146997	147019	23	22
BART9-3P	BART9	TAACACTTCATGGGTCCCGTctg	146997	147019	23	5
BART9-3P	BART9	TAACACTTCATGGGTCCCGTctT	146997	147019	23	5
BART9-3P	BART9	TAACACTTCATGGGTCCCGTgac	146997	147019	23	4
BART9-3P	BART9	TAACACTTCATGGGTCCCGTgaT	146997	147019	23	6
BART9-3P	BART9	TAACACTTCATGGGTCCCGTgGc	146997	147019	23	3
BART9-3P	BART9	TAACACTTCATGGGTCCCGTgGT	146997	147019	23	113
BART9-3P	BART9	TAACACTTCATGGGTCCCGTgtc	146997	147019	23	3
BART9-3P	BART9	TAACACTTCATGGGTCCCGTgtT	146997	147019	23	5
BART9-3P	BART9	TAACACTTCATGGGTCCCGTtaT	146997	147019	23	13
BART9-3P	BART9	TAACACTTCATGGGTCCCGTtca	146997	147019	23	8
BART9-3P	BART9	TAACACTTCATGGGTCCCGTtcT	146997	147019	23	3
BART9-3P	BART9	TAACACTTCATGGGTCCCGTtGT	146997	147019	23	54
BART9-3P	BART9	TAACACTTCATGGGTCCCtaAGT	146997	147019	23	6
BART9-3P	BART9	TAACACTTCATGGGTCCCtacGT	146997	147019	23	6
BART9-3P	BART9	TAACACTTCATGGGTCCCtcAGT	146997	147019	23	3
BART9-3P	BART9	TAACACTTCATGGGTCCCtctGT	146997	147019	23	6
BART9-3P	BART9	TAACACTTCATGGGTCCCtgAGT	146997	147019	23	30
BART9-3P	BART9	TAACACTTCATGGGTCCCtgtGT	146997	147019	23	6
BART9-3P	BART9	TAACACTTCATGGGTCCCtTAGT	146997	147019	23	62
BART9-3P	BART9	TAACACTTCATGGGTCCCtTtca	146997	147019	23	4
BART9-3P	BART9	TAACACTTCATGGGTCCCtTtGT	146997	147019	23	9
BART9-3P	BART9	TAACACTTCATGGGTCCgGcAGT	146997	147019	23	5
BART9-3P	BART9	TAACACTTCATGGGTCCgGTAGa	146997	147019	23	16
BART9-3P	BART9	TAACACTTCATGGGTCCgGTAGT	146997	147019	23	156
BART9-3P	BART9	TAACACTTCATGGGTCCtaTAGT	146997	147019	23	10
BART9-3P	BART9	TAACACTTCATGGGTCCtGcAGT	146997	147019	23	5
BART9-3P	BART9	TAACACTTCATGGGTCCtGTAGa	146997	147019	23	5
BART9-3P	BART9	TAACACTTCATGGGTCCtGTAGc	146997	147019	23	6
BART9-3P	BART9	TAACACTTCATGGGTCCtGTAGT	146997	147019	23	209
BART9-3P	BART9	TAACACTTCATGGGTCCtGTgGT	146997	147019	23	3
BART9-3P	BART9	TAACACTTCATGGGTCCttTAGT	146997	147019	23	4
BART9-3P	BART9	TAACACTTCATGGGTCtCGTAGT	146997	147019	23	4
BART9-3P	BART9	TAACACTTCgTGGGTCCCGTAGT	146997	147019	23	9
BART9-3P	BART9	TAACACTTgtTGGGTCCCGTAGT	146997	147019	23	3
BART9-3P	BART9	TAACACTTtATGGGTCCCGTAGT	146997	147019	23	8
BART9-3P	BART9	TAACAtTTCATGGGTCCCGTAGT	146997	147019	23	4
BART9-3P	BART9	TAAtACTTCATGGGTCCCGTAGT	146997	147019	23	4
BART9-3P	BART9	TAgCACTTCATGGGTCCCGTAGT	146997	147019	23	17
BART9-3P	BART9	TcACACTTCATGGGTCCCGcAGT	146997	147019	23	5
BART9-3P	BART9	TgACACTTCATGGGTCCCGatGT	146997	147019	23	12
BART9-3P	BART9	TgACACTTCATGGGTCCCGTAGT	146997	147019	23	4
BART9-3P	BART9	gAACACTTCATGGGTCCCGTAGTa	146997	147020	24	4
BART9-3P	BART9	TAACACTTCATGGGTCCCacAGTG	146997	147020	24	8
BART9-3P	BART9	TAACACTTCATGGGTCCCagAGTG	146997	147020	24	16
BART9-3P	BART9	TAACACTTCATGGGTCCCaTAcTc	146997	147020	24	4
BART9-3P	BART9	TAACACTTCATGGGTCCCaTAGca	146997	147020	24	8
BART9-3P	BART9	TAACACTTCATGGGTCCCaTAGTa	146997	147020	24	6
BART9-3P	BART9	TAACACTTCATGGGTCCCaTAGTG	146997	147020	24	76
BART9-3P	BART9	TAACACTTCATGGGTCCCaTAGTt	146997	147020	24	6
BART9-3P	BART9	TAACACTTCATGGGTCCCcTAGTG	146997	147020	24	9
BART9-3P	BART9	TAACACTTCATGGGTCCCGaAGTa	146997	147020	24	3
BART9-3P	BART9	TAACACTTCATGGGTCCCGaAGTG	146997	147020	24	8
BART9-3P	BART9	TAACACTTCATGGGTCCCGacGTG	146997	147020	24	12
BART9-3P	BART9	TAACACTTCATGGGTCCCGagGTG	146997	147020	24	3
BART9-3P	BART9	TAACACTTCATGGGTCCCGatGTG	146997	147020	24	5
BART9-3P	BART9	TAACACTTCATGGGTCCCGcAGca	146997	147020	24	4
BART9-3P	BART9	TAACACTTCATGGGTCCCGcAGTG	146997	147020	24	33
BART9-3P	BART9	TAACACTTCATGGGTCCCGgAGTG	146997	147020	24	14
BART9-3P	BART9	TAACACTTCATGGGTCCCGTAaTG	146997	147020	24	24
BART9-3P	BART9	TAACACTTCATGGGTCCCGTAcac	146997	147020	24	8
BART9-3P	BART9	TAACACTTCATGGGTCCCGTAcaG	146997	147020	24	78
BART9-3P	BART9	TAACACTTCATGGGTCCCGTAcTG	146997	147020	24	12

BART9-3P	BART9	TAACACTTCATGGGTCCCGTAGaG	146997	147020	24	7
BART9-3P	BART9	TAACACTTCATGGGTCCCGTAGat	146997	147020	24	3
BART9-3P	BART9	TAACACTTCATGGGTCCCGTAGca	146997	147020	24	23
BART9-3P	BART9	TAACACTTCATGGGTCCCGTAGcc	146997	147020	24	3
BART9-3P	BART9	TAACACTTCATGGGTCCCGTAGcG	146997	147020	24	8
BART9-3P	BART9	TAACACTTCATGGGTCCCGTAGct	146997	147020	24	3
BART9-3P	BART9	TAACACTTCATGGGTCCCGTAGgc	146997	147020	24	16
BART9-3P	BART9	TAACACTTCATGGGTCCCGTAGgG	146997	147020	24	10
BART9-3P	BART9	TAACACTTCATGGGTCCCGTAGgt	146997	147020	24	15
BART9-3P	BART9	TAACACTTCATGGGTCCCGTAGTa	146997	147020	24	89
BART9-3P	BART9	TAACACTTCATGGGTCCCGTAGTc	146997	147020	24	42
BART9-3P	BART9	TAACACTTCATGGGTCCCGTAGTG	146997	147020	24	825
BART9-3P	BART9	TAACACTTCATGGGTCCCGTAGTt	146997	147020	24	20
BART9-3P	BART9	TAACACTTCATGGGTCCCGTAtaG	146997	147020	24	3
BART9-3P	BART9	TAACACTTCATGGGTCCCGTAtTa	146997	147020	24	11
BART9-3P	BART9	TAACACTTCATGGGTCCCGTAtTG	146997	147020	24	87
BART9-3P	BART9	TAACACTTCATGGGTCCCGTcacG	146997	147020	24	4
BART9-3P	BART9	TAACACTTCATGGGTCCCGTcGTG	146997	147020	24	3
BART9-3P	BART9	TAACACTTCATGGGTCCCGTgacG	146997	147020	24	6
BART9-3P	BART9	TAACACTTCATGGGTCCCGTgaTG	146997	147020	24	9
BART9-3P	BART9	TAACACTTCATGGGTCCCGTgcTG	146997	147020	24	23
BART9-3P	BART9	TAACACTTCATGGGTCCCGTgGTG	146997	147020	24	11
BART9-3P	BART9	TAACACTTCATGGGTCCCGTtGTG	146997	147020	24	14
BART9-3P	BART9	TAACACTTCATGGGTCCCtTAGTG	146997	147020	24	3
BART9-3P	BART9	aAACACTTCATGGGTCCCGTAGTcc	146997	147021	25	4
BART9-3P	BART9	TAACACTTCATGGGTCCCGaAGTat	146997	147021	25	6
BART9-3P	BART9	TAACACTTCATGGGTCCCGcAGTaA	146997	147021	25	4
BART9-3P	BART9	TAACACTTCATGGGTCCCGcAGTat	146997	147021	25	3
BART9-3P	BART9	TAACACTTCATGGGTCCCGgAGTaA	146997	147021	25	3
BART9-3P	BART9	TAACACTTCATGGGTCCCGgAGTat	146997	147021	25	8
BART9-3P	BART9	TAACACTTCATGGGTCCCGTAaTat	146997	147021	25	5
BART9-3P	BART9	TAACACTTCATGGGTCCCGTAaTct	146997	147021	25	3
BART9-3P	BART9	TAACACTTCATGGGTCCCGTAcTat	146997	147021	25	4
BART9-3P	BART9	TAACACTTCATGGGTCCCGTAGaat	146997	147021	25	3
BART9-3P	BART9	TAACACTTCATGGGTCCCGTAGact	146997	147021	25	3
BART9-3P	BART9	TAACACTTCATGGGTCCCGTAGaGA	146997	147021	25	6
BART9-3P	BART9	TAACACTTCATGGGTCCCGTAGatA	146997	147021	25	4
BART9-3P	BART9	TAACACTTCATGGGTCCCGTAGatt	146997	147021	25	7
BART9-3P	BART9	TAACACTTCATGGGTCCCGTAGctA	146997	147021	25	3
BART9-3P	BART9	TAACACTTCATGGGTCCCGTAGTaA	146997	147021	25	36
BART9-3P	BART9	TAACACTTCATGGGTCCCGTAGTac	146997	147021	25	11
BART9-3P	BART9	TAACACTTCATGGGTCCCGTAGTat	146997	147021	25	77
BART9-3P	BART9	TAACACTTCATGGGTCCCGTAGTcA	146997	147021	25	3
BART9-3P	BART9	TAACACTTCATGGGTCCCGTAGTct	146997	147021	25	4
BART9-3P	BART9	TAACACTTCATGGGTCCCGTAGTGA	146997	147021	25	4
BART9-3P	BART9	TAACACTTCATGGGTCCCGTAGTGc	146997	147021	25	8
BART9-3P	BART9	TAACACTTCATGGGTCCCGTAGTGg	146997	147021	25	3
BART9-3P	BART9	TAACACTTCATGGGTCCCGTAGTGt	146997	147021	25	12
BART9-3P	BART9	TAACACTTCATGGGTCCCGTAGTtA	146997	147021	25	4
BART9-3P	BART9	TAACACTTCATGGGTCCCGTAGTtt	146997	147021	25	8
BART9-3P	BART9	TAACACTTCATGGGTCCCGTgGTaA	146997	147021	25	3
BART9-3P	BART9	TAACACTTCATGGGTCCCGTAGTGAC	146997	147022	26	10
BART9-3P	BART9	TAACACTTCATGGGTCCCGTAGTGAg	146997	147022	26	3
BART9-3P	BART9	TAACACTTCATGGGTCCCGTAGTGAt	146997	147022	26	5
BART9-3P	BART9	TAACACTTCATGGGTCCCGTAGTGtC	146997	147022	26	19
BART9-3P	BART9	TAACACTTCATGGGTCCCGTgacGACA	146997	147023	27	5
BART9-3P	BART9	TAACACTTCATGGGTCCCGTgacGACg	146997	147023	27	7
BART9-3P	BART9	TAACACTTCATGGGTCCCGTAGTGACAA	146997	147024	28	3
BART9-3P	BART9	AACACTTCATGGGTCCCGcA	146998	147017	20	9
BART9-3P	BART9	AACACTTCATGGGTCCCGTA	146998	147017	20	56
BART9-3P	BART9	AACACTTCATGGGTCaCGTAt	146998	147018	21	4
BART9-3P	BART9	AACACTTCATGGGTCCCacgG	146998	147018	21	14
BART9-3P	BART9	AACACTTCATGGGTCCCGTAa	146998	147018	21	15
BART9-3P	BART9	AACACTTCATGGGTCCCGTAG	146998	147018	21	67
BART9-3P	BART9	AACACTTCATGGGTCCCGTAt	146998	147018	21	8
BART9-3P	BART9	AACACTTCATGGGTCCaGTAGT	146998	147019	22	45
BART9-3P	BART9	AACACTTCATGGGTCCCaTtGT	146998	147019	22	9
BART9-3P	BART9	AACACTTCATGGGTCCCGcAGT	146998	147019	22	4

BART9-3P	BART9	AACACTTCATGGGTCCCGTAac	146998	147019	22	3
BART9-3P	BART9	AACACTTCATGGGTCCCGTAGa	146998	147019	22	13
BART9-3P	BART9	AACACTTCATGGGTCCCGTAGc	146998	147019	22	7
BART9-3P	BART9	AACACTTCATGGGTCCCGTAGg	146998	147019	22	8
BART9-3P	BART9	AACACTTCATGGGTCCCGTAGT	146998	147019	22	177
BART9-3P	BART9	AACACTTCATGGGTCCtGTAGT	146998	147019	22	12
BART9-3P	BART9	AACACTTCATGGGTCCCGTAGca	146998	147020	23	5
BART9-3P	BART9	AACACTTCATGGGTCCCGTAGgc	146998	147020	23	4
BART9-3P	BART9	AACACTTCATGGGTCCCGTAGTG	146998	147020	23	28
BART22-5P		TGCTAGACCCTGGAGTTGAAC	147169	147189	21	5
BART22-5P		TGCTAGACCCTGGAGTTGAACC	147169	147190	22	5
BART22-3P	BART22	TTACAAAGTCATGGTCTAGTA	147203	147223	21	10
BART22-3P	BART22	TTACAAAGTCATGGTCTAGTg	147203	147223	21	7
BART22-3P	BART22	TTACAAAGTCATGGTtTAGTA	147203	147223	21	3
BART22-3P	BART22	TTACAAAGTCATGGTCaAGTAG	147203	147224	22	4
BART22-3P	BART22	TTACAAAGTCATGGTCcAGTAG	147203	147224	22	3
BART22-3P	BART22	TTACAAAGTCATGGTCgAGTAG	147203	147224	22	20
BART22-3P	BART22	TTACAAAGTCATGGTCTAGcga	147203	147224	22	3
BART22-3P	BART22	TTACAAAGTCATGGTCTAGTAc	147203	147224	22	9
BART22-3P	BART22	TTACAAAGTCATGGTCTAGTAG	147203	147224	22	83
BART22-3P	BART22	TTACAAAGTCATGGTCTAGTga	147203	147224	22	4
BART22-3P	BART22	TTACAAAGTCATGGTCTgGTAG	147203	147224	22	4
BART22-3P	BART22	TTACAAAGTCATGGTCTtGTAG	147203	147224	22	6
BART22-3P	BART22	TTACAAAGTCATGGTCTAaTAGT	147203	147225	23	5
BART22-3P	BART22	TTACAAAGTCATGGTCTAaTgGT	147203	147225	23	4
BART22-3P	BART22	TTACAAAGTCATGGTCTAGacGT	147203	147225	23	3
BART22-3P	BART22	TTACAAAGTCATGGTCTAGcAGT	147203	147225	23	9
BART22-3P	BART22	TTACAAAGTCATGGTCTAGccGT	147203	147225	23	8
BART22-3P	BART22	TTACAAAGTCATGGTCTAGcgGT	147203	147225	23	7
BART22-3P	BART22	TTACAAAGTCATGGTCTAGTAaT	147203	147225	23	4
BART22-3P	BART22	TTACAAAGTCATGGTCTAGTAca	147203	147225	23	4
BART22-3P	BART22	TTACAAAGTCATGGTCTAGTAGa	147203	147225	23	5
BART22-3P	BART22	TTACAAAGTCATGGTCTAGTAGc	147203	147225	23	7
BART22-3P	BART22	TTACAAAGTCATGGTCTAGTAGg	147203	147225	23	10
BART22-3P	BART22	TTACAAAGTCATGGTCTAGTAGT	147203	147225	23	167
BART22-3P	BART22	TTACAAAGTCATGGTCTAGTAtT	147203	147225	23	3
BART22-3P	BART22	TTACAAAGTCATGGTCTAGTgGT	147203	147225	23	4
BART22-3P	BART22	TTACAAAGTCATGGTCTAtTAGT	147203	147225	23	3
BART22-3P	BART22	TTACAAAGTCATGGTCTcGTAGT	147203	147225	23	3
BART22-3P	BART22	TTACAAAGTCATGGTCTgGTAGT	147203	147225	23	10
BART22-3P	BART22	TTACAAAGTCATGGTCTtGTAGT	147203	147225	23	4
BART22-3P	BART22	TTACAAAGTCATGGTCTAaTAGgT	147203	147226	24	4
BART22-3P	BART22	TTACAAAGTCATGGTCTAGTAaTT	147203	147226	24	3
BART22-3P	BART22	TTACAAAGTCATGGTCTAGTAGcT	147203	147226	24	5
BART22-3P	BART22	TTACAAAGTCATGGTCTAGTAGgT	147203	147226	24	5
BART22-3P	BART22	TTACAAAGTCATGGTCTAGTAGTc	147203	147226	24	3
BART22-3P	BART22	TTACAAAGTCATGGTCTAGTAGTT	147203	147226	24	15
BART22-3P	BART22	TTACAAAGTCATGGTCTAGTAtgT	147203	147226	24	4
BART22-3P	BART22	TTACAAAGTCATGGTCTAGTgGTT	147203	147226	24	11
BART22-3P	BART22	TTACAAAGTCATGGTCTAGTtcgT	147203	147226	24	3
BART22-3P	BART22	TACAAAGTCATGGTCgAGTAG	147204	147224	21	3
BART22-3P	BART22	TACAAAGTCATGGTCTAGTAG	147204	147224	21	6
BART22-3P	BART22	TACAAAGTCATGGTCTAGTAGa	147204	147225	22	4
BART22-3P	BART22	TACAAAGTCATGGTCTAGTAGT	147204	147225	22	40
BART22-3P	BART22	TACAAAGTCATGGTCTAaTAGTT	147204	147226	23	3
BART22-3P	BART22	TACAAAGTCATGGTCTAGTAacT	147204	147226	23	3
BART22-3P	BART22	TACAAAGTCATGGTCTAGTAGTT	147204	147226	23	6
BART10-5P	BART10-star	GGGCCACCTCTTTGGTTCTGcA	147319	147340	22	5
BART10-5P	BART10-star	GCCACCTCTTTGGTTCcGTA	147321	147340	20	40
BART10-5P	BART10-star	GCCACCTCTTTGGTTCTGTA	147321	147340	20	6
BART10-5P	BART10-star	GCCACCTCTTTGGTTaTGTAC	147321	147341	21	3
BART10-5P	BART10-star	GCCACCTCTTTGGTTCaGTAC	147321	147341	21	17
BART10-5P	BART10-star	GCCACCTCTTTGGTTCTGTAC	147321	147341	21	81
BART10-5P	BART10-star	GCCACCTCTTTGGTTCTtTAC	147321	147341	21	3
BART10-5P	BART10-star	GCCACCTCTTTGGTTgTGcAC	147321	147341	21	3
BART10-3P	BART10	TACATAACCATGGAGaacG	147356	147374	19	5
BART10-3P	BART10	TACATAACCATGGAaTTGGC	147356	147375	20	3
BART10-3P	BART10	TACATAACCATGGAGaTGGC	147356	147375	20	3

BART10-3P	BART10	TACATAACCATGGAGTTGaC	147356	147375	20	13
BART10-3P	BART10	TACATAACCATGGAGTTGGC	147356	147375	20	4
BART10-3P	BART10	TACATAACCATGGAGTcaGCT	147356	147376	21	10
BART10-3P	BART10	TACATAACCATGGAGTcGGCT	147356	147376	21	4
BART10-3P	BART10	TACATAACCATGGAGTTGaCT	147356	147376	21	3
BART10-3P	BART10	TACATAACCATGGAGTTGGCc	147356	147376	21	3
BART10-3P	BART10	TACATAACCATGGAGTTGGCT	147356	147376	21	8
BART10-3P	BART10	TACATAACCATGGAGTaGaCTG	147356	147377	22	3
BART10-3P	BART10	TACATAACCATGGAGTTaaCTG	147356	147377	22	3
BART10-3P	BART10	TACATAACCATGGAGTTaGCTG	147356	147377	22	6
BART10-3P	BART10	TACATAACCATGGAGTTGaCaG	147356	147377	22	7
BART10-3P	BART10	TACATAACCATGGAGTTGaCga	147356	147377	22	4
BART10-3P	BART10	TACATAACCATGGAGTTGaCgt	147356	147377	22	4
BART10-3P	BART10	TACATAACCATGGAGTTGaCTa	147356	147377	22	6
BART10-3P	BART10	TACATAACCATGGAGTTGaCTG	147356	147377	22	12
BART10-3P	BART10	TACATAACCATGGAGTTGagTG	147356	147377	22	3
BART10-3P	BART10	TACATAACCATGGAGTTGGacG	147356	147377	22	3
BART10-3P	BART10	TACATAACCATGGAGTTGGCaG	147356	147377	22	4
BART10-3P	BART10	TACATAACCATGGAGTTGGCTa	147356	147377	22	11
BART10-3P	BART10	TACATAACCATGGAGTTGGCTc	147356	147377	22	5
BART10-3P	BART10	TACATAACCATGGAGTTGGCTG	147356	147377	22	50
BART10-3P	BART10	aACATAACCATGGAGTTGGCcGT	147356	147378	23	5
BART10-3P	BART10	TACATAACCATGGAGTTaaCTaa	147356	147378	23	3
BART10-3P	BART10	TACATAACCATGGAGTTaaCTGa	147356	147378	23	5
BART10-3P	BART10	TACATAACCATGGAGTTaaCTGg	147356	147378	23	4
BART10-3P	BART10	TACATAACCATGGAGTTaaCTGT	147356	147378	23	19
BART10-3P	BART10	TACATAACCATGGAGTTaGCaGa	147356	147378	23	24
BART10-3P	BART10	TACATAACCATGGAGTTaGCaGT	147356	147378	23	7
BART10-3P	BART10	TACATAACCATGGAGTTaGCcGa	147356	147378	23	7
BART10-3P	BART10	TACATAACCATGGAGTTaGCcGT	147356	147378	23	12
BART10-3P	BART10	TACATAACCATGGAGTTaGCTag	147356	147378	23	3
BART10-3P	BART10	TACATAACCATGGAGTTaGCTGa	147356	147378	23	44
BART10-3P	BART10	TACATAACCATGGAGTTaGCTGg	147356	147378	23	3
BART10-3P	BART10	TACATAACCATGGAGTTaGCTGT	147356	147378	23	75
BART10-3P	BART10	TACATAACCATGGAGTTcGCcGc	147356	147378	23	10
BART10-3P	BART10	TACATAACCATGGAGTTcGCTGa	147356	147378	23	5
BART10-3P	BART10	TACATAACCATGGAGTTcGCTGT	147356	147378	23	7
BART10-3P	BART10	TACATAACCATGGAGTTGaaTGT	147356	147378	23	3
BART10-3P	BART10	TACATAACCATGGAGTTGaCaGa	147356	147378	23	7
BART10-3P	BART10	TACATAACCATGGAGTTGaCaGT	147356	147378	23	10
BART10-3P	BART10	TACATAACCATGGAGTTGaCcGa	147356	147378	23	9
BART10-3P	BART10	TACATAACCATGGAGTTGaCcGc	147356	147378	23	3
BART10-3P	BART10	TACATAACCATGGAGTTGaCcGT	147356	147378	23	25
BART10-3P	BART10	TACATAACCATGGAGTTGaCgGT	147356	147378	23	7
BART10-3P	BART10	TACATAACCATGGAGTTGaCTaa	147356	147378	23	11
BART10-3P	BART10	TACATAACCATGGAGTTGaCTaT	147356	147378	23	7
BART10-3P	BART10	TACATAACCATGGAGTTGaCTGa	147356	147378	23	141
BART10-3P	BART10	TACATAACCATGGAGTTGaCTGc	147356	147378	23	10
BART10-3P	BART10	TACATAACCATGGAGTTGaCTGg	147356	147378	23	3
BART10-3P	BART10	TACATAACCATGGAGTTGaCTGT	147356	147378	23	449
BART10-3P	BART10	TACATAACCATGGAGTTGaCTtg	147356	147378	23	3
BART10-3P	BART10	TACATAACCATGGAGTTGatTGa	147356	147378	23	9
BART10-3P	BART10	TACATAACCATGGAGTTGatTGT	147356	147378	23	10
BART10-3P	BART10	TACATAACCATGGAGTTGcCTGa	147356	147378	23	7
BART10-3P	BART10	TACATAACCATGGAGTTGcCTGT	147356	147378	23	11
BART10-3P	BART10	TACATAACCATGGAGTTGGacGa	147356	147378	23	8
BART10-3P	BART10	TACATAACCATGGAGTTGGagGa	147356	147378	23	3
BART10-3P	BART10	TACATAACCATGGAGTTGGaTac	147356	147378	23	6
BART10-3P	BART10	TACATAACCATGGAGTTGGaTGa	147356	147378	23	11
BART10-3P	BART10	TACATAACCATGGAGTTGGCaaT	147356	147378	23	11
BART10-3P	BART10	TACATAACCATGGAGTTGGCaGa	147356	147378	23	50
BART10-3P	BART10	TACATAACCATGGAGTTGGCaGT	147356	147378	23	141
BART10-3P	BART10	TACATAACCATGGAGTTGGCcGa	147356	147378	23	23
BART10-3P	BART10	TACATAACCATGGAGTTGGCcGc	147356	147378	23	3
BART10-3P	BART10	TACATAACCATGGAGTTGGCcGT	147356	147378	23	89
BART10-3P	BART10	TACATAACCATGGAGTTGGCctT	147356	147378	23	5
BART10-3P	BART10	TACATAACCATGGAGTTGGCTaa	147356	147378	23	17
BART10-3P	BART10	TACATAACCATGGAGTTGGCTac	147356	147378	23	19

BART10-3P	BART10	TACATAACCATGGAGTTGGCTaT	147356	147378	23	15
BART10-3P	BART10	TACATAACCATGGAGTTGGCTGa	147356	147378	23	34
BART10-3P	BART10	TACATAACCATGGAGTTGGCTGc	147356	147378	23	18
BART10-3P	BART10	TACATAACCATGGAGTTGGCTGg	147356	147378	23	6
BART10-3P	BART10	TACATAACCATGGAGTTGGCTGT	147356	147378	23	171
BART10-3P	BART10	TACATAACCATGGAGTTGGCTtg	147356	147378	23	9
BART10-3P	BART10	TACATAACCATGGAGTTGGCTtT	147356	147378	23	5
BART10-3P	BART10	TACATAACCATGGAGTTGGtTGa	147356	147378	23	3
BART10-3P	BART10	TACATAACCATGGAGTTGGtTGT	147356	147378	23	3
BART10-3P	BART10	TACATAACCATGGAGTTGtCaGa	147356	147378	23	7
BART10-3P	BART10	TACATAACCATGGAGTTGtCTGa	147356	147378	23	3
BART10-3P	BART10	TACATAACCATGGAGTTGtCTGT	147356	147378	23	10
BART10-3P	BART10	TACATAACCATGGAGTTtGCTGT	147356	147378	23	6
BART10-3P	BART10	TACATAACCgTGGAGTTGGCTGT	147356	147378	23	11
BART10-3P	BART10	TACATgACCATGGAGTTGaCTGT	147356	147378	23	3
BART10-3P	BART10	TACATtACCATGGAGTTGGCTGT	147356	147378	23	4
BART10-3P	BART10	TACATAACCATGGAGTTGaCcGTG	147356	147379	24	9
BART10-3P	BART10	TACATAACCATGGAGTTGaCTGTG	147356	147379	24	11
BART10-3P	BART10	TACATAACCATGGAGTTGaCTGTt	147356	147379	24	8
BART10-3P	BART10	TACATAACCATGGAGTTGatTGTa	147356	147379	24	4
BART10-3P	BART10	TACATAACCATGGAGTTGGCaGTG	147356	147379	24	3
BART10-3P	BART10	TACATAACCATGGAGTTGGCcGTa	147356	147379	24	6
BART10-3P	BART10	TACATAACCATGGAGTTGGCTaca	147356	147379	24	4
BART10-3P	BART10	TACATAACCATGGAGTTGGCTact	147356	147379	24	5
BART10-3P	BART10	TACATAACCATGGAGTTGGCTaTG	147356	147379	24	3
BART10-3P	BART10	TACATAACCATGGAGTTGGCTGaG	147356	147379	24	6
BART10-3P	BART10	TACATAACCATGGAGTTGGCTGTa	147356	147379	24	6
BART10-3P	BART10	TACATAACCATGGAGTTGGCTGTc	147356	147379	24	3
BART10-3P	BART10	TACATAACCATGGAGTTGGCTGTG	147356	147379	24	59
BART10-3P	BART10	TACATAACCATGGAGTTGGCTGTt	147356	147379	24	16
BART10-3P	BART10	TACATAACCATGGAGTTGGCTGaGa	147356	147380	25	4
BART10-3P	BART10	TACATAACCATGGAGTTGGCTGctG	147356	147380	25	5
BART10-3P	BART10	TACATAACCATGGAGTTGGCTGgGa	147356	147380	25	3
BART10-3P	BART10	TACATAACCATGGAGTTGGCTGTat	147356	147380	25	4
BART10-3P	BART10	TACATAACCATGGAGTTGGCTGTGc	147356	147380	25	7
BART10-3P	BART10	TACATAACCATGGAGTTGGCTGTGG	147356	147380	25	22
BART10-3P	BART10	TACATAACCATGGAGTTGGCTGTta	147356	147380	25	3
BART10-3P	BART10	ACATAACCATGGAGTTGGC	147357	147375	19	3
BART10-3P	BART10	ACATAACCATGGAGTTtcC	147357	147375	19	3
BART10-3P	BART10	ACATAACCATGGAGTacaCT	147357	147376	20	3
BART10-3P	BART10	ACATAACCATGGAGTTGaCT	147357	147376	20	13
BART10-3P	BART10	ACATAACCATGGAGTTGGCT	147357	147376	20	34
BART10-3P	BART10	ACATAACCATGGAGTTaGCTG	147357	147377	21	11
BART10-3P	BART10	ACATAACCATGGAGTTGaCct	147357	147377	21	3
BART10-3P	BART10	ACATAACCATGGAGTTGaCTG	147357	147377	21	8
BART10-3P	BART10	ACATAACCATGGAGTTGatTG	147357	147377	21	3
BART10-3P	BART10	ACATAACCATGGAGTTGcCcc	147357	147377	21	4
BART10-3P	BART10	ACATAACCATGGAGTTGGCaa	147357	147377	21	3
BART10-3P	BART10	ACATAACCATGGAGTTGGCac	147357	147377	21	4
BART10-3P	BART10	ACATAACCATGGAGTTGGCat	147357	147377	21	4
BART10-3P	BART10	ACATAACCATGGAGTTGGCTG	147357	147377	21	25
BART10-3P	BART10	ACATAACCATGGAGccaGCTGT	147357	147378	22	4
BART10-3P	BART10	ACATAACCATGGAGccGGCTGT	147357	147378	22	6
BART10-3P	BART10	ACATAACCATGGAGTTaaCcGa	147357	147378	22	5
BART10-3P	BART10	ACATAACCATGGAGTTaaCTGa	147357	147378	22	8
BART10-3P	BART10	ACATAACCATGGAGTTaaCTGT	147357	147378	22	24
BART10-3P	BART10	ACATAACCATGGAGTTacCTGT	147357	147378	22	4
BART10-3P	BART10	ACATAACCATGGAGTTaGCaGa	147357	147378	22	6
BART10-3P	BART10	ACATAACCATGGAGTTaGCaGT	147357	147378	22	15
BART10-3P	BART10	ACATAACCATGGAGTTaGCcaT	147357	147378	22	11
BART10-3P	BART10	ACATAACCATGGAGTTaGCcGa	147357	147378	22	5
BART10-3P	BART10	ACATAACCATGGAGTTaGCcGT	147357	147378	22	6
BART10-3P	BART10	ACATAACCATGGAGTTaGCTGa	147357	147378	22	13
BART10-3P	BART10	ACATAACCATGGAGTTaGCTGT	147357	147378	22	57
BART10-3P	BART10	ACATAACCATGGAGTTcGCTGa	147357	147378	22	4
BART10-3P	BART10	ACATAACCATGGAGTTcGCTGT	147357	147378	22	16
BART10-3P	BART10	ACATAACCATGGAGTTGaCaGT	147357	147378	22	4
BART10-3P	BART10	ACATAACCATGGAGTTGaCcGa	147357	147378	22	4

BART10-3P	BART10	ACATAACCATGGAGTTGaCcGT	147357	147378	22	16
BART10-3P	BART10	ACATAACCATGGAGTTGaCTaa	147357	147378	22	7
BART10-3P	BART10	ACATAACCATGGAGTTGaCTGa	147357	147378	22	85
BART10-3P	BART10	ACATAACCATGGAGTTGaCTGc	147357	147378	22	7
BART10-3P	BART10	ACATAACCATGGAGTTGaCTGg	147357	147378	22	6
BART10-3P	BART10	ACATAACCATGGAGTTGaCTGT	147357	147378	22	406
BART10-3P	BART10	ACATAACCATGGAGTTGagTGT	147357	147378	22	7
BART10-3P	BART10	ACATAACCATGGAGTTGatTGT	147357	147378	22	13
BART10-3P	BART10	ACATAACCATGGAGTTGcCTGa	147357	147378	22	12
BART10-3P	BART10	ACATAACCATGGAGTTGcCTGT	147357	147378	22	18
BART10-3P	BART10	ACATAACCATGGAGTTGGaTGa	147357	147378	22	4
BART10-3P	BART10	ACATAACCATGGAGTTGGaTGc	147357	147378	22	3
BART10-3P	BART10	ACATAACCATGGAGTTGGaTGT	147357	147378	22	7
BART10-3P	BART10	ACATAACCATGGAGTTGGCaGa	147357	147378	22	37
BART10-3P	BART10	ACATAACCATGGAGTTGGCaGc	147357	147378	22	4
BART10-3P	BART10	ACATAACCATGGAGTTGGCaGT	147357	147378	22	106
BART10-3P	BART10	ACATAACCATGGAGTTGGCcaT	147357	147378	22	5
BART10-3P	BART10	ACATAACCATGGAGTTGGCcGa	147357	147378	22	6
BART10-3P	BART10	ACATAACCATGGAGTTGGCcGT	147357	147378	22	87
BART10-3P	BART10	ACATAACCATGGAGTTGGCTaa	147357	147378	22	23
BART10-3P	BART10	ACATAACCATGGAGTTGGCTac	147357	147378	22	10
BART10-3P	BART10	ACATAACCATGGAGTTGGCTaT	147357	147378	22	19
BART10-3P	BART10	ACATAACCATGGAGTTGGCTca	147357	147378	22	7
BART10-3P	BART10	ACATAACCATGGAGTTGGCTGa	147357	147378	22	25
BART10-3P	BART10	ACATAACCATGGAGTTGGCTGc	147357	147378	22	22
BART10-3P	BART10	ACATAACCATGGAGTTGGCTGg	147357	147378	22	8
BART10-3P	BART10	ACATAACCATGGAGTTGGCTGT	147357	147378	22	105
BART10-3P	BART10	ACATAACCATGGAGTTGGCTtg	147357	147378	22	3
BART10-3P	BART10	ACATAACCATGGAGTTGGgTGT	147357	147378	22	8
BART10-3P	BART10	ACATAACCATGGAGTTGtaTGT	147357	147378	22	3
BART10-3P	BART10	ACATAACCATGGAGTTGtCTGa	147357	147378	22	3
BART10-3P	BART10	ACATAACCATGGAGTTGtCTGT	147357	147378	22	11
BART10-3P	BART10	ACATAACCATGGAGTTGaCcGTG	147357	147379	23	3
BART10-3P	BART10	ACATAACCATGGAGTTGaCTGaG	147357	147379	23	10
BART10-3P	BART10	ACATAACCATGGAGTTGaCTGTG	147357	147379	23	15
BART10-3P	BART10	ACATAACCATGGAGTTGcCTGTG	147357	147379	23	9
BART10-3P	BART10	ACATAACCATGGAGTTGGacGTG	147357	147379	23	3
BART10-3P	BART10	ACATAACCATGGAGTTGGCTaTG	147357	147379	23	8
BART10-3P	BART10	ACATAACCATGGAGTTGGCTGac	147357	147379	23	3
BART10-3P	BART10	ACATAACCATGGAGTTGGCTGgG	147357	147379	23	4
BART10-3P	BART10	ACATAACCATGGAGTTGGCTGTa	147357	147379	23	6
BART10-3P	BART10	ACATAACCATGGAGTTGGCTGTG	147357	147379	23	27
BART10-3P	BART10	ACATAACCATGGAGTTGGCTGTt	147357	147379	23	8
BART10-3P	BART10	ACATAACCATGGAGTTGGtTGTG	147357	147379	23	3
BART10-3P	BART10	ACATAACCATGGAGTTGGCaGTaG	147357	147380	24	5
BART10-3P	BART10	ACATAACCATGGAGTTGGCTaTaa	147357	147380	24	4
BART10-3P	BART10	ACATAACCATGGAGTTGGCTGTaa	147357	147380	24	9
BART10-3P	BART10	ACATAACCATGGAGTTGGCTGTGc	147357	147380	24	6
BART10-3P	BART10	ACATAACCATGGAGTTGGCTGTGG	147357	147380	24	6
BART10-3P	BART10	ACATAACCATGGAGTTGGCTGTta	147357	147380	24	7
BART10-3P	BART10	ACATAACCATGGAGTTGGCTGTtc	147357	147380	24	4
BART10-3P	BART10	CATAACCATGGAGTTGaCcGT	147358	147378	21	5
BART10-3P	BART10	CATAACCATGGAGTTGaCTGT	147358	147378	21	10
BART10-3P	BART10	CATAACCATGGAGTTGGCaGT	147358	147378	21	5
BART10-3P	BART10	CATAACCATGGAGTTGGCTGT	147358	147378	21	9
BART10-3P	BART10	CATAACCATGGAGTTGGgTGT	147358	147378	21	4
BART10-3P	BART10	CATAACCATGGAGTTGGCTGTaa	147358	147380	23	11
BART10-3P	BART10	CATAACCATGGAGTTGGCTGTGG	147358	147380	23	10
BART10-3P	BART10	CATAACCATGGAGTTGGCcGTaaT	147358	147381	24	5
BART10-3P	BART10	tTAACCATGGAGTTGGCTaT	147359	147378	20	3
BART10-3P	BART10	ATAACCATGGAGTTGGCTGTaa	147359	147380	22	3
BART10-3P	BART10	ATAACCATGGAGTTGGCTGTat	147359	147380	22	5
BART10-3P	BART10	ATAACCATGGAGTTGGCTGTGG	147359	147380	22	4
BART10-3P	BART10	ATAACCATGGAGTTGGCTGTGt	147359	147380	22	3
BART11-5P	BART11-5P	TCAGACAGTTTGGTGCaCTAG	147537	147557	21	6
BART11-5P	BART11-5P	TCAGACAGTTTGGTGCGCTAc	147537	147557	21	6
BART11-5P	BART11-5P	TCAGACAGTTTGGTGCGCTAGT	147537	147558	22	32
BART11-5P	BART11-5P	TCAGACAGTTTGGTGCGCTctg	147537	147558	22	60

BART11-5P	BART11-5P	TCAGACAGTTTGGTGCGCTgGT	147537	147558	22	3
BART11-5P	BART11-5P	TCAGACAGTTTGGTGCGCTtGT	147537	147558	22	4
BART11-5P	BART11-5P	TCAGACAGTTTGGTGCGaTAGTT	147537	147559	23	8
BART11-5P	BART11-5P	TCAGACAGTTTGGTGCGCTAGTg	147537	147559	23	3
BART11-5P	BART11-5P	TCAGACAGTTTGGTGCGCTAGTT	147537	147559	23	89
BART11-5P	BART11-5P	TCAGACAGTTTGGTGCGCTgtcT	147537	147559	23	3
BART11-5P	BART11-5P	TCAGACAGTTTGGTGCGCTttTT	147537	147559	23	3
BART11-5P	BART11-5P	TCAGACAGTTTGGTGCGCgAGTTG	147537	147560	24	10
BART11-5P	BART11-5P	TCAGACAGTTTGGTGCGCTAGTat	147537	147560	24	14
BART11-5P	BART11-5P	TCAGACAGTTTGGTGCGCTAGTgc	147537	147560	24	6
BART11-5P	BART11-5P	TCAGACAGTTTGGTGCGCTAGTTa	147537	147560	24	11
BART11-5P	BART11-5P	TCAGACAGTTTGGTGCGCTAGTTG	147537	147560	24	30
BART11-5P	BART11-5P	TCAGACAGTTTGGTGCGCTAGTTt	147537	147560	24	7
BART11-5P	BART11-5P	TCAGACAGTTTGGTGCGCTAGTTaT	147537	147561	25	3
BART11-5P	BART11-5P	TCAGACAGTTTGGTGCGCTAGTTGg	147537	147561	25	4
BART11-5P	BART11-5P	TCAGACAGTTTGGTGCGCTAGTTGT	147537	147561	25	26
BART11-5P	BART11-5P	TCAGACAGTTTGGTGCGCTgGTTGT	147537	147561	25	5
BART11-5P	BART11-5P	TCAGACAGTTTGGTGCGCTAGaTGTG	147537	147562	26	17
BART11-3P	BART11-3P	AACGCACACCAGGCTGACaGCCT	147574	147596	23	3
BART11-3P	BART11-3P	ACGCACACCAGGCTGACTG	147575	147593	19	10
BART11-3P	BART11-3P	ACGCACACCAGGCTaACTGC	147575	147594	20	26
BART11-3P	BART11-3P	ACGCACACCAGGCTcACTGC	147575	147594	20	4
BART11-3P	BART11-3P	ACGCACACCAGGCTGACacC	147575	147594	20	8
BART11-3P	BART11-3P	ACGCACACCAGGCTGACaGC	147575	147594	20	17
BART11-3P	BART11-3P	ACGCACACCAGGCTGACcaC	147575	147594	20	7
BART11-3P	BART11-3P	ACGCACACCAGGCTGACcGC	147575	147594	20	26
BART11-3P	BART11-3P	ACGCACACCAGGCTGACcGg	147575	147594	20	17
BART11-3P	BART11-3P	ACGCACACCAGGCTGACgcC	147575	147594	20	21
BART11-3P	BART11-3P	ACGCACACCAGGCTGACgGC	147575	147594	20	4
BART11-3P	BART11-3P	ACGCACACCAGGCTGACTaa	147575	147594	20	15
BART11-3P	BART11-3P	ACGCACACCAGGCTGACTaC	147575	147594	20	3
BART11-3P	BART11-3P	ACGCACACCAGGCTGACTGa	147575	147594	20	18
BART11-3P	BART11-3P	ACGCACACCAGGCTGACTGC	147575	147594	20	145
BART11-3P	BART11-3P	ACGCACACCAGGCTGACTGg	147575	147594	20	6
BART11-3P	BART11-3P	ACGCACACCAGGCTGACTGt	147575	147594	20	10
BART11-3P	BART11-3P	ACGCACACCAGGCTGAtaGC	147575	147594	20	4
BART11-3P	BART11-3P	ACGCACACCAGGCTGcCTGC	147575	147594	20	12
BART11-3P	BART11-3P	ACGCACACCAGGCTGgCTGC	147575	147594	20	7
BART11-3P	BART11-3P	ACGCACACCAGGCTGtCTGC	147575	147594	20	6
BART11-3P	BART11-3P	ACGCACACCAGGCTtACTGC	147575	147594	20	6
BART11-3P	BART11-3P	ACGCACACCAGtaTGACTGC	147575	147594	20	3
BART11-3P	BART11-3P	ACGCACACCAGGCTGAaTGCC	147575	147595	21	7
BART11-3P	BART11-3P	ACGCACACCAGGCTGACacCC	147575	147595	21	3
BART11-3P	BART11-3P	ACGCACACCAGGCTGACaGCC	147575	147595	21	7
BART11-3P	BART11-3P	ACGCACACCAGGCTGACcaCC	147575	147595	21	16
BART11-3P	BART11-3P	ACGCACACCAGGCTGACcagC	147575	147595	21	3
BART11-3P	BART11-3P	ACGCACACCAGGCTGACccCC	147575	147595	21	3
BART11-3P	BART11-3P	ACGCACACCAGGCTGACcGCC	147575	147595	21	11
BART11-3P	BART11-3P	ACGCACACCAGGCTGACgcCC	147575	147595	21	5
BART11-3P	BART11-3P	ACGCACACCAGGCTGACTaCC	147575	147595	21	33
BART11-3P	BART11-3P	ACGCACACCAGGCTGACTagC	147575	147595	21	3
BART11-3P	BART11-3P	ACGCACACCAGGCTGACTGaC	147575	147595	21	5
BART11-3P	BART11-3P	ACGCACACCAGGCTGACTGCC	147575	147595	21	70
BART11-3P	BART11-3P	ACGCACACCAGGCTGAtaGCC	147575	147595	21	3
BART11-3P	BART11-3P	ACGCACACCAGGCTGcCTGCC	147575	147595	21	16
BART11-3P	BART11-3P	ACGCACACCAGGCTGgCTaCC	147575	147595	21	6
BART11-3P	BART11-3P	ACGCACACCAGGCTGgCTGCC	147575	147595	21	3
BART11-3P	BART11-3P	ACGCACACCAGGCTGAaTGCCT	147575	147596	22	12
BART11-3P	BART11-3P	ACGCACACCAGGCTGACaGCCT	147575	147596	22	12
BART11-3P	BART11-3P	ACGCACACCAGGCTGACcaCCT	147575	147596	22	13
BART11-3P	BART11-3P	ACGCACACCAGGCTGACcGCCT	147575	147596	22	7
BART11-3P	BART11-3P	ACGCACACCAGGCTGACTaCCT	147575	147596	22	34
BART11-3P	BART11-3P	ACGCACACCAGGCTGACTGCaT	147575	147596	22	3
BART11-3P	BART11-3P	ACGCACACCAGGCTGACTGCCa	147575	147596	22	7
BART11-3P	BART11-3P	ACGCACACCAGGCTGACTGCCT	147575	147596	22	159
BART11-3P	BART11-3P	ACGCACACCAGGCTGACTtCCT	147575	147596	22	3
BART11-3P	BART11-3P	ACGCACACCAGGCTGACTtgCT	147575	147596	22	4
BART11-3P	BART11-3P	ACGCACACCAGGCTGAtTGCCT	147575	147596	22	4

BART11-3P	BART11-3P	ACGCACACCAGGCTGACaGCCaT	147575	147597	23	3
BART11-3P	BART11-3P	ACGCACACCAGGCTGACTaCCag	147575	147597	23	3
BART11-3P	BART11-3P	ACGCACACCAGGCTGACTGCacT	147575	147597	23	4
BART11-3P	BART11-3P	ACGCACACCAGGCTGACTGCCaa	147575	147597	23	5
BART11-3P	BART11-3P	ACGCACACCAGGCTGACTGCCac	147575	147597	23	5
BART11-3P	BART11-3P	ACGCACACCAGGCTGACTGCCaT	147575	147597	23	7
BART11-3P	BART11-3P	ACGCACACCAGGCTGACTGCCga	147575	147597	23	4
BART11-3P	BART11-3P	ACGCACACCAGGCTGACTGCCTa	147575	147597	23	3
BART11-3P	BART11-3P	ACGCACACCAGGCTGACTGCCTT	147575	147597	23	3
BART11-3P	BART11-3P	ACGCACACCAGGCTGACTGCCagA	147575	147598	24	4
BART11-3P	BART11-3P	ACGCACACCAGGCTGACTGCCaTA	147575	147598	24	14
BART11-3P	BART11-3P	ACGCACACCAGGCTGACTGCCcaA	147575	147598	24	3
BART11-3P	BART11-3P	ACGCACACCAGGCTGACTGCCTaA	147575	147598	24	6
BART11-3P	BART11-3P	ACGCACACCAGGCTGACTGCCTTA	147575	147598	24	21
BART11-3P	BART11-3P	ACGCACACCAGGCTGACTGCCagAGCAG	147575	147602	28	3
BART12-5P		gACCCGCCCATCACCACCaGA	147900	147920	21	12
BART12-5P		gACCCGCCCATCACCACCGGACAG	147900	147923	24	11
BART12-5P		ACCCGCCCATCACCACCGGA	147901	147920	20	5
BART12-5P		ACCCGCCCATCACCACaGGAC	147901	147921	21	3
BART12-5P		ACCCGCCCATCACCACCGGAC	147901	147921	21	19
BART12-5P		ACCCGCCCATCACCACCaGtaA	147901	147922	22	3
BART12-5P		ACCCGCCCATCACCACCGGACA	147901	147922	22	7
BART12-5P		ACCCGCCCATCACCACtGaACA	147901	147922	22	3
BART12-5P		ACCCGCCCATCACCACCGaACAc	147901	147923	23	3
BART12-5P		ACCCGCCCATCACCACCGGACAc	147901	147923	23	8
BART12-5P		ACCCGCCCATCACCACCGGACAG	147901	147923	23	8
BART12-3P	BART12	TCCTGTGGTGTTTGGTGTGG	147936	147955	20	4
BART12-3P	BART12	TCCTGTGGTGTTTGGTGTaGT	147936	147956	21	4
BART12-3P	BART12	TCCTGTGGTGTTTGGTGTGGT	147936	147956	21	4
BART12-3P	BART12	TCCTGTGGTGTTTGGTGTGGTT	147936	147957	22	16
BART12-3P	BART12	TCCTGTGGTGTTTaGTGTGGTTT	147936	147958	23	3
BART12-3P	BART12	TCCTGTGGTGTTTGGTGcGGTTa	147936	147958	23	4
BART12-3P	BART12	TCCTGTGGTGTTTGGTGgGGTTa	147936	147958	23	3
BART12-3P	BART12	TCCTGTGGTGTTTGGTGTGaTcc	147936	147958	23	3
BART12-3P	BART12	TCCTGTGGTGTTTGGTGTGGcTT	147936	147958	23	6
BART12-3P	BART12	TCCTGTGGTGTTTGGTGTGGTaa	147936	147958	23	4
BART12-3P	BART12	TCCTGTGGTGTTTGGTGTGGTaT	147936	147958	23	5
BART12-3P	BART12	TCCTGTGGTGTTTGGTGTGGTTa	147936	147958	23	9
BART12-3P	BART12	TCCTGTGGTGTTTGGTGTGGTTT	147936	147958	23	25
BART12-3P	BART12	TCCTGTGGTGTTTGGTGTaGTTaT	147936	147959	24	3
BART12-3P	BART12	TCCTGTGGTGTTTGGTGTGGTaTa	147936	147959	24	7
BART12-3P	BART12	TCCTGTGGTGTTTGGTGTGGTTaa	147936	147959	24	4
BART12-3P	BART12	TCCTGTGGTGTTTGGTGTGGTTac	147936	147959	24	3
BART12-3P	BART12	TCCTGTGGTGTTTGGTGTGGTTaT	147936	147959	24	5
BART12-3P	BART12	TCCTGTGGTGTTTGGTGTGGTTTa	147936	147959	24	16
BART12-3P	BART12	TCCTGTGGTGTTTGGTGTGGTTTT	147936	147959	24	9
BART12-3P	BART12	TCCTGTGGTGTTTGGTGTGGTccTa	147936	147960	25	5
BART19-5P	BART19-5P	CAACATTCCCCGCAAACAcGA	148213	148233	21	3
BART19-5P	BART19-5P	CAACATTCCCCGCAAACAcGAC	148213	148234	22	3
BART19-5P	BART19-5P	ACATTCCCCGCAAACAcaA	148215	148233	19	14
BART19-5P	BART19-5P	ACATTCCCCGCAAACAcGA	148215	148233	19	425
BART19-5P	BART19-5P	ACATTCCCCGCAAACAcGc	148215	148233	19	7
BART19-5P	BART19-5P	ACATTCCCCGCAAACATcA	148215	148233	19	9
BART19-5P	BART19-5P	ACATTCCCCGCAAACATGA	148215	148233	19	5
BART19-5P	BART19-5P	ACATTCCCCGCAAACAcaAC	148215	148234	20	8
BART19-5P	BART19-5P	ACATTCCCCGCAAACAcatC	148215	148234	20	6
BART19-5P	BART19-5P	ACATTCCCCGCAAACAcGAa	148215	148234	20	4
BART19-5P	BART19-5P	ACATTCCCCGCAAACAcGAC	148215	148234	20	368
BART19-5P	BART19-5P	ACATTCCCCGCAAACATaAC	148215	148234	20	6
BART19-5P	BART19-5P	ACATTCCCCGCAAACATcAC	148215	148234	20	38
BART19-5P	BART19-5P	ACATTCCCCGCAAACATGAC	148215	148234	20	17
BART19-5P	BART19-5P	ACATTCCCCGCAAACAaGACA	148215	148235	21	11
BART19-5P	BART19-5P	ACATTCCCCGCAAACAcaACA	148215	148235	21	69
BART19-5P	BART19-5P	ACATTCCCCGCAAACAcacCA	148215	148235	21	4
BART19-5P	BART19-5P	ACATTCCCCGCAAACAccgCA	148215	148235	21	7
BART19-5P	BART19-5P	ACATTCCCCGCAAACAcGACA	148215	148235	21	966
BART19-5P	BART19-5P	ACATTCCCCGCAAACAcGACc	148215	148235	21	80
BART19-5P	BART19-5P	ACATTCCCCGCAAACAcGACg	148215	148235	21	5

BART19-5P	BART19-5P	ACATTCCCCGCAAACAcGAtA	148215	148235	21	10
BART19-5P	BART19-5P	ACATTCCCCGCAAACAcGcCA	148215	148235	21	23
BART19-5P	BART19-5P	ACATTCCCCGCAAACAcGgCA	148215	148235	21	8
BART19-5P	BART19-5P	ACATTCCCCGCAAACActcCt	148215	148235	21	4
BART19-5P	BART19-5P	ACATTCCCCGCAAACAgaACA	148215	148235	21	9
BART19-5P	BART19-5P	ACATTCCCCGCAAACAgGACA	148215	148235	21	28
BART19-5P	BART19-5P	ACATTCCCCGCAAACATcACA	148215	148235	21	4
BART19-5P	BART19-5P	ACATTCCCCGCAAACATGACA	148215	148235	21	18
BART19-5P	BART19-5P	ACATTCCCCGCAAACATGAtc	148215	148235	21	3
BART19-5P	BART19-5P	ACATTCCCCGCAAACATGgtA	148215	148235	21	4
BART19-5P	BART19-5P	ACATTCCCCGCAAACATGtgc	148215	148235	21	20
BART19-5P	BART19-5P	ACATTCCCCGCAAACccaACA	148215	148235	21	14
BART19-5P	BART19-5P	ACATTCCCCGCAAACccGACA	148215	148235	21	63
BART19-5P	BART19-5P	ACATTCCCCGCAAACccGACc	148215	148235	21	28
BART19-5P	BART19-5P	ACATTCCCCGCAAACgcaACA	148215	148235	21	15
BART19-5P	BART19-5P	ACATTCCCCGCAAACgcGACA	148215	148235	21	15
BART19-5P	BART19-5P	ACATTCCCCGCAAACgcGcCA	148215	148235	21	5
BART19-5P	BART19-5P	ACATTCCCCGCAAACtcGACA	148215	148235	21	5
BART19-5P	BART19-5P	cCATTCCCCGCAAACATGAgg	148215	148235	21	3
BART19-5P	BART19-5P	ACATTCCCCGCAAACAaGACAa	148215	148236	22	4
BART19-5P	BART19-5P	ACATTCCCCGCAAACAaGACAT	148215	148236	22	254
BART19-5P	BART19-5P	ACATTCCCCGCAAACAaGACga	148215	148236	22	3
BART19-5P	BART19-5P	ACATTCCCCGCAAACAcaACAa	148215	148236	22	3
BART19-5P	BART19-5P	ACATTCCCCGCAAACAcaACAT	148215	148236	22	270
BART19-5P	BART19-5P	ACATTCCCCGCAAACAcaACga	148215	148236	22	4
BART19-5P	BART19-5P	ACATTCCCCGCAAACAccACAa	148215	148236	22	3
BART19-5P	BART19-5P	ACATTCCCCGCAAACAccACAT	148215	148236	22	23
BART19-5P	BART19-5P	ACATTCCCCGCAAACAccACca	148215	148236	22	4
BART19-5P	BART19-5P	ACATTCCCCGCAAACAcGACAa	148215	148236	22	145
BART19-5P	BART19-5P	ACATTCCCCGCAAACAcGACAc	148215	148236	22	118
BART19-5P	BART19-5P	ACATTCCCCGCAAACAcGACAg	148215	148236	22	9
BART19-5P	BART19-5P	ACATTCCCCGCAAACAcGACAT	148215	148236	22	1842
BART19-5P	BART19-5P	ACATTCCCCGCAAACAcGACca	148215	148236	22	22
BART19-5P	BART19-5P	ACATTCCCCGCAAACAcGACga	148215	148236	22	26
BART19-5P	BART19-5P	ACATTCCCCGCAAACAcGACgc	148215	148236	22	11
BART19-5P	BART19-5P	ACATTCCCCGCAAACAcGACgT	148215	148236	22	36
BART19-5P	BART19-5P	ACATTCCCCGCAAACAcGACta	148215	148236	22	5
BART19-5P	BART19-5P	ACATTCCCCGCAAACAcGACtg	148215	148236	22	7
BART19-5P	BART19-5P	ACATTCCCCGCAAACAcGACtT	148215	148236	22	3
BART19-5P	BART19-5P	ACATTCCCCGCAAACAcGAtAT	148215	148236	22	4
BART19-5P	BART19-5P	ACATTCCCCGCAAACAcGAtgT	148215	148236	22	20
BART19-5P	BART19-5P	ACATTCCCCGCAAACAcGcCAT	148215	148236	22	118
BART19-5P	BART19-5P	ACATTCCCCGCAAACAcGgCAT	148215	148236	22	3
BART19-5P	BART19-5P	ACATTCCCCGCAAACAcGtCAT	148215	148236	22	4
BART19-5P	BART19-5P	ACATTCCCCGCAAACActACAT	148215	148236	22	10
BART19-5P	BART19-5P	ACATTCCCCGCAAACActcCAT	148215	148236	22	6
BART19-5P	BART19-5P	ACATTCCCCGCAAACAgGACAT	148215	148236	22	33
BART19-5P	BART19-5P	ACATTCCCCGCAAACATcACAT	148215	148236	22	5
BART19-5P	BART19-5P	ACATTCCCCGCAAACATGACAT	148215	148236	22	210
BART19-5P	BART19-5P	ACATTCCCCGCAAACATGAtgT	148215	148236	22	4
BART19-5P	BART19-5P	ACATTCCCCGCAAACATGgtAT	148215	148236	22	3
BART19-5P	BART19-5P	ACATTCCCCGCAAAtgTGACAT	148215	148236	22	3
BART19-5P	BART19-5P	gCATTCCCCGCAAACATGACAT	148215	148236	22	4
BART19-5P	BART19-5P	ACATTCCCCGCAAACAcaACAcG	148215	148237	23	19
BART19-5P	BART19-5P	ACATTCCCCGCAAACAcaACATa	148215	148237	23	9
BART19-5P	BART19-5P	ACATTCCCCGCAAACAcaACATG	148215	148237	23	78
BART19-5P	BART19-5P	ACATTCCCCGCAAACAcaACATt	148215	148237	23	5
BART19-5P	BART19-5P	ACATTCCCCGCAAACAcaACcTG	148215	148237	23	7
BART19-5P	BART19-5P	ACATTCCCCGCAAACAcagCATG	148215	148237	23	9
BART19-5P	BART19-5P	ACATTCCCCGCAAACAcGACAaa	148215	148237	23	14
BART19-5P	BART19-5P	ACATTCCCCGCAAACAcGACAac	148215	148237	23	8
BART19-5P	BART19-5P	ACATTCCCCGCAAACAcGACAat	148215	148237	23	15
BART19-5P	BART19-5P	ACATTCCCCGCAAACAcGACAca	148215	148237	23	65
BART19-5P	BART19-5P	ACATTCCCCGCAAACAcGACAcc	148215	148237	23	30
BART19-5P	BART19-5P	ACATTCCCCGCAAACAcGACAcG	148215	148237	23	253
BART19-5P	BART19-5P	ACATTCCCCGCAAACAcGACAct	148215	148237	23	27
BART19-5P	BART19-5P	ACATTCCCCGCAAACAcGACAga	148215	148237	23	3
BART19-5P	BART19-5P	ACATTCCCCGCAAACAcGACAgt	148215	148237	23	9

BART19-5P	BART19-5P	ACATTCCCCGCAAACAcGACATa	148215	148237	23	140
BART19-5P	BART19-5P	ACATTCCCCGCAAACAcGACATc	148215	148237	23	19
BART19-5P	BART19-5P	ACATTCCCCGCAAACAcGACATG	148215	148237	23	633
BART19-5P	BART19-5P	ACATTCCCCGCAAACAcGACATt	148215	148237	23	50
BART19-5P	BART19-5P	ACATTCCCCGCAAACAcGACcTG	148215	148237	23	10
BART19-5P	BART19-5P	ACATTCCCCGCAAACAcGACgaG	148215	148237	23	3
BART19-5P	BART19-5P	ACATTCCCCGCAAACAcGACgTG	148215	148237	23	9
BART19-5P	BART19-5P	ACATTCCCCGCAAACAcGACtTG	148215	148237	23	7
BART19-5P	BART19-5P	ACATTCCCCGCAAACAcGAtATG	148215	148237	23	3
BART19-5P	BART19-5P	ACATTCCCCGCAAACAcGAttTG	148215	148237	23	3
BART19-5P	BART19-5P	ACATTCCCCGCAAACAcGgCATG	148215	148237	23	9
BART19-5P	BART19-5P	ACATTCCCCGCAAACATGACAat	148215	148237	23	3
BART19-5P	BART19-5P	ACATTCCCCGCAAACATGACATG	148215	148237	23	13
BART19-5P	BART19-5P	ACATTCCCCGCAAAtgTGACATG	148215	148237	23	6
BART19-5P	BART19-5P	ACATTCCCCGCAAACAcacCATGG	148215	148238	24	3
BART19-5P	BART19-5P	ACATTCCCCGCAAACAcGAaATGG	148215	148238	24	9
BART19-5P	BART19-5P	ACATTCCCCGCAAACAcGACAcaG	148215	148238	24	3
BART19-5P	BART19-5P	ACATTCCCCGCAAACAcGACAcGG	148215	148238	24	8
BART19-5P	BART19-5P	ACATTCCCCGCAAACAcGACAgtG	148215	148238	24	4
BART19-5P	BART19-5P	ACATTCCCCGCAAACAcGACATGa	148215	148238	24	6
BART19-5P	BART19-5P	ACATTCCCCGCAAACAcGACATGc	148215	148238	24	17
BART19-5P	BART19-5P	ACATTCCCCGCAAACAcGACATGG	148215	148238	24	474
BART19-5P	BART19-5P	ACATTCCCCGCAAACAcGACATGt	148215	148238	24	10
BART19-5P	BART19-5P	ACATTCCCCGCAAACAcGACATtG	148215	148238	24	3
BART19-5P	BART19-5P	ACATTCCCCGCAAACAcGACgTGG	148215	148238	24	5
BART19-5P	BART19-5P	ACATTCCCCGCAAACAcGAgATGG	148215	148238	24	3
BART19-5P	BART19-5P	ACATTCCCCGCAAACAcGAtATGG	148215	148238	24	8
BART19-5P	BART19-5P	ACATTCCCCGCAAACAcGcCATGG	148215	148238	24	23
BART19-5P	BART19-5P	ACATTCCCCGCAAACAcGgCATGG	148215	148238	24	36
BART19-5P	BART19-5P	ACATTCCCCGCAAACAcGtCATGG	148215	148238	24	9
BART19-5P	BART19-5P	ACATTCCCCGCAAACATGACATGG	148215	148238	24	11
BART19-5P	BART19-5P	ACATTCCCCGCAAACAcGACAcacG	148215	148239	25	3
BART19-5P	BART19-5P	ACATTCCCCGCAAACAcGACATGcG	148215	148239	25	4
BART19-5P	BART19-5P	ACATTCCCCGCAAACAcGACATGGa	148215	148239	25	5
BART19-5P	BART19-5P	ACATTCCCCGCAAACAcGACATGGc	148215	148239	25	8
BART19-5P	BART19-5P	ACATTCCCCGCAAACAcGACATGGG	148215	148239	25	19
BART19-5P	BART19-5P	ACATTCCCCGCAAACAcGACATGGt	148215	148239	25	7
BART19-5P	BART19-5P	ACATTCCCCGCAAACAcGACATGtG	148215	148239	25	3
BART19-5P	BART19-5P	ACATTCCCCGCAAACAcGACATtGG	148215	148239	25	5
BART19-5P	BART19-5P	CATTCCCCGCAAACAcaACAT	148216	148236	21	3
BART19-5P	BART19-5P	ATTCCCCGCAAACAccACAT	148217	148236	20	9
BART19-5P	BART19-5P	ATTCCCCGCAAACAcGACATG	148217	148237	21	3
BART19-3P	BART19-3P	TGTTTTGTTTGCTTGGaAATGC	148252	148273	22	3
BART19-3P	BART19-3P	TGTTTTGTTTGCTTGGGAATGCc	148252	148274	23	4
BART19-3P	BART19-3P	TGTTTTGTTTGCTTGGGAATGCT	148252	148274	23	4
BART19-3P	BART19-3P	TGTTTTGTTTGCTTGGGAATGCTa	148252	148275	24	4
BART19-3P	BART19-3P	TTTTGTTTGCTTGGGAATGCT	148254	148274	21	3
BART19-3P	BART19-3P	TTTTGTTTGCTTGGGAATGCTC	148254	148275	22	12
BART19-3P	BART19-3P	TTTTGTTTGCTTGGGAATGCTCT	148254	148276	23	3
BART19-3P	BART19-3P	TTTTGTTTGCTTGGGAATGCTCga	148254	148277	24	3
BART20-5P	BART20-5P	TAGCAGGCATGTCTTCcTca	148339	148358	20	3
BART20-5P	BART20-5P	TAGCAGGCATGTCTTCATTCC	148339	148359	21	3
BART20-3P	BART20-3P	CATGAAGGCACAGCCTGTTA	148374	148393	20	7
BART20-3P	BART20-3P	CATGAAGGCACAGCCTGTTc	148374	148393	20	3
BART20-3P	BART20-3P	CATGAAGGCACAGCCTGTTAC	148374	148394	21	15
BART20-3P	BART20-3P	CATGAAGGCACAGCCTaTTACC	148374	148395	22	20
BART20-3P	BART20-3P	CATGAAGGCACAGCCTGaaACC	148374	148395	22	3
BART20-3P	BART20-3P	CATGAAGGCACAGCCTGgTACC	148374	148395	22	4
BART20-3P	BART20-3P	CATGAAGGCACAGCCTGTcACC	148374	148395	22	4
BART20-3P	BART20-3P	CATGAAGGCACAGCCTGTTACC	148374	148395	22	99
BART20-3P	BART20-3P	CATGAAGGCACAGCCTGTTgCC	148374	148395	22	5
BART20-3P	BART20-3P	CATGAAGGCACAGCCTGgTACCA	148374	148396	23	3
BART20-3P	BART20-3P	CATGAAGGCACAGCCTGTTACCATaac	148374	148400	27	3
BART13-5P	BART13-star	AACCGGCTCGTGGCTCaTA	148526	148544	19	11
BART13-5P	BART13-star	AACCGGCTCGTGGCTCGcA	148526	148544	19	3
BART13-5P	BART13-star	AACCGGCTCGTGGCTCGTAa	148526	148545	20	9
BART13-5P	BART13-star	AACCGGCTCGTGGCTCGTAC	148526	148545	20	29
BART13-5P	BART13-star	AACCGGCTCGTGGCTCGTAt	148526	148545	20	7

BART13-5P	BART13-star	AACCGGCTCGTGGCTCGcACA	148526	148546	21	5
BART13-5P	BART13-star	AACCGGCTCGTGGCTCGcAgA	148526	148546	21	4
BART13-5P	BART13-star	AACCGGCTCGTGGCTCGgcCA	148526	148546	21	3
BART13-5P	BART13-star	AACCGGCTCGTGGCTCGTAaA	148526	148546	21	8
BART13-5P	BART13-star	AACCGGCTCGTGGCTCGTACA	148526	148546	21	39
BART13-5P	BART13-star	AACCGGCTCGTGGCTCGTACt	148526	148546	21	3
BART13-5P	BART13-star	AACCGGCTCGTGGCTCGTcaA	148526	148546	21	5
BART13-5P	BART13-star	AACCGGCTCGTGGCTCGTtCA	148526	148546	21	5
BART13-5P	BART13-star	AACCGGCTCGTGGCTtGTACA	148526	148546	21	4
BART13-5P	BART13-star	AACCGGCTCGTGGCTCagACAG	148526	148547	22	8
BART13-5P	BART13-star	AACCGGCTCGTGGCTCaTACAG	148526	148547	22	3
BART13-5P	BART13-star	AACCGGCTCGTGGCTCGTACAG	148526	148547	22	35
BART13-5P	BART13-star	AACCGGCTCGTGGCTCGTACtG	148526	148547	22	4
BART13-5P	BART13-star	AACCGGCTCGTGGCTCGTAtAG	148526	148547	22	7
BART13-5P	BART13-star	AACCGGCTCGTGGCTCGaACAGA	148526	148548	23	12
BART13-5P	BART13-star	AACCGGCTCGTGGCTCGcACAGA	148526	148548	23	17
BART13-5P	BART13-star	AACCGGCTCGTGGCTCGgACAGA	148526	148548	23	23
BART13-5P	BART13-star	AACCGGCTCGTGGCTCGgACAGt	148526	148548	23	3
BART13-5P	BART13-star	AACCGGCTCGTGGCTCGTACAaA	148526	148548	23	5
BART13-5P	BART13-star	AACCGGCTCGTGGCTCGTACAGA	148526	148548	23	59
BART13-5P	BART13-star	AACCGGCTCGTGGCTCGTACgGA	148526	148548	23	4
BART13-5P	BART13-star	AACCGGCTCGTGGCTCGTACAatC	148526	148549	24	3
BART13-5P	BART13-star	AACCGGCTCGTGGCTCGTACAcAC	148526	148549	24	3
BART13-5P	BART13-star	AACCGGCTCGTGGCTCGTACAGAC	148526	148549	24	6
BART13-5P	BART13-star	AACCGGCTCGTGGCTCGTACAGct	148526	148549	24	6
BART13-3P	BART13	TGTAACTTGCCAGGGACGGa	148563	148582	20	4
BART13-3P	BART13	TGTAACTTGCCAGGGACaaCT	148563	148583	21	4
BART13-3P	BART13	TGTAACTTGCCAGGGACGGCT	148563	148583	21	3
BART13-3P	BART13	TGTAACTTGCCAGGGACGGCaG	148563	148584	22	5
BART13-3P	BART13	TGTAACTTGCCAGGGACGGCTG	148563	148584	22	4
BART13-3P	BART13	TGTAACTTGCCAGGGACaaCTGA	148563	148585	23	10
BART13-3P	BART13	TGTAACTTGCCAGGGACaGCaGA	148563	148585	23	7
BART13-3P	BART13	TGTAACTTGCCAGGGACaGCcGA	148563	148585	23	5
BART13-3P	BART13	TGTAACTTGCCAGGGACaGCTaA	148563	148585	23	4
BART13-3P	BART13	TGTAACTTGCCAGGGACaGCTGA	148563	148585	23	23
BART13-3P	BART13	TGTAACTTGCCAGGGACcGCcGA	148563	148585	23	3
BART13-3P	BART13	TGTAACTTGCCAGGGACGaCaGA	148563	148585	23	4
BART13-3P	BART13	TGTAACTTGCCAGGGACGaCTGA	148563	148585	23	68
BART13-3P	BART13	TGTAACTTGCCAGGGACGaCTGc	148563	148585	23	3
BART13-3P	BART13	TGTAACTTGCCAGGGACGaCTGg	148563	148585	23	10
BART13-3P	BART13	TGTAACTTGCCAGGGACGcCTGA	148563	148585	23	6
BART13-3P	BART13	TGTAACTTGCCAGGGACGGaaaA	148563	148585	23	4
BART13-3P	BART13	TGTAACTTGCCAGGGACGGaTGA	148563	148585	23	4
BART13-3P	BART13	TGTAACTTGCCAGGGACGGCaGA	148563	148585	23	36
BART13-3P	BART13	TGTAACTTGCCAGGGACGGCaGt	148563	148585	23	6
BART13-3P	BART13	TGTAACTTGCCAGGGACGGCcGA	148563	148585	23	3
BART13-3P	BART13	TGTAACTTGCCAGGGACGGCgaA	148563	148585	23	4
BART13-3P	BART13	TGTAACTTGCCAGGGACGGCTaA	148563	148585	23	4
BART13-3P	BART13	TGTAACTTGCCAGGGACGGCTGA	148563	148585	23	32
BART13-3P	BART13	TGTAACTTtCCAGGGACGaCTGt	148563	148585	23	3
BART13-3P	BART13	TGTAACTTGCCAGGGACGaCTGAC	148563	148586	24	3
BART13-3P	BART13	TGTAACTTGCCAGGGACGaCTGcC	148563	148586	24	3
BART13-3P	BART13	TGTAACTTGCCAGGGACGGCTGAC	148563	148586	24	26
BART13-3P	BART13	TGTAACTTGCCAGGGACGGCTGct	148563	148586	24	4
BART13-3P	BART13	TGTAACTTGCCAGGGACGGCTGACc	148563	148587	25	8
BART13-3P	BART13	TGTAACTTGCCAGGGACGGCTGACG	148563	148587	25	26
BART13-3P	BART13	TGTAACTTGCCAGGGACGGCTGACGAT	148563	148589	27	5
BART14-5P	BART14-star	TACCCTACGCTGCCGATca	148744	148762	19	3
BART14-5P	BART14-star	TACCCTACGCTGCCGATgT	148744	148762	19	7
BART14-5P	BART14-star	TACCCTACGCTGCCGATTg	148744	148762	19	7
BART14-5P	BART14-star	TACCCTACGCTGCCGATTT	148744	148762	19	11
BART14-5P	BART14-star	TACCCTACGCTGCCGAaccA	148744	148763	20	3
BART14-5P	BART14-star	TACCCTACGCTGCCGAcccA	148744	148763	20	3
BART14-5P	BART14-star	TACCCTACGCTGCCGATacA	148744	148763	20	6
BART14-5P	BART14-star	TACCCTACGCTGCCGATTTA	148744	148763	20	20
BART14-5P	BART14-star	TACCCTACGCTGCCGAccTAC	148744	148764	21	6
BART14-5P	BART14-star	TACCCTACGCTGCCGAcTTAC	148744	148764	21	4
BART14-5P	BART14-star	TACCCTACGCTGCCGATTTAC	148744	148764	21	50

BART14-5P	BART14-star	TACCCTACGCTGCCGgTTTAC	148744	148764	21	13
BART14-5P	BART14-star	TACCCTACGCTGCCGAacTACA	148744	148765	22	5
BART14-5P	BART14-star	TACCCTACGCTGCCGAcTTACA	148744	148765	22	3
BART14-5P	BART14-star	TACCCTACGCTGCCGAgTTACA	148744	148765	22	8
BART14-5P	BART14-star	TACCCTACGCTGCCGAgTTACg	148744	148765	22	4
BART14-5P	BART14-star	TACCCTACGCTGCCGATaaACA	148744	148765	22	5
BART14-5P	BART14-star	TACCCTACGCTGCCGATaTACA	148744	148765	22	4
BART14-5P	BART14-star	TACCCTACGCTGCCGATcTACA	148744	148765	22	51
BART14-5P	BART14-star	TACCCTACGCTGCCGATTcACA	148744	148765	22	3
BART14-5P	BART14-star	TACCCTACGCTGCCGATTgACA	148744	148765	22	5
BART14-5P	BART14-star	TACCCTACGCTGCCGATTTACA	148744	148765	22	27
BART14-5P	BART14-star	TACCCTACGCTGCCGATTTACg	148744	148765	22	8
BART14-5P	BART14-star	TACCCTACGCTGCCGATTaACAT	148744	148766	23	3
BART14-5P	BART14-star	TACCCTACGCTGCCGATTcACAc	148744	148766	23	4
BART14-5P	BART14-star	TACCCTACGCTGCCGATTTACAT	148744	148766	23	16
BART14-5P	BART14-star	TACCCTACGCTGCCGATTTACgTA	148744	148767	24	3
BART14-5P	BART14-star	ACCCTACGCTGCCGATcTAC	148745	148764	20	4
BART14-5P	BART14-star	ACCCTACGCTGCCGATggcC	148745	148764	20	4
BART14-5P	BART14-star	ACCCTACGCTGCCGATTTAC	148745	148764	20	38
BART14-5P	BART14-star	ACCCTACGCTGCCGATTTACA	148745	148765	21	8
BART14-5P	BART14-star	ACCCTACGCTGCCGATTTggA	148745	148765	21	3
BART14-5P	BART14-star	ACCCTACGCTGCCGATTcACtg	148745	148766	22	3
BART14-5P	BART14-star	ACCCTACGCTGCCGATTgACAT	148745	148766	22	9
BART14-5P	BART14-star	ACCCTACGCTGCCGATTTACAg	148745	148766	22	5
BART14-5P	BART14-star	CCCTACGCTGCCGATTTACAT	148746	148766	21	4
BART14-3P	BART14	TAAATGCTGCAGTAGTAGGG	148778	148797	20	4
BART14-3P	BART14	TAAATGCTGCAGTAGTAGGGA	148778	148798	21	10
BART14-3P	BART14	TAAATGCTGCAGTAGTAGGaAT	148778	148799	22	4
BART14-3P	BART14	TAAATGCTGCAGTAGTAGGGAa	148778	148799	22	10
BART14-3P	BART14	TAAATGCTGCAGTAGTAGGGAT	148778	148799	22	13
BART14-3P	BART14	TAAATGCTGCAGTAGTAGGGAaa	148778	148800	23	3
BART14-3P	BART14	TAAATGCTGCAGTAGTAGGGATa	148778	148800	23	3
BART14-3P	BART14	TAAATGCTGCAGTAGTAGGGATC	148778	148800	23	9
BART14-3P	BART14	TAAATGCTGCAGTAGTAGGGATt	148778	148800	23	8
BART14-3P	BART14	TAAATGCTGCAGTAGTAGGtcaC	148778	148800	23	8
BART14-3P	BART14	TAAATGCTGCAGTAGTAGGGATCa	148778	148801	24	3
BART14-3P	BART14	TAAATGCTGCAGTAGTAGGGATCT	148778	148801	24	5
BART14-3P	BART14	AAATGCTGCAGTAGTtGGGAT	148779	148799	21	13
BART2-5P	BART2-5P	TATTTTCTGCATTCaCCCT	152747	152765	19	3
BART2-5P	BART2-5P	TATTTTCTGCATTCGCCCT	152747	152765	19	9
BART2-5P	BART2-5P	TATTTTCTGCATTCaCCCTT	152747	152766	20	6
BART2-5P	BART2-5P	TATTTTCTGCATTCGCCCaT	152747	152766	20	10
BART2-5P	BART2-5P	TATTTTCTGCATTCGCCCcT	152747	152766	20	4
BART2-5P	BART2-5P	TATTTTCTGCATTCGCCCTT	152747	152766	20	35
BART2-5P	BART2-5P	TATTTTCTGCATTCGCCgTT	152747	152766	20	7
BART2-5P	BART2-5P	TATTTTCTGCATTCGCCtgT	152747	152766	20	3
BART2-5P	BART2-5P	TATTTTCTGCATTCGCCtTT	152747	152766	20	13
BART2-5P	BART2-5P	TATTTTCTGCATTCGaCCTTG	152747	152767	21	39
BART2-5P	BART2-5P	TATTTTCTGCATTCGCaCTTG	152747	152767	21	3
BART2-5P	BART2-5P	TATTTTCTGCATTCGCCCgaa	152747	152767	21	7
BART2-5P	BART2-5P	TATTTTCTGCATTCGCCCTac	152747	152767	21	6
BART2-5P	BART2-5P	TATTTTCTGCATTCGCCCTgc	152747	152767	21	6
BART2-5P	BART2-5P	TATTTTCTGCATTCGCCCTTa	152747	152767	21	19
BART2-5P	BART2-5P	TATTTTCTGCATTCGCCCTTG	152747	152767	21	104
BART2-5P	BART2-5P	TATTTTCTGCATTCGtCCTTG	152747	152767	21	17
BART2-5P	BART2-5P	TATTTTCTGCATTCGCaCaTGC	152747	152768	22	5
BART2-5P	BART2-5P	TATTTTCTGCATTCGCaCTTGC	152747	152768	22	57
BART2-5P	BART2-5P	TATTTTCTGCATTCGCCaTTGC	152747	152768	22	18
BART2-5P	BART2-5P	TATTTTCTGCATTCGCCCagaC	152747	152768	22	11
BART2-5P	BART2-5P	TATTTTCTGCATTCGCCCaTGC	152747	152768	22	44
BART2-5P	BART2-5P	TATTTTCTGCATTCGCCCccGC	152747	152768	22	8
BART2-5P	BART2-5P	TATTTTCTGCATTCGCCCcTGC	152747	152768	22	47
BART2-5P	BART2-5P	TATTTTCTGCATTCGCCCgTGC	152747	152768	22	5
BART2-5P	BART2-5P	TATTTTCTGCATTCGCCCTaGC	152747	152768	22	19
BART2-5P	BART2-5P	TATTTTCTGCATTCGCCCTcGa	152747	152768	22	42
BART2-5P	BART2-5P	TATTTTCTGCATTCGCCCTcGC	152747	152768	22	19
BART2-5P	BART2-5P	TATTTTCTGCATTCGCCCTgat	152747	152768	22	7
BART2-5P	BART2-5P	TATTTTCTGCATTCGCCCTgGC	152747	152768	22	6

BART2-5P	BART2-5P	TATTTTCTGCATTCGCCCTTaC	152747	152768	22	12
BART2-5P	BART2-5P	TATTTTCTGCATTCGCCCTTag	152747	152768	22	5
BART2-5P	BART2-5P	TATTTTCTGCATTCGCCCTTGa	152747	152768	22	6
BART2-5P	BART2-5P	TATTTTCTGCATTCGCCCTTGC	152747	152768	22	605
BART2-5P	BART2-5P	TATTTTCTGCATTCGCCCTTGt	152747	152768	22	6
BART2-5P	BART2-5P	TATTTTCTGCATTCGCCCTTta	152747	152768	22	10
BART2-5P	BART2-5P	TATTTTCTGCATTCGCCCTTtC	152747	152768	22	4
BART2-5P	BART2-5P	TATTTTCTGCATTCGCCgaTGC	152747	152768	22	7
BART2-5P	BART2-5P	TATTTTCTGCATTCGCCgTTGC	152747	152768	22	9
BART2-5P	BART2-5P	TATTTTCTGCATTCGCCtTTGC	152747	152768	22	4
BART2-5P	BART2-5P	TATTTTCTGCATTCGCgCTTGC	152747	152768	22	13
BART2-5P	BART2-5P	TATTTTCTGCATTCGCtCTTGC	152747	152768	22	34
BART2-5P	BART2-5P	TATTTTCTGCgccCGCCCTTGC	152747	152768	22	7
BART2-5P	BART2-5P	TATTTTCTGCgccCGCCCTTGt	152747	152768	22	3
BART2-5P	BART2-5P	TATTTTCTGCATTCGCCCacaCa	152747	152769	23	3
BART2-5P	BART2-5P	TATTTTCTGCATTCGCCCaTaCt	152747	152769	23	7
BART2-5P	BART2-5P	TATTTTCTGCATTCGCCCgaGCa	152747	152769	23	4
BART2-5P	BART2-5P	TATTTTCTGCATTCGCCCTaGCa	152747	152769	23	3
BART2-5P	BART2-5P	TATTTTCTGCATTCGCCCTTaCG	152747	152769	23	4
BART2-5P	BART2-5P	TATTTTCTGCATTCGCCCTTaCt	152747	152769	23	3
BART2-5P	BART2-5P	TATTTTCTGCATTCGCCCTTcgc	152747	152769	23	8
BART2-5P	BART2-5P	TATTTTCTGCATTCGCCCTTGaa	152747	152769	23	3
BART2-5P	BART2-5P	TATTTTCTGCATTCGCCCTTGac	152747	152769	23	10
BART2-5P	BART2-5P	TATTTTCTGCATTCGCCCTTGat	152747	152769	23	13
BART2-5P	BART2-5P	TATTTTCTGCATTCGCCCTTGCc	152747	152769	23	3
BART2-5P	BART2-5P	TATTTTCTGCATTCGCCCTTGCG	152747	152769	23	28
BART2-5P	BART2-5P	TATTTTCTGCATTCGCCCTTGCt	152747	152769	23	13
BART2-5P	BART2-5P	TATTTTCTGCATTCGCCCTTGgc	152747	152769	23	9
BART2-5P	BART2-5P	TATTTTCTGCATTCGCCCTTGCaT	152747	152770	24	3
BART2-5P	BART2-5P	TATTTTCTGCATTCGCCCTTGCGg	152747	152770	24	3
BART2-5P	BART2-5P	TATTTTCTGCATTCGCCCTTGCGT	152747	152770	24	6
BART2-5P	BART2-5P	TATTTTCTGCATTCGCCCTTGtaT	152747	152770	24	3
BART2-5P	BART2-5P	TATTTTCTGCATTCGCCCTTGCGTGcCC	152747	152774	28	4
BART2-5P	BART2-5P	TATTTTCTGCATTCGCCCTTGCGTGTCC	152747	152774	28	4
BART2-5P	BART2-5P	TATTTTCTGCATTCGCCCTTGCGTGTtC	152747	152774	28	3
BART2-5P	BART2-5P	ATTTTCTGCATTCGCCCTT	152748	152766	19	5
BART2-5P	BART2-5P	ATTTTCTGCATTCGCCCTTa	152748	152767	20	4
BART2-5P	BART2-5P	ATTTTCTGCATTCGCCCaTGC	152748	152768	21	3
BART2-5P	BART2-5P	ATTTTCTGCATTCGCCCTTcC	152748	152768	21	4
BART2-5P	BART2-5P	ATTTTCTGCATTCGCCCTTGC	152748	152768	21	9
BART2-5P	BART2-5P	ATTTTCTGCATTCGCtCTTta	152748	152768	21	4
BART2-3P	BART2-3P	AAGGAGCGATTTGGAGAAAATA	152783	152804	22	4
BART2-3P	BART2-3P	AAGGAGCGATTTGGAGAAAATAA	152783	152805	23	8
