# Supplementary material for: Characterization of Epstein-Barr Virus miRNAome in Nasopharyngeal Carcinoma by Deep Sequencing
Source: PLoS One. 2010 Sep 20;5(9):e12745. doi: 10.1371/journal.pone.0012745 (PMC2942828; doi:10.1371/journal.pone.0012745)
Supplement: Table S4 — Major EBV miRNAs detected by SOLiD sequencing in T10 and N10 samples. (0.04 MB PDF) [file pone.0012745.s007.pdf]

Table S4. Major EBV miRNAs detected by SOLiD sequencing in T10 and N10 samples [a]

EBV miRNA	miRBase Name	T10 total [b]	T10 reads [c]	%	N10 [d]	Sequence [e]	Start [f]	End [f]	Length	Note [g]
BART3-5P	BART3-star	245	43	18%	3	AACCTAGTGTTAGTGTTGTGC	139086	139106	21	(-1,-1)
			31	13%		ACCTAGTGTTAGTGTTGTGCTGT	139087	139109	23	
BART3-3P	BART3	17990	6064	34%	120	CGCACCACTAGTCACCAGGTGT	139124	139145	22	RefSeq
			712	4%		CGCACCACTAGTCACCAGaTGT	139124	139145	22	
			630	4%		CGCACCACTAGTCACCAGGTGTC	139124	139146	23	
			605	3%		CGCACCACTAGTCACCgGGTGT	139124	139145	22	
BART4-5P	BART4	9204	1622	18%	33	GACCTGATGCTGCTGGTGTGCT	139228	139249	22	RefSeq
			1159	13%		GACCTGATGCTGCTGGTGTGC	139228	139248	21	
			829	9%		GACCTGATGCTGCTGGTGTGCTG	139228	139250	23	
BART4-3P	BART4-star	128	66	52%		CACATCACGTAGGCACCAGGTGT	139266	139288	23	RefSeq
			20	16%		CACATCACGTAGGCACCAGGTG	139266	139287	22	
BART1-5P	BART1-5P	462	18	4%	8	TCTTAGTGGAAGTGACGTGCTGTG	139351	139374	24	RefSeq
BART1-3P	BART1-3P	5551	1204	22%		TAGCACCGCTATCCACTATGT	139387	139407	21	
			881	16%		TAGCACCGCTATCCACTATGTCT	139387	139409	23	
			674	12%	3	TAGCACCGCTATCCACTATGTC	139387	139408	22	RefSeq
BART15-5P		3	3	100%		AGGGAAACATGACCACCTGAAGTC	139519	139542	24	novel
BART15-3P	BART15	1894	1212	64%	50	GTCAGTGGTTTTGTTTCCTTGA	139553	139574	22	RefSeq
BART5-5P	BART5	15654	2601	17%	64	CAAGGTGAATATAGCTGCCCATCG	139675	139698	24	RefSeq
			1228	8%		CAAGGTGAATATAGCTGCCCATC	139675	139697	23	
			1027	7%		CAAGGTGAATATAGCTGCCCATCGA	139675	139699	25	
			929	6%		CAAGGTGAATATAGCTGCCCAT	139675	139696	22	
			791	5%		AAGGTGAATATAGCTGCCCATCG	139676	139698	23	
			651	4%		AAGGTGAATATAGCTGCCCATCGA	139676	139699	24	
BART5-3P	BART5-star	300	110	37%		GTGGGCCGCTGTTCACCTAA	139717	139736	20	(0,2)
			46	15%		GTGGGCCGCTGTTCACCTAAA	139717	139737	21	
BART16-5P	BART16	121	23	19%		TTAGATAGAGTGGGTGTGTGC	139795	139815	21	(0,-2)
			18	15%		TTAGATAGAGTGGGTGTGTGCTCTTaTT	139795	139822	28	
			9	7%		TTAGATAGAGTGGGTGTGTGCTC	139795	139817	23	
BART16-3P		705	199	28%		ATCACCACCCTCTATCCATAT	139836	139856	21	novel
			120	17%		AGATCACCACCCTCTATCCAT	139834	139854	21	
			72	10%		AGATCACCACCCTCTATCCATAT	139834	139856	23	
BART17-5P	BART17-3P	194	63	33%		TAAGAGGACGCAGGCATAC	139915	139933	19	(0,-2)
			38	20%		TAAGAGGACGCAGGCATACAA	139915	139935	21	
			38	20%		TAAGAGGACGCAGGCATACAA	139915	139935	21	
			34	18%		TAAGAGGACGCAGGCATACA	139915	139934	20	
BART17-3P	BART17-3P	1568	664	42%	9	TGTATGCCTGGTGTCCCCTTAGT	139953	139975	23	RefSeq
BART6-5P	BART6-5P	1482	709	48%		TAAGGTTGGTCCAATCCATAGGC	140033	140055	23	
			15	1%		TAAGGTTGGTCCAATCCATAGG	140033	140054	22	RefSeq
BART6-3P	BART6-3P	1865	430	23%	9	CGGGGATCGGACTAGCCTTAGA	140072	140093	22	RefSeq
			195	11%		CGGGGATCGGACTAGCCTTAG	140072	140092	21	
BART21-5P	BART21-5P	159	58	37%		TCACTAGTGAAGGCAACTAACA	145514	145535	22	
			36	23%		TCACTAGTGAAGGCAACTAAC	145514	145534	21	RefSeq
BART21-3P	BART21-3P	16	9	56%		CTAGTTGTGCCCACTGGTGTTT	145548	145569	22	RefSeq
			4	25%		CTAGTTGTGCCCACTGGTGcaT	145548	145569	22	

			3	19%		CTAGTTGTGCCCACTGGTGaaT	145548	145569	22	
BART18-5P	BART18-5P	1853	296	16%		TCAAGTTCGCACTTCCTATAC	145962	145982	21	
			290	16%	4	TCAAGTTCGCACTTCCTATACA	145962	145983	22	RefSeq
			290	16%		TCAAGTTCGCACTTCCTATACA	145962	145983	22	
BART18-3P	BART18-3P	44	8	18%		TATCGGAAGTTTGGGCTTCGTC	145998	146019	22	RefSeq
			6	14%		TATCGGAAGTTTGGGCTTCaTC	145998	146019	22	
			6	14%		TATCGGAAGTTTGGGCTTCGT	145998	146018	21	
			5	11%		TATCGGAAGTTTGGGCTcCGT	145998	146018	21	
BART7-5P	BART7-star	276	112	41%		CCTGGACCTTGACTATGAAACA	146439	146460	22	RefSeq
			51	19%		CCTGGACCTTGACTATGAAAC	146439	146459	21	
BART7-3P	BART7	8313	573	7%		CATCATAGTCCAGTGTCCAG	146475	146494	20	
			539	7%		CATCATAGTCCAGTGTCCAGGGA	146475	146497	23	
			197	2%	5	CATCATAGTCCAGTGTCCAGGG	146475	146496	22	RefSeq
BART8-5P	BART8	2498	1532	61%	49	TACGGTTTCCTAGATTGTACAG	146772	146793	22	RefSeq
BART8-3P	BART8-star	4869	1499	31%	26	GTCACAATCTATGGGGTCGTAGA	146807	146829	23	RefSeq
BART9-5P	BART9-star	191	29	15%		TACTGGACCCTGAATTGGAAAC	146959	146980	22	RefSeq
			23	12%		TACTGGACCCTGAATTGGAAACA	146959	146981	23	
			21	11%		TACTGGACCCTGAATTGGAAA	146959	146979	21	
			20	11%		TACTGGACCCTGAATTGaAAACA	146959	146981	23	
BART9-3P	BART9	16398	5266	32%	77	TAACACTTCATGGGTCCCGTAGT	146997	147019	23	RefSeq
			1066	7%		TAACACTTCATGGGTCCCGTAG	146997	147018	22	
			825	5%		TAACACTTCATGGGTCCCGTAGTG	146997	147020	24	
			608	4%		TAACACTTCATGGGTCCCGTAa	146997	147018	22	
			503	3%		TAACACTTCATGGGTCCCGTAGc	146997	147019	23	
BART22-5P		10	5	50%	1	TGCTAGACCCTGGAGTTGAACC	147169	147190	22	novel
BART22-3P	BART22	534	167	31%	6	TTACAAAGTCATGGTCTAGTAGT	147203	147225	23	RefSeq
			83	16%		TTACAAAGTCATGGTCTAGTAG	147203	147224	22	
BART10-5P	BART10-star	158	81	51%	3	GCCACCTCTTTGGTTCTGTAC	147321	147341	21	(0,-1)
			40	25%		GCCACCTCTTTGGTTCcGTA	147321	147340	20	
			17	11%		GCCACCTCTTTGGTTCaGTAC	147321	147341	21	
BART10-3P	BART10	3633	449	12%		TACATAACCATGGAGTTGaCTGT	147356	147378	23	
			406	11%		ACATAACCATGGAGTTGaCTGT	147357	147378	22	
			171	5%	4	TACATAACCATGGAGTTGGCTGT	147356	147378	23	RefSeq
BART11-5P	BART11-3P	350	89	25%		TCAGACAGTTTGGTGCGCTAGTT	147537	147559	23	
			60	17%		TCAGACAGTTTGGTGCGCTctg	147537	147558	22	
			30	9%	1	TCAGACAGTTTGGTGCGCTAGTTG	147537	147560	24	RefSeq
BART11-3P	BART11-3P	918	159	17%		ACGCACACCAGGCTGACTGCCT	147575	147596	22	
			145	16%		ACGCACACCAGGCTGACTGC	147575	147594	20	
			70	8%	1	ACGCACACCAGGCTGACTGCC	147575	147595	21	RefSeq
BART12-5P		82	19	23%		ACCCGCCCATCACCACCGGAC	147901	147921	21	novel
			12	15%		gACCCGCCCATCACCACCaGA	147900	147920	21	
			11	13%		gACCCGCCCATCACCACCGGACAG	147900	147923	24	
BART12-3P	BART12	142	25	18%		TCCTGTGGTGTTTGGTGTGGTTT	147936	147958	23	
			16	11%		TCCTGTGGTGTTTGGTGTGGTT	147936	147957	22	RefSeq
			16	11%		TCCTGTGGTGTTTGGTGTGGTTTa	147936	147959	24	
BART19-5P	BART19-5P	7713	1842	24%		ACATTCCCCGCAAACAcGACAT	148215	148236	22	
			966	13%		ACATTCCCCGCAAACAcGACA	148215	148235	21	

			633	8%		ACATTCCCCGCAAACAcGACATG	148215	148237	23	
			13	0%		ACATTCCCCGCAAACATGACATG	148215	148237	23	RefSeq
BART19-3P	BART19-3P	36	12	33%		TTTTGTTTGCTTGGGAATGCTC	148254	148275	22	
			4	11%		TGTTTTGTTTGCTTGGGAATGCc	148252	148274	23	
			4	11%		TGTTTTGTTTGCTTGGGAATGCT	148252	148274	23	
			4	11%		TGTTTTGTTTGCTTGGGAATGCTa	148252	148275	24	
			3	8%		TTTTGTTTGCTTGGGAATGCT	148254	148274	21	RefSeq
BART20-5P	BART20-5P	6	3	50%		TAGCAGGCATGTCTTCATTCC	148339	148359	21	RefSeq
			3	50%		TAGCAGGCATGTCTTCcTca	148339	148358	20	
BART20-3P	BART20-3P	166	99	60%	4	CATGAAGGCACAGCCTGTTACC	148374	148395	22	RefSeq
			20	12%		CATGAAGGCACAGCCTaTTACC	148374	148395	22	
BART13-5P	BART13-star	333	59	18%		AACCGGCTCGTGGCTCGTACAGA	148526	148548	23	
			39	12%		AACCGGCTCGTGGCTCGTACA	148526	148546	21	
			35	11%	1	AACCGGCTCGTGGCTCGTACAG	148526	148547	22	RefSeq
BART13-3P	BART13	334	68	20%		TGTAACTTGCCAGGGACGaCTGA	148563	148585	23	
			36	11%		TGTAACTTGCCAGGGACGGCaGA	148563	148585	23	
			32	10%	1	TGTAACTTGCCAGGGACGGCTGA	148563	148585	23	RefSeq
BART14-5P	BART14-star	360	51	14%		TACCCTACGCTGCCGATcTACA	148744	148765	22	
			50	14%		TACCCTACGCTGCCGATTTAC	148744	148764	21	
			38	11%		ACCCTACGCTGCCGATTTAC	148745	148764	20	
			27	8%	1	TACCCTACGCTGCCGATTTACA	148744	148765	22	RefSeq
BART14-3P	BART14	93	13	14%		TAAATGCTGCAGTAGTAGGGAT	148778	148799	22	RefSeq
			13	14%		AAATGCTGCAGTAGTtGGGAT	148779	148799	21	
			10	11%		TAAATGCTGCAGTAGTAGGGA	148778	148798	21	
			10	11%		TAAATGCTGCAGTAGTAGGGAa	148778	148799	22	
BART2-5P	BART2-5P	1470	605	41%	6	TATTTTCTGCATTCGCCCTTGC	152747	152768	22	RefSeq
BART2-3P	BART2-3P	12	8	67%		AAGGAGCGATTTGGAGAAAATAA	152783	152805	23	(0,-1)
			4	33%		AAGGAGCGATTTGGAGAAAATA	152783	152804	22	

a : Only sequences matched to miRBase RefSeq and major isomiRs (> 10% or > 500 reads) are included.
b : Total reads in the T10 sample mapping to start sites within ± 2 nts on the 5' end of the mature miRNA, minimum 19-nt in length
c : Exact reads detected in the T10 sample
d : Exact reads detected in the N10 sample
e : 5' to 3', nucleotides in lowercase denote sequence deviation from the reference EBV genome (NC_007605).
f : Position according to the EBV genome (NC_007605).
g : Number in parathesis indicates offset from miRBase RefSeq position at 5' and 3' ends.
