# Supplementary material for: Characterization of Epstein-Barr Virus miRNAome in Nasopharyngeal Carcinoma by Deep Sequencing
Source: PLoS One. 2010 Sep 20;5(9):e12745. doi: 10.1371/journal.pone.0012745 (PMC2942828; doi:10.1371/journal.pone.0012745)
Supplement: Table S5 — Expression levels of EBV miRNAs detected by RT-PCR in 13 NPC tissues and c666-1 cells. (0.02 MB PDF) [file pone.0012745.s008.pdf]

Table S5. Expression levels of EBV miRNAs detected by RT-PCR in 13 NPC tissues and c666-1 cells [a]

EBV miRNA	miRBase Name	CT2	CT4	CT5	CT6	CT7	CT8	CT10	CT11	CT13	CT17	CT18	CT20	CT21	c666-1
BART10-3P	BART10	9.65	12.34	10.40	11.95	11.35	12.13	9.01	11.94	7.21	10.89	12.29	8.73	11.49	14.38
BART10-5P	BART10-star	10.63	11.59	11.04	11.97	11.08	12.24	10.13	12.22	7.76	10.76	12.92	10.20	12.42	12.59
BART11-3P	BART11-3p	13.96	12.77	13.85	13.87	13.07	15.25	12.49	14.58	12.32	13.87	14.87	12.67	15.01	15.24
BART11-5P	BART11-5p	10.86	11.67	11.70	11.82	10.56	11.85	8.89	11.90	8.40	11.11	12.14	9.59	12.38	13.97
BART13-3P	BART13	8.75	10.65	10.51	11.80	9.96	11.86	9.46	11.97	7.24	15.30	10.98	8.69	11.84	12.78
BART1-3P	BART1-3p	9.96	12.94	12.13	13.83	12.64	14.43	12.11	11.75	6.90	12.27	13.40	10.34	13.30	14.87
BART14-5P	BART14-star	7.54	9.48	10.32	11.47	9.99	11.44	9.61	8.52	6.18	9.83	10.49	8.20	10.97	11.25
BART1-5P	BART1-5p	11.52	13.44	13.54	14.42	13.80	13.96	11.90	13.53	9.35	13.08	14.55	11.79	13.98	15.98
BART16-5P	BART16	7.97	8.68	9.99	10.32	11.35	13.12	9.62	9.81	7.27	10.78	10.87	8.77	11.07	13.90
BART17-3P	BART17-3p	12.42	13.27	14.80	15.93	14.85	15.27	13.37	15.85	8.98	14.62	14.34	13.33	15.43	16.75
BART18-3P	BART18-3p	3.91	5.51	3.84	5.58	5.28	5.57	5.15	4.16	4.14	3.01	4.79	2.65	4.67	6.17
BART19-5P	BART19-5p	12.50	13.15	12.80	9.62	12.44	14.06	12.24	12.14	10.35	12.75	13.53	10.41	14.14	15.09
BART20-3P	BART20-3p	4.51	4.91	4.75	4.90	4.35	4.74	7.51	2.42	1.79	2.92	5.47	1.89	3.04	12.64
BART20-5P	BART20-5p	4.04	6.15	6.37	8.07	5.82	7.41	4.47	4.93	2.56	4.64	7.38	4.66	7.44	9.90
BART2-3P	BART2-3p	2.58	4.73	4.60	4.78	4.22	4.53	4.91	3.53	0.20	3.83	5.71	1.88	4.59	6.04
BART3-3P	BART3	11.00	14.17	11.75	13.42	12.86	13.90	11.49	13.86	10.77	11.92	13.58	10.18	12.80	13.33
BART3-5P	BART3-star	9.32	11.32	10.20	11.47	11.07	11.82	8.32	10.40	6.71	10.45	11.86	8.61	11.39	14.14
BART4-5P	BART4	15.34	17.72	16.58	17.61	17.38	18.43	15.64	18.53	14.87	16.74	18.08	15.51	17.98	17.75
BART5-5P	BART5	11.02	13.04	12.47	13.65	12.70	13.65	11.01	12.81	7.73	12.16	13.43	10.78	9.01	15.69
BART6-3P	BART6-3p	11.67	15.53	13.96	15.35	14.51	14.84	13.72	16.62	11.09	13.97	14.61	12.90	15.49	14.64
BART6-5P	BART6-5p	9.49	12.86	11.73	12.93	12.37	12.97	9.88	12.64	6.96	11.82	12.93	10.08	12.75	14.36
BART7-3P	BART7	14.56	14.95	15.51	16.60	16.41	16.98	15.49	15.98	10.70	15.33	16.25	13.86	16.49	18.14
BART7-5P	BART7-star	6.95	9.07	7.88	8.91	8.39	9.52	7.60	7.95	3.68	7.54	9.62	5.41	8.95	10.28
BART9-3P	BART9	14.36	15.69	15.93	16.87	15.84	16.56	15.49	16.60	10.60	15.52	16.90	14.14	16.79	18.31
BART9-5P	BART9-star	8.39	10.10	9.37	9.75	9.69	9.88	8.97	8.60	8.01	9.18	10.08	8.00	9.58	10.99

a : Expression data expressed in 39 - Ct.
